# Analysis of rice glycosyl hydrolase family 1 and expression of Os4bglu12 β-glucosidase

**DOI:** 10.1186/1471-2229-6-33

**Published:** 2006-12-29

**Authors:** Rodjana Opassiri, Busarakum Pomthong, Tassanee Onkoksoong, Takashi Akiyama, Asim Esen, James R Ketudat Cairns

**Affiliations:** 1 Institute of Science, Suranaree University of Technology, Nakhon Ratchasima 30000, Thailand; 2 Department of Low-Temperature Science, National Agricultural Research Center for Hokkaido Region, Sapporo 062-8555, Japan; 3 Department of Biology, Virginia Polytechnic Institute and State University, Blacksburg, VA 24061-0406, USA

## Abstract

**Background:**

Glycosyl hydrolase family 1 (GH1) β-glucosidases have been implicated in physiologically important processes in plants, such as response to biotic and abiotic stresses, defense against herbivores, activation of phytohormones, lignification, and cell wall remodeling. Plant GH1 β-glucosidases are encoded by a multigene family, so we predicted the structures of the genes and the properties of their protein products, and characterized their phylogenetic relationship to other plant GH1 members, their expression and the activity of one of them, to begin to decipher their roles in rice.

**Results:**

Forty GH1 genes could be identified in rice databases, including 2 possible endophyte genes, 2 likely pseudogenes, 2 gene fragments, and 34 apparently competent rice glycosidase genes. Phylogenetic analysis revealed that GH1 members with closely related sequences have similar gene structures and are often clustered together on the same chromosome. Most of the genes appear to have been derived from duplications that occurred after the divergence of rice and *Arabidopsis thaliana *lineages from their common ancestor, and the two plants share only 8 common gene lineages. At least 31 GH1 genes are expressed in a range of organs and stages of rice, based on the cDNA and EST sequences in public databases. The cDNA of the *Os4bglu12 *gene, which encodes a protein identical at 40 of 44 amino acid residues with the N-terminal sequence of a cell wall-bound enzyme previously purified from germinating rice, was isolated by RT-PCR from rice seedlings. A thioredoxin-Os4bglu12 fusion protein expressed in *Escherichia coli *efficiently hydrolyzed β-(1,4)-linked oligosaccharides of 3–6 glucose residues and laminaribiose.

**Conclusion:**

Careful analysis of the database sequences produced more reliable rice GH1 gene structure and protein product predictions. Since most of these genes diverged after the divergence of the ancestors of rice and *Arabidopsis thaliana*, only a few of their functions could be implied from those of GH1 enzymes from *Arabidopsis *and other dicots. This implies that analysis of GH1 enzymes in monocots is necessary to understand their function in the major grain crops. To begin this analysis, Os4bglu12 β-glucosidase was characterized and found to have high exoglucanase activity, consistent with a role in cell wall metabolism.

## Background

β-glucosidases (3.2.1.21) are glycosyl hydrolases that hydrolyze the β-O-glycosidic bond at the anomeric carbon of a glucose moiety at the nonreducing end of a carbohydrate or glycoside molecule. These enzymes are found essentially in all living organisms and have been implicated in a diversity of roles, such as biomass conversion in microorganisms [1] and activation of defense compounds [2,3], phytohormones [4,5], lignin precursors [6], aromatic volatiles [7], and metabolic intermediates by releasing glucose blocking groups from the inactive glucosides in plants [8]. To achieve specificity for these various functions, β-glucosidases must bind to a wide variety of aglycones, in addition to the glucose of the substrate.

The β-glucosidases that have been characterized to date fall predominantly in glycosyl hydrolase families 1 and 3 [9], with family 1 enzymes being more numerous in plants. Glycosyl hydrolase family 1 (GH1) contains a wide range of β-glycosidases, including β-galactosidases, β-mannosidases, phospho-β-galactosidases, phospho-β-glucosidases, and thioglucosidases, in addition to β-glucosidases. The plant enzymes in this family generally fall in a closely related subfamily, but, despite their high sequence similarity, display a wide range of activities. Besides β-glucosidases with diverse specificities, these plant enzymes include thio-β-glucosidases or myrosinases, β-mannosidases, disaccharidases, such as primeverosidase and furcatin hydrolase, and hydroxyisourate hydrolase, which hydrolyzes an internal bond in a purine ring, rather than a glycosidic linkage [7,9-11]. In addition, many enzymes in this group are capable of releasing multiple kinds of sugars from aglycones, such as isoflavonoid β-glucosidases, which can release the disaccharide acuminose and malonyl glucose, in addition to glucose itself, from isoflavonoids [12,13]. Other β-glucosidases in this subfamily may have high specificity for glucosides or glucosides and fucosides, or may hydrolyze other glycosides, such as β-galactosides, β-mannosides, and β-xylosides, as well. Primeverosidase has high specificity for primeverosides, with no hydrolysis of glucosides [7], while furcatin hydrolase can hydrolyze glucosides as well as disaccharide glycosides [10]. Clearly, plant family 1 glycosyl hydrolases show a range of sugar specificities.

Plant family 1 glycosyl hydrolases tend to show high specificity for their aglycones, though many hydrolyze synthetic, nonphysiological substrates, like *p*-nitrophenol (*p*NP)-β-glycosides [14]. The aglycones span a wide range of structures, including sugars [15-17], hydroxaminic acids [18], isoflavonoids [12,13], rotenoids [19], alkaloids [20,21] hydroxyquinones [3], cyanogenic nitriles [2], etc. It is the specificity for these aglycones which is thought to specify the function of most of these enzymes [14]. Since many β-glucosidases function in plants, it is important that these enzymes specifically hydrolyze their own substrates and not other substrates with which they may come into contact. It seems evident that the substrate specificity, localization of the enzymes with respect to potential substrates, and the activities of the substrates and hydrolysis products will determine the roles of these enzymes.

Xu *et al*. [22] described 47 GH1 genes in the *Arabidopsis *genome, including 7 apparent thioglucosidases, and one enzyme that had high β-mannosidase activity, in agreement with the prediction from its similarity to tomato β-mannosidase. With the completion of high quality drafts of the rice genome, a thorough analysis of GH1 can be conducted in rice. To date, only a few rice β-glucosidase isozymes have been functionally characterized, with the activities described being hydrolysis of gibberellin glucosides, pyridoxine glucosides and oligosaccharides [16,17,23,24].

To assess the functions of GH1 in rice, genes homologous to GH1 β-glucosidase genes have been identified from the rice genome, and their structures, predicted protein products and evidence of expression evaluated. In addition, we have cloned a β-glucosidase from germinating rice based on genomic data, and assessed its biochemical properties after expression in *E. coli*.

## Results and discussion

### Glycosyl hydrolase family 1 β-glucosidase family

The completion of the *Oryza sativa *L. spp. *japonica *Rice Genome Project and the complementary *indica *rice (*O. sativa *L. spp. *indica*) genome project by the Beijing Genomic Institute (BGI) has allowed genome-wide analysis of gene families in this important crop [25,26]. The sequence and mapping information provided to the public databases by these projects enabled us to identify the genes for glycosyl hydrolase family 1 members (putative β-glucosidases) in rice, determine their gene structures and genomic organization, and model their protein products and phylogenetic relationships. In this study, we used the DNA sequences of *japonica *rice in the Monsanto Rice Genome Sequencing Project, the Torrey Mesa Research Institute and GenBank at NCBI and the *indica *rice sequences of the BGI as the starting point to examine the sequences homologous to GH1 members by manual annotation. By examination of the gene structures and prediction based on the knowledge of other plant GH1 genes, we rectified any errors in gene structures from the automatic annotation by the Rice Genome Sequencing Project contigs. Thereafter, the GH1 members of *indica *rice were compared with those of *japonica *rice to identify which genes are orthologues (see Table 1). Finally, all contig sequences were searched against the completed sequences of the 12 rice chromosomes in GenBank to map each contig position on the chromosomes and identify the new GH1 members that were not present in the other databases. A new systematic code for the genes based on their chromosome location was devised with the chromosome number followed by a bglu number counting from the top of chromosome 1 through the bottom of chromosome 12 (Table 1). To avoid confusion, previously published synonyms for all family members are provided in Table 1. The retrieved gene sequences were searched against the dbEST and *japonica *rice full-length cDNA databases to determine the mRNA expression patterns of each gene in rice.

Forty β-glucosidase genes, including 34 full-length genes, 2 pseudogenes, 2 gene fragments, and 2 intronless genes, were identified, as listed in Table 1. Thirty-six out of 40 genes are found in both *japonica *and *indica *rice with 98–100% sequence identity. The *Os11bglu35 *gene was present only in *japonica *rice sequences, while *Os11bglu37*, *Osbglu39 *and *Osbglu40 *were only found in *indica *rice. The thirty-eight mapped GH1 genes are distributed over all chromosomes, except chromosome 2 (Table 1). The *Osbglu39 *and *Osbglu40 *sequences have not been mapped to any chromosome, and it is possible they represent contamination of endophytic genes remaining in the *indica *genome draft. Twenty-two out of 40 gene sequences are derived from the automated annotation in the public databases and 18 genes are derived from manual annotation. We corrected 4 of 22 automated annotation contigs that had misassigned one or more intron-exon boundaries. *Os11bglu35 *and *Os11bglu37 *appear to be pseudogenes, since they have premature stop codons and cannot produce full-length proteins.

The size of rice GH1 is not unexpected, since a search of the *Arabidopsis thaliana *genome identified 47 glycosyl hydrolase family 1 homologues, including 8 probable pseudogenes and 3 intronless genes, which are distributed throughout all five chromosomes [22]. The slightly larger size of the family in *Arabidopsis *may be due to the presence of myrosinases, which are not found in rice, and a larger number of pseudogenes. The large size of both rice and *Arabidopsis *GH1 may reflect different substrate specificity and expression patterns in rice tissues and/or in response to environmental conditions among the GH1 members.

The presence of many GH1 genes in rice suggests they may hydrolyze an array of possible substrates, depending on their substrate specificity and localization with respect to the substrates. Although a number of glycosides that could serve as potential substrates for rice GH 1 β-glucosidases have been purified from rice tissues, there have been few reports about the hydrolysis of these substrates by the enzymes. The major glycosides found in various tissues of rice include glycosylsterols, flavonoid glucosides, hormone glucosides, a vitamin glucoside, and pantonic acid glucoside. Glycosylsterols found in rice are glycosyl-sitosterol, -campesterol and -stigmasterol in rice bran [27] and β-sitosterol-3-O-β-D-glucoside in rice hulls [28]. The major flavonoid glucosides present in rice include 1) anthocyanins, such as cyanidin-O-β-D-glucoside and peonidin-O-β-D-glucoside, in black rice [29,30]; 2) tricin-O-glucoside in rice hulls, bran, leaf and stem [28,31]; and 3) hydroxycinnamate sucrose esters, such as 6'-O-feruloylsucrose and 6'-O-sinapoylsucrose in germinated brown rice [32]. Hormone glucosides found in rice include gibberellin glucosides in ungerminated seeds and anther [23,33], salicylic glucoside [34] and indole-3-acetic acid (IAA)-glucoside [35]. Pyridoxine-β-D-glucoside was found in rice bran, callus and seedling [36-38]. Another glycoside, namely R(-) pantoyllactone-β-D-glucoside, was found in the shoots but not the roots of rice seedlings [39].

Many compounds (including glycosides) have been found in rice tissues in response to environmental stresses and in transgenic rice plants. Recently, it was found that there is a high accumulation of IAA-glucoside in tryptophan-overproducing transgenic rice [35] and of salicylic glucoside in rice overproducing NH1, a key regulator of salicylic acid mediated systematic acquired resistance, in transgenic rice [34]. The level of pyridoxine glucoside was reported to be increased by the application of pyridoxine to rice callus and germinating seeds [37,38]. Markham *et al*. [40] reported that exposing UV-tolerant rice to high UV-B levels increased the levels of flavone glucosides. These results may indicate that the presence of high amounts of some metabolic compounds is corrected by converting them to the glucoside-conjugated forms. It still needs to be shown whether or not these compounds are later reactivated by β-glucosidases.

### Protein sequence alignment and phylogenetic analysis

**Figure 1:**
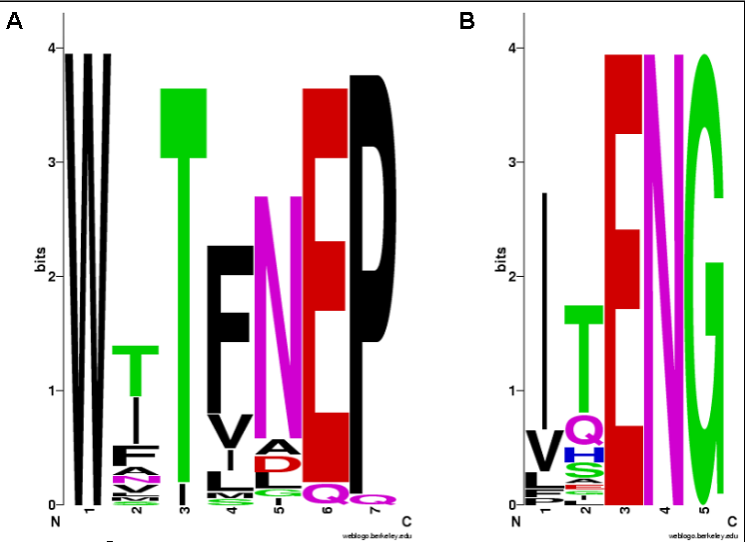
Sequence Logos for the residues surrounding the catalytic acid/base (A) and catalytic nucleophile (B) in rice GH1 genes. The logos show the size of the different amino acids at each position in proportion to their relative abundance within the 40 rice Glycosyl Hydrolase 1 gene protein sequences. The logos were drawn with the weblogo facility [73].

The open reading frames (ORFs) of thirty-seven gene-derived cDNAs (excluding *Os11bglu36*, *Osbglu39 *and *Osbglu40*, which are more closely related to bacterial GH1 genes) showed a high level of shared deduced amino acid sequence identity to each other and other known plant β-glucosidase sequences. All deduced β-glucosidase protein sequences contain the putative catalytic acid/base and nucleophilic glutamate residues, except Os4bglu14 and Os9bglu33, in which the acid/base glutamate is replaced with glutamine, as seen in thioglucosidases. The catalytic acid/base and nucleophile consensus sequences are: W-X-T/I-F/L/I/V/S/M-N/A/L/I/D/G-E/Q-P/I/Q and V/I/L-X-E-N-G, respectively, with relative frequencies of amino acids at each position shown in Figure 1. These sequences are similar to the consensus sequences previously derived from known GH1 β-glucosidase sequences [41,42]. The presence of the appropriate active site glutamic acids in the consensus sequences motifs suggests that all the genes identified in the rice genome database, except *Os4bglu14 *and *Os9bglu33*, at least have the potential to produce catalytically active β-glucosidases. β-glucosidases with Q instead of E at the acid/base position have been shown to be effective transferases in the presence of a good leaving group aglycone and a nucleophilic acceptor [43], therefore even Os4bglu14 and Os9bglu33 might be active if such glucosyl transfer reactions are catalyzed *in vivo*. Additionally, as seen in multiple sequence alignment (Additional Files 1, 2, 3), the amino acids identified by Czjzek *et al*. [41] as critical for glucose binding (Q38, H142, E191, E406, E464 and W465 in maize Bglu1) are generally well conserved in these predicted sequences. Only the predicted Os1bglu5 has Q instead of H142 in maize, whereas maize W465 is replaced by F in Os8bglu28, Os9bglu32 and Os9bglu33, Y in Os1bglu5 and Os9bglu31, L in Os1bglu2, Os1bglu3, Os5bglu21, Os5bglu22 and Os5bglu23, M in Os5bglu19, I in Os5bglu20 and S in Osbglu39. The residues that line the active site cleft and interact with the substrate aglycone of maize [41] are indeed quite variable in the predicted rice β-glucosidases, as would be expected for β-glucosidases with different substrate specificities.

**Figure 2:**
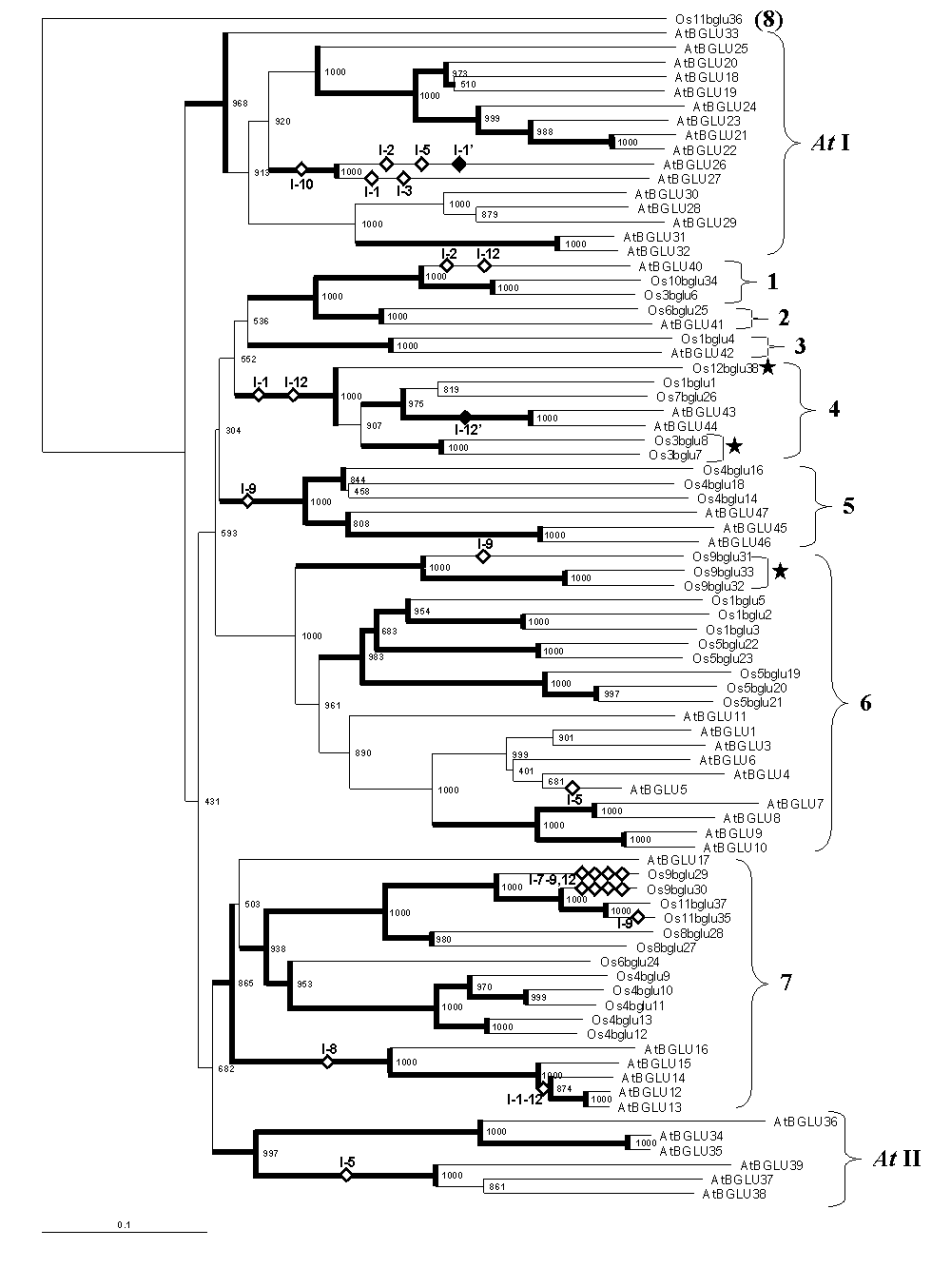
Phylogenetic tree of predicted protein sequences of rice and *Arabidopsis *Glycosyl Hydrolase Family 1 genes. The tree was derived by the Neighbor-joining method from the protein sequence alignment in the Supplementary Data Additional File 2 made with Clustalx with default settings, followed by manual adjustment. Large gap regions were removed for the tree calculation. The tree is drawn as an unrooted tree, but is rooted by the outgroup, Os11bglu36, for the other sequences. The bootstrap values are shown at the nodes. The clusters supported by a maximum parsimony analysis are shown as bold lines, and the loss and gain of introns are shown as open and closed diamonds, respectively. The 7 clusters that contain both *Arabidopsis *and rice sequences that are clearly more closely related to each other than to other *Arabidopsis *or rice sequences outside the cluster are numbered 1–7, while the outgroup cluster for which the *Arabidopsis *orthologue is not shown in numbered (8). Two *Arabidopsis *clusters that are more distantly diverged from the clusters containing both rice and *Arabidopsis *are numbered *At *I and *At *II, while rice genes and groups of genes that appear to have diverged before subclusters containing both rice and *Arabidopsis *are marked with stars.

Amino acid sequence alignment and phylogenetic analysis of 36 members including 34 full-length genes and 2 pseudogenes, but not including the intronless bacteria-like enzyme genes *Osbglu39 *and *Osbglu40*, and gene fragments, *Os4bglu15 *and *Os4bglu17*, showed that the sequences share a common evolutionary origin (Figure 2). Interestingly, many members that contain closely related sequences and cluster together are located on the same chromosome, such as the members in chromosomes 1, 4, 5, 8, 9 and 11, indicating localized (intrachromosomal) duplication events. Some of the closely related GH1 members of *Arabidopsis *also cluster on the same chromosome [22]. Comparison between rice and *Arabidopsis *GH1 members revealed that 7 clearly distinct clusters of plant-like GH1 genes (marked 1 to 7 in Figure 2) contain both *Arabidopsis *and rice genes that are clearly more closely related to each other than to other GH1 genes within their own species. In addition, the *Arabidopsis SFR2 *gene (not shown) forms another interspecies cluster with its rice homologue, *Os11bglu36*, which is marked (8) in Figure 2. Thus, it appears the ancestor of rice and *Arabidopsis *had at least 8 GH1 genes. However, 22 out of 40 *Arabidopsis *genes group in two large clusters without rice gene members (marked *At*I and *At*II in Figure 2), which incorporate several of the subfamilies defined by Xu *et al*. [22], and appear to have diverged before the rice and *Arabidopsis*. These include the myrosinases, which are not known to occur in rice, but also many apparent β-glucosidases. Similarly, some rice genes appear to have diverged from their cluster of *Arabidopsis *and rice genes before the other *Arabidopsis *and rice genes diverged. These include the *Os3bglu7 and Os3bglu8 *genes, which diverged from the lineage containing the *Arabidopsis *β-mannosidase genes before those genes diverged from *Os1bglu1 *and *Os7bglu26*. This suggests that the closest homologue of *Os3bglu7 *and *Os3bglu8*, which represent the most highly expressed GH1 genes in rice based on EST analysis, was lost from *Arabidopsis*. Thus, genes found in the common ancestor, including two that were duplicated into most of the *Arabidopsis *GH1 repertoire, appear to have been lost in the other plant's lineage. However, it is possible that rapid evolution of these genes caused them to be misplaced by the phylogenetic analysis, so care must be taken in interpreting these analyses. This analysis suggests that the common ancestor of monocots and dicots had at least 11–13 GH1 genes, 8 of which are represented by common lineages in modern rice and *Arabidopsis*.

Taken together, the great divergence of rice and *Arabidopsis *genes after the divergence of the species and the loss of important lineages from either rice or *Arabidopsis *suggest that much of the functional divergence of GH1 may have occurred after the monocot-dicot divergence. Therefore, it may be difficult to extrapolate functions found in *Arabidopsis *to those in rice and vice-versa, except in a few cases (such as *AtBGLU41 *and *Os6bglu25*, which have not duplicated since the divergence of the species).

**Figure 3:**
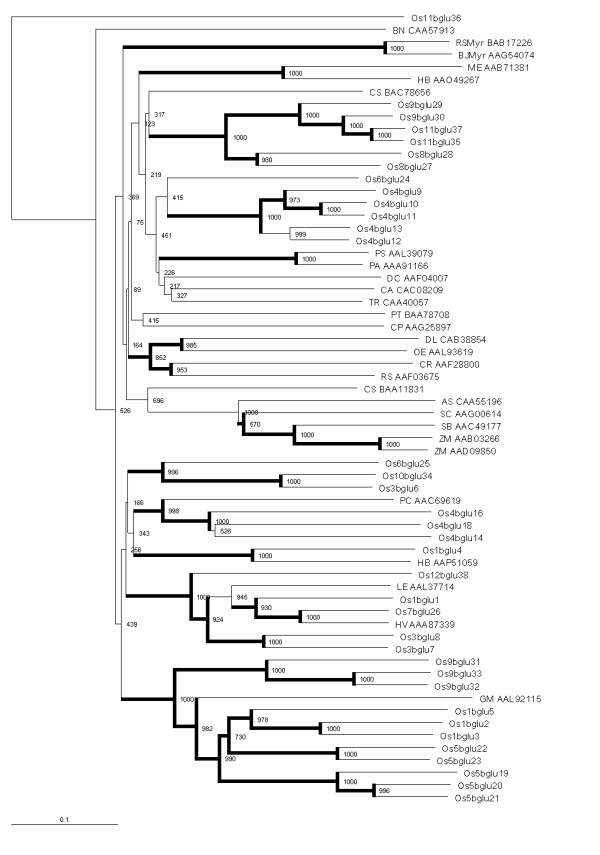
Relationship between rice and other plant GH1 protein sequences described by a phylogenetic tree rooted by Os11bglu36. The sequences were aligned with ClustalX, then manually adjusted, followed by removal of N-terminal, C-terminal and large gap regions to build the data model. The tree was produced by the neighbor joining method and analyzed with 1000 bootstrap replicates. The internal branches supported by a maximum parsimony tree made from the same sequences are shown as bold lines. The sequences other than rice include: ME AAB71381, *Manihot esculenta *linamarase; RSMyr BAB17226, *Raphanus sativus *myrosinase; BJMyr AAG54074, *Brassica juncea *myrosinase; BN CAA57913, *Brassica napus *zeatin-O-glucoside-degrading β-glucosidase; HB AAO49267, *Hevea brasiliensis *rubber tree β-glucosidase; CS BAA11831, *Costus speciosus *furostanol glycoside 26-O-β-glucosidase (F26G); PS AAL39079*Prunus serotina *prunasin hydrolase isoform PH B precursor; PA AAA91166, *Prunus avium *ripening fruit β-glucosidase; TR CAA40057, *Trifolium repens *white clover linamarase; CA CAC08209, *Cicer arietinum *epicotyl β-glucosidase with expression modified by osmotic stress; DC AAF04007, *Dalbergia cochinchinensis *dalcochinin 8'-O-β-glucoside β-glucosidase; PT BAA78708, *Polygonum tinctorium *β-glucosidase; DL CAB38854, *Digitalis lanata *cardenolide 16-O-glucohydrolase; OE AAL93619, *Olea europaea *subsp. europaea β-glucosidase; CR AAF28800, *Catharanthus roseus *strictosidine β-glucosidase; RS AAF03675, *Rauvolfia serpentina *raucaffricine-O-β-D-glucosidase; CP AAG25897, *Cucurbita pepo *silverleaf whitefly-induced protein 3; AS CAA55196, *Avena sativa *β-glucosidase; SC AAG00614, *Secale cereale *β-glucosidase; ZM AAB03266, *Zea mays *cytokinin β-glucosidase; ZM AAD09850, *Zea mays *β-glucosidase; SB AAC49177, *Sorghum bicolor *dhurrinase; LE AAL37714, *Lycopersicon esculentum *β-mannosidase; HV AAA87339, barley BGQ60 β-glucosidase; HB AAP51059, *Hevea brasiliensis *latex cyanogenic β-glucosidase; PC AAC69619*Pinus contorta *coniferin β-glucosidase; GM AAL92115, *Glycine max *hydroxyisourate hydrolase; CS BAC78656, *Camellia sinensis *β-primeverosidase.

Phylogenetic analysis of rice GH1 members with other plant enzymes also led to several interesting observations (Figure 3). Some rice and *Arabidopsis *members that are clustered in the same groups were found to be closely related to β-glucosidases from other plants. For example, Os4bglu14, Os4bglu16 and Os4bglu18, which cluster with *Arabidopsis *BGLU45, 46 and 47, are grouped with *Pinus contorta *coniferin/syringin β-glucosidase (PC AAC69619) [6], suggesting that they may be involved in lignification. In fact, recombinantly expressed *Arabidopsis *BGLU45 and BGLU46 have recently been shown to hydrolyze lignin precursors [44]. Although *Arabidopsis *BGLU11 and rice enzymes (Os1bglu2, Os1bglu3, Os1bglu5, and Os5bglu19 through Os5bglu23) have sequences closely related to *Glycine max *hydroxyisourate hydrolase (GM AAL92115) [11] and cluster into the same large group, they do not have HENG catalytic nucleophile motif found in hydroxyisourate hydrolase, whereas the somewhat more distantly related Os9bglu31, Os9bglu32, and Os9bglu33 do. However, the rice enzymes generally still contain the conserved glucose binding residues lost from the *G. max *hydroxyisourate hydrolase, so they may still act as glycosyl hydrolases, rather than as other kinds of hydrolases.

Os1bglu1, Os3bglu7, Os3bglu8, Os7bglu26 and Os12bglu38 β-glucosidases clearly grouped with barley BGQ60 β-glucosidase/β-mannosidase [15,45]. Kinetic analysis showed that the hydrolytic activity of Os3bglu7 (rice BGlu1 in Opassiri *et al*. [24]) toward β-linked glucose oligosaccharides is similar to that of the barley enzyme [17]. Barley BGQ60 also shares high sequence identity and similar gene organization with *Arabidopsis *BGLU44 and tomato β-mannosidase. Recombinant AtBGLU44 protein shows a preference for β-mannoside and β-mannan oligosaccharides [22], as does barley BGQ60 [46,47], while Os3bglu7 prefers glucoside 10-fold over mannoside [17]. Thus, within this cluster of closely related genes, both exo-β-glucanase and β-mannosidase (exo-β-mannanase) activities are found.

Several GH1 enzymes associated with defense do not have clear orthologues in either rice or *Arabidopsis *(Figure 3 and [22]). No rice GH1 members cluster with the monocot chloroplast targeted enzymes, such as maize Bglu1 and sorghum dhurrinase, while the 2 groups cluster loosely with the dicot defense enzymes, such as white clover and cassava linamarinases. The chromosome 4 cluster of Os4bglu9-12 and Os6bglu24 form one group embedded within the dicot defense enzymes, while Os8bglu27, Os8bglu28, Os9bglu29, Os9bglu30, Os11bglu35, and Os11bglu37 form another cluster within this group. The association of these genes with the defense enzymes was seen in both distance-based and sequence-based phylogenetic analysis, but they were not strongly supported by bootstrap analysis in either case. As noted by Henrissat and Davies [48], it is not generally possible to assign glycosyl hydrolase function based on sequence similarity scores alone, and the high divergence between the rice and defense-related β-glucosidases makes it unclear which, if any, play a role in defense.

There is only low sequence similarity between *Os11bglu36 *and the other rice GH1 members, suggesting that it diverged from the other plant enzyme genes before plants evolved. *Os11bglu36 *is most similar to the *Arabidopsis SFR2 *β-glucosidase-like gene, AC: AJ491323 [49]. The *SFR2 *gene is also found in other plant species, such as maize, wheat, *Glycine max, Lycopersicon esculentum, Pinus taeda*, sorghum, and barley.

### Gene organization

**Figure 4:**
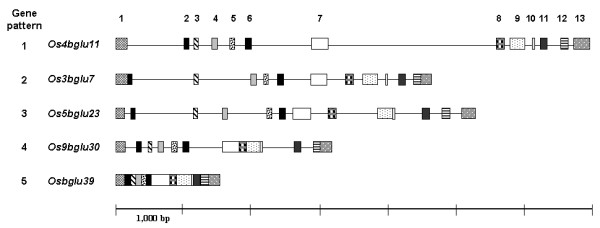
Predicted gene structure patterns for putative rice GH1 β-glucosidase genes. Exons are shown as boxes with corresponding exons having the same pattern. Introns, represented as simple lines, are drawn in proportion to their length. Note that 5 gene organization patterns can be seen in rice genes, those with 13, 12, 11, or 9 exons and intronless patterns, with the splice sites conserved in each group and between groups for common exons and introns.

Gene structural analysis of the β-glucosidases showed intron-exon boundaries and intron numbers are highly conserved among rice and other plant β-glucosidase genes. Intron sizes in these genes, however, are highly variable. In most cases, very long introns contained retro-transposon-like sequences, while the orthologous short introns did not. Five patterns of gene structures are distinguished by the number of exons and introns, which are 13, 12, 11, or 9 exons, and intronless (Figure 4). However in each case, existent introns maintained the same splice sites. It was found that *Arabidopsis *also has several GH1 gene organization patterns, though some are different from rice [22]. *Arabidopsis *GH1 genes exhibit 10 distinct exon-intron organization patterns and 3 members exhibit a new intron that is not found in rice and is inserted into exon 13 to yield two novel exons. Only gene structure patterns 1, 3 and 5 of rice GH1 are found in *Arabidopsis*. Similar to *Arabidopsis*, the most common gene pattern, found in 22 rice genes, is pattern 1, in which there are 13 exons separated by 12 introns (Table 1). The results from deduced amino acid sequence alignment and phylogenetic analysis (Figure 2) showed that the sequences in intron-exon pattern groups 2, 3, 4 and 5 are usually more closely related to each other within their groups than to the other groups.

The genes with 13 exons (group 1) are more divergent, indicating this pattern is probably the ancestral gene organization. Those genes with 11 exons clustered together in one group with barley BGQ60, while those with 9 and 12 exons clustered in separate groups. This phylogeny is consistent with an ancestral plant β-glucosidase having 13 exons and 12 introns, with losses of introns in groups 2, 3 and 4. To generate this phylogeny by gain of introns would require intron insertion at the exact same splice site position multiple times to generate the divergent genes with the 13 exon pattern. For a similar reason, though the sequence analysis shown in Figure 2 suggests *Os9bglu29 *diverged from *Os9bglu30 *before it diverged from the ancestor gene of *Os11bglu35 *and *Os11bglu37*, the loss of the same introns (6, 7, 8 and 9) in *Os9bglu29 *and *Os9bglu30*, suggests they are more recently diverged. Since *Os11bglu35 *also lacks intron 9, it may have diverged more recently than *Os11bglu37 *as well, though it is possible this was an independent intron loss. Thus, it appears that rapid accumulation of changes in *Os9bglu29 *and *Os9bglu30 *caused their sequences to differ more than would be expected from the recent divergence indicated by their shared gene structures.

The two intronless genes found in the BGI database may be contamination left from endophytes which has not been removed from the *indica *database, since originally there were 5 other intronless GH1 genes that were in this database. Support for this hypothesis is provided by their sequences, since Osbglu39 shows 58% identity with *Lactobacillis *β-glucosidase, and Osbglu40 has 70% identity with bacterial proteins, while they only share 28–30% identity with the other rice proteins. Alternatively, they may have been gene transcripts that were captured by retrotransposons and reincorporated into the rice genome, or may have been obtained by lateral gene transfer from a bacteria. The intron-exon boundaries of the *Os11bglu36 *gene do not correspond to those of other rice β-glucosidase genes, indicating it is from a separate lineage, though also of plant origin.

### Expression of rice β-glucosidase genes

In order to begin to analyze the tissue specific expression of the β-glucosidase genes in rice, a search for ESTs corresponding to each of the 40 different predicted genes was performed in dbEST and the full-length cDNA clones of *japonica *rice databases [50]. As shown in Table 1, an initial homology search with β-glucosidase sequences identified 823 ESTs and 55 "full" cDNAs, which are derived from 31 GH1 genes. The *Os3bglu7 *is most highly represented in the dbEST database, with 326 ESTs. *Os3bglu8 *has the second highest abundance of ESTs with 77 ESTs. Other GH1 genes with a relatively large numbers of ESTs are *Os4bglu12*, *Os5bglu22, Os7bglu 26, Os9bglu30, Os9bglu31, and Os9bglu32 *(Table 1). However, the high abundance of ESTs for some rice genes might not reflect the relative expression levels in particular tissues, because of bias in selecting plant parts and developmental stages for production of EST cDNA libraries [22]. It should be noted that *Os4bglu14 *and *Os9bglu33*, which lack the catalytic acid/base, both have transcripts in the database, which indicates that they are transcriptionally active although the protein product may not have hydrolase activity. Several genes are not represented in the EST/full-length cDNA databases (i.e., the full-length genes: *Os5bglu21*, *Os5bglu23*, and *Os6bglu24*; pseudogenes: *Os11bglu35 *and *Os11bglu37*, gene fragments: *Os4bglu15 *and *Os4bglu17*; and intronless genes: *Osbglu39 *and *Osbglu40*). So, whether and where the full-length gene members are expressed remains unclear. It is possible that the expression levels of these genes are very low, or their expression may be induced by particular environmental conditions.

The source libraries for rice GH1 gene ESTs include callus, seedling (shoot and root), immature plant parts (stem, root, leaf), mature plant (leaf), panicle at flowering and ripening stage, and immature seeds. Some rice β-glucosidase genes have ESTs from stressed plant tissue libraries, such as salt (i.e. *Os3bglu7*, *Os4bglu12*), drought (i.e. *Os1bglu2*, *Os1bglu4*, *Os4bglu12*), cold (i.e. *Os3bglu6*, *Os3bglu7*), heat (i.e. *Os3bglu7*) and fungus infection (i.e. *Os1bglu2*, *Os1bglu3*, *Os1bglu4*) (see Table 1). In addition, some genes (i. e.* Os1bglu1*,* Os1bglu3*,* Os3bglu7*,* Os3bglu8*) are also expressed in transgenic rice, such as in the leaf of rice overexpressing ABA-responsive element binding transcription factor 3 (TF3). These EST/cDNA sequences were used to identify the 3'UTR sequence for each gene and it was found that all cDNAs contain unique 3'UTR sequences, which may therefore be used as unique probes for each gene. The occurrence of the ESTs/cDNAs of β-glucosidase genes in tissues may correlate with growth and development. As mentioned by Xu *et al*. [22], the members of a given subfamily may have the same biochemical function and may be expressed in different cells, tissues, or organs and may be expressed in response to different environmental conditions and stresses. However, the multiple forms of rice β-glucosidases may also represent functional redundancy and be expressed in the same tissues.

One question of interest was why the chloroplast β-glucosidases seemed most predominant in maize, oat, sorghum and wheat, while such genes have lower expression in rice. A comparison of ESTs from several grain species showed that the chloroplast β-glucosidases of other cereals have the most EST hits of GH1 genes, while in rice and barley, the rice BGlu1/barley BGQ60-like genes were more predominant (Additional File 4). However, since the genome and transcriptome analysis of these grasses is not completed, some bias may have been introduced in the selection of the tissues studied. Given the large number of ESTs in maize and rice, it seems likely to be a reasonable comparison, despite these limitations. If so, it may be that the defense function of the chloroplast isozymes in maize and other grasses, has been replaced by other defenses or by the abundance of Os3bglu7, which might be found in a separate compartment from defensive substrates, as well. Though Os3bglu7 is thought to function in hydrolysis of oligosaccharides released from the cell wall [24], it might be possible for it to fulfill more than one role. Recently, barley β-glucosidase, which is thought to help in hydrolysis of cell wall oligosaccharides during germination, has been found to hydrolyze cyanogenic glycosides from barley leaves [51], giving support to the possibility of one enzyme playing roles in both the cell wall and defense.

A few reports described the expression patterns of β-glucosidases in rice plants. Based on enzyme activity, gibberellic acid glucoside and pyridoxine glucoside β-glucosidases are found in rice bran [23,52], and the cell wall-bound enzyme is found in seedlings [16]. Northern blot analysis showed that *Os3bglu7 *and *Os9bglu30 *(rice *bglu 2 *in Opassiri *et al*. [24])*β*-glucosidase genes are highly expressed in seedling shoots, but only *Os3bglu7 *is expressed in flowers [24]. Microarray analysis indicated that the transcripts of the ESTs BE607353 and BG101702, whose sequences are homologous to *Os3bglu7 *and *Os4bglu12 *β-glucosidases genes, respectively, are upregulated in response to high salinity stress in salt-tolerant rice (var Pokkali), but not in the salt-sensitive cultivar IR29 [53]. Subtractive hybridization cDNA library screening indicated that the transcript level of the EST contig BPHiw028, homologous to *Os4bglu12*, is upregulated in response to brown planthopper [54]. The presence of tricin-O-glucoside, a probing stimulant for planthopper [31], suggests that the role of this enzyme is to release an active flavonol for defense. However, these studies did not show the specific roles of these enzymes in rice cells in response to such stresses. Therefore, identification of natural substrates for the enzymes is needed to understand the functions of these enzymes.

### Properties of predicted proteins

The deduced precursor proteins were analyzed for potential signal sequences using SignalP, and cellular location by PSORT. Almost all β-glucosidase ORFs, except Os1bglu4 and Osbglu39, were predicted to have signal peptides ranging in length from 18 to 44 amino acids, which would target them to the secretory pathway (Table 2). Three *Arabidopsis *GH1 members, AtBGLU26, 27, and 42 were predicted to not have signal peptides [22]. In *Arabidopsis*, putative signal peptides were predicted to range in length from 19–38 aa. The predicted cellular locations for rice GH1 proteins included the cell exterior, cytoplasm, peroxisome, vacuole, ER lumen, ER membrane, plasma membrane, and mitochondrial matrix, which are similar to *Arabidopsis *proteins. Though assignment of cellular location was generally unclear using the PSORT program, Os1bglu2 and Os11bglu36 (*Arabidopsis SFR2 *homolog) are predicted to localize to the chloroplast, like maize, sorghum, wheat and oat β-glucosidases, though they are not closely related phylogenetically. However, none of the *Arabidopsis *β-glucosidases seemed to be targeted to plastids, except possibly *SFR*2 (which is closely related to Os11bglu36 and gave a weak prediction of this localization). The deduced proteins were also analyzed for predicted molecular mass, pI, and potential N-linked glycosylation sites (Table 2). Predicted precursor protein lengths vary from 458–647 amino acids, which correspond to protein molecular weights of 55.3 to 73.2 kD. Mature polypeptide lengths vary from 474–592 amino acids, corresponding to MW 53.8–70.8 kD. All but Os1bglu4 contain one to six N-linked glycosylation sites. Isoelectric points (pI) of predicted proteins are divided into 3 groups, acidic (4.96–6.66), neutral (6.99–7.78), and basic (8.36–8.96), and 21 of 35 of these proteins are in the acidic group. Predicted protein properties of rice GH1 members are similar to *Arabidopsis *GH1 proteins, which have predicted MW of precursor proteins and mature proteins in the range of 56–70.1 and 53–68 kD, respectively, and contain one to five N-glycosylation sites [22]. Similar to Os1bglu4, AtBGLU25 and 27 do not contain N glycosylation sites. The number of likely isozymes complicates the interpretation of results from traditional biochemical approaches, such as measuring enzyme activities in tissue extracts. Protein purification may also be difficult due to the similar sizes and pI of several predicted isozymes, as seen in Table 2.

Although the occurrence of a number of glycosides in rice is known, few rice β-glucosidases have been studied and none of them has been tested for activity on most of the known natural glycosides. The first report of rice β-glucosidase activity against the synthetic substrate *p*NP-β-D-glucoside (*p*NPG) was by Palmiano and Juliano [55]. Partially purified β-glucosidases from rice have been described that hydrolyze gibberellin glucosides and pyridoxine glucosides [23,52]. Analysis of thoroughly purified rice β-glucosidases has been described for a β-glucosidase from a cell wall-bound fraction (possibly Os4bglu12) and Os3bglu7 cloned from rice seedlings [16,17,24]. Both enzymes showed high hydrolytic activity against cello- and laminari-oligosaccharides. In order to better characterize the function of the GH1 multi-enzyme family in rice, recombinant expression of these genes or their cDNAs to produce the enzymes is necessary. The recombinant production and characterization of Os4bglu12 is presented below as a first step in establishing the biochemical function of the rice GH1 enzymes.

### Os4bglu12 β-glucosidase cDNAs cloning and sequence analysis

The protein product for *Os4bglu12 *gene has highest sequence similarity to the previously described cell wall-bound β-glucosidase purified from rice seedlings [16]. Therefore, it was chosen for expression to test if the protein would have the expected activity. The sequence of the *Os4bglu12 β*-glucosidase mRNA from rice was confirmed by RT-PCR cloning and sequencing, using rice cultivar KDML105 cDNA as the template. A specific PCR product of 1635 bp was produced, and its sequence overlapped that of the *indica *rice contig AAAA02014151.

The reconstructed cDNA sequence of *Os4bglu12 *included a 1530-nucleotide long open reading frame encoding a 510 amino acid long precursor protein. The Signal P program predicted the protein to contain a 24 amino acid signal sequence and a 486 amino acid mature protein (Table 2). The deduced Os4bglu12 N-terminal amino acid sequence was identical to the N-terminal amino acid sequence of the previously purified cell-wall-bound rice β-glucosidase at 40 of 44 residues [16].

### Functional expression of recombinant Os4bglu12

**Figure 5:**
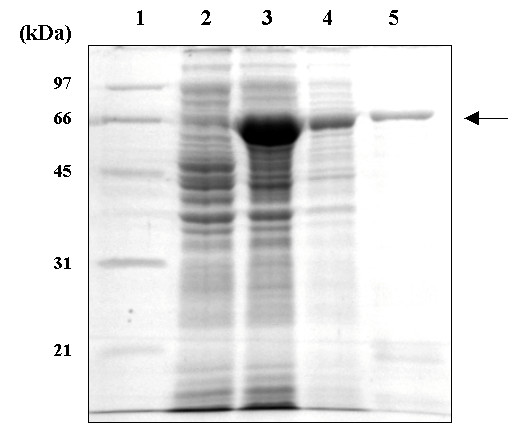
SDS-PAGE profiles of Os4bglu12 recombinant protein expressed in *OrigamiB *(DE3)*E. coli *after incubation in the presence of 0.3 mM IPTG, at 20°C for 8 h. Lanes: 1, standard marker (Bio-RAD); 2, total protein in *E. coli *cells containing pET32a(+) without an insert; 3, total protein of *E. coli *cells containing pET32a(+)/DEST-*Os4bglu12*; 4, soluble fraction of *E. coli *cells containing pET32a(+)/DEST-*Os4bglu12*; 5, purified Os4bglu12 recombinant protein. The arrow points to the position of thioredoxin fusion protein monomer.

The *Os4bglu12 *cDNA CDS including the stop codon was inserted into pET32a(+)/DEST. The construct was used to transform *OrigamiB *(DE3) *E. coli*. Comparison of the protein profile of induced cultures with the *Os4bglu12 *insert with those of empty plasmid controls by SDS-PAGE showed the thioredoxin-Os4bglu12 fusion protein as an intense band at 69 kDa on SDS-PAGE. The fusion protein was purified by IMAC, and a band corresponding to 69 kDa was observed in SDS-PAGE (Figure 5). The enzyme was found to hydrolyze *p*NPG with optimal activity at pH 5.0 and 37°C. The enzyme activity with *p*NPG at 70°C and 80°C drops about 17% and 39%, respectively, from the optimal activity at 37°C in a 10 min assay. It was stable at 4°C for several months.

### Os4bglu12 substrate specificity

**Figure 6:**
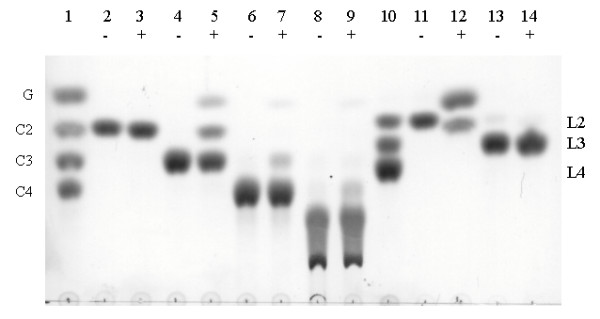
Hydrolysis of oligosaccharide substrates by Os4bglu12 detected by TLC. The Os4bglu12 was incubated with 5 mM substrates for 30 min and the products were detected after TLC by the carbohydrate staining. Samples were incubated with (+) or without (-) enzyme. Lanes: 1, glucose (G) and cello-oligosaccharides of DP 2–4 (C2-C4) marker; 2 and 3, cellobiose; 4 and 5, cellotriose; 6 and 7, cellotetraose, 8 and 9, cellopentaose, 10 laminari-oligosaccharides of DP 2–4 (L2-L4) marker; 11 and 12, laminaribiose; 13–14 laminaritriose.

The activity of the purified rice Os4bglu12 β-glucosidase towards natural and artificial glycosides is summarized in Table 3. The Os4bglu12 hydrolyzed the β-1,3-linked glucose disaccharide laminaribiose, but not cellobiose (β-1,4) or gentiobiose (β-1,6). It showed high hydrolytic efficiency at different rates with β-(1,4)-linked oligosaccharides with degree of polymerization (DP) of 3–6. Hydrolysis of β-(1,3)-linked oligosaccharides with DP > 2, laminarin and barley 1,3, 1,4-β-glucans by this enzyme could not be detected. The rate of hydrolysis of oligomeric substrates tended to remain approximately constant with increasing DP, which is a characteristic often observed with β-glucosidases [56]. On the TLC, Os4bglu12 showed hydrolytic activity towards 5 mM laminaribiose and cello-oligosaccharides, but no measurable transglycosylation activity (Figure 6).

Hydrolysis of *p*NP-glycosides with different glycone moieties was used to assess glycone specificity of Os4bglu12. It hydrolyzed *p*NPG and *p*NP-β-D-fucoside with 2–3 fold lower efficiency than oligosaccharides. It also hydrolyzed *p*NP-β-D-galactoside, *p*NP-β-D-xyloside, and *p*NP-α-L-arabinoside, at 45%, 45% and 26% the rate of *p*NPG, respectively. Hydrolysis of *p*NP-β-D-mannoside, *p*NP-β-D-cellobioside, *p*NP-α-D-glucoside, and *p*NP-β-L-fucoside was not detectable. High hydrolysis of β-xyloside is similar to white clover β-glucosidase, but otherwise rare in GH1 enzymes that have been characterized to date [57].

Rice Os4bglu12, Os3bglu7 [24], and cell wall-bound β-glucosidases [16] and barley β II β-glucosidase [45] are enzymes that hydrolyze β-linked glucose oligosaccharides, but not polysaccharides. However, the specificity for glycones and substrate chain lengths of these enzymes are different. In contrast to barley and rice cell wall-bound enzyme, Os4bglu12 did not hydrolyze β-(1,3)-linked oligosaccharides longer than laminaribiose, but hydrolyzed various *p*NP-derivatives of monosaccharides. This substrate preference was not expected, since it was initially expected that *Os4bglu12 *was the gene for the cell wall-bound β-glucosidase, and the sequence differences might be due to cultivar differences or sequencing errors. The substrate preference of Os4bglu12 is somewhat similar to Os3bglu7, in that they both show slightly faster hydrolysis of *p*NP-β-D-fucoside than *p*NPG and hydrolyze laminaribiose and cello-oligosaccharides. However, there were many differences between these enzymes. For example, in contrast to rice Os3bglu7, Os4bglu12 hydrolyzed β-(1,4)-linked oligosaccharides and laminaribiose at higher rates than *p*NPG, and did not hydrolyze cellobiose, gentiobiose, *p*NP-β-D-mannoside, and *p*NP-β-D-cellobioside. Their sequence differences are likely to reflect the differences in substrate binding to the active site between these enzymes. The amino acids identified by Czjzek *et al*. [41] as critical for glucose binding (Q38, H142, E191, E406, E464 and W465 in maize Bglu1) are conserved in rice Os4bglu12, Os3bglu7, and barley β-glucosidase. Interestingly, the Os3bglu7 protein sequence was closest to barley BGQ60 at some of substrate binding residues that line the active site cleft and interact with the substrate aglycone of maize Bglu1 (W378, F198, F205, and F466) [41], suggesting Os3bglu7 and BGQ60 may have a similar substrate-specificity. However, these above mentioned amino acid residues were different from those in the Os4bglu12 enzyme, which may account for the different substrate specificities for some oligosaccharides and glycones. For instance, Os3bglu7 and barley BGQ60 cluster with tomato and *Arabidopsis *β-mannosidase and can hydrolyze β-mannoside, while Os4bglu12 does not, and they also hydrolyze longer chain 1,3-linked oligosaccharides [17,46]. All three enzymes prefer shorter 1,3-linked oligosaccharides, with Os4bglu12 being the most extreme, only hydrolyzing the dimer with this linkage. This likely reflects the bent shape of oligosaccharides with the 1,3-linkage, which is somehow incompatible with the active site for longer chains. Elucidation of the tertiary structures of these enzymes would help to clarify the enzyme-substrate binding mechanism leading to these preferences.

## Conclusion

In summary, forty genes encoding GH1 β-glucosidases have been identified from the rice genome databases. Gene-derived cDNAs were predicted and compared to experimentally derived cDNA in the database. Intron-exon boundaries and intron numbers are highly conserved among rice and other plant β-glucosidase genes. At least 31 rice β-glucosidase genes have corresponding ESTs, indicating their transcription, and these ESTs come from many tissues, indicating their temporal and spatial regulation and importance for the rice plant. Most of these genes appear to have diverged from each other after the divergence of rice and *Arabidopsis *from their common ancestor, implying that their functions may not be easily defined by studies in *Arabidopsis *and other dicots. To begin a functional analysis of rice GH1 enzymes, the *Os4bglu12 *cDNA encoding the protein with the amino acid sequence that was most similar to the previously purified and characterized cell wall-bound β-glucosidase was cloned by RT-PCR and expressed in *E. coli*. Recombinant Os4bglu12 protein hydrolyzed β-linked oligosaccharides and *p*NP-glycosides. The specificity of Os4bglu12 for oligosaccharides and *p*NP-glycosides was different from the previously characterized GH1 β-glucosidases/exoglucanases, cell wall-bound rice β-glucosidase, Os3bglu7, and barley β II β-glucosidase. This work represents a start toward determining the roles of the GH1 β-glucosidases in rice, which provides an opportunity to investigate the molecular basis for differences in substrate specificity and the evolution of enzyme functions.

## Methods

### Plant materials and growth conditions

Rice (*Oryza sativa *L. spp. *indica *cv. KDML105) seeds were germinated in the dark from day 0 to day 3 and in 12 h light-12 h dark from day 4 to day 6 at 28°C on germinating paper moistened with sterile distilled water. The whole seedlings were harvested and kept at -70°C.

### Database searching and sequence analysis

Identification of rice genes homologous to GH1 β-glucosidase genes was done using the BLAST suite of programs [58] in 4 databases: GenBank at NCBI [59], the Monsanto Rice Genome Draft Database [60], the Beijing Genomic Institute, BGI [26] and the Syngenta Torrey Mesa Research Institute database [61]. Because all genes could be found in the GenBank *japonica *and BGI *indica *sequences, the other databases were not included. Identification of homologous genes and cDNA was done using tBLASTn with known β-glucosidase protein sequences from GenBank: rice *bglu1 *(AC U28047) maize *bglu1 *(AC U33816), barley BGQ60 (AC L41869), and *Arabidopsis *psr3.2 (AC U72155), as queries, while BLASTn was used to identify sequences from the same gene. Coding regions of genes were identified by BLASTx searches against the NCBI nr protein database. Exact splice sites were predicted by identification of splice site consensus sequences near the ends of identified coding regions, which maintained the correct reading frame. When available, full-length cDNA and expressed sequence tag (EST) sequences were used to confirm the gene predictions. Translation of gene sequences was done using the 6-frame translation facility at the Baylor College of Medicine (BCM) search launcher site [62,63]. The ClustalX implementation of ClustalW was used for protein sequence alignments [64,65] and phylogenetic analyses done by the built in NJ-tree facility of this program with bootstrapping (1000 iterations), after manual adjustment of the alignment with the Genedoc program. Bootstrapped neighbor joining and maximum parsimony trees with and without gap sequences were also developed with the PHYLYP suite [66], and the results were compared to those generated with ClustalX. The rice *SFR2 *homologue, *Os11bglu36*, was used as the outgroup in these analyses, since it is derived from a distinct lineage within GH1.

The organization of the genes was diagramed and categorized from the conservation of introns and exons in rice β-glucosidase gene structures. The sequence and gene structure analyses were correlated to describe the evolutionary relationships among the genes. Each β-glucosidase gene sequence was searched against the GenBank at NCBI using BLASTn to identify the chromosomal locations. Cellular locations of predicted proteins were predicted by PSORT [67], signal sequences were predicted by SignalP [68], N-glycosylation sites were predicted by NetNGlyc, and the molecular weights (MW) and isoelectric points (pI) of the proteins were predicted by ProtParam at the Expasy proteomics server [69].

In order to determine the relative abundance of mRNAs of each GH1 gene in rice, a BLASTn search with the derived cDNA sequence for each predicted gene was performed in dbEST and the *japonica *rice full-length cDNA clones [50]. All EST/cDNA clone IDs were retrieved and collected in the catalog to compare gene expression in various library sources. In addition, rice-specific tBLASTn searches using known β-glucosidase protein sequences were performed in the dbEST to identify all ESTs/cDNAs encoding β-glucosidase proteins from rice, as described for gene identification. Final EST/cDNA collections for each gene were compared with the Unigene facility of the NCBI GenBank database.

### Cloning of rice Os4bglu12 β-glucosidase cDNA

Total RNA was isolated from 100 mg 5-6-d-old rice seedlings using Trizol Reagent (Invitrogen, Carlsbad, CA). The total RNA (5 μg) was used as the template to synthesize the first-strand cDNA with SuperScript II reverse transcriptase according to the manufacturer's protocol (Invitrogen). Primers for amplifying the full-length coding sequence (CDS) cDNA (designated *Os4bglu12*) and a cDNA encoding the mature protein of rice Os4bglu12 *β*-glucosidase were designed from the GenBank *indica *rice genome contig number AAAA02014151 and the AK100820 and AK105375 cDNA sequences [50]. A 5' sense primer, Os4bglu12_fullf (5'-TGTCCATGGCGGCAGCAG-3'), and the antisense primer, Os4bglu12_3'UTRr (5'-AACTGGATTACTTCCATCTC-3'), were used to amplify the full-length cDNA. The amplification was done with 30 cycles of 94°C, 30 s, 53°C 30 s and 72°C 4 min, and *Pfu *DNA polymerase (Promega, Madison, WI). A full-length product was cloned into the *Eco*R V site of pBlueScript II SK+ (Stratagene, La Jolla, CA), and sequenced.

### Protein expression in *Escherichia coli*

The cDNA encoding the mature protein of rice Os4bglu12 *β*-glucosidase was cloned by RT-PCR and inserted into pENTR-D/TOPO Gateway entry vector and transferred to the pET32a (+)/DEST Gateway expression vector for expression. The Gateway Conversion cassette A was ligated into the *EcoR*V site of pET32a (+) (Novagen, Madison, WI) according to the Invitrogen Gateway Conversion Kit directions, to create the pET32a (+)/DEST Gateway expression vector. The cDNA encoding the mature protein of the Os4bglu12 was PCR amplified using cDNA cloned as the template with the Os4bglu12matNcoIf (5'-CACCATGGCCTACAATAGCGCCGGCGAG-3') and Os4bglu12stopr (5'-ATCATTTCAGGAGGAACTTCTTG-3') primers and *Pfu *DNA polymerase to introduce a directional cloning site at the 5' end. The amplification was done as above, but with 45°C annealing temperature. The PCR product was cloned into the pENTR-D/TOPO Gateway entry vector, according to the supplier's directions (Invitrogen). The cDNA insert in the pENTR-D/TOPO vectors was subcloned into the pET32a (+)/DEST Gateway expression vector by LR Clonase recombination by the recommended protocol (Invitrogen) and sequenced completely. The recombinant pET32a (+)/DEST-*Os4bglu12 *plasmid was transformed into *OrigamiB *(DE3) *E. coli *by the CaCl_2 _method [70], and positive clones were selected on a 15 μg/mL kanamycin, 12.5 μg/mL tetracycline and 100 μg/mL ampicillin LB-agar plate.

For recombinant protein expression, the selected clones were grown in LB medium containing 15 μg/mL kanamycin, 12.5 μg/mL tetracycline and 100 μg/mL ampicillin at 37°C until the optical density at 600 nm reached 0.5–0.6, IPTG was added to a final concentration of 0.3 mM, and the cultures were incubated at 20°C for 8 h. Induced cultures were harvested by centrifugation at 5000 × g at 4°C for 10 min. The cell pellets were resuspended in freshly prepared extraction buffer (50 mM phosphate buffer (pH 8.0), 200 μg/mL lysozyme, 1% Triton-X 100, 1 mM phenylmethylsulfonylfluoride, 40 μg/mL DNase I), and incubated at room temperature for 30 min. The soluble protein was recovered by centrifugation at 12,000 × g at 4°C for 10 min. The expressed thioredoxin-Os4bglu12 fusion protein was purified by immobilized metal affinity chromatography (IMAC) with TALON cobalt resin according to the manufacturer's instructions (Clonetech, Palo Alto, CA). The fractions with *p*NPG hydrolysis activity were pooled and concentrated with 10 kDa-cut-off centrifugal ultrafiltration membranes (YM-10, Amicon). All of the protein samples were subjected to SDS-PAGE by the standard method [71].

### β-glucosidase assays

Substrate specificity of thioredoxin-Os4bglu12 fusion protein was tested against oligosaccharides and polysaccharides. For oligosaccharides, 0.05 μg (0.72 pmol) enzyme was incubated with 1 mM substrate in 50 mM sodium acetate (pH 5.0) for 5 min at 37°C and the reaction was stopped by boiling. The release of the glucose was determined by the peroxidase/glucose oxidase (PGO) assay method and visualized on TLC, as previously described [18,24]. The enzyme was also tested with polysaccharides. In the assay, 1–5 μg enzyme was incubated separately with 0.5% (w/v) laminarin and barley β-glucans in 50 mM sodium acetate (pH 5.0) at 37°C for 30–60 min. The reaction was stopped by the addition of *p*-hydroxybenzoic acid hydrozide reagent as described by [72], and the increase in reducing sugars was measured colorimetrically.

The glycon specificity of recombinant Os4bglu12 β-glucosidase was tested against synthetic substrates, *p*NP-glycosides. In a 100 μL reaction, 0.05 μg (0.72 pmol) enzyme was incubated with 1 mM *p*NP-glycoside substrate in 50 mM sodium acetate buffer, pH 5.0, for 5 min at 37°C. Then, 70 μL of 0.4 M sodium carbonate was added to stop the reaction, and the absorbance of the liberated *p*NP was measured at 405 nm. One unit of β-glucosidase activity was defined as the amount of enzyme that produced 1 μmol of product per min. Protein assays were performed by the Bio-Rad protein assay kit (Bio-Rad, Richmond, CA) using bovine serum albumin as a standard.

The pH optimum was determined by measuring the release of *p*NP from *p*NPG in different 50 mM buffers ranging in pH from 3.5 to 10 in 0.5 pH unit increments for 10 min (formate, pH 3.5–4.5; sodium acetate, pH 4.0–5.5; sodium phosphate, pH 5.5–8; Tris, pH 7.5–9.0; CAPS, pH 9.0–10). To find the temperature optimum, *p*NPG hydrolysis was measured in 50 mM sodium acetate (pH 5.0) at temperatures ranging from 5°C to 90°C in 5° increments for 10 min.

## Abbreviations

BGI, Beijing Genomic Institute; CDS, coding sequence; DP, degree of polymerization; EST, expressed sequence tag; GH1, glycosyl hydrolase family 1; IAA, indole-3-acetic acid; IMAC, immobilized metal affinity chromatography; pI, isoelectric points; MW, molecular weights; ORFs, open reading frames; *p*NP, *p*-nitrophenol; *p*NPG, *p*-nitrophenyl-β-D-glucoside; PGO, peroxidase/glucose oxidase.

## Authors' contributions

RO carried out the sequence analysis, participated in recombinant protein production and enzyme assay, and drafted the manuscript. BP carried out the enzyme assay. TO carried out cDNA cloning and recombinant protein production. TA participated and advised in enzyme assays and manuscript development. AE advised in sequence analysis and manuscript correction. JKC carried out sequence analysis, phylogenetic analysis, and drafted the manuscript. All authors read and approved the final submission.

## Supplementary Material

Additional File 1 **Alignment of full-length derived sequences of rice and *Arabidopsis *showing full predicted sequences**. All the full-length predicted proteins from rice GH1 genes, including Os11bglu36, which is from a distinct GH1 lineage, but not its *Arabidopsis *homologue and the possible endophyte genes Osbglu39 and Osbglu40, were aligned with ClustalX and the alignment adjusted and shaded with Genedoc, as described in the methods. Darkest shading represents highest conservation, and the consensus for highly conserved regions is shown below the alignment. The file was exported as a rich text (.rtf) document for this picture. Click here for file

Additional File 2 **Alignment of derived sequences of rice and *Arabidopsis *after removal of end regions and large gaps for use in phylogenetic tree generation**. The alignment in Additional file 1 was edited in Genedoc to remove the nonconserved N-terminal and C-terminal sequences and most of the large gap regions. This adjusted alignment was used for generation of the phylogenetic trees shown in Figure 2. Darkest shading represents highest conservation, and the consensus for highly conserved regions is shown below the alignment. The file was exported as a rich text (.rtf) document for this picture. Click here for file

Additional File 3 **Alignment of full-length derived sequences of rice and other plant GH1 enzymes**. All the full-length predicted proteins from rice GH1 genes, including Os11bglu36, which is from a distinct GH1 lineage, but not the possible endophyte genes *Osbglu39 *and *Osbglu40*, were aligned the related sequences defined in Figure 3 using ClustalX. The alignment was edited and shaded with Genedoc, as described in the methods. Darkest shading represents highest conservation, and the consensus for highly conserved regions is shown below the alignment. The file was exported as a rich text (.rtf) document for this picture. Click here for file

Additional File 4 Supplementary Table 1: Most predominant genes in terms of EST numbers in cereals. Click here for file

## Figures and Tables

**Table 1:** Summary of identified genes homologous to glycosyl hydrolase family 1 glucosidase

Gene name	BGI ID (AAAA...)^a^	RGP GenBank ID^c^	Gene locus ID/position^e^/Chr^f^	Gene pattern	Corresponding cDNAs^g^	Number ESTs^h^	Tissue libraries^i^	Comment
*Os1bglu1*	02002143 (F) 02002142 (aa 110–189^b^)	AP003217 (F) (BAD73293^d^) AP008207 (F)	AP008207/17752382 bp-17760802 bp/chr 1	2	AK069177 (F) AK060988 (n)	13	sh, pn, wh-TL, 2 wk lf-ABF3	
*Os1bglu2*	02004130 (aa 1–105) 02004129 (aa 106–561)	AP003570 (F) AP004331 (F) AP008207 (F)	AP008207/34595732 bp-34582220 bp/chr 1	1	-	4	pn-FW, wh-TL, 35 d lf-Dr, 3 wk lf-Bl	
*Os1bglu3*	02004130 (F) 02004127 (aa 134–288)	AP003570 (F) AP004331 (F) AP008207 (F)	AP008207/34604232 bp-34599017 bp/chr 1	1	AK067934 (F?) AK063065 (n)	4	sh, 2 wk lf-ABF3, 3 wk lf-Bl	
*Os1bglu4*	02004468 (aa 1–414) 02004470 (aa 426–479)	AP003349 (F) (BAD82183) AP003418 (F) (BAD82346) AP008207 (F)	AP008207/38998348 bp-39003033 bp/chr 1	1	-	9	sh, pn-FW, pn-FW-Dr, 3 wk lf-Bl	
*Os1bglu5*	02004619 (F)	AP003272 (F) (BAD87322) AP004330 (F) (BAD88178) AP008207 (F)	AP008207/40834604 bp-40840341 bp/chr 1	1	AK070499 (F) AK119221 (F)	23	sh, st-IM, pn, pn-FW, wh-TL, wh-BT, wh-TF, 2 wk lf-AtJMT, lf-Dr, 3 wk lf-Ls	
*Os3bglu6*	02008013 (F)	AC146619 (F) AP008209 (F)	AP008209/5850657 bp-5844110 bp/chr 3	1	AY129294 (F) AK119546 (F)	14	sh, pn-FW, cl-Co, 3 wk lf-Bl	
*Os3bglu7*	02010831 (aa 1–99) 02006516 (aa 100–504)	AC091670 (F) (AAX95519) AC133334 (F) (AAS07254) AP008209 (F)	AP008209/28041529 bp-28037050 bp/chr 3	2	OSU28047(F)AK100165 (F) AK103027 (F) AK105026 (F) AK059920 (n)	326	cl, sh, rt-SD, st-IM, pn, pn-FW, wh-TL, cl-ABA, cl-NAA, cl-BAP, cl-Cd, cl-heat, cl-Co; sh-UV, sh-Co, 35 d lf-Dr, 3–4 wk rt-Sa, 2 wk lf-ABF3, 2 wk cl-HDAC1, 3 wk lf-Bl, lf-M-Bl	*bglu1 *^j^
*Os3bglu8*	02010831 (F)	AC091670 (F) (AAX95520) AC133334 (F) (AAS07251) AP008209 (F)	AP008209/28050325 bp-28045526 bp/chr 3	2	AK120790 (F) AK105850 (n)AK059517 (n)	77	sh, pn, pn-FW, wh-TL, wh-TF, cl-BAP, sh-Co, 2 wk lf ABF3	
*Os4bglu9*	02014146 (F)	AL731582 (F) AP008210 (F)	AP008210/23697091 bp-23691010 bp/chr 4	1	AK066908 (F?)	11	sh, lf-IM, 3–4 wk rt-Sa	
*Os4bglu10*	02014151 (F)	AL731582 (F) (CAE05481) AP008210 (F)	AP008210/23708851 bp-23703582 bp/chr 4	1	AK065793 (F) AK062029 (F) AK073031 (n) AK068304 (n)	17	sh, lf-M, wh-TL, 2 wk lf ABF3, 2 wk lf- AtJMT, 3 wk lf-Bl	
*Os4bglu11*	02014151 (F)	AL731582 (F) (CAE05482) AP008210 (F)	AP008210/23717688 bp-23710742 bp/chr 4	1	-	4	sh-Co	
*Os4bglu12*	02014151 (F)	AL731582 (F) (CAE05483) AP008210 (F)	AP008210/23728066 bp-23723058 bp/chr 4	1	AK062776 (n) AK100820 (n) AK105375 (n)	30	cl, sh, 2 wk lf and rt, sp, wh-TL, wh-TF, 1 wk rt-Sa, sd-Co, pn-FW-Dr, 2 wk cl-HDAC1, 2 wk sd-Ph, 3 wk lf-Bl, lf-BT-Xa	
*Os4bglu13*	02014151 (F) 02014154 (aa 465–520)	AL73182 (F) (CAE05485) AP008210 (F)	AP008210/23742711 bp-23738108 bp/chr 4	1	AK070962 (F)	22	sh, pn, wh-TL, wh-TF, 3 wk lf-Wd, 3 wk lf-Bl, lf-M-Bl	
*Os4bglu14*	02014354 (F)	AL606622 (F) (CAE03397) AP008210 (F)	AP008210/25617791 bp-25613930 bp/chr 4	3	AK067841 (F)	1	sh	
*Os4bglu15*	02014355	AL606622 (n) (CAE003399) AP008210 (n)	AP008210/25626016 bp-25623565 bp/chr 4	-	-	0		Gene fragment, lacks exon 1–8
*Os4bglu16*	02014360 (aa 1–69) 02014359 (aa 70–516)	AL606622 (F) (CAE54544) AL606659 (F) (CAE01908) AP008210 (F)	AP008210/25631832 bp-25640157 bp/chr 4	3	AK066850 (F?) AK068772 (F?)	14	rt-SD, sh, pn, pn-FW, wh-TL, cl-Co, 3 wk rt-Sa, 3 wk lf-Bl, lf-M-Bl	
*Os4bglu17*	02014358	AL606622 (n) AL606659 (n) AP008210 (n)	AP008210/25646002 bp-25648366 bp/chr 4	-	-	0		Gene fragment lacks exon 9–13
*Os4bglu18*	02014362 (aa 1–46) 02014361 (aa 47–505)	AL606622 (F) (CAE01910) AL606659 (F) (CAE54546) AP008210 (F)	AP008210/25667349 bp-25654991 bp/chr 4	3	AK058333 (n)	10	sh, pn-FW, 3 wk lf-Bl	
*Os5bglu19*	02017035 (F) 02016858 (aa 1–272)	AC121366 (F) (AAS79738) AC135927 (F) AC137618 (F) AP008211 (F)	AP008211/17386160 bp-17389960 bp/chr 5	1	AK105546 (F?)	5	pn-FW, pn-FW-Dr, 2 wk lf- AtJMT, 3 wk lf-Wd	
*Os5bglu20*	02016859 (F) 02017035 (F)	AC121366 (F) AC137618 (F) AP008211 (F)	AP008211/17403620 bp-17407871bp/chr 5	1	AK120998 (F?)	0		
*Os5bglu21*	02016862 (F)	AC121366 (F) AC137618 (F) AP008211 (F)	AP008211/17421799 bp-17427364 bp/chr 5	1	-	0		
*Os5bglu22*	02016869 (F) 02016867 (aa 1–61)	AC121366 (F) AC137618 (F) (AAV31358) AP008211 (F)	AP008211/17450999 bp-17456012 bp/chr 5	1	AK071469 (F)	39	sh, lf-M, pn-FW, cl-BAP, cl-NAA, 3 wk lf-Ls, 3 wk lf-Bl, lf-M-Bl	
*Os5bglu23*	02016873 (F) 02016872 (aa 251–380)	AC137618 (F?) AC104279 (F?) AP008211 (F?)	AP008211/17470463 bp-17477059 bp/chr 5	3	-	0		AC137618 AC104279 AP008211 frameshift in exon 1
*Os6bglu24*	02019101 (F)	AP003543 (F) (BAD61620) AP008212 (F)	AP008212/12285539 bp-12280797 bp/chr 6	1	-	0		
*Os6bglu25*	02020792 (F)	AP003766 (F) AP004797 (1–284) AP008212 (F)	AP008212/28093582 bp-28097231 bp/chr 6	1	AK120488 (F?) AK068614 (F?)	4	sh, pn-FW, 3 wk lf-Bl	
*Os7bglu26*	02022575 (F)	AP005182 (F) AP005184 (F) AP008213 (F)	AP008213/27562097 bp-27564748 bp/chr 7	2	AK068499(F?)	30	cl, sh, 2 wk lf, pn, pn-FW, pn-RP, 3 wk lf-Bl	
*Os8bglu27*	02025921 (F) 02025924 (aa 403–499)	AP005816 (F) (BAD10670) AP006049 (F) (BAC57391) AP008214 (F)	AP 008214/25247245 bp-25243519 bp/chr 8	1	AK067001(F) AK067231 (F) AK120430 (F)	19	sh, wh-TF, lf-TF, pn-FW, sh-Co, 2 wk lf- AtJMT, 3 wk lf-Bl	
*Os8bglu28*	02025922 (F)	AP006049 (F) (BAC57391) AP005816 (BAD10672) AP008214 (F)	AP008214/25259660 bp-25253178 bp/chr 8	1	AK105908 (F) AK059210 (F) AK098938 (F)	12	cl, sh, 35 d lf-Dr	
*Os9bglu29*	02027760 (F)	AC108758 (F) AC108762 (F) AP008215 (F)	AP008215/18724216 bp-18720410 bp/chr 9	4	-	2	rt-SD	
*Os9bglu30*	02027762 (F)	AC108758 (F) AC108762 (F) AP008215 (F)	AP008215/18739405 bp-18736646 bp/chr 9	4	AY056828(F) AK066710 (F) AK104707 (n) AK061340 (n)	27	sh, 2 wk lf, lf-IM, st-IM, pn, pn-FW, wh-TL, sh-UV, 2 wk lf-ABF3	*bglu2 *^j^
*Os9bglu31*	02027832 (F)	AC137594 (F) AP008215 (F)	AP008215/19592828 bp-19587946 bp/chr 9	3	AK121679 (F) AK102869 (F), AK121935 (F?)	48	cl, sh, rt-SD, lf, pn-FW, pn-RP, isd, wh-TL, cl-NAA, cl-BAP, cl-Cd; 2 wk cl-HDAC1, sc-Ac, 3 wk lf-Ls	
*Os9bglu32*	02027836 (F)	AC137594 (F) AP006752 (F) AP008215 (F)	AP008215/19609411 bp-19606016 bp/chr 9	1	AK101420 (F?)	31	cl, sh, rt-SD, pn-FW, isd, wh-TL, wh-BT, wh-TF, pn-FW-Dr, 3 wk lf-Bl, lf-M-Bl	
*Os9bglu33*	02027845 (aa 1–399) 02027838 (aa 435–501)	AC137594 (F) AP006752 (F) AP008215 (F)	AP008215/19619402 bp-19614063 bp/chr 9	1	AK066336 (F)	4	sh, pn-FW, 3 wk lf-Bl	
*Os10bglu34*		AC074354 (F) (AAK9258) AE016959 (F) AP008216 (F)	AP008216/8447928 bp-8449554 bp/chr 10	1	AK071372 (F)	1	pn	
*Os11bglu35*		AC134047 (F?) (AAY23259) AP008217 (F?)	AP008217/4243306 bp-4245678 bp/chr 11	3	-	0		pseudogene
*Os11bglu36*	02033149 (F)	AC135190 (F) AP008217 (F)	AP008217/26778370 bp-26774474 bp/chr 11	-	AJ491323 (F) AK119461 (F) AK067619 (F?)	11	sh, 1 wk lf-Sa, 3 wk lf-Bl, 3 wk lf-Ls,	
*Os11bglu37*	02030895 (F)	-	AAAA02030895/43041 bp-40310 bp/chr 11	1	-	0		Pseudogene has stop after aa 434
*Os12bglu38*	02034198 (F) 02034197 (aa 1–113)	AL731785 (F) AL732381 (F) AP008218 (F)	AP008218/13144002 bp-13146818 bp/chr 12	2	AK071058 (F)	11	sh, sp, pn-FW, pn-FW-Dr	
*Osbglu39*	02042985 (F)	-	AAA02042985 bp/1652–3025 bp/chr -	5	-	0		Intronless
*Osbglu40*	02048307 (n)	-	AAAA02048307/815 bp -3 bp/chr -	5	-	0		Intronless, lacks exon 10–13

^a ^contig number in Beijing Genome Institute (the number start with 'AAAA').

^b^aa means the length of gene where its CDS covers the given range of amino acid residues.

^c ^GenBank accession number. F means full length gene/cDNA, n is not.

^d ^annotated deduced β-glucosidase in GenBank.

^e ^chromosome location was determined by mapping of corresponding gene on the 12 rice chromosomes in GenBank.

^f ^Chr means the number of the chromosome onwhich the gene is located.

^g^the full-length cDNA clones of *japonica *rice databases (Kikuchi *et al*. [50])

^h^Number EST means number of ESTs that match each gene. EST sequences were retrieved from the dbEST section of NCBI GenBank by BLASTn search with gene sequences. They were inspected to ensure they matched the gene-coding region and their full files retrieved to determine cDNA library source tissue and clone number when necessary. The ESTs assigned to each gene had greater than 97% identity and no higher similarity with another gene.

^i^The type of library where the conrresponding ESTs were found. Tissues: cl: callus, isd: immature seed, lf: leaf, pn: panicle or flower, rt: root, sc: suspension culture, sh: shoot, sp: spikelet before heading, st: stem, wh: whole plant. Stages (capital letters): BT: booting, FW: flowering, IM: 3–5 leaf stage or immature stage, M: mature, RP: ripening, SD: seedling, TF: trefoil, TL: tillering, 1 wk: 1 week-old, 2 wk: 2 week-old, 3 wk: 3 week-old, 3–4 wk: 3–4 week-old, 35 d: 35 day-old Growth or stress conditions: Cd: Cadmium, Co: cold, Dr: drought, heat: heat, Sa: salt, UV: UV light, Wd: wound, ABA: abscissic acid, BAP: benzyl amino purine, NAA: naphthaleneacetic acid, Bl: blast infected, Ls: lession mimics, Ph: brown plant hopper infested, Xa: *Xanthomonas oryzae *induced, Ac: *Acidovorax avenae *infected, ABF3: ABA-responsive element binding TF3 overexpression, AtJMT: *Arabidosis *jasmonate carboxyl methyltransferase overexpression, HDAC1: histone deacetylase overexpression.

^j ^Opassiri *et al*. [24]

**Table 2:** Predicted rice GH family 1 β-glucsidase protein properties and locations.

Gene name	Gene ID	Pre-protein			Mature protein				
		
		MW^a^	AA^b^	Cleavage site^c^	MW^a^	AA^b^	pI^a^	N-gly site^d^	Possible destination^e^
*Os1bglu1*	AP003217 (BAD73293)	58.0	516	21–22	55.9	495	7.78	5	Out, per, ERm, ERl
*Os1bglu2*	AP003570	62.4	561	44–45	57.6	517	5.21	3	M inn, plas, chl, m int
*Os1bglu3*	AP003570	57.5	514	22–23	55.3	492	7.29	3	Out, per, ERm, ERl
*Os1bglu4*	AP003349 (BAD82183)	55.3	483	-	-	-	5.16	0	Per, cyt, m mat, ERm
*Os1bglu5*	AP003272 (BAD87322)	57.4	513	26–27	54.9	487	5.31	3	Out, per, ERm, ERl
*Os3bglu6*	AC146619	58.5	521	31–32	55.4	490	6.36	3	Out, per, ERm, ERl
*Os3bglu7*	AC091670	56.9	504	26–27	54.3	478	8.96	3	Out, per, ERm, ERl
*Os3bglu8*	AAAA02010831	63.1	568	33–34	59.7	535	6.21	3	Plas, per, ERm, ERl
*Os4bglu9*	AAAA02014146	58.3	514	28–29	55.6	486	7.73	4	Out, vac, per, ERm
*Os4bglu10*	AL731582 (CAE05481)	58.1	510	23–24	55.8	487	8.07	4	Out, vac, per, nuc
*Os4bglu11*	AL731582 (CAE05482)	59.8	530	25–26	57.4	505	7.29	4	Out, vac, per, nuc
*Os4bglu12*	AAAA02014151	57.5	510	24–25	55.3	486	8.85	6	Out, vac, per, ERm
*Os4bglu13*	AL731582 (CAE05485)	57.1	506	25–26	54.8	481	6.66	6	Out, vac, per, ERm
*Os4bglu14*	AL606622 (CAE03397)	58.8	516	23–24	56.4	493	7.69	6	Out, per, ERm, ERl
*Os4bglu15*	AL606622 (CAE003399)	-	-	-	-	-	-	-	-
*Os4bglu16*	AL606622 (CAE54544)	58.6	516	27–28	56.0	489	6.13	4	Out, per, ERm, ERl
*Os4bglu17*	AL606622	-	-	-	-	-	-	-	-
*Os4bglu18*	AL606659 (CAE54546)	57.6	505	26–27	55.0	479	5.3	1	Out, per, ERm, ERl
*Os5bglu19*	AC121366 (AAS79738)	59.8	530	31–32	56.2	499	5.05	6	Out, per, ERm, ERl
*Os5bglu20*	AAAA02016859	58.6	520	30–31	55.1	490	5.23	5	Out, per, ERm, ERl
*Os5bglu21*	AAAA02016862	59.2	526	34–35	55.3	492	5.67	4	Out, vac, per, ERm
*Os5bglu22*	AC137618 (AAV31358)	59.5	533	24–25	57.1	509	4.96	5	Out, vac, per, ERm
*Os5bglu23*	AAAA02016873	58.5	523	27–28	55.8	496	5.19	3	Out, vac, per, ERm
*Os6bglu24*	AP003543 (BAD61620)	57.8	504	18–19	55.8	486	7.78	5	Out, per, ERm, ERl
*Os6bglu25*	AP003766	57.2	501	19–20	55.2	482	5.51	2	Out, per, ERm, ERl
*Os7bglu26*	AP005182	58.5	510	27–28	55.6	483	6.49	6	M inn, per, plas, m int
*Os8bglu27*	AAAA02025912	56.8	499	19–20	54.8	480	8.36	5	Out, per, ERm, ERl
*Os8bglu28*	AP006049 (BAC57391)	56.6	500	24–25	53.9	476	8.4	6	M out, vac, out, per
*Os9bglu29*	AC108758	57.7	508	28–29	54.8	480	8.76	4	Out, per, ERm, ERl
*Os9bglu30*	AC108758	57.4	500	25–26	54.6	475	6.99	6	Out, vac, per, ERm
*Os9bglu31*	AC137594	58.4	523	22–23	56.3	501	5.32	2	Out, per, ERm, ERl
*Os9bglu32*	AAAA02027836	57.1	510	30–31	54.1	480	5.51	2	Out, per, vac, ERm
*Os9bglu33*	AC137594	56.8	503	30–31	53.8	473	5.62	2	Out, vac, per, ERm
*Os10bglu34*	AAAA02028915	58.0	510	26–27	55.3	484	6.34	5	Out, per, ERm, ERl
*Os11bglu35*	AC134047	-	-	-	-	-	-	-	-
*Os11bglu36*	AC135190	73.2	647	26–27	70.8	621	6.1	1	M inn, per, ERm, m int, chl
*Os11bglu37*	AAAA02030895	-	-	-	-	-	-	-	-
*Os12bglu38*	AL731785	57.0	492	21–22	54.8	471	7.44	5	Out, per, ERm, ERl
*Osbglu39*	AAAA02042985	53.0	458	-	-	-	5.91		Per, cyt, m mat, chl
*Osbglu40*	AAAA02048307	-	-	-	-	-	-	-	-

^a ^determined by ProtParam, ^b ^AA means number of amino acids, ^c^predicted by SignalP, ^d^predicted by NetNGlyc at the Expasy proteomics server [69], ^e^cellular locations predicted by PSORT. Chl: chloroplast; cyt: cytoplasm; ERm: endoplasmic reticulum membrane; ERl: endoplasmic reticulum lumen; m inn, m int, m mat, m out: mitochondria inner membrane, intermembrane space, matrix, outer membrane, respectively; per: peroxisome; plas: plasma membrane; vac: vacuole.

**Table 3:** Substrate specificity of the purified rice Os4bglu12

**Substrate**	**Relative activity**^**a**^**(%)**
Laminaribiose^b^	238
Laminaritriose	0
Laminaritetraose	0
Laminaripentaose	0
Cellobiose	0
Cellotriose^b^	231
Cellotetraose^b^	301
Cellopentaose^b^	279
Cellohexaose^b^	295
Gentiobiose	0
Laminarin	0
Barley 1,3, 1,4-β-glucans	0
*p*NP-β-D-glucoside	100
*p*NP-β-D-fucoside	118
*p*NP-β-D-galactoside	45
*p*NP-β-D-xyloside	45
*p*NP-α-L-arabionoside	26
*p*NP-β-D-mannoside	0
*p*NP-β-D-cellobioside	0
*p*NP-α-D-glucoside	0
*p*NP-β-L-fucoside	0

^a^Percent activity relative to glucose or *p*NP released from *p*NP-β-D-glucoside. The assay contained 1 mM substrate in 50 mM sodium acetate (pH 5.0) buffer at 37°C. ^b^Note that the values for oligosaccharides are in terms of total glucose released.
