# Supplementary material for: Analysis of rice glycosyl hydrolase family 1 and expression of Os4bglu12 β-glucosidase
Source: BMC Plant Biol. 2006 Dec 29;6:33. doi: 10.1186/1471-2229-6-33 (PMC1781453; doi:10.1186/1471-2229-6-33)
Supplement: Additional File 4 — Supplementary Table 1: Most predominant genes in terms of EST numbers in cereals. [file 1471-2229-6-33-S4.doc]

**Supplementary Table 1** Most predominant genes in terms of EST numbers in cereals.

| Gene | No. of ESTs | location | Note |
| --- | --- | --- | --- |
| BGlu1-like  1. *Os1bglu1*  2. *Os3bglu7*  3. *Os3bglu8*  4. *Os6bglu26*  5. *Os12bglu38* | 11  326  77  30  11 |  |  |
| Sorghum (found 2 genes)  1. AAC49177 *bglu1*  2. AF253508 (72% identity with  AAC49177) | 302  300 | Chloroplast  Chloroplast |  |
| Wheat (found 3 genes)  1. AB236422  2. AB100035 (96% identity  with AB236422)  3. AB236423 (93% identity with  AB236422) | 3 genes have 162 hits | Chloroplast  Chloroplast  Chloroplast |  |
| Rye (found 2 genes)  1. AF293849  2. BE494053 (in EST db) | 5  1 | Chloroplast  Chloroplast |  |
| Maize (found 11 genes)  1. AAB03266  2. AY109328  3. U44087  4. BT016439  5. AY106991  6. AM238660 (sfr2)  7. AY109400  8. BT018598  9. AY106297  10. AY106943  11. AY111218 | 318  330  27  109  24  10  112  16  20  8  8 | Chloroplast  Chloroplast  Chloroplast  Chloroplast  No chloroplast  Chloroplast  Chloroplast  Cannot predict  Cannot predict  Cannot predict  Cannot predict | No 5’region  No 5’region  No 5’region  No 5’region |
| Oat (found 2 genes)  1. AF082991  2. X78433 | 1  17 | Chloroplast  Chloroplast |  |
| Barley (found 4 genes)  1. L41869 (BGQ60)  2. EST (94-97% identity with BGQ60)  3. BF620280 (60% identity with BGQ60)  4. BF627691 (71% identity with BGQ60)  5. Est partial sequences | 15  8  1  1  15 | No chloroplast  No chloroplast  Chloroplast  Cannot predict | No 5’region |
