# Supplementary material for: Analysis of rice glycosyl hydrolase family 1 and expression of Os4bglu12 β-glucosidase
Source: BMC Plant Biol. 2006 Dec 29;6:33. doi: 10.1186/1471-2229-6-33 (PMC1781453; doi:10.1186/1471-2229-6-33)
Supplement: Additional File 1 — Alignment of full-length derived sequences of rice and Arabidopsis showing full predicted sequences. All the full-length predicted proteins from rice GH1 genes, including Os11bglu36, which is from a distinct GH1 lineage, but not its Arabidopsis homologue and the possible endophyte genes Osbglu39 and Osbglu40, were aligned with ClustalX and the alignment adjusted and shaded with Genedoc, as described in the methods. Darkest shading represents highest conservation, and the consensus for highly conserved regions is shown below the alignment. The file was exported as a rich text (.rtf) document for this picture. [file 1471-2229-6-33-S1.doc]

Supplementary Figure 1A: Alignment of deduced rice and *Arabidopsis* protein sequences.

```

                      *        20         *        40         *        60         *
Os10bglu34 : ------------------------------MGNGGRC--------MVEVVILLVLMAMSQ--------------- :  22
Os3bglu6   : --------------------------MGRIKSSSGRCSTARLEAVAVLVVVFGVASSSLR--------------- :  34
AtBGLU40   : ------------------------------MAHRRLIMT-----MTKMMMMVTMMMMMDK--------------- :  25
Os6bglu25  : ------------------------------------------MSLLTLVHILVSFS------------------- :  14
AtBGLU41   : -----------------------------------------MESLMRLVLVLFPFFVVFF--------------- :  19
Os1bglu4   : -----------------------------------------------MGSTG----------------------- :   5
AtBGLU42   : -----------------------------------------------MAQKLNLLN--L---------------- :  10
Os1bglu1   : ---------------------------MGHRLVVVLL-------LALLVAGAARAAEQAA--------------- :  26
Os7bglu26  : ----------------------------MRKFIAALR-------LALAAAAHLLLTLPPA--------------- :  25
AtBGLU43   : ---------------------------------MFLF-------LLLLSASRSGEESPSG--------------- :  20
AtBGLU44   : ------------------------MRHLSSPPWPLLL-------LLLLSSFTSGESSLSA--------------- :  29
Os3bglu8   : ---------------------------MGCAPAAHYLPGGGRRRLLVVVVALVVLDRAGA--------------- :  33
Os3bglu7   : ---------------------------MAARRANCAL-------VLVLALALLAARDAGA--------------- :  26
Os12bglu38 : ---------------------------------MNMP--------LLLLIAIVVVSLSHG--------------- :  19
Os11bglu37 : ------------------------------------MGIRMGRRLLLITLLLGALLCNN---------------- :  23
Os11bglu35 : ------------------------------------MGIRMGRRLLLITLLLGALLCNN---------------- :  23
Os9bglu30  : ------------------------------------MGIRMGRR-LLFTLFLGALFCN----------------- :  21
Os9bglu29  : ---------------------------------MAWLGIGMGRQ-IVPVLVFVAVLCS----------------- :  24
Os8bglu28  : ----------------------------------------MDRRLLLSALLFIALACSSN--------------- :  20
Os8bglu27  : ------------------------------------------MRWLLLALFLVALVSNGA--------------- :  18
Os4bglu10  : ---------------------------------MAVAGAMVMSGGVLLL-LLAFTCAAYN--------------- :  26
Os4bglu11  : ---------------------------------MAVAGAMVMSGALLLLHLLAFTCVACN--------------- :  27
Os4bglu9   : ---------------------------------MAVAGAVAMSGGILLL-LLLLLAAACV--------------- :  26
Os4bglu13  : ---------------------------------MAAAGEVVMLGGILLPLLLVVAVS------------------ :  24
Os4bglu12  : ---------------------------------MAAAG--AMPGGLLLTFLLLAVVASGAYN------------- :  27
Os6bglu24  : ---------------------------------MELLW-------LLLLLLMASSTSSRSEM------------- :  22
AtBGLU17   : ---------------------------------MAIKSIFIIIIISIITSISELYALDPS--------------- :  27
AtBGLU12   : ---------------------------------------MRTIYLSLLVFIIVLALNEVMAK------------- :  23
AtBGLU13   : ---------------------------------------MRTKYFSLLVFIIVLASNEVIAK------------- :  23
AtBGLU14   : ---------------------------------------MTSKYFSVLVFII-LASNEVVAK------------- :  22
AtBGLU15   : ---------------------------------------MRGNYLSLLVVLIVLASNDVLAN------------- :  23
AtBGLU16   : ---------------------------------------MISHKISFLDLLLLLIS---VKE------------- :  20
AtBGLU18   : --------------------------------MVRFEKVHLVLGLALVLTLVGAPTKAQ---------------- :  27
AtBGLU19   : ------------------------------------MKIP-LLGLLLLISLVGSPTRAE---------------- :  22
AtBGLU20   : --------------------------------MGRFHKFP-LLGLVLFLGLTGSLIAAN---------------- :  26
AtBGLU21   : ---------------------------------MALQKFPLMGL-LLLLTILVSVTTAV---------------- :  25
AtBGLU22   : ---------------------------------MALQKFPLLGL-LFLITIVVSSTIAV---------------- :  25
AtBGLU23   : ---------------------------------MVLQKLPLIGL-LLLLTIVASPANA----------------- :  24
AtBGLU24   : ---------------------------------MVLQKLPLMSIGLLWLLIIVGPLVNA---------------- :  26
AtBGLU25   : ------------------------------------MALKAILFLGLFLVVIVSPITVY---------------- :  23
AtBGLU26   : ---------------------------------------------MAHLQRTFPTEMS----------------- :  13
AtBGLU27   : ---------------------------------------------------MYSKKNS----------------- :   7
AtBGLU28   : -----------------------------------------MKMHFFILLVITSWLSEK---------------- :  18
AtBGLU29   : -----------------------------------------MNVQIFILLLIISWLTPK---------------- :  18
AtBGLU30   : ---------------------------------------MAKGSWFFIILFIISMLENM---------------- :  20
AtBGLU31   : --------------------------------------MAIKLIALVITLCVASWDVAQ---------------- :  21
AtBGLU32   : --------------------------------------MAIKLIALVITICVASWDSAQ---------------- :  21
AtBGLU33   : ---------------------------------MATATLTLFLGLLALTSTILSFNADARPQPSDEDLGTIIGPH :  42
AtBGLU34   : --------------------------------MAIPKAHYSLAVLVLLFVVVSSSQKVCN--------------- :  28
AtBGLU35   : --------------------------------MAIPKAHYSLAVLVLLFVVVSSSQKVCN--------------- :  28
AtBGLU36   : --------------------------------------------------MQSRMQGQRT--------------- :  10
AtBGLU37   : ---------------------------MQHNTYIYILTMKLLGFALAILLVVATCKPEEE--------------- :  33
AtBGLU38   : --------------------------------------MKLLMLAFVFLLALATCKGDE---------------- :  21
AtBGLU39   : --------------------------------------MKFRALGLVLLLAVETCKAEE---------------- :  21
Os4bglu18  : ---------------------------------------MAGGSKTRIHASLVSTLLLL---------------- :  20
Os4bglu14  : ------------------------------------------MAAAWLVVLLTVHRLLH---------------- :  17
Os4bglu16  : ---------------------------------------MAVAAATRIAVVVVVLALAV---------------- :  20
AtBGLU45   : -----------------------------------MKNLTSFVIVILLQSLLFHVYG------------------ :  22
AtBGLU46   : -----------------------------------MKTFANFAILFLLQSLLFPLYSSC---------------- :  24
AtBGLU47   : ----------------MKKSIVYEIMETKSSMYLSQFRLWLCFIITTLVSLSSSTRWYD---------------- :  43
Os9bglu33  : -----------------------------------MATGELALVSSLFIVVVFLLLGAV---------------- :  24
Os9bglu32  : -----------------------------------MAAGARALVPSPFIVVVFLLLAAA---------------- :  24
Os9bglu31  : ------------------------------------MTPARVVFICCVVLLAAAAAAAS---------------- :  23
Os1bglu2   : MDVLILSSRAGSQTSLISLQKHNRRSNLLLILFFLLIIMGAAAAAGFFFVLLFLSVQGGAVG------------- :  62
Os1bglu3   : ----------------------------------------MAAAAAFFCALLFISVQHGVLGG------------ :  23
Os1bglu5   : ---------------------------------------MAAAIAVVYLSLLLLLLHGAAPAV------------ :  24
Os5bglu22  : ----------------------------------MAVSSSTSTCSSFSLLLLLLLLAAAPWRSG------EA--- :  32
Os5bglu23  : ------------------------------------MAAYTSSLVSLLLLLLLLLLVAG---------------- :  23
Os5bglu20  : -----------------------------ME-RRPLHLHLLLLFFSAWLMLLPLL-------------------- :  25
Os5bglu21  : -----------------------------METRRPLHL-LLVFLSSPWLLLLLLLLLVV---------------- :  29
Os5bglu19  : -----------------------------METRRPLHP--LLLFFSPWLLLLLLL--VV---------------- :  26
AtBGLU1    : ----------------------------------------MEDVLTLITMIVLLLLAFHGFG------------- :  22
AtBGLU3    : ----------------------------------------MELTLSLLTIFLLFFALS---G------------- :  19
AtBGLU4    : ----------------------------------------MEQILALFAIFLAFAFSG----------------- :  18
AtBGLU5    : ----------------------------------------MEQFFALFTIFLSFAFPG----------------- :  18
AtBGLU6    : ----------------------------------------MEKTFALITIFLAFAFSG----------------- :  18
AtBGLU7    : -----------------------------------------MKPFSQFFVFVVTVSAT----------------- :  17
AtBGLU8    : -----------------------------------------MKHFNLLSIILVIVLAT----------------- :  17
AtBGLU9    : -----------------------------------------MKHFSLLFIFLVILLAT----------------- :  17
AtBGLU10   : -----------------------------------------MKLYSLLSVFLVILLAT----------------- :  17
AtBGLU11   : -----------------------------------------MKLLSNSLMFLPLLALALT--------------- :  19
Os11bglu36 : --------------------------------MPLPAFVAAAARLAVLVAAAATAANAAS--------------- :  28


                80         *       100         *       120         *       140         *
Os10bglu34 : -----------------------------GCDAQNT--------TG----GLTRKSFP-----------NGFVFG :  45
Os3bglu6   : -----------------------------GCIAQQS--------GG----GLTRGSFP-----------EGFVFG :  57
AtBGLU40   : -----------------------------TCICAD----------------ISRGSFP-----------KGFVFG :  44
Os6bglu25  : ----------------------------------AC--------VE----AISRADFP-----------PGFIFG :  32
AtBGLU41   : -----------------------------VPLDHVS--------SE----SISRANFP-----------DGFVFG :  42
Os1bglu4   : ----------------------------------RD--------AE----VT-RGDFP-----------DGFVFG :  22
AtBGLU42   : ----------------------------------AV--------PP----VTHRSNFP-----------STFTFG :  28
Os1bglu1   : -----------------------------GEDGIRGGAGADHQEAAGITGGLSRRSFP-----------AGFVFG :  61
Os7bglu26  : -----------------------------QCYWLN----PEIYDAG----GLSRRAFP-----------EGFVFG :  52
AtBGLU43   : -----------------------------DAVPLA---------TG----GLNRKSFP-----------EGFLFG :  42
AtBGLU44   : -----------------------------EKNKLH---------TG----GLSRQSFP-----------KGFVFG :  51
Os3bglu8   : -----------------------------RVRAAD-----D--DTG----GLSRAAFP-----------KGFVFG :  57
Os3bglu7   : -----------------------------AAVPKP-----N--WLG----GLSRAAFP-----------KRFVFG :  50
Os12bglu38 : -----------------------------NGEQTD----------------LTRETFP-----------AGFVFG :  38
Os11bglu37 : -------------------------------VAYAK---------------FSRYSFP-----------KDFIFG :  41
Os11bglu35 : -------------------------------VAYAK---------------FSRYSFP-----------KDFIFG :  41
Os9bglu30  : -------------------------------GVYAK---------------FTRYSFP-----------KDFIFG :  39
Os9bglu29  : -------------------------------GVDAS---------------FNRYSFP-----------KDFIFG :  42
Os8bglu28  : -------------------------------RVHGA---------------LNRHSFP-----------EGFLFG :  38
Os8bglu27  : -------------------------------AVHGA---------------FNRFSFP-----------EDFIFG :  36
Os4bglu10  : -----------------------------DAGELPP---------------ISRRSFP-----------KGFIFG :  46
Os4bglu11  : -----------------------------GGSELPP---------------ISRRSFP-----------KGFIFG :  47
Os4bglu9   : -----------------------------EAGELPP---------------ISRRSFP-----------KGFIFG :  46
Os4bglu13  : -------------------------------GEPPP---------------ISRRSFP-----------EGFIFG :  42
Os4bglu12  : -----------------------------SAGEPP----------------VSRRSFP-----------KGFIFG :  46
Os6bglu24  : -----------------------------KAGEVI-----------------RRSQFP-----------EDFFFG :  40
AtBGLU17   : -----------------------------FLRLSTS---------------LQRSSFP-----------QDFRFG :  47
AtBGLU12   : -----------------------------KHSSTPK---------------LRRSDFP-----------EDFIFG :  43
AtBGLU13   : -----------------------------KHSSTPK---------------LRRSDFP-----------KDFIFG :  43
AtBGLU14   : -----------------------------RHSSTPK---------------LRKTDFP-----------EDFIFG :  42
AtBGLU15   : -----------------------------NNSSTPK---------------LRRSDFP-----------EDFIFG :  43
AtBGLU16   : -----------------------------LHMKMVE---------------V----------------------- :  28
AtBGLU18   : ----------------------------GPVCGAGLPD------------KFSRLNFP-----------EGFIWG :  51
AtBGLU19   : ---------------------------EGPVCPK--TE------------TLSRASFP-----------EGFMFG :  45
AtBGLU20   : ----------------------------EYACSS-TDI------------HFTRANFP-----------KGFIFG :  49
AtBGLU21   : ---------------------------DDPVCPATS--------------KLSRASFP-----------NGFLFG :  48
AtBGLU22   : ---------------------------DDPVCPTTS--------------KLSRASFP-----------NGFVFG :  48
AtBGLU23   : ---------------------------DGPVCPPSN--------------KLSRASFP-----------EGFLFG :  47
AtBGLU24   : ---------------------------DGPVCPPKPSD------------KLSRAHFP-----------KGFLFG :  51
AtBGLU25   : ---------------------------GGAVCPASS--------------TFGRGSFP-----------DGFLFG :  46
AtBGLU26   : ---------------------------------------------------KGRASFP-----------KGFLFG :  26
AtBGLU27   : ---------------------------------------------------FGRSDFP-----------EGFLFG :  20
AtBGLU28   : ---------------------------ITSLPPDSR--------------VFDRHGFP-----------DNFVFG :  41
AtBGLU29   : ---------------------------ITSLPPESQ--------------VLDRSSFP-----------DDFVFG :  41
AtBGLU30   : ---------------------------INSL---E----------------LDRHSFP-----------DDFIFG :  38
AtBGLU31   : ---------------------------GRSLRFSTT--------------PLNRYSFP-----------PHFDFG :  44
AtBGLU32   : ---------------------------GRSLRFSTT--------------PLNRYSFP-----------PHFDFG :  44
AtBGLU33   : QTSFDDEIGIVIGPHATVDDEDIDMDMGTTVGPQTNLNDDDLGTIIGPEFEIHKQDFP-----------ADFIFG : 106
AtBGLU34   : -----------------------------PECKAKEPFH------CDNTHAFNRTGFP-----------RNFTFG :  57
AtBGLU35   : -----------------------------PECKAKEPFH------CDNTHAFNRSGFP-----------KNFTFG :  57
AtBGLU36   : -----------------------------LQLRQNS---------CIQPKWISQ---------------KNFTFG :  32
AtBGLU37   : -----------------------------ITCEENVPFT------CSQTDRFNKQDFE-----------SDFIFG :  62
AtBGLU38   : -----------------------------FVCEENEPFT------CNQTKLFNSGNFE-----------KGFIFG :  50
AtBGLU39   : -----------------------------ITCEETKPFT------CNQTDRFNRKHFD-----------DDFIF- :  49
Os4bglu18  : ------------------------------LPLAS---------------AIHRSDFP-----------ASFLFG :  39
Os4bglu14  : ------------------------------LSGVS---------------AVDRSQFP-----------PDFLFG :  36
Os4bglu16  : ------------------------------LAPAAR--------------GLRRDDFP-----------PGFLFG :  40
AtBGLU45   : ------------------------------RHQSSSKNI-----------LVDSSPFP-----------SDFLFG :  45
AtBGLU46   : ------------------------------LHQTSD----------------DSSPFP-----------SDFLFG :  42
AtBGLU47   : ------------------------------DHISLKEIH-----------AEETFHFP-----------KNFLFG :  66
Os9bglu33  : ------------------------------AREASA---------------LTRHDFP-----------EGFVFG :  43
Os9bglu32  : ------------------------------ARDASA---------------LTRHDFP-----------EGFVFG :  43
Os9bglu31  : ------------------------------SSTAAG---------------ITRADFP-----------PEFIFG :  42
Os1bglu2   : ---------------------------------------------------YTRSDFP-----------RDFVFG :  75
Os1bglu3   : ---------------------------------------------------YTRNDFP-----------ADFVFG :  36
Os1bglu5   : ----------------------------------LG---------------YTRGDFP-----------EDFVFG :  39
Os5bglu22  : ---------------------------AAAAARALN---------------FTRQDFP-----------GEFVFG :  54
Os5bglu23  : ---------------------------EATAEAALN---------------FTRQDFP-----------GDFVFG :  45
Os5bglu20  : -----------------------------QGVSSLQ---------------FRRDDFP-----------DGFAFG :  45
Os5bglu21  : -----------------------------QGMSSLQ---------------FTRDDFP-----------HDFAFG :  49
Os5bglu19  : -----------------------------QGVRSLQ---------------FTRDDFP-----------DGFTFG :  46
AtBGLU1    : ------------------------------KCSSDL---------------YSRSDFP-----------EGFVFG :  41
AtBGLU3    : ------------------------------RCS-------------------DKNDFP-----------EGFIFG :  34
AtBGLU4    : ------------------------------KCS-DV---------------FSRSDYP-----------EGFVFG :  36
AtBGLU5    : ------------------------------RCS-DV---------------FSRSDFP-----------EGFLFG :  36
AtBGLU6    : ------------------------------KCS-DV---------------FSRCDFP-----------EGFVFG :  36
AtBGLU7    : ------------------------------SYI-DA---------------FTRNDFP-----------NDFLFG :  35
AtBGLU8    : ------------------------------SYI-DA---------------FTRNDFP-----------EDFLFG :  35
AtBGLU9    : ------------------------------SYS-DA---------------FTRNSFP-----------KDFLFG :  35
AtBGLU10   : ------------------------------SDS-DA---------------FTRNNFP-----------KDFLFG :  35
AtBGLU11   : ------------------------------AVSSLK---------------YSRNDFP-----------PGFVFG :  38
Os11bglu36 : --------------------YARYRRRHLRRIPSPIDESADP--------LADFRAFPSSDADDSEEDNFFFGLA :  75
                                                                      p             f  g

                    160         *       180         *       200         *       220
Os10bglu34 : TASS-------------------------AYQY-----------EGAVKEDGR---GPTIWD------------- :  68
Os3bglu6   : TASA-------------------------AYQY-----------EGAVKEDGR---GQTIWD------------- :  80
AtBGLU40   : TASS-------------------------AFQH-----------EGAVKAEGR---GPTIWD------------- :  67
Os6bglu25  : TASS-------------------------AYQY-----------EGAVNEGQR---GPTIWD------------- :  55
AtBGLU41   : TASS-------------------------AYQF-----------EGAVKEGNK---GESIWD------------- :  65
Os1bglu4   : VATS-------------------------AYQI-----------EGARREGGK---GDNIWD------------- :  45
AtBGLU42   : VATS-------------------------AYQI-----------EGGWNEGKK---GPSIWD------------- :  51
Os1bglu1   : TAAS-------------------------AYQV-----------EGMALKDGR---GPSIWD------------- :  84
Os7bglu26  : TAAS-------------------------AYQV-----------EGMAKQGGR---GPSIWD------------- :  75
AtBGLU43   : TATS-------------------------AYQV-----------EGETHQDGR---GPSIWD------------- :  65
AtBGLU44   : TATS-------------------------AYQV-----------EGETHQDGR---GPSIWD------------- :  74
Os3bglu8   : TATS-------------------------AFQV-----------EGMAASGGR---GPSIWD------------- :  80
Os3bglu7   : TATS-------------------------AYQV-----------EGMAASGGR---GPSIWD------------- :  73
Os12bglu38 : TASS-------------------------AYQV-----------EGNALQYGR---GPCIWD------------- :  61
Os11bglu37 : TGSA-------------------------AYQY-----------EGAYKEGGK---GPSIWD------------- :  64
Os11bglu35 : TGSA-------------------------AYQY-----------EGAYKEGGK---GPSIWD------------- :  64
Os9bglu30  : TGSA-------------------------AYQY-----------EGAYKEGGK---GPSVWD------------- :  62
Os9bglu29  : TGSA-------------------------AYQY-----------EGAAKEGGR---GPSVWD------------- :  65
Os8bglu28  : TGTS-------------------------AYQY-----------EGAVDK--R---GQNIWD------------- :  59
Os8bglu27  : TGSA-------------------------AYQY-----------EGAVNEGGR---GPSIWD------------- :  59
Os4bglu10  : TSSS-------------------------SYQF-----------EGAAAKGGR---GPSIWD------------- :  69
Os4bglu11  : TSSS-------------------------SYQF-----------EGGAVLGGR---GPSIWD------------- :  70
Os4bglu9   : TSSA-------------------------SYQC-----------EGGAAEGGR---GSSIWD------------- :  69
Os4bglu13  : TASS-------------------------SYQY-----------EGGAREGGR---GPSIWD------------- :  65
Os4bglu12  : TASS-------------------------SYQY-----------EGGAAEGGR---GPSIWD------------- :  69
Os6bglu24  : TASS-------------------------AYQY-----------EGAVREGGR---GPSIWD------------- :  63
AtBGLU17   : AASS-------------------------AYQS-----------EGAANVDGR---EPSIWD------------- :  70
AtBGLU12   : AATS-------------------------AYQV-----------EGAAHEDGR---GPSIWD------------- :  66
AtBGLU13   : AATS-------------------------AYQV-----------EGAAHEDGR---GPSIWD------------- :  66
AtBGLU14   : AATS-------------------------AYQV-----------EGAAQEDGR---GPSIWD------------- :  65
AtBGLU15   : SATS-------------------------AYQV-----------EGGAHEDGR---GPSIWD------------- :  66
AtBGLU16   : -------------------------------QV-----------SG----------TPS---------------- :  35
AtBGLU18   : TATA-------------------------AFQV-----------EGAVNEGCR---GPSMWD------------- :  74
AtBGLU19   : TATA-------------------------AFQV-----------EGAVNEGCR---GPSLWD------------- :  68
AtBGLU20   : TATA-------------------------AFQV-----------EGAVNEGCR---GPSMWD------------- :  72
AtBGLU21   : TATA-------------------------AFQV-----------EGAINETCR---GPALWD------------- :  71
AtBGLU22   : TATA-------------------------AFQV-----------EGAINETCR---GPALWD------------- :  71
AtBGLU23   : TATA-------------------------AYQV-----------EGAINETCR---GPALWD------------- :  70
AtBGLU24   : TATA-------------------------AYQV-----------EGAVNETCR---GPSVWD------------- :  74
AtBGLU25   : ATTS-------------------------AFQH-----------EGAAEEGGR---GSSIWD------------- :  69
AtBGLU26   : TASS-------------------------SYQY-----------EGAVNEGAR---GQSVWD------------- :  49
AtBGLU27   : TASS-------------------------AYQY-----------EGARNEAPR---GESVWD------------- :  43
AtBGLU28   : TAAS-------------------------AFQY-----------EGATSEGGK---SPSIWD------------- :  64
AtBGLU29   : TAIS-------------------------AFQS-----------EGATSEGGK---SPTIWD------------- :  64
AtBGLU30   : TAAS-------------------------AFQY-----------EGATSEGGK---SPTIWD------------- :  61
AtBGLU31   : VASS-------------------------AYQY-----------EGAVEEGGR---SLSIWD------------- :  67
AtBGLU32   : VASS-------------------------AYQY-----------EGAVEEGGR---SPSIWD------------- :  67
AtBGLU33   : TSVS-------------------------AYQV-----------EGAKKGSGR---GLTSWD------------- : 129
AtBGLU34   : AATS-------------------------AYQI-----------EGAAH---R---ALNGWD------------- :  77
AtBGLU35   : AATS-------------------------AYQI-----------EGAAH---R---ALNGWD------------- :  77
AtBGLU36   : AATS-------------------------AYQV-----------EGAAH---R---ALNGWD------------- :  52
AtBGLU37   : VASS-------------------------AYQI-----------EGGRG---R---GLNVWD------------- :  82
AtBGLU38   : VASS-------------------------AYQV-----------EGGRG---R---GLNVWD------------- :  70
AtBGLU39   : --------------------------------------------EGGKG---R---GLNVWD------------- :  61
Os4bglu18  : TATS-------------------------SYQI-----------EGAYLEGNK---SLSNWD------------- :  62
Os4bglu14  : TSSS-------------------------AYQV-----------EGGYLEGNK---GLSNWD------------- :  59
Os4bglu16  : AATS-------------------------AYQI-----------EGAYLDDNK---GLNNWD------------- :  63
AtBGLU45   : TASS-------------------------AYQY-----------EGAFLTDGK---SLNNWD------------- :  68
AtBGLU46   : TASS-------------------------AFQY-----------EGAFLTDGK---GLNNWD------------- :  65
AtBGLU47   : TASS-------------------------AYQY-----------EGAYLTDGK---TLSNWD------------- :  89
Os9bglu33  : AGSS-------------------------AFQV-----------EGAAAEDGR---KPSIWD------------- :  66
Os9bglu32  : AGTS-------------------------AFQV-----------EGAAAEDGR---KPSIWD------------- :  66
Os9bglu31  : AGSS-------------------------AYQV-----------EGAFAEDGR---KPSIWD------------- :  65
Os1bglu2   : AATS-------------------------AYQY-----------DGAAAEDGR---SPTIWD------------- :  98
Os1bglu3   : AATS-------------------------AYQY-----------EGAAAEDGR---GASIWD------------- :  59
Os1bglu5   : SATS-------------------------SYQY-----------EGGFDEDGR---SPSNWD------------- :  62
Os5bglu22  : AGTS-------------------------AYQY-----------EGATDEDGR---SPSIWD------------- :  77
Os5bglu23  : AGTS-------------------------AYQY-----------EGATGEDGR---TPSIWD------------- :  68
Os5bglu20  : AGTA-------------------------AYQY-----------EGAAAEDGR---TPSIWD------------- :  68
Os5bglu21  : AGTS-------------------------AYQY-----------EGGAAEDGR---TPSIWD------------- :  72
Os5bglu19  : AGTA-------------------------AFQY-----------EGAAAEDGR---TPSIWD------------- :  69
AtBGLU1    : AGIS-------------------------AYQW-----------EGAVDEDGR---KPSVWD------------- :  64
AtBGLU3    : SATS-------------------------AYQW-----------EGAFDEDGR---KPSVWD------------- :  57
AtBGLU4    : AGTS-------------------------AYQW-----------EGAAAEDGR---KPSLWD------------- :  59
AtBGLU5    : AGTS-------------------------AYQW-----------EGAAAEDGR---KPSVWD------------- :  59
AtBGLU6    : SSTS-------------------------AYQW-----------EGAVAEDGR---KPSVWD------------- :  59
AtBGLU7    : AATS-------------------------AYQW-----------EGAFDEDGK---SPSVWD------------- :  58
AtBGLU8    : AGTS-------------------------AYQW-----------EGAANEDGR---TPSVWD------------- :  58
AtBGLU9    : AATS-------------------------AYQW-----------EGAVAEDGR---TPSVWD------------- :  58
AtBGLU10   : AATS-------------------------AYQW-----------EGAVAEDGR---TPSVWD------------- :  58
AtBGLU11   : SGTS-------------------------AYQV-----------EGAADEDGR---TPSIWD------------- :  61
Os11bglu36 : TAPAHVEDRLEDAWLQFATETSCDDNGNVRDQRPVDALMASAAGDGGSQQSWRSTGGENIGDREQRKPLRVAMEA : 150
                                          a q            eG               d

                 *       240         *       260         *       280         *       300
Os10bglu34 : ---KFAH-TFGK-IIDFSNADVAVDQYH---------RF----EEDIQLMADMGMDAYRFSIAWSRIFPNG---T : 122
Os3bglu6   : ---TFAH-TFGK-ITDFSNADVAVDQYH---------RF----EEDIQLMADMGMDAYRFSIAWSRIYPNG---V : 134
AtBGLU40   : ---TFSH-TFGK-ITDFSNADVAVDQYH---------RY----EEDVQLMKNMGMDAYRFSISWTRIFPNG---V : 121
Os6bglu25  : ---TLTK-RPGR-VIDFSNADVAVDHYH---------RY----KEDVELMNDIGMDAYRFSISWSRIFPNG---T : 109
AtBGLU41   : ---TFTKEKPGK-ILDFSNADTTVDQYH---------RF----HNDIDLMKDLRMDAYRFSISWSRIFPNG---T : 120
Os1bglu4   : ---VFTE-NKER-ILDGSSGEVAVDHYH---------RY----KEDIELMASLGFRAYRFSISWPRIFPDG---L :  99
AtBGLU42   : ---KFTH-IEGK-ILDGSNGDVAVDHYH---------RY----KEDVDLIGQLGFGAYRFSISWSRIFPDG---L : 105
Os1bglu1   : ---AFVK-TPGE-IANNATADVTVDEYH---------RY----KEDVNIMKSMGFDAYRFSISWSRIFPTG---T : 138
Os7bglu26  : ---AFIE-KPGT-IPNNATADVTVDEYH---------RY----KEDVNIMKNMGFDAYRFSISWSRIFPNG---T : 129
AtBGLU43   : ---AFVK-IPGK-IANNATAEITVDQYH---------RY----KEDVDLMQNLNIDAYRFSISWSRIFPEG---S : 119
AtBGLU44   : ---AFVK-IPGK-IAKNATAEITVDQYH---------RY----KEDVDLMKKLNFDAYRFSISWSRIFPEG---S : 128
Os3bglu8   : ---PFVH-TPGN-IAGNGNADVTTDEYH---------RY----KEDVDLLKSLNFDAYRFSISWSRIFPDG---E : 134
Os3bglu7   : ---AFAH-TPGN-VAGNQNGDVATDQYH---------RY----KEDVNLMKSLNFDAYRFSISWSRIFPDG---E : 127
Os12bglu38 : ---TFLM-QPGV-TPDNSTANVTVDEYH---------RY----MDDVDNMVRVGFDAYRFSISWSRIFPSG---L : 115
Os11bglu37 : ---TFTH-VPGK-ILNNDTGDVASDFYH---------RY----KEDVNLLKDMNMDAFRFSIAWTRILPNG---S : 118
Os11bglu35 : ---TFTH-IPGK-ILNNDTGDVANDFHP---------DT----REDVNLLKDMNMDAFRFSIAWTRILPNG---S : 118
Os9bglu30  : ---NFTH-IPGK-ILNNDNGDVANDFYH---------RY----KEDVSLLKDMNMDAFRFSIAWTRILPNG---S : 116
Os9bglu29  : ---TFSH-IPGK-ILNGDTGDVADDFYH---------RY----KEDVNLLKDMNMDAFRFSISWSRILPNG---T : 119
Os8bglu28  : ---TFSR-IPGK-IADGSNADIANDFYH---------RY----KEDLNLITAMNMDSFRFSIAWSRILPNG---T : 113
Os8bglu27  : ---TYAH-IPGK-VEDGSNGDVAVDFYH---------RY----KEDLNFVTDMNMDAFRFSIAWSRILPNG---T : 113
Os4bglu10  : ---TFTHQYPDK-ITDKSNGDGACNSYH---------LY----KEDVRIMKEMGMDAYRFSISWSRILPNG---S : 124
Os4bglu11  : ---TFTHQSPDK-ITDRSNGDVACDSYH---------LY----KEDVRSMKEMGMDAYRFSISWSRILPS----A : 124
Os4bglu9   : ---TFTYQHPDK-IADKSNGDVADNTYH---------LY----KEDVHMMKEMGMDAYRFSISWSRILPNG---S : 124
Os4bglu13  : ---TFTHQHPDK-IADKSNGDVAADSYH---------LY----KEDVRIMKDMGVDAYRFSISWTRILPNG---S : 120
Os4bglu12  : ---TFTHQHPEK-IADRSNGDVASDSYH---------LY----KEDVRLMKDMGMDAYRFSISWTRILPNG---S : 124
Os6bglu24  : ---TFTHNHPEK-IANGSNGDIAIDSYH---------RY----KEDVGIMKGLGLNAYRFSVSWPRILPNG---K : 118
AtBGLU17   : ---TFTKQYPEK-ISDGSNGDVADEFYY---------RF----KEDVAHMKEIGLDSFRFSISWSRILPRG---T : 125
AtBGLU12   : ---TFSEKYPEK-IKDGSNGSIASDSYH---------LY----KEDVGLLHQIGFDAYRFSISWSRILPRE---N : 121
AtBGLU13   : ---TFSEKYPEK-IKDGTNGSIASDSYH---------LY----KEDVGLLHQIGFGAYRFSISWSRILPRG---N : 121
AtBGLU14   : ---TFSEKYPEK-IKDGSNGSIADDSYH---------LY----KEDVGLLHQIGFNAYRFSISWSRILPRG---N : 120
AtBGLU15   : ---TFSEKYPEK-IKDGSNGSVADNSYH---------LY----KEDVALLHQIGFNAYRFSISWSRILPRG---N : 121
AtBGLU16   : -------------LK---NS-----------------------QEDVNLLHQIGFDAYRFSISWSRILPRG---T :  68
AtBGLU18   : ---TFTKKFP-H-RCENHNADVAVDFYH---------RY----KEDIQLMKDLNTDAFRLSIAWPRIFPHG---R : 128
AtBGLU19   : ---IYTKKFP-H-RVKNHNADEAVDFYH---------RY----KEDIQLMKKLNTDGFRLSISWPRIFPHG---R : 122
AtBGLU20   : ---VYTKKFP-H-KCNYHNADVAVDFYH---------RY----KEDIKLMKNLNTDGFRFSIAWPRIFPHG---R : 126
AtBGLU21   : ---IYCRRNP-E-RCSGDHADVAVDFFH---------RY----KEDIQLMKNLNTDAFRLSIAWSRIFPHG---R : 125
AtBGLU22   : ---IFCKRNP-E-RCSGHNADVAVDFFH---------RY----KEDIQLMKNLNTDAFRLSIAWSRIFPHG---R : 125
AtBGLU23   : ---IYCRRYP-E-RCNNDNGDVAVDFFH---------RY----KEDIQLMKNLNTDAFRMSIAWPRIFPHG---R : 124
AtBGLU24   : ---IYCKKYP-E-KCNGDNGTQAVDFFY---------RY----KEDIQLMKNLNTDSFRLSISWTRIFPHG---R : 128
AtBGLU25   : ---SFTLKQHSE-SNNNLDGRLGVDFYH---------HY----KEDVQLLKKLNMDAFRFSISWSRIFPHG---K : 124
AtBGLU26   : ---HFSNRFPHR-ISDSSDGNVAVDFYH---------RY----KEDIKRMKDINMDSFRLSIAWPRVLPYG---K : 104
AtBGLU27   : ---TFVRKYPER-NCYSN-ADQAIEFYN---------HY----KDDIQRMKDINMDAFRFSISWPRIFPLG---K :  97
AtBGLU28   : ---YFSHTFP-E-RTRMQNADVAVDFYH---------RY----KDDIKLMKELNMDAFRFSISWARLIPSG---K : 118
AtBGLU29   : ---YFSHTFP-E-RTNMQNADVAVDFYH---------RY----KDDIKLIEELNVDAFRFSISWARLIPSG---K : 118
AtBGLU30   : ---HFSLTYP-E-RTKMHNADVAIDFYH---------RY----KDDIKLMKELNMDAFRFSISWSRLIPSG---K : 115
AtBGLU31   : ---NFTHAFP-E-RTNMDNGDVAVDFYH---------RY----KEDIKLIKEMNMDSFRFSLSWSRILPSG---K : 121
AtBGLU32   : ---NFTHAFP-E-RTNMDNGDVAVDFYH---------RY----KDDIKLIKEMNMDSFRFSLSWSRILPSG---K : 121
AtBGLU33   : ---EFTHMFPEK-VQQNGDGDEGVDFYT---------RY----KDDIKLMKELNTNGFRFSISWTRILPYG---T : 184
AtBGLU34   : ---YFTHRYPEK-VPDRSSGDLACDSYD---------LY----KDDVKLLKRMNVQAYRLSIAWSRVLPKG---R : 132
AtBGLU35   : ---YFTHRYPEK-VPDRSSADLACDSYD---------LY----KDDVKLLKRMNVQAYRLSIAWSRVLPKG---R : 132
AtBGLU36   : ---YFTHRYPER-VSDRSIGDLACNSYD---------LY----KDDVKLLKRMNVQAYRFSIAWSRVLPKG---R : 107
AtBGLU37   : ---GFTHRYPEKGGADLGNGDTTCDSYR---------TW----QKDLDVMEELGVKGYRFSFAWSRILPKG---K : 138
AtBGLU38   : ---SFTHRFPEKGGADLGNGDTTCDSYT---------LW----QKDIDVMDELNSTGYRFSIAWSRLLPKG---K : 126
AtBGLU39   : ---GFTHRYPEKGGPDLGNGDSTCGSYE---------HW----QKDIDVMTELGVDGYRFSLAWSRIAPR----- : 115
Os4bglu18  : ---VFTHLPG--NIKDGSNGDIADDHYH---------RY----EEDVELMNSLGVNAYRFSISWSRILPKG---R : 116
Os4bglu14  : ---VFTHKQG--TIEDGSNGDTANDHYH---------RY----MEDIELMHSLGVNSYRFSISWARILPKG---R : 113
Os4bglu16  : ---VFTHTQAG-RISDGRNGDVADDHYH---------RY----TEDVDILHNLGVNSYRFSISWARILPRG---R : 118
AtBGLU45   : ---VFTHKNPG-KILDKNNADRAVDQYN---------RF----LEDIQLMSFLGVNSYRFSISWCRILPRG---R : 123
AtBGLU46   : ---VFAHENPG-KIVDGSNGDIATDQYH---------RY----MEDIQSMNFLGVNSYRLSISWSRVLPNG---R : 120
AtBGLU47   : ---VFTN-ISG-KIADGSHGKVAVDHYH---------RY----PGDLDLMEDLGVNSYRLSLSWARILPKG---R : 143
Os9bglu33  : ---TFINQG---YMPDGSNADVSADQYH---------HY----KEDVKLMYDMGLDAYRFSIAWPRLIPDG---R : 119
Os9bglu32  : ---TFTHQG---YSPGGAIADVSADQYH---------LY----KEDVKLMYDMGLDAYRFSIAWPRLIPDG---R : 119
Os9bglu31  : ---TFSHSG---YSVDGATGDVTADQYH---------KY----KEDVKLLQDMGVDAYRMSISWSRLIPDG---R : 118
Os1bglu2   : ---TFAHEGQ--KTKDKGTGDVAADGYH---------KY----KGDVKLMAETGLEAYKFSISWSRLIPNG---R : 152
Os1bglu3   : ---TFTHAG---KMKDKSTGDVASDGYH---------KY----KGDVKLMTETGLEAYRFSISWSRLIPSG---R : 112
Os1bglu5   : ---IFTHQG---KMPGRSTADVAADGYH---------KY----KDDLKLMVDTNLEAYRLSISWSRIIPNG---R : 115
Os5bglu22  : ---TFTHAG---KMPDKSTGDMGAGGYH---------KY----KEDVKLMSDTSLEAYRFSISWSRLIPRG---R : 130
Os5bglu23  : ---TFTHSG---RMADNSTGDRAAAGYH---------KY----KEDVKLMSDTGLEAYRFSISWSRLIPRG---R : 121
Os5bglu20  : ---TYTHSG---RHPEDGTGDVASDGYH---------KY----KEDVKLMTEIGLEAYRFTISWSRLIPSG---R : 121
Os5bglu21  : ---TYTHSG---RHPEDETGDVASDGYH---------KY----KEDVKLMSEIGLEAYRFTISWSRLIPSG---R : 125
Os5bglu19  : ---TYAHSW---RNPGGETGDVACDGYH---------KY----KEDVMLMNETGLEAYRFTISWSRLIPSG---R : 122
AtBGLU1    : ---TFLHCR---K-MDN--GDIACDGYH---------KY----KEDVQLMAETGLHTFRFSISWSRLISNG---R : 114
AtBGLU3    : ---TFLHTR---N-LSN--GDITSDGYH---------KY----KEDVKLMVETGLDAFRFSISWSRLIPNG---R : 107
AtBGLU4    : ---TLCHSR---D-QGN--GDIACDGYH---------KY----KDDVKLMVDTNLDAFRFSISWSRLIPNG---R : 109
AtBGLU5    : ---TLCYSR---N-IGN--GDVTCDGYH---------KY----KEDVKLMVDTNLDAFRFSISWSRLIPNG---R : 109
AtBGLU6    : ---RFCHSH---NNQGN--GDITCDGYH---------KY----KEDVKLMVDTNLDAFRFSISWSRLIPNR---R : 110
AtBGLU7    : ---TTSHCD---SGSNN--GDIACDGYH---------KY----KEDVMLMAEMGLESFRFSISWSRLIPNG---R : 109
AtBGLU8    : ---TTSHCY---NGSN---GDIACDGYH---------KY----KEDVKLMAEMGLESFRFSISWSRLIPNG---R : 108
AtBGLU9    : ---TFSNSY---DTG---NGDVTSDGYH---------KY----KEDVKLMATMGLESFRFSISWSRLIPNG---R : 108
AtBGLU10   : ---TFSHTY---NRGNLGNGDITSDGYH---------KY----KEDVKLMAEMGLESFRFSISWSRLIPNG---R : 111
AtBGLU11   : ---VFAHAG---H-SGVAAGNVACDQYH---------KY----KEDVKLMADMGLEAYRFSISWSRLLPSG---R : 113
Os11bglu36 : MLRGFEILAESGESAGGDNCSHNVAAWHNVPCPQERLRFWSDPDAELKLAKETGISVFRMGVDWARLMPEEPTEE : 225
                                        h                 d           54f   W R6 p g

                      *       320         *       340         *       360         *
Os10bglu34 : G--EVNQAGIDHYNKLINALLAKG--------IEPYVTLYHWDLPQALEDKYTGWLDRQIINDYAVYAETCFQAF : 187
Os3bglu6   : G--QVNQAGIDHYNKLIDALLAKG--------IQPYVTLYHWDLPQALEDKYKGWLDRQIVDDFAAYAETCFREF : 199
AtBGLU40   : G--HINEAGIDHYNKLINALLAKG--------IEPYVTLYHWDLPQALHDRYLGWLNPQIINDFAAYAEVCFQRF : 186
Os6bglu25  : G--EPNEEGLSYYNSLIDALLDKG--------IEPYVTLFHWDLPQALEDRYGGWLNSEIIEDFVQYAFTCFKEF : 174
AtBGLU41   : G--EVNPDGVKYYNSLIDALLAKG--------IKPYVTLYHWDLPQALEDRYEGWLSREVVDDFEHYAFTCFKAF : 185
Os1bglu4   : GK-NVNEQGVAFYNDLINFMIEKG--------IEPYATLYHWDLPHNLQQTVGGWLSDKIVEYFALYAEACFANF : 165
AtBGLU42   : GT-EVNEEGIAFYNDLINTLLEKG--------IQPYVTLYHWDLPSHLQEAIGGWTNRKIVDYFGLYADACFANF : 171
Os1bglu1   : G--KVNWKGVAYYNRLINYMLKIG--------ITPYANLYHYDLPEALEVQYGGLLNRKIVEAFADYAEFCFKTF : 203
Os7bglu26  : G--MVNQEGVDYYNRLIDYMVKKG--------IKPYANLYHYDLPLALHEQYLGWLSPNIVEAFADYADFCFQTF : 194
AtBGLU43   : G--KINSNGVAYYNRLIDYLIEKG--------ITPYANLYHYDLPLALEQKYQGLLSKQ--GRFCGLRRVLFQTF : 182
AtBGLU44   : G--KVNWKGVAYYNRLIDYMVQKG--------ITPYANLYHYDLPLALENKYKGLLGRQVVKDFADYAEFCYKTF : 193
Os3bglu8   : G--KVNTEGVAYYNNLIDYVIKQG--------LIPYVNLNHYDLPLALQKKYEGWLSPKIVGVFSDYAEFCFKTY : 199
Os3bglu7   : G--RVNQEGVDYYNNLINYLLQKG--------ITPYVNLYHYDLPLALEKKYGGWLNAKMADLFTEYADFCFKTF : 192
Os12bglu38 : G--KINKDGVDYYHRLIDYMLANN--------IIPYVVLYHYDLPQVLHDQYKGWLHPRIVRDFVRFADFCFKTY : 180
Os11bglu37 : LSGGINREGVAFYNSLINDVIAKG--------MIPFVTIFHWDTPQALESKYGGFLSENIVKDYVDFAEVCFREF : 185
Os11bglu35 : LSGGINREGVAFYNSLINDVIAKG--------MIPFVTIFHWDTPQALESKYGGFLSENIVKDYADFAEVCFHEF : 185
Os9bglu30  : LSGGINKEGVAFYNSLINDVIAKG--------MIPFVTIFHWDTPLALESKYGGFLSEDIVKEYVDFAEVCFREF : 183
Os9bglu29  : LSGGVNKEGVAFYNNLINEIIAKG--------MKPFVTIFHWDTPQALESKYGGFLSENIIKDYVDFAEVCFREF : 186
Os8bglu28  : ISGGINKEGVEFYNSLINEVIAKG--------LKPFVTIFHFDTPQALEDKYGGFLSENIVKDYVDYADLCFSLF : 180
Os8bglu27  : ISGGINKEGIAFYNSLINEVISRG--------LKPFVTIFHFDTPQALEDKYRSFLSENIVKDFVDYADVCFREF : 180
Os4bglu10  : LSGGVNREGINYYNNLINELLSKE--------VQPFATLFHFDTPQALEDKYKGFLSPNIINDYKDYAEICFKEF : 191
Os4bglu11  : LSGGVNREGISYYNNLINELLSKG--------VQPFVTLFHWDSPQALEDKYKGFLSPNIINDYKEYAETCFKEF : 191
Os4bglu9   : LNGGVNIEGINYYNNLINELLLKG--------VQSFVTLFHYDTPQALEDKYNGFLSPNIINDYKDYAEICFKEF : 191
Os4bglu13  : LSGGINREGISYYNNLINELLLKG--------VQPFVTLFHWDSPQALEDKYNGFLSPNIINDYKEYAETCFKEF : 187
Os4bglu12  : LRGGVNKEGIKYYNNLINELLSKG--------VQPFITLFHWDSPQALEDKYNGFLSPNIINDFKDYAEICFKEF : 191
Os6bglu24  : LSGGVNLEGIKYYNNLIDELISKG--------VEPFVTLFHWDSPQALEQQYGGFLSNLIVEDFRDYADICFREF : 185
AtBGLU17   : VAGGVNQAGINFYNHLINELISNG--------IRPLVTLFHWDTPQALEDEYGGFLNPQIVKDFVEYVDICFKEF : 192
AtBGLU12   : LKGGINQAGIDYYNNLINELLSKG--------IKPFATIFHWDTPQSLEDAYGGFLGAEIVNDFRDYADICFKNF : 188
AtBGLU13   : LKGGINQAGIDYYNNLINELLSKG--------IKPFATIFHWDTPQSLEDAYGGFFGAEIVNDFRDYADICFKNF : 188
AtBGLU14   : LKGGINQAGIDYYNNLINELLSKG--------IKPFATIFHWDTPQDLEDAYGGFRGAEIVNDFRDYADICFKSF : 187
AtBGLU15   : LKGGINQAGIDYYNNLINELLSKG--------IKPFATMFHWDTPQALEDAYGGFRGAEIVNDFRDYADICFKNF : 188
AtBGLU16   : LKGGINQAGIEYYNNLINQLISKG--------VKPFVTLFHWDLPDALENAYGGLLGDEFVNDFRDYAELCFQKF : 135
AtBGLU18   : MSKGINKVGVQFYHDLIDELLKNN--------IIPLVTVFHWDTPQDLEDEYGGFLSGRIVQDFTEYANFTFHEY : 195
AtBGLU19   : MEKGISKEGVQFYHDLIDELLKND--------ITPLVTVFHWDTPADLEDEYGGFLSERIVPDFVEYANFTFHEY : 189
AtBGLU20   : MEKGISKAGVQYYHDLIDELLANG--------ITPLVTVFHWDTPQDLEDEYGGFLSDRIIKDFTEYANFTFQEY : 193
AtBGLU21   : KEKGVSQAGVQFYHELIDELLKN----------VPFVTVFHWDTPQDLEDEYGGFLSQNIVKDFREYADYVFTEY : 190
AtBGLU22   : KEKGVSQAGVKFYHDLIDELLKNG--------IIPFVTVFHWDTPQDLEDEYGGFLSENIVKDFREYADYVFTEY : 192
AtBGLU23   : KEKGVSQAGVQFYHDLIDELIKNG--------ITPFVTVFHWDTPQDLEDEYGGFLSERIVKDFREYADFVFQEY : 191
AtBGLU24   : EENGVSKSGVQFYHDLIDELKRNG--------IIPFVTVFHWDTPQTLENEYGGFLSAHIVKDFREYAEFVFKEY : 195
AtBGLU25   : KDKGVSETGVKFYNDLINELIANG--------VTPLVTLFQWDVPQALEDEYGGFLSDRILEDFRDFAQFAFNKY : 191
AtBGLU26   : RDRGVSEEGIKFYNDVIDELLANE--------ITPLVTIFHWDIPQDLEDEYGGFLSEQIIDDFRDYASLCFERF : 171
AtBGLU27   : KSKGVNKEGIQFYNDLIDELLANG--------ITPLATLFHWDTPQALEDEYSGFLSEEAVDDFKDFAALCFEEF : 164
AtBGLU28   : VKDGVNKEGVEFYKALIDELVANG--------IEPSMTLYHWDHPQSLEDEYGGFLSPQIVEDFRDFSRVCFEEF : 185
AtBGLU29   : VKDGVNKEGVQFYKALIDELIANG--------IQPSVTLYHWDHPQALEDEYGGFLNPQIIEDFRNFARVCFENF : 185
AtBGLU30   : LKDGVNKEGVQFYKDLIDELLAND--------IQPSMTLYHWDHPQSLEDEYGGFLSPKIVEDFRDFARICFEEF : 182
AtBGLU31   : LSDGVNKEGVQFYKNLIDELIENG--------IKPFVTIYHWDIPQALDDEYGSFLSPRIIDDFRNYARFCFQEF : 188
AtBGLU32   : LSDGVNKEGVQFYKNLIDELIKNG--------IKPFVTIYHWDIPQALDDEYGSFLSPRIIDDFRNFARFCFQEF : 188
AtBGLU33   : IKKGVNEEGVKFYNDLINELLANG--------IQPSVTLFHWESPLALEMEYGGFLNERIVEDFREFANFCFKEF : 251
AtBGLU34   : LTGGVDENGITYYNNLINELKANG--------IEPYVTIFHWDVPQTLEDEYGGFLSTRIVEDYTNYAELLFQRF : 199
AtBGLU35   : LTGGVDENGITYYNNLINELKANG--------IEPYVTIFHWDVPQTLEDEYGGFLSTRIVEDYTNYAELLFQRF : 199
AtBGLU36   : LIGGVDENGITYYNNLINELKANG--------IEPFVTIFHWDVPQDFRRRIWRLLKP-TYSDFKNYAELLFQRF : 173
AtBGLU37   : RSRGINEDGINYYSGLIDGLIARN--------ITPFVTLFHWDLPQSLQDEYEGFLDRTIIDDFKDYADLCFERF : 205
AtBGLU38   : RSRGVNPGAIKYYNGLIDGLVAKN--------MTPFVTLFHWDLPQTLQDEYNGFLNKTIVDDFKDYADLCFELF : 193
AtBGLU39   : ES---NQAGVKYYNDLIDGLLAKN--------ITPFVTLFHWDLPQVLQDEYEGFLNHEIIDDFKDYANLCFKIF : 179
Os4bglu18  : FG-GVNPAGIDFYNKLIDSILLKG--------IQPFVTLTHYDIPQELEDRYGAWLNAEIQSDFGHFADVCFGAF : 182
Os4bglu14  : FG-DVNPDGVAFYNALIDGLVQKG--------IQPFVTICHYDIPHELDERYGGWLSPEIQKDFSYFAEVCFKLF : 179
Os4bglu16  : LG-GVNSAGIAFYNRLINALLQKG--------IQPFVTLNHFDIPHELETRYGGWLGAAIREEFEYYSDVCFNAF : 184
AtBGLU45   : FG-EINYLGIKYYNIFIDALISRG--------IKPFVTLNHVDYPQELEDRFQSWLNPEMQKEFGYLADICFKHF : 189
AtBGLU46   : FG-VINYKGIKYYNNLIDALIKKG--------ITPFVTLNHFDYPQELENRFKSWLSSEMQKDFGYLADICFKHF : 186
AtBGLU47   : FG-DVNMGGIDHYNRMINDILKTG--------IEPFVTLTHYDIPQELEYRYGSWLNPQIREDFEHYANICFRHF : 209
Os9bglu33  : G--EINPKGLEYYNNLIDELIMHG--------IQPHVTIYHFDLPQALQDEYGGILSPRFIEDYSAYAEVCFKNF : 184
Os9bglu32  : G--EINPKGLEYYNNLIDELIMHG--------IQPHVTIYHFDLPQALQDEYGGILSPRFIEDYTAYAEVCFKNF : 184
Os9bglu31  : G--AVNPKGLEYYNNLIDELLSHG--------IQPHVTIYHFDFPQALQDEYNGILSPRFVEDFTAYADVCFKNF : 183
Os1bglu2   : G--AVNQEGLKYYNNVIDELAKRG--------IQPHIMLCHLDLPQALEDEYDGWLSPRIVDDFTAYADVCFREF : 217
Os1bglu3   : G--AVNQQGLKYYNNIIDELTKRG--------IQVHVMLYHLDLPQALEDEYAGWLSPRIVEDFTAYADVCFREF : 177
Os1bglu5   : G--DVNPKGLQYYNDIIDGLVKNG--------IQVHIMLYQLDLPQVLEDEYDGWLSPRILEDFKAYADVCFKEF : 180
Os5bglu22  : G--PVNPKGLEYYNSLIDELVERG--------IEIHVTLYHLDFPQILEDEYHGWLSPRVIDDFTAYADVCFREF : 195
Os5bglu23  : G--PINPKGLEYYNDLIDKLVKRGEICDCSMGIEIHVTLYHLDFPQALQDEYNGWLSPRIIEDFTAYADVCFREF : 194
Os5bglu20  : G--AVNPKGLQFYNNMINELVKAG--------IQIQVVLYHSDLPQSLQDEYGGWINPKIVDDFTAYADVCFREF : 186
Os5bglu21  : G--AVNLKALQFYNSMINELVKAG--------IQIHVVMYHMDLPQSLQDEYGGWISPKIVDDFTAYADVCFREF : 190
Os5bglu19  : G--AVNPKGLQFYNSMINELVKAG--------IQIHAVLYHIDLPQSLQDEYGGWVSPKVVDDFAAYADVCFREF : 187
AtBGLU1    : G--SINPKGLQFYKNFIQELVKHG--------IEPHVTLHHYDFPQYLEDDYGGWTNRKIIKDFTAYADVCFREF : 179
AtBGLU3    : G--PVNPKGLQFYKNFIQELVSHG--------IEPHVTLFHYDHPQYLEDEYGGWINRRIIQDFTAYANVCFREF : 172
AtBGLU4    : G--PVNQKGLQFYKNLIQELVSHG--------IEPHVTLYHYDHPQSLEDEYGGWLNHRMIKDFTTYADVCFREF : 174
AtBGLU5    : G--SVNQKGLQFYKNLISELITHG--------IEPHVTLYHYDHPQYLEDEYGGWVNNMMIKDFTAYVDVCFREF : 174
AtBGLU6    : G--PVNQKGLQFYKNLIQELVNHG--------IEPYVTLHHFDHPQYLEDEYEGWLNHMIVEDFTAYADVCFREF : 175
AtBGLU7    : G--RINPKGLLFYKNLIKELRSHG--------IEPQVTLYHYDLPQSLEDEYGGWINRKIIEDFTAFADVCFREF : 174
AtBGLU8    : G--RINPKGLLFYKNLIKELRSHG--------IEPHVTLYHYDLPQSLEDEYGGWINHKIIEDFTAFADVCFREF : 173
AtBGLU9    : G--LINPKGLLFYNNLIKDLKSHG--------IEPHVTLYHYDLPQSLEDEYGGWINRKIIEDFTAYADVCFREF : 173
AtBGLU10   : G--LINPKGLLFYKNLIKELISHG--------IEPHVTLYHYDLPQSLEDEYGGWINRKIIEDFTAYADVCFREF : 176
AtBGLU11   : G--PINPKGLQYYNNLIDELITHG--------IQPHVTLHHFDLPQALEDEYGGWLSQEIVRDFTAYADTCFKEF : 178
Os11bglu36 : LKSSVNFAALERYRWIIQRVREYG--------MKVMLTLFHHSLP-PWAGKYGGWKMEKTVTYFMDFVRLVVDCV : 291
                     g6  Y   I  6   g          p  t6 h d P  l     g        d5   a  c

               380         *       400         *       420         *       440         *
Os10bglu34 : GDRVKHWITFNEPHTVAVQAYDSG-MHAPGRCSVLLH------LYCKK--GNSGTEPYIVAHNMILSHATVSDIY : 253
Os3bglu6   : GDRVKHWITLNEPHTVAIQGYDAG-LQAPGRCSVLLH------LYCKA--GNSGTEPYVVAHHFILAHAAAASIY : 265
AtBGLU40   : GDRVKHWITFNEPHTFAIQGYDVG-LQAPGRCTILFK------LTCRE--GNSSTEPYIVGHNVILTHATVSDIY : 252
Os6bglu25  : GDRVKHWITFNEPYNFAIDGYDLG-IQAPGRCSILSH------VFCRE--GKSSTEPYIVAHNILLAHAGAFRAY : 240
AtBGLU41   : GDRVKYWITFNEPHGVSIQGYDTG-IQAPGRCSLLGH------WFCKK--GKSSVEPYIVAHNILLSHAAAYHTY : 251
Os1bglu4   : GDRVKHWITINEPLQTAVNGYGIG-HFAPGGC------------------EGETARCYLAAHYQILAHAAAVDVY : 221
AtBGLU42   : GDRVKHWITLNEPLQTSVNGHCIG-IFAPGRN------------------EKPLIEPYLVSHHQVLAHATAVSIY : 227
Os1bglu1   : GDRVKNWMTFNEPRVVAALGYDDG-NFAPGRCT----------KCTAG---NSATEPYIVAHHLILSHASAVQRY : 264
Os7bglu26  : GDRVKDWFTFNEPRCVAALGYDNG-FHAPGRCS----------GCDAG--GNSTTEPYLAAHHLILSHAAAVKRY : 256
AtBGLU43   : GDRVKNWMTFNEPRVVAALGYDNG-IFAPGRCSEAFG------NCTDG---NSATEPYIVAHHLILAHAAAVQRY : 247
AtBGLU44   : GDRVKNWMTFNEPRVVAALGYDNG-IFAPGRCSKAFG------NCTEG---NSATEPYIVTHHLILAHAAAVQRY : 258
Os3bglu8   : GDRVKNWFTFNEPRIVAALGHDTG-TDPPNRCT----------KCAAG--GNSATEPYIVAHNIILSHATAVDRY : 261
Os3bglu7   : GNRVKHWFTFNEPRIVALLGYDQG-TNPPKRCT----------KCAAG--GNSATEPYIVAHNFLLSHAAAVARY : 254
Os12bglu38 : GHKVKNWFTINEPRMMANHGYGDG-FFPPGRCT----------GCQPG--GNSATEPYIAAHNLLLSHAAAVRTY : 242
Os11bglu37 : GDRVKYWTTFNEPFTYNAYGYGKG-VFAPGRCSPYVS-----KSCGAG---DSSREPYLVTHHIHLSHAAADLPY : 251
Os11bglu35 : GDRVKYWTTFNEPFTYSAYGYGGG-VFASGRCAPYVS-----KSCGAG---DSSREPYLVTHHIHLSHAAVVHLY : 251
Os9bglu30  : GDRVKYWFTFNEPFTYSAYGYGKG-VFAPGRCSSYVS-----KSCGVG---DSSREPYLVAHHIHLSHAAAVQLY : 249
Os9bglu29  : GDRVKFWATFNEPWTYCSQGYGTG-IHALGRCSPYVS-----TSCAGG---DSSREPYLAAHHVILAHATAVHLY : 252
Os8bglu28  : GDRVKLWNTFNEPTIFCMNGYATG-IMAPGRCSPYAS-----ASCAAGG--DSGREPYVAGHHLLVAHAEAVRLY : 247
Os8bglu27  : GDRVKSWNTFNEPMIFCAGGYGSG-TKAPGRCSPYVS-----KKCAPG---DSGNEPYVAGHNLLLAHAEAVRLY : 246
Os4bglu10  : GDRVKHWITFNEPWNFCSMGYASG-TMAPGRCSSWEK-----GKCRVG---DSGREPYTACHHQLLAHAETVRLY : 257
Os4bglu11  : GDRVKHWITFNEPWTFCSMGYASG-IMAPGRCSSWEV-----GKCRVG---DSGREPYTACHHQLLAHAETVRLY : 257
Os4bglu9   : GDRVKHWITFNEPWIFCSKAYASG-TYAPGRCSPWEM-----GKCSVG---DSGREPYTACHHQLLAHAETVRLY : 257
Os4bglu13  : GDRVKHWITFNEPLSFCVAGYASGGMFAPGRCSPWE------GNCSAG---DSGREPYTACHHQLLAHAETVRLY : 253
Os4bglu12  : GDRVKNWITFNEPWTFCSNGYATG-LFAPGRCSPWEK-----GNCSVG---DSGREPYTACHHQLLAHAETVRLY : 257
Os6bglu24  : GDRVKYWITFNEPWSFSIGGYSNG-ILAPGRCSSQGK-----SGCSKG---DSGREPYIVAHNQLLAHAAVVQIY : 251
AtBGLU17   : GDRVKEWITINEPNMFAVLGYNVG-NIAPGRCSSYV------QNCTVG---NSATEPYLVAHYLILSHAATVQLY : 257
AtBGLU12   : GDRVKHWMTLNEPLTVVQQGYVAG-VMAPGRCSKFTN-----PNCTAG---NGATEPYIVGHNLILAHGEAVKVY : 254
AtBGLU13   : GDRVKHWMTLNEPLTVVQQGYVAG-VMAPGRCSKFTN-----PNCTAG---NGATEPYIVGHNLILAHGEAVKVY : 254
AtBGLU14   : GDRVKHWITLNEPLTVVQQGYVAG-VMAPGRCSKFTN-----PNCTAG---NGATEPYIVGHNLILAHGEAIKVY : 253
AtBGLU15   : GDRVKHWMTLNEPLTVVQQGYVAG-VMAPGRCSKFTN-----PNCTDG---NGATEPYIVGHNLILSHGAAVQVY : 254
AtBGLU16   : GDRVKQWTTLNEPYTMVHEGYITG-QKAPGRCSNFYK-----PDCLGG---DAATEPYIVGHNLLLAHGVAVKVY : 201
AtBGLU18   : GHKVKHWITFNEPWVFSRAGYDNG-KKAPGRCSPYIPGYG--QHCQD---GRSGYEAYQVSHNLLLSHAYAVDAF : 264
AtBGLU19   : GDKVKNWITFNEPWVFSRSGYDVG-KKAPGRCSPYVKEFG--KLCQD---GRSGFEPYVVSHNLLVGHAEAVDAF : 258
AtBGLU20   : GDKVKHWITFNEPWVFSRAGYDIG-NKAPGRCSKYIKEHG--EMCHD---GRSGHEAYIVSHNMLLAHADAVDAF : 262
AtBGLU21   : GGKVKNWITFNEPWVFAHAGYDLG-KKAPGRCSRYVPG------CEDR-EGQSGKEAYLVSHNLLNAHAEAVEVF : 257
AtBGLU22   : GGKVKNWITFNEPWVFAHAGYDVG-KKAPGRCSRYLKG------CEDR-DGRSGYEAYLVSHNLLNAHAEAVEVF : 259
AtBGLU23   : GGKVKHWITFNEPWVFSHAGYDVG-KKAPGRCSSYVNAK-----CQD---GRSGYEAYLVTHNLLISHAEAVEAY : 257
AtBGLU24   : GGKVKHWITFNEPWVFAHAGYDVG-KKAPGRCSPYAKDETVKGDCLG---GRSGYEAYLVSHNLLNAHAEAVEAF : 266
AtBGLU25   : GDRVKHWVTINEPYEFSRGGYETG-EKAPGRCSKYVNEK-----CVA---GKSGHEVYTVSHNLLLAHAEAVEEF : 257
AtBGLU26   : GDRVSLWCTMNEPWVYSVAGYDTG-RKAPGRCSKYVNG-----ASVA---GMSGYEAYIVSHNMLLAHAEAVEVF : 237
AtBGLU27   : GDRVKLWVTLNEPWVYSIGGYDTG-RKAPGRASKYMNE-----AAVA---GESGLEVYTVSHNLLLAHAEAVEVF : 230
AtBGLU28   : GDKVKMWTTINEPYVITVAGYDTG-NKAVGRCSKWVNS-----KCQG---GDSGTEPYIASHHLLLAHAAAVQEF : 251
AtBGLU29   : GDKVKMWTTINEPYVISVAGYDTG-IKAVGRCSKWVNS-----RCQA---GDSAIEPYIVSHHLLLSHAAAVQEF : 251
AtBGLU30   : GDKVKMWTTINEPYIMTVAGYDQG-NKAAGRCSKWVNE-----KCQA---GDSSTEPYIVSHHTLLAHAAAVEEF : 248
AtBGLU31   : GDKVSMWTTFNEPYVYSVSGYDAG-NKAMGRCSKWVNS-----LCIA---GDSGTEPYLVSHHLLLAHAAAVEEF : 254
AtBGLU32   : GDKVSMWTTFNEPYVYSVSGYDAG-NKAIGRCSKWVNS-----LCIA---GDSGTEPYLVSHNLLLAHAAAVEEF : 254
AtBGLU33   : GDRVKNWATFNEPSVYSVAGYSKG-KKAPGRCSKWQAP-----KCPT---GDSSEEPYIVAHNQILAHLAAVDEF : 317
AtBGLU34   : GDRVKFWITLNQPFSLATKGYGDG-SYPPGRCTG---------CELG---GDSGVEPYTVAHNQLLAHAKTVSLY : 261
AtBGLU35   : GDRVKFWITLNQPLSLALKGYGNG-SYPPGRCTG---------CELG---GDSGVEPYTVAHNQLLAHAKTVSLY : 261
AtBGLU36   : GDRVKFWITLNQPYSLAVKGYGDG-QYPPGRCTD---------CEFG---GDSGTEPYIVGHHELLAHMEAVSLY : 235
AtBGLU37   : GDRVKHWITINQLFTVPTRGYALG-TDAPGRCSQWVDK-----RCYG---GDSSTEPYIVAHNQLLAHATVVDLY : 271
AtBGLU38   : GDRVKNWITINQLYTVPTRGYALG-TDAPGRCSPKIDV-----RCPG---GNSSTEPYIVAHNQLLAHAAAVDVY : 259
AtBGLU39   : GDRVKKWITINQLYTVPTRGYAMG-TDAP--------------------------EPYIVAHNQLLAHAKVVHLY : 227
Os4bglu18  : GDRVKYWTTFNEPNVAVRHGYMLG-TYPPSRCSPPFG------HCARGG--DSHAEPYVAAHNVILSHATAIEIY : 248
Os4bglu14  : GDRIKFWTTFNQPNLSIKFSYMDG-FYSPGRCSEPFG------KCALG---NSSIEPYVAGHNIILSHANAVSVY : 244
Os4bglu16  : GDRVRFWTTFNEPNLSTRHQYILG-EFPPNHCSPPFG------NCSSG---DSRREPYAAAHNILLSHAAAVHNY : 249
AtBGLU45   : GNRVKYWTTLNEPNQQLILGYLTG-KFPPSRCSSPYG------NCSQG---NSETEPFIAAHNMILAHAKAVNIY : 254
AtBGLU46   : GDRVKHWITINEPNQHISLAYRSG-LFPPARCSMPYG------NCTHG---NSETEPFIAAHNMILAHAKAIQIY : 251
AtBGLU47   : GDRVKFWSTFNEPNVQVILGYRTG-TYPPSRCSKPFG------NCSCG---DSYIEPLVAAHNIILSHLAAVNLY : 274
Os9bglu33  : GDRVKHWATFNQPNIEPIGGFDAG-DRPPRRCSYPFG-----TNC---TGGDSSTEPYIVAHHLLLAHASAVSIY : 250
Os9bglu32  : GDRVKHWVTVNEPNIEPIGGYDAG-VQPPRRCSYPFG-----TNC---TGGDSSTEPYIVAHHLLLAHASAVSIY : 250
Os9bglu31  : GDRVKHWSTVNEPNIEPIGGYDQG-ILPPRRCSFPFGV----LSC---DNGNSTTEPYIVAHHLLLAHSSAVSLY : 250
Os1bglu2   : GDRVLHWTTLAEPNIAALGGYDTG-VLSPGHCSDPFGL----TEC---TVGNSTVEPYIAAHNMILTHAAVVRLY : 284
Os1bglu3   : GDRVSHWTILAEPNVAALGGYDTG-EFAPGRCSDPFGV----TKC---TVGNSSVEPYVAAHNMILTHAAVVRLY : 244
Os1bglu5   : GDRVAHWITIDEPNVASIGSYDSG-QLAPGRCSDPFGI----RKC---TVGNSSVEPYIAVHNMLLAHASVTKLY : 247
Os5bglu22  : GDRVRHWTTMDEPNVLSIAAYDSG-AFPPCRCSPPFGA----N-C---TAGNSTVEPYVVAHNSILAHASVTRLY : 261
Os5bglu23  : GDLVRHWTTVGEPNVLSIAGYDSG-VIPPCRCSPPFGT----S-C---AAGDSTVEPYVAAHNSILAHASAVRLY : 260
Os5bglu20  : GDRVAHWTTVLEPNVMAQGCYDTG-TLPPNHCSYPFG-----SNC---TGGNSTVEPYLFIHHNLLAHASAVRLY : 252
Os5bglu21  : GDRVVHWTTVLEPNAMAQAGYDMG-ILPPNRCSYPFG-----SNC---TAGNSSVEPYLFIHHSLLAHASAVRLY : 256
Os5bglu19  : GDRVAHWTTSIEPNVMAQSGYDDG-YLPPNRCSYPFGR----SNC---TLGNSTVEPYLFIHHTLLAHASAVRLY : 254
AtBGLU1    : GNHVKFWTTINEANIFTIGGYNDG-NSPPGRCSFPGR------NC---TLGNSSTETYIVGHNLLLAHASVSRLY : 244
AtBGLU3    : GHHVKFWTTINEANIFTIGGYNDG-ITPPGRCSSPGR------NC---SSGNSSTEPYIVGHNLLLAHASASRLY : 237
AtBGLU4    : GNHVKLWTTINEANIFSIGGYNDG-DTPPGRCSKPSK------NC---SSGNSSIEPYIVGHNLLLAHASVSRRY : 239
AtBGLU5    : GNYVKFWTTVNEANVFTIGGYNDG-DTPPGRCSLPGK------NC---LLGNSSTETYIVGHNLLLAHASASRLY : 239
AtBGLU6    : GNHVKFWTTINEGNIFSIGGYNDG-DSPPGRCSIPGQ------NC---LLGNSSTEPYIVGHNLLLAHASVSRLY : 240
AtBGLU7    : GEDVKLWTKINEATLFAIGSYGD--GMRYGHCP-P-------MNY---STANVCTETYIAGHNMLLAHSSASNLY : 236
AtBGLU8    : GEDVKLWTTINEATIFAFAFYGK--DVRYGNC----------------TTGNYCMETYIAGHNMLLAHASASNLY : 230
AtBGLU9    : GEDVKLWTTINEATIFAIGSYDQG-TAPPGHCS-PNKF----VNC---STGNSSTEPYIAGHNILLAHASASKLY : 239
AtBGLU10   : GEDVKLWTTINEATIFAIGSYDQG-ISPPGHCS-PNKF----INC---TSGNSSTEPYLAGHNILLAHASASKLY : 242
AtBGLU11   : GDRVSHWTTINEVNVFALGGYDQG-ITPPARCSPPFG-----LNC---TKGNSSIEPYIAVHNMLLAHASATILY : 244
Os11bglu36 : SNLVDYWVIFNEPHVFVMLTYCAG--AWPGGDP---N------AIEVATSTLPTGVYNQALHWMAIAHSEAYDYI : 355
             g  6  W t  2p      g   g    pgrc            c          ep    H     Ha

                    460         *       480         *       500         *       520
Os10bglu34 : RKKYKA-----------------------SQNGELGISFDVIWYEPMSN---STADIEAAKRAQEFQLGWFADPF : 302
Os3bglu6   : RTKYKA-----------------------TQNGQLGIAFDVMWFEPMSN---TTIDIEAAKRAQEFQLGWFADPF : 314
AtBGLU40   : RKKYKA-----------------------KQGGSLGIAFDVMWFEPESN---KTEDIEAAQRAQDFQLGWFLDPL : 301
Os6bglu25  : EQHFKN-----------------------EQGGLIGIALNSRWYEPFSN---ADEDTEAAARAMDFELGWFLDPL : 289
AtBGLU41   : QRNFKE-----------------------KQRGQIGISLDAKWYEPMSD---CDEDKDAARRAMDFGLGWFMDPL : 300
Os1bglu4   : RRKFKA-----------------------VQGGEVGLVVDCEWAEPFSE---KTEDQVAAERRLDFQLGWYLDPI : 270
AtBGLU42   : RSKYKE-----------------------SQGGQIGLSVDCEWAEPNSE---KPEDKVAADRRIDFQLGWFLDPL : 276
Os1bglu1   : RHKYQH-----------------------IQKGKIGILLDFVWYEGLTN---STADQAAAQRSRDFHVGWFLHPI : 313
Os7bglu26  : REKYQL-----------------------YQKGRIGILLDFVWYEPFSD---SNADRAAAQRARDFHLGWFLDPI : 305
AtBGLU43   : RQNYQE-----------------------KQKGRVGILLDFVWFEPLTS---SQADNDAAQRARDFHVGWFIHPI : 296
AtBGLU44   : RKYYQA-----------------------KQKGRVGILLDFVWYEPLTR---SKADNLAAQRARDFHIGWFIHPL : 307
Os3bglu8   : RNKFQA-----------------------SQKGKIGIVLDFNWYEPLTN---STEDQAAAQRARDFHVGWFLDPL : 310
Os3bglu7   : RTKYQA-----------------------AQQGKVGIVLDFNWYEALSN---STEDQAAAQRARDFHIGWYLDPL : 303
Os12bglu38 : RDKYQA-----------------------IQKGKIGILLDFVWYEPLTD---KEEDHAAAHRAREFTLGWYLHPI : 291
Os11bglu37 : ----QP-----------------------TQKGQIGMVVMTHWFVPYDD---TVAHRGAVQRSLDFMFGWFMDPL : 296
Os11bglu35 : RTRYQP-----------------------TQKGQIGMVVVTHWFVPYDD---TAADRGAVQRSLDFMFGWFMDPL : 300
Os9bglu30  : RTKYQP-----------------------TQKGQIGMVVVTHWFVPYDN---SDADRGAVQRSLDFIYGWFMDPI : 298
Os9bglu29  : RTKYQP-----------------------TQHGQIGITAVSHWFVPYND---TAADRRVVQRSLDFMYGWFLDPI : 301
Os8bglu28  : RARYRA-----------------------AHGGEVGITQVSHWFEPYDAG--SAADRRARRRALDFMLGWFMHPV : 297
Os8bglu27  : RQKYQA-----------------------TQKGQIGITQVSHWFVPYSD---AAADKHAVRRSLDFMYGWFMDPI : 295
Os4bglu10  : KEKYQFTEEAIRQSPFIRDNNLNRRSAKALQKGRIGIILNSEWFVPFSQ---SKSSNDAARRVLDFMLGWFMDPL : 329
Os4bglu11  : KEKYQ-----------------------ALQKGKIGIILNADWFVPLSQ---SKSSSDAARRALDFMLGWFMDPL : 306
Os4bglu9   : REKYQ-----------------------ALQKGKIGIIVNSQWYVPFSQ---SKTNKDAARRVLDFVLGWLMDPL : 306
Os4bglu13  : KEKYQ-----------------------VLQKGKIGITLVSNWFVPFSR---SKSNIDAARRALDFMLGWFMDPL : 302
Os4bglu12  : KAKYQ-----------------------ALQKGKIGITLVSHWFVPFSR---SKSNNDAAKRAIDFMFGWFMDPL : 306
Os6bglu24  : REKYQ-----------------------GGQKGKIGIAIVSNWMIPYED---SKEDKHATKRALDFMYGWFMDPL : 300
AtBGLU17   : REKYQS-----------------------FHGGTIGMTIQTYWMIPKYN---TPACREAAKRALDFFFGWFADPI : 306
AtBGLU12   : REKYKA-----------------------SQKGQVGIALNAGWNLPYSE---SAEDRLAAARAMAFTFDYFMEPL : 303
AtBGLU13   : REKYKA-----------------------SQKGQVGIALNAGWNLPYTE---SAEDRLAAARAMAFTFDYFMEPL : 303
AtBGLU14   : RKKYKA-----------------------SQKGQVGIALNAGWNLPYTE---SAEDRLAAARAMAFTFDYFMEPL : 302
AtBGLU15   : REKYKA-----------------------SQQGQVGIALNAGWNLPYTE---SPKDRLAAARAMAFTFDYFMEPL : 303
AtBGLU16   : REKYQA-----------------------TQKGEIGIALNTAWHYPYSD---SYADRLAATRATAFTFDYFMEPI : 250
AtBGLU18   : RNCKQC-----------------------A-GGKIGIAHSPAWFEPQDL----EHVGGSIERVLDFILGWHLAPT : 311
AtBGLU19   : RKCEKC-----------------------K-GGKIGIAHSPAWFEPEDV----EGGQATVNRVLDFVIGWHLDPT : 305
AtBGLU20   : RKCDKC-----------------------K-GGKIGIAHSPAWFEAHELS--DEEHETPVTGLIDFILGWHLHPT : 311
AtBGLU21   : R--QKV-----------------------K-GGKIGIAHSPAWFEPHDLK--DSNDAPTVSRVLDFMLGWHLEPT : 304
AtBGLU22   : R--QKV-----------------------K-GGKIGIAHSPAWFEPHDLK--DSNDVPTVSRVLDFMLGWHLDPT : 306
AtBGLU23   : RKCEKC-----------------------K-GGKIGIAHSPAWFEAHDLA--DSQDGASIDRALDFILGWHLDTT : 306
AtBGLU24   : RQCEKC-----------------------K-GGKIGIAHSPAWFEPHDFK--DEQSGATIDRALDFIMGWHLDTT : 315
AtBGLU25   : RKCGKC-----------------------T-GGKIGIVQSPMWFEPYDKKSTSSPSEEIVKRAMDFTLGWHMEPI : 308
AtBGLU26   : RKCDHI-----------------------K-NGQIGIAHNPLWYEPYDPS--DPDDVEGCNRAMDFMLGWHQHPT : 286
AtBGLU27   : RNNPKC-----------------------K-DGKIGIAHCPVWFEPYDSN--CPKDIEACERAMEFMFGWHMDPT : 279
AtBGLU28   : RKCNKT-----------------------Q-DGQIGIVLSPLWFEPYDSA--SPADNEAVKRALATELDWHLDPV : 300
AtBGLU29   : RNCNKT-----------------------LQDGKIGIVISPWWLEPYDST--SSADKEAVERGLPLELEWHLNPV : 301
AtBGLU30   : RKCEKT-----------------------SHDGQIGIVLSPRWFEPYHSD--STDDKEAAERALAFEIGWHLDPV : 298
AtBGLU31   : RKCDKI-----------------------SQDSKIGIVLSPYWFEPYDSA--SNADKEAVERALAFNIGWHLSPL : 304
AtBGLU32   : RKCDKI-----------------------SQDAKIGIVLSPYWFEPYDID--SESDKEAVERALVFNIGWHLSPL : 304
AtBGLU33   : RNCKKV-----------------------EGGGKIGIVLVSHWFEPKDPN--SSEDVKAARRSLEYQLGWFLRPL : 367
AtBGLU34   : RKRYQK-----------------------FQGGKIGTTLIGRWFAPLNEF--SELDKAAAKRAFDFFVGWFLDPL : 311
AtBGLU35   : RKRYQK-----------------------FQGGKIGTTLIGRWFVPLNEF--SELDKAAAKRAFDFFVGWFLDPL : 311
AtBGLU36   : RKRYQK-----------------------FQGGKIGTTLIGRWFIPLNET--NDLDKAAAKREFDFSVLGSTGVR : 285
AtBGLU37   : RTRYK------------------------YQGGKIGPVMITRWFLPYDD---TLESKQATWRAKEFFLGWFMEPL : 319
AtBGLU38   : RTKYKD-----------------------DQKGMIGPVMITRWFLPFDH---SQESKDATERAKIFFHGWFMGPL : 308
AtBGLU39   : RKKYKP-----------------------KQRGQIGVVMITRWFVPYDS---TQANIDATERNKEFFLGWFMEPL : 276
Os4bglu18  : KRKYQS-----------------------KQRGMIGMVLYSTWYEPLRD---VPEDRLATERALAFETPWFLDPL : 297
Os4bglu14  : RNKYQG-----------------------KQGGQIGIALSITWYEPFRN---TTIDLLAVKRALSFGASWFLDPI : 293
Os4bglu16  : KTNYQA-----------------------KQGGSIGIVIAVKWYEPLTN---STEDVRAARRALAFEVDWFLDPI : 298
AtBGLU45   : KTKYQK-----------------------EQKGSIGIVVQTSWFEPISD---SNADKEAAERAQSFYSNWILDPV : 303
AtBGLU46   : RTKYQR-----------------------EQKGIIGIVVQTSWFEPISD---SIADKNAAERAQSFYSNWILDPV : 300
AtBGLU47   : RTKFQE-----------------------QQRGQIGIVMNTIWFEPISD---SLADRLAADRAQAFYLTWFLDPV : 323
Os9bglu33  : RQKYQA-----------------------IQGGQIGITLMVRWHEPYTD---KTADAAAAIRMNEFHIGWFLHPL : 299
Os9bglu32  : RRKYQA-----------------------IQGGQIGITLLGWWYEPYTD---AVADAAAAIRMNEFHIGWFMNPL : 299
Os9bglu31  : REKYQA-----------------------TQGGQIGLTLLGWWYEPGTQ---DPEDVAAAARMNDFHIGWYMHPL : 299
Os1bglu2   : REKYQA-----------------------LQKGIVGINMFSLWSYPLTN---SIADLQAAQRYKDFSYGWILHPL : 333
Os1bglu3   : REKYQT-----------------------LQKGIVGINVLSLWSYPLTD---STADLQAAQRYKDFTYGWILHPL : 293
Os1bglu5   : REKYQV-----------------------AGKGIIGISVYTFWAYPLTN---STVDLEATKRCQDFIVHWVLRPL : 296
Os5bglu22  : RDKYQA-----------------------TQEGFVGMNIYSFWNYPFSS---SSADIAATQRALDFMVGWILDPL : 310
Os5bglu23  : WDKYQA-----------------------KQKGVVGTNIYSFWPYPLSR---SCADIDAVQRVLDFTIGWILDPL : 309
Os5bglu20  : REKYQV-----------------------AQKGIVGINIYSLWFYPLTD---SAEDIGATERAKQFMYGWILHPL : 301
Os5bglu21  : REKYKV-----------------------AQKGIIGINIYSMWFYPFTD---SAEDIGATERAKKFIYGWILHPL : 305
Os5bglu19  : REKHQA-----------------------AQKGVVGMNIYSMWFYPLTE---STEDIAATERVKDFMYGWILHPL : 303
AtBGLU1    : KQKYKD-----------------------IQGGSVGFSLFAMNFTPSTN---SKDDEIATKRANDFYLGWMLEPL : 293
AtBGLU3    : KQKYKD-----------------------MQGGSVGFSLFSLGFTPSTS---SKDDDIAVQRAKDFYFGWMLEPF : 286
AtBGLU4    : KQKYKD-----------------------KQGGSIGFSLFILGLIPTTS---SKDDATATQRAQDFYVGWFLRPL : 288
AtBGLU5    : KQKYKD-----------------------KQGGSIGFGLYLMGLTPSTS---SKDDAIATQRAKDFYFGW----- : 283
AtBGLU6    : KQNYKD-----------------------KQGGSIGFSILTIGFSPSTS---SKDDAIATQRANDFFNGWMLGPL : 289
AtBGLU7    : KLKYKT-----------------------KQRGSVGLSIYAYGLSPYTD---SKDDETATERAEAFLFGWMLKPL : 285
AtBGLU8    : KLKYKS-----------------------KQRGSIGLSIFALGLTPYTN---SKDDEIATQRAKAFLYGWMLKPL : 279
AtBGLU9    : KLKYKS-----------------------KQKGSIGLSIFAFGLSPYTN---SKDDEIATQRAKTFLYGWMLKPL : 288
AtBGLU10   : KLKYKS-----------------------TQKGSIGLSIFAFGLSPYTN---SKDDEIATQRAKAFFYGWMLKPL : 291
AtBGLU11   : KQQYKY-----------------------KQHGSVGISVYTYGAVPLTN---SVKDKQATARVNDFYIGWILHPL : 293
Os11bglu36 : HSKSKN-----------------------ERKPIVGVAHHVSFTRP---------------------YGLFDVAA : 386
                                             g 6G         p            a  r      g    p

                 *       540         *       560         *       580         *       600
Os10bglu34 : FFGDY-PATMRSRVGS-RLPKFTEKEAAL-VNGSLDFMGINHYTTFYTKDDQSTVIE---KLLNNTLADTATISV : 371
Os3bglu6   : FFGDY-PATMRARVGE-RLPRFTADEAAV-VKGALDFVGINHYTTYYTRHNNTNIIG---TLLNNTLADTGTVSL : 383
AtBGLU40   : MFGDY-PSSMRSRVGS-RLPVFTGSQSSL-VKGSLDFVGINHYTTYYARNNATNLIG---TLLHDAVSDSGTVTL : 370
Os6bglu25  : MFGHY-PPSMQKLAGD-RLPQFSTHASKL-VSGSLDFVGINHYTTLYARNDRLRIRK---LVMDDASTDSAVIPT : 358
AtBGLU41   : INGDY-PASMKSLVEE-RLPKITPEMYKT-IKGAFDYVGINHYTTLYARNDRTRIRK---LILQDASSDSAVITS : 369
Os1bglu4   : YFGDY-PESMRQRLGD-DLPTFSEKDKEF-IRNKIDFVGINHYTSRFIAHHQDPE-D---IYFYRVQQVERIEKW : 338
AtBGLU42   : FFGDY-PASMRQKLGD-NLPRFTPEEKEFMLQNSWDFLGLNHYTSRLISHVSNKEAE---SNFYQAQELERIVEL : 346
Os1bglu1   : IYGEY-PKSLQVIVKE-RLPKFTADEVHM-VKGSIDYVGINQYTAYYVRDQQPNATT---LPS-YSSDWHAAPIY : 381
Os7bglu26  : IHGRY-PYSMLEIVKD-RMPTFSDEESRM-VKDSIDYVGINHYTSFYMKDPGPWNLT---PTS-YQDDWHVGFAY : 373
AtBGLU43   : VYGEY-PNTLQNIVKE-RLPKFTEEEVKM-VKGSIDFVGINQYTTYFMSDPKISTTP---KDLGYQQDWNVTFNF : 365
AtBGLU44   : VYGEY-PKTMQNIVKE-RLPKFTEKEVKM-VKGSIDFVGINQYTTYYMSEPHPTTKP---KDLGYQQDWNVEFGF : 376
Os3bglu8   : INGQY-PKNMRDIVKE-RLPTFTPEQAKL-VKGSADYFGINQYTANYMADQPAPQQA---ATS-YSSDWHVSFIF : 378
Os3bglu7   : INGHY-SQIMQDLVKD-RLPKFTPEQARL-VKGSADYIGINQYTASYMKGQQLMQQT---PTS-YSADWQVTYVF : 371
Os12bglu38 : TYGHY-PETMQNAVKE-RLPNFTREQSEM-IKGSADYIAINHYTTYYVS--HHVNKT---SIS-YLNDWDVKISY : 357
Os11bglu37 : VHGDY-PGTMRGWLGD-RLPKLTLAQSAM-VKGSYDFIGINYYTTYYAKSMPP-PNS---NELSYDVDSRANTTG : 364
Os11bglu35 : VHGDY-PGTMRGWLGD-RLPKFTPAQSAM-VKGSYDFIGINYYTTYYAKSVPP-PNS---NELSYDVDSRANTTG : 368
Os9bglu30  : VHGDY-PGTMRGWLGN-RLPEFTPEQSAM-VKGSYDFIGVNYYTTYYAKSIPP-PNS---NELSYDLDNRANTTG : 366
Os9bglu29  : VHGDY-PGTMRGWLGA-RLPAFTAEQAAA-VRGSYDFIGVNYYTTYYAKSVPL-PSS---NRLSYDTDIRANTTG : 369
Os8bglu28  : AHGEY-PPAMRRLVGG-RLPAFTAEQSEM-LRGSFDFIGLNYYTSNYAVAAPP-PNK---LHPSYLTDNWVNATG : 365
Os8bglu27  : VFGDY-PGTMRKLVGD-RLPKFTAEQSEL-VKGSYDFIGLNYYTTNYAKSVLRRPSK---LKPAYATDNWVNQTA : 364
Os4bglu10  : IRGDY-PLSMRELVGN-RLPEFSKEQSEM-VKGAFDFIGLNYYASSYADNDPP-SYG---HNNSYNTDSHAKITG : 397
Os4bglu11  : IRGDY-PLSMRELVGN-RLPEFSKEQSGM-VKGAFDFIGLNYYTSSYADNDPP-SHG---HNNSYNTDAHAKITG : 374
Os4bglu9   : IRGDY-PLNMRELVGN-RLPKFTKEQSEM-VKGAFDFIGLNYYSSSYAENVLP-SYG---LKNSYNTDFHARITG : 374
Os4bglu13  : IRGEY-PLSMRELVRN-RLPQFTKEQSEL-IKGSFDFIGLNYYTSNYAGSLPP-SNG---LNNSYSTDARANLTA : 370
Os4bglu12  : IRGDY-PLSMRGLVGN-RLPQFTKEQSKL-VKGAFDFIGLNYYTANYADNLPP-SNG---LNNSYTTDSRANLTG : 374
Os6bglu24  : TKGDY-PVSMRTLVGN-RLPRFTKEQSKA-INGSFDFIGLNYYTARYIQGTKQ-DSN---SHKSYSTDSLTNERV : 368
AtBGLU17   : TYGDY-PKTMRELVGN-RLPKFTKKQSKM-VRGSFDFFGLNYYTSRYVEDVMF-YAN---TNLSYTTDSRVNQTT : 374
AtBGLU12   : VTGKY-PIDMVNYVKGGRLPTFTAKQSKM-LKGSYDFIGRNYYSSSYAKDVPC--SS---ENVTLFSDPCASVTG : 371
AtBGLU13   : VTGKY-PVDMVNNVKDGRLPTFTAKQSKM-LKGSYDFIGINYYSSSYAKDVPC--SS---ENVTLFSDPCASVTG : 371
AtBGLU14   : VTGKY-PVDMVNNVKGGRLPTFTSKQSNM-LKGSYDFIGINYYSSSYAKDVPC--SS---ENVTMFSDPCASVTG : 370
AtBGLU15   : VTGKY-PVDMVNNVKG-RLPIFTAQQSKM-LKGSYDFIGINYYSSTYAKDVPC--ST---KDVTMFSDPCASVTG : 370
AtBGLU16   : VYGRY-PIEMVSHVKDGRLPTFTPEESEM-LKGSYDFIGVNYYSSLYAKDVPC--AT---ENITMTTDSCVSLVG : 318
AtBGLU18   : TYGDY-PQSMKDRVGH-RLPKFTEAEKKL-LKGSTDYVGMNYYTSVFAKEIS--PDP---KSPSWTTDSLVDWDS : 378
AtBGLU19   : TFGDY-PQSMKDAVGS-RLPRFTKAQKAK-LKDSTDFVGINYYTSFFAKADQK-VDS---RNPTWATDALVEFEP : 373
AtBGLU20   : TYGDY-PQSMKDHIGH-RLPKFTEAQKEK-LKNSADFVGINYYTSVFALHDEE-PDP---SQPSWQSDSLVDWEP : 379
AtBGLU21   : TSGDY-PQIMKDLLGY-RLPQFTAAQKAK-LKDSTDFVGLNYYTSTFSNYNEK-PDP---SKPSWKQDSLVSWEP : 372
AtBGLU22   : TFGDY-PQIMKDLLGH-RLPKFTSSQKAK-LKDSTDFVGLNYYTSTFSNHNEK-PDP---STPSWKQDSLVAWEP : 374
AtBGLU23   : TFGDY-PQIMKDIVGH-RLPKFTTEQKAK-LKASTDFVGLNYYTSVFSNHLEK-PDP---SKPRWMQDSLITWES : 374
AtBGLU24   : MFGDY-PQTMKDIVGH-RLPKFTTEQIAK-LKNSADFVGINYYTSTFSKHLEK-PNH---AEPKFKQDSLVEWKN : 383
AtBGLU25   : THGDY-PQAMKDVVGS-RLPSFTPEQKEK-LKGSYDFVGINYFTSTFVAHTDN-VNP---EKPSWEADSRLQLHS : 376
AtBGLU26   : ACGDY-PETMKKSVGD-RLPSFTPEQSKK-LIGSCDYVGINYYSSLFVKSIKH-VDP---TQPTWRTDQGVDWMK : 354
AtBGLU27   : VYGDY-PAVMKKSIGK-RLPSFTAAQSKK-LRGSFDFVGVNYYSAFYVKNIDE-VNH---DKPNWRSDARIEWRK : 347
AtBGLU28   : IHGDY-PEMMKKLAGN-RLPSFTPEQSKM-LKNSSDFIGINYYTARYVAHIPQ-ADP---ARPRFVTDHQLQWRV : 368
AtBGLU29   : IYGDY-PETMKKHVGN-RLPAFTPEQSKM-LINSSDFIGVNYYSIHFTAHLPH-IDH---TRPRFRTDHHFEKKL : 369
AtBGLU30   : IHGDY-PEIVKKYAGN-KLPSFTVEQSKM-LQNSSDFVGINYYTARFAAHLPH-IDP---EKPRFKTDHHVEWKL : 366
AtBGLU31   : VFGDY-PETIKISAGN-RLPSFTKEQSMM-VKNSFDFIGVNYYTARFVAHDLN-VDI---SRPRFMTDQHLQYKL : 372
AtBGLU32   : VFGDY-PETIKTTAGN-RLPSFTKEQSMM-LQNSFDFIGINYYTARFVAHDLH-VDL---SRPRFTTDQHLQYKL : 372
AtBGLU33   : TYGQY-PAEMLEDVNI-RLREFTPEESEK-LRKSLDFVGLNYYGAFFSTPLAK-VNS---SQLNYETDLRVNWTV : 435
AtBGLU34   : VYGKY-PTIMREMVGD-RLPEFTPEQSAL-VKGSLDFLGLNYYVTQYATDAPP--P----TQLNAITDARVTLGF : 377
AtBGLU35   : VYGKY-PTIMREMVGD-RLPEFTPEESAL-VKGSLDFLGLNYYVSQYATDAPP--P----TQPNAITDARVTLGF : 377
AtBGLU36   : TISK---D--NERLGD-RLPKFTPKQSAL-LKGSLDFLGLNYYVTRYATYRPPPMP----TQHSVLTDSGVTIGF : 349
AtBGLU37   : TKGKY-PYIMRKLVGN-RLPKFNSTEARL-LKGSYDFLGLNYYVTQYAHALDPSPP----EKLTAMTDSLANLTS : 387
AtBGLU38   : TEGKY-PDIMREYVGD-RLPEFSETEAAL-VKGSYDFLGLNYYVTQYAQNNQTIVPS---DVHTALMDSRTTLTS : 377
AtBGLU39   : TKGKY-PDIMRKLVGR-RLPKFNKKEAKL-VKGSYDFLGINYYQTQYVYAIPANPP----NRLTVLNDSLSAFSY : 344
Os4bglu18  : VYGDY-PPEMRQILGG-RLPSFSPEDRRK-LRYKLDFIGVNHYTTLYARDCMFSDCP---QGQ--ETQHALAAVT : 364
Os4bglu14  : LLGDY-PTEMREVLGQ-SLPKFTSKQKNRLQSTKLDFIGLNHYTTCYVKDCIFSPCE---IDP--VNADARVFSL : 361
Os4bglu16  : FFGDY-PREMREILSS-NLPKFTPEEKKLLQNNKVDFIGINHYTAIYAKDCIYSPCT---LDT--YEGNALVYAI : 366
AtBGLU45   : IYGKY-PKEMVDILGP-ALPQFSSNEVKNLEKSRADFVGINHYTSYFIQDCLTSACN---TGHGAFKAEGYALKL : 373
AtBGLU46   : VYGKY-PEEMVNLLGS-ALPKFSSNEMNSLMSYKSDFLGINHYTSYFIQDCLITACN---SGDGASKSEGLALKL : 370
AtBGLU47   : VFGRY-PREMREILGD-DLPEFTKDDLKS-SKNALDFIGINQYTSRYAKDCLHSVCE---PGKGGSRAEGFVYAN : 392
Os9bglu33  : VHGDY-PPVMRSRVGV-RLPSITASDSEK-IRGSFDFIGINHYYVIFVQSIDAN----EQKLRDYYIDAGVQ--- : 364
Os9bglu32  : VHGDY-PPVMRSRVGA-RLPSITASDSEK-IRGSFDFIGINHYFVIFVQSSDANH---DQKLRDYYVDAGVQENG : 368
Os9bglu31  : VYGDY-PPVMRKNVGS-RLPSFTAEESKR-VLESYDFVGFNHYVAIFVRADLSKL---DQSLRDYMGDAAVKYDL : 368
Os1bglu2   : VFGDY-PQVMKKTIGS-RLPSFSQVQTEL-IKGAIDFIGINHYYSAYVNYRPLVE-----GVRDYVADRSVSAR- : 399
Os1bglu3   : VFGDY-PQVMKKAIGS-RLPSFSKVQTEL-VKGTLDFIGVNHYFSLYVSDLPLAK-----GVRDFIADRSVSCRG : 360
Os1bglu5   : VFGDY-PQVMKNIVGS-RLPSFTKAQSED-VKGSLDFIGMNHYYSLYVNDRPLGK-----GTRDFVADISIYYRG : 363
Os5bglu22  : VYGDY-PEIMKKKAGS-RIPSFTEEQSEL-IRGSADFIGINHYTSVYISDASNGETV---GPRDYSADMAATFRI : 379
Os5bglu23  : VYGDY-PEIMKKQAGS-RIPSFTKEQSEL-IRGSADFIGINHYKSLYVSDGSNREKA---GLRDYNADMAAHFRG : 378
Os5bglu20  : VFGDY-PETIKKVVGS-RLPFFSNHESEL-VTNAFDFVGLNHYSSVYTSNNNNVVKA---PLQDLTADIATLFRA : 370
Os5bglu21  : VFGDY-PDTMKKAAGS-RLPIFSNHESEM-VTNSFDFIGLNHYSSVYTSNNNNVVKA---PLQDLTADVATLFRV : 374
Os5bglu19  : VFGDY-PETMKKAAGS-RLPLFSDYESEL-VTNAFDFIGLNHYTSNYVSDNSNAVKA---PLQDVTDDISSLFWA : 372
AtBGLU1    : IYGDY-PDVMKRTIGS-RLPVFSKEESEQ-VKGSSDFIGVIHYLTALVTNIDINPSL--SGIPDFNSDMG----- : 358
AtBGLU3    : IFGDY-PDEMKRTVGS-RLPVFSKEESEQ-VKGSSDFIGIIHYLAASVTSIKIKPSI--SGNPDFYSDMGVSMTW : 356
AtBGLU4    : LFGDY-PDTMKRTIGS-RLPVFSEKESEQ-VKGSCDFVGVIHYHAASVTNIKSKPSL--SGNPDFYSYMET--DF : 356
AtBGLU5    : --------------------------------------------------------------------------- :   -
AtBGLU6    : IYGDY-PDTMKRIVGS-RMPVFSEEESEQ-VKGSSDYIGINHYLAASITNSKLKPSI--SGNPDFYSDMNV--IL : 357
AtBGLU7    : VVGDY-PDIMKRTLGS-RLPVFSEEESKQ-VKGSSDFVGVVHYNTFYVTNRPAPSLV-TSINKLFFADIGAYLIA : 356
AtBGLU8    : VFGDY-PDEMKRTLGS-RLPVFSEEESEQ-VKGSSDFVGIIHYTTVYVTNQPAPYIFPSSTNKDFFTDMGAYIIS : 351
AtBGLU9    : VFGDY-PDEMKKTVGS-RLPVFSEEESEQ-VKGSSDFIGIIHYTTFYVTNHQPSASLFPSMGEGFFKDMGVYIIP : 360
AtBGLU10   : VFGDY-PDEMKRTVGS-RLPVFSEEESEQ-LKGSSDFIGIIHYTTFYVTN-KPSPSIFPSMNEGFFKDMGVYMIS : 362
AtBGLU11   : VFGDY-PETMKTNVGS-RLPAFTEEESEQ-VKGAFDFVGVINYMALYVKDNSSSLKP---NLQDFNTDIAVEMTL : 362
Os11bglu36 : VALAN-------SLT--LFPYVDSI-CD---KL--DFIGINYYGQEVISGPGLKLVDN----DEYSESGRG---- : 438
               g y p            p               d  g n                          d

                      *       620         *       640         *       660         *
Os10bglu34 : PFRNGQ---------PIGDRANSIWLYIVPRSM-RILMNYVKDRYNKP-TVYITENGM----DDGNSPF--ISLK : 429
Os3bglu6   : PFKNGK---------PIGDRANSIWLYIVPRGM-RSLMNYVKERYNSP-PVYITENGM----DDSNNPF--ISIK : 441
AtBGLU40   : PFK-GLS--------TIGDRASSIWLYIVPRGM-RSLMNYIKHRYGNP-PVFITENGM----DDPNSIL--ISRK : 428
Os6bglu25  : AYRHGK---------KIGETAASSWLHIVPWGM-FKLMKHVKEKYGNP-PVVITENGM----DDANHPF--SRLE : 416
AtBGLU41   : SFRGGV---------AIGERAGSSWLHIVPWGI-RKLAVYVKDIYGNP-PVFITENGM----DEKNSPF--IDME : 427
Os1bglu4   : NT--GE---------KIGERAASEWLFIVPWGL-RKLLNYAAKRYGNP-VIYVTENGM----DEEDDQS--ATLD : 394
AtBGLU42   : EN--GD---------LIGERAASDWLYAVPWGI-RKTLNYMSKKYNHP-PIFITENGM----DDEDDGS--ASIH : 402
Os1bglu1   : ER-DGV---------PIGPRANSDWLYIVPWGL-YKAVTYVKEKYGNP-TMFLSENGM----DDPGN----VTIA : 436
Os7bglu26  : ER-NGV---------PIGAQANSYWLYIVPWGI-NKAVTYVKETYGNP-TMILSENGM----DQPGN----VSIT : 428
AtBGLU43   : AK-NGT---------PIGPRAHSEWLYNVPWGM-YKALMYIEERYGNP-TMILSENGM----DDPGN----ITLT : 420
AtBGLU44   : AK-LGK---------PIGPRAYSSWLYNVPWGM-YKALMYMKERYGNP-TMILSENGM----DDPGN----VTLA : 431
Os3bglu8   : QR-NGV---------PIGQQANSNWLYIVPTGM-YGAVNYIKEKYNNP-TIIISENGM----DQSGN----LTRE : 433
Os3bglu7   : AK-NGK---------PIGPQANSNWLYIVPWGM-YGCVNYIKQKYGNP-TVVITENGM----DQPAN----LSRD : 426
Os12bglu38 : ER-NGV---------PIGKQAYSNWLYVVPWGI-YKAVMHVKEKYKDP-IIIIGENGI----DQPGN----ETLP : 412
Os11bglu37 : FR-NGK---------PISPQEFTPIFFNYPPGI-REVLLYTKRRYNNP-AIFT-ENGI----DEGNNSTVP---- : 418
Os11bglu35 : FR-NGK---------PIGPQ-FTPIFFNYPPGI-REVLLYTKRRYNNP-AIYITENGI----DEGNNSTVP---- : 422
Os9bglu30  : FR-NGK---------PIGPQEFTPIFFNYPPGL-RELLLYTKRRYNNP-TIYVTENGI----DEGNNSTLP---- : 421
Os9bglu29  : FR-NGK---------PIGPQEFTPIFFNYPPGL-RELLLYTKRRYNNP-IIYVTENGI----AEGNNKSLPI--T : 426
Os8bglu28  : YR-NSI---------PIGPPAYTPIFFNYPPGL-RELLLYVKRRYNNP-TIYITENGT----DEANNSTIPI--S : 422
Os8bglu27  : YR-NGV---------PIGPPAFTKIFFTYAPGL-RELLLYTKRKYNDP-DIYIAENGT----DEANNSTIPI--A : 421
Os4bglu10  : SR-NGI---------PIGPQAASFWFYIYPEGL-RELLLHIKENYGNP-TIYITENGV----DEINNKTMRL--K : 454
Os4bglu11  : SR-NGI---------PIGPQAASFWFHIYPEGI-CEMLLYVKENYGNP-TIYITENGV----DEVNNKTMPL--E : 431
Os4bglu9   : SR-NGT---------LIGPQAASSWLHIYPQGL-RELLLYIKENYGNP-TIFITENGV----DEVNNKTMPL--K : 431
Os4bglu13  : VR-NGI---------PIGPQAASPWLYIYPQGF-RELVLYVKENYGNP-TIYITENGV----DEFNNKTLPL--Q : 427
Os4bglu12  : VR-NGI---------PIGPQAASPWLYVYPQGF-RDLLLYVKENYGNP-TVYITENGV----DEFNNKTLPL--Q : 431
Os6bglu24  : ER-NGT---------DIGPKAGSSWLYIYPKGI-EELLLYTKRTYNNP-TIYITENGV----DEVNNENLSL--K : 425
AtBGLU17   : EK-NGV---------PVGEPTSADWLFICPEGF-QDVLLYIKSKFQNP-VILVTENGM----PSENDKSLSV--N : 431
AtBGLU12   : ER-EGV---------PIGPKAASDWLLIYPKGI-RDLLLYAKYKFKDP-VMYITENGR----DEASTGKID---- : 426
AtBGLU13   : ER-EGV---------PIGPKAASDWLLIYPKGI-RDLLLYAKYKFKDP-VMYITENGR----DEASTGKID---- : 426
AtBGLU14   : ER-DG--------------------------GI-RDLILYAKYKFKDP-VMYITENGR----DEASTGKIL---- : 408
AtBGLU15   : ER-DGV---------PIGPKAASDWLLIYPKGI-RDLVLYAKYKFKDP-VMYITENGR----DEFSTNKIF---- : 425
AtBGLU16   : ER-NGV---------PIGPAAGSDWLLIYPKGI-RDLLLHAKFRYNDP-VLYITENGV----DEANIGKIF---- : 373
AtBGLU18   : KSVDGY---------KIGSKPFNGKLDVYSKGL-RYLLKYIKDNYGDP-EVIIAENGY----GEDLGEKHN-DVN : 437
AtBGLU19   : KTVDGSI--------KIGSQPNTAKMAVYAKGL-RKLMKYIKDRYNSP-EIIITENGY----GEDLGDKDT-DLS : 433
AtBGLU20   : RYVDKFN--------AFANKPDVAKVEVYAKGL-RSLLKYIKDKYGNP-EIMITENGY----GEDLGEQDT-SLV : 439
AtBGLU21   : KNVDHS---------AIGSMPLTAALPVYAKGF-RKLLKYIKDKYANP-EIMIMENGY----GDKLGTTD--SVD : 430
AtBGLU22   : KNVDHS---------AIGSQPLTAALPVYAKGF-RSLLKYIKDKYANP-EIMIMENGY----GDKLKDKD--SVE : 432
AtBGLU23   : KNAQNY---------AIGSKPLTAALNVYSRGF-RSLLKYIKDKYANP-EIMIMENGY----GEELGASD--SVA : 432
AtBGLU24   : KNVNNI---------TIGSKPETGPLPVYSTGF-RKVLKYVKDKYANP-EIIIMENGY----GENLKEND--SVE : 441
AtBGLU25   : NNVDGF---------KIGSQPATAKYPVCADGL-RKVLKYIKENYNDP-EIIVTGNGY----KETLEEKD--VLP : 434
AtBGLU26   : TNIDGK---------QIAKQGGSEWSFTYPTGL-RNILKYVKKTYGNP-PILITENGY----GEVAEQSQSLYMY : 414
AtBGLU27   : ENNAGQ---------TLGVRGGSEWDFLYPQGL-RKFLNYAKNKYESP-KFMITENGH----CDIDYEKK--PKL : 405
AtBGLU28   : TNHSNH---------QFGPGEDRGILQSHPEGL-RKVLNYIKDKYNNP-IVYIKENGI----NDYDDGTK--SRE : 426
AtBGLU29   : INRSNH---------ETGPGDDRGKIHSHPEGL-RRVLNYIKDKYNNP-IVYVKENGI----DHYDDGTK--SRE : 427
AtBGLU30   : TNHSGH---------IIGPGEERGFLFSHPEGL-RKVLNYIKERYNNM-PVYIKENGI----NDNDDGTK--PRE : 424
AtBGLU31   : TNRTGDT--------ISLESDGTKILWSYPEGL-RKILNYIKNKYNNP-TIYITENGF----DDYENGTV--TRE : 431
AtBGLU32   : TNRSGDH--------ISSESDGTKILWSYPEGL-RKLLNYIKNKYNNP-TIYITENGF----DDYENGSV--TRE : 431
AtBGLU33   : ITNNLS---------LPDLQTTSMGIVIYPAGL-KNILKHIKDEYMDP-EIYIMENGM----DEIDYGTK--NIT : 493
AtBGLU34   : Y-RNGV---------PIGVVAP--SFVYYPPGF-RQILNYIKDNYKNP-LTYITENGV----ADLDLG-N-VTLA : 432
AtBGLU35   : Y-RNGS---------PIGVVAS--SFVYYPPGF-RQILNYIKDNYKNP-LTYITENGV----ADLDLG-N-VTLA : 432
AtBGLU36   : E-RNGV---------SIGVKA---SINFDVKDL-RHLVDFFLFVELLLLSTRIPSDSK----SHQKQELL-MLIA : 405
AtBGLU37   : LDANGQ---------PPGPPFSK-GSYYHPRGM-LNVMEHFKTKYGDP-LIYVTENGF----STSGGP---IPFT : 443
AtBGLU38   : KNATGH---------APGPPFNA-ASYYYPKGI-YYVMDYFKTTYGDP-LIYVTENGF----STPGD----EDFE : 432
AtBGLU39   : ENKDG----------PIGPWFNA-DSYYHPRGI-LNVLEHFKTKYGNP-LVYITENGELLILSGCNVK---GYFA : 403
Os4bglu18  : GESNGL---------PIGTPTAMPTFYVVPDGI-EKMVKYFMRRYNNL-PMFITENGY----AQGGDSYT--DAE : 422
Os4bglu14  : YERDGV---------PIGKATGAPFFHDVPRGM-EEAVTYYKQRYNNT-PTYITENGY----SQASNSNM--TAK : 419
Os4bglu16  : GRRNGK---------IIGKPTALHGYFVVPEAM-EKVVMYVNDRYRNT-TIYITENGY----SQHSDT----SME : 422
AtBGLU45   : DRKGNV---------TIGELTDVNWQHIDPTGF-HKMLNYLKDRYPNM-PMFITENGF----GDLQKPET--TDK : 431
AtBGLU46   : DRKGNV---------SIGELTDVNWQHIDPNGF-RKMLNYLKNRYHNI-PMYITENGF----GQLQKPET--TVE : 428
AtBGLU47   : ALKDGL---------RLG----------EPVGM-EEMLMYATERYKNI-TLYVTENGF----G-----EN--NTG : 435
Os9bglu33  : ------------------GEDDTENIQCHSWSL-GKVLNHLKLEYGNP-PVMIHENGY---------SDSPDIFG : 410
Os9bglu32  : -----------------GGGFDKEHYQLHPWAL-GKMLHHLKLKYGNP-PVMIHENGD---------ADSPETPG : 415
Os9bglu31  : PFLKSNN---EF-----PLGLTSDFMTSTPWAL-KKMLNHLQEKYKNP-IVMIHENGA---------AGQPDPSG : 424
Os1bglu2   : ----VRFININMNVGN----YEPTEYPNDPKGL-QLALEYLRESYGDF-PFYIEENGK--------------GST : 450
Os1bglu3   : LLQGVRFIAQTMQA--------PTRSMGDPHGL-QLMLQHLKESYGDL-PIYVQENGK--LKLDLLPVFSGKASS : 423
Os1bglu5   : ----SKTDPPPGKAA-------PTSIGPDPQGL-RLMVQYLQETYGNL-PIYILENGY--------------GSS : 411
Os5bglu22  : SRN-------DTPSGQ----FVPTRLPRDPKGL-QCMLEYLRDTYQGI-PVYIQENGF-----------GHFGKD : 430
Os5bglu23  : TT------------------YAPSKTLSDPKGL-QCMLEYLKDTYEGI-PVYVQENGF-----------GQFDKE : 422
Os5bglu20  : TKNDTPT------------PEFLPGNTVDPQGL-ENALEYIRENYGNL-TIYIQENGS--------------GAP : 417
Os5bglu21  : TKNDTPT------------PVFVPGTIVDPRGL-EHALKYIREKYGNL-PIYIQENGI----------ASGSGSS : 425
Os5bglu19  : SKNSTPTRETVTWFCLLLLRQFLPGTSLDPRGL-ELALEYLQEKYGNL-LFYIQENGS--------------GS- : 430
AtBGLU1    : ------------------------------------ILEYIKQSYGNP-PVYILENGK-----------TMNQDL : 385
AtBGLU3    : TVLGNFS---------------AFEYAVAPWAM-ESVLEYIKQSYGNP-PIYILENGT-----------PMKQDL : 403
AtBGLU4    : GKSLDF------------------QYANTPWAM-EVVLEYIKQSYGNP-PVYILESAKFT-FQWQQIGTPMKQDS : 410
AtBGLU5    : --------------------------------------------------------------------------- :   -
AtBGLU6    : SFFANFS---------------SSEYDVAPWAI-EAVL------------------------------------- : 379
AtBGLU7    : AGNASL-----------------FEFDAVPWGL-EGILQHIKQSYNNP-PIYILENGK-----------PMKHGS : 401
AtBGLU8    : TGNSSS-----------------FVFDAVPWGL-EGVLQHIKHRYNNP-PIYILENGS-----------PMKHDS : 396
AtBGLU9    : TGNSSF-----------------LVWEATPWGL-EGILEYIKQSYNNP-PVYILENGM-----------PMVRDS : 405
AtBGLU10   : AANSSF-----------------LLWEATPWGL-EGILEYIKQSYNNP-PIYILENGM-----------PMGRDS : 407
AtBGLU11   : VGNTSIE---------------N-EYANTPWSL-QQILLYVKETYGNP-PVYILENGQ-----------MTPHSS : 408
Os11bglu36 : ---------------------------VYPDGLFRILIQFNERYKRLNIPFVITENGV--------------SDE : 472
                                          p g                      e g

               680         *       700         *       720         *       740         *
Os10bglu34 : NALKD-DKRTKYHNDYLT-NLADSIREDGCDVRGYFAWSLLDNWEWAAGY-TSRFGLYYVDYK-----NRKRYPK : 496
Os3bglu6   : DALKD-SKRIKYHNDYLT-NLAASIKEDGCDVRGYFAWSLLDNWEWAAGY-SSRFGLYFVDYKD----NLKRYPK : 509
AtBGLU40   : DALKD-AKRIKYHHDYLS-SLQASIKEDGCNVKGYFVWSLLDNWEWAAGY-SSRFGLYFVDYRD----NLKRYPK : 496
Os6bglu25  : DVLQD-DKRIQYHNDYMS-NLLDAIRKEGCNVHGYFVWSLLDNWEWNSGY-TVRFGLYYIDYKN----NLTRIPK : 484
AtBGLU41   : KALKD-DKRIGFHRDYLS-NLSAAIRNDECDVRGYFVWSLLDNWEWNSGY-TVRFGIYYVDYKN----NLTRIPK : 495
Os1bglu4   : QVLND-TTRVGYFKGYLA-SVAQAIK-DGADVRGYFAWSFLDNFEWAMGY-TKRFGIVYVDYKN----GLSRHPK : 461
AtBGLU42   : DMLDD-KRRVDYFKSYLA-NVSQAIE-DGVDIKGYFAWSLLDNFEWAQGY-TKRFGLVYVDYKN----GLTRHPK : 469
Os1bglu1   : QGVHD-TTRVAYYRSYIT-KLKEAID-DGANCIGYFAWSLLDNFEWKLGY-TSRFGLVYVDFR-----TLRRYPK : 502
Os7bglu26  : QGVHD-TVRIRYYRNYIT-ELKKAID-DGAKVIGYFAWSLLDNFEWRLGY-TSRFGIVYVDYK-----TLKRYPK : 494
AtBGLU43   : QGLND-TTRVKYYRDYLV-QLKKAVD-DGANLTGYFAWSLLDNFEWLSGY-TSRFGIVYVDYK-----DLKRYPK : 486
AtBGLU44   : QGLHD-TTRIKYYKDYLT-NLKKARD-DGANVVGYFAWSLLDNFEWLSGY-TSRFGIVYVDYK-----TLKRYPK : 497
Os3bglu8   : EFLHD-TERIEFYKNYLT-ELKKAID-DGANVVAYFAWSLLDNFEWLSGY-TSKFGIVYVDFT-----TLKRYPK : 499
Os3bglu7   : QYLRD-TTRVHFYRSYLT-QLKKAID-EGANVAGYFAWSLLDNFEWLSGY-TSKFGIVYVDFN-----TLERHPK : 492
Os12bglu38 : GALYD-FFRIQYFDQYLH-ELKRAIK-DGARVTGYFAWSLLDNFEWRLGF-TSKFGIVYVDRS-----TFTRYPK : 478
Os11bglu37 : EALRD-GHRIEFHSKHL--FVNHAIR-NGVNVKGYFTWTFMDCFEWGDGY-LDLFGLIYVDRKT-----LTRYRK : 483
Os11bglu35 : EALRD-GHRIEFHSKHLQ-FVNHAIR-NGVNVKGYFT-TFMDCFEWGDGY-LDRFGLIYVDRKT-----LTRYRK : 487
Os9bglu30  : EALKD-GHRIEFHSKHLQ-FVNHAIK-NGVNVKGYFTWTFMDCFEWGDGY-LDRFGLIYVDRKT-----LKRYRK : 487
Os9bglu29  : EALKD-GHRIEFHSKHLQ-FVNHAIK-NGVNVKGYFTWTFMDCFEWGDGY-LDRFGLIYIDRLN----NLKRYHK : 493
Os8bglu28  : EALKD-ETRIGFHYKHLQ-FVHKAIQ-EGVKVKGYFTWTFMDCFEFGDGF-KDRFGLIYVDRAT-----LARFRK : 488
Os8bglu27  : EALKD-DNRISFHYQHLR-FTQLAIK-EGVKVKGYFTWTFMDDFEWGDGY-TGRFGLIYVDRET-----LKRYRK : 487
Os4bglu10  : EALKD-DIRIEYYHKHLL-ALLSAMR-DGANVKGYFAWSLLDNFEWSEGY-TVRFGINFVDYDN----GMKRYPK : 521
Os4bglu11  : EALKD-DTRIEYYHKHLL-ALLSAMR-DGANVKGYFAWSLLDNFEWAEGY-TVRFGINFVDYDD----GMKRYPK : 498
Os4bglu9   : EALND-NTRIEYYHKHLL-ALRNAMR-DGANVKGYFAWSLLDNFEWADGY-TLRFGLNFVDYDD----GMKRHPK : 498
Os4bglu13  : EALKD-DTRIDYYHKHLL-SLLSAIR-DGANVKGYFAWSLLDNFEWSNGY-TVRFGINFVDYND----GAKRYPK : 494
Os4bglu12  : EALKD-DARIEYYHKHLL-SLLSAIR-DGANVKGYFAWSLLDNFEWSNGY-TVRFGINFVDYND----GRKRYPK : 498
Os6bglu24  : EALID-TTRIEFYRQHLF-HVQRALR-QGVDVRGYFAWSLFDNFEWMDGY-SVRFGINYIDYKD----GLKRYPK : 492
AtBGLU17   : IALND-EAKIKYHQLHLT-ALLEAVS-QGADVRGYYIWSLMDDFEWEFGY-KYRYGLVYVDFQD----GLKRHLK : 498
AtBGLU12   : --LKD-SERIDYYAQHLK-MVQDAIS-IGANVKGFFAWSLLDNFEWATGY-AVRFGLVYVDFNG----GRKRYPK : 491
AtBGLU13   : --LKD-SERIDYYAQHLK-MVQDAIS-IGANVKGFFAWSLLDNFEWATGY-SVRFGLVYVDFND----GRKRYPK : 491
AtBGLU14   : --LKD-GDRIDYYARHLK-MVQDAIL-IGANVKGFFAWSLLDNFEWASGY-TVRFGLVYVDFND----RRKRYLK : 473
AtBGLU15   : --LKD-GDRIDYYARHLE-MVQDAIS-VGANVKGFFAWSLLDNFEWAMGY-TVRFGLVYVDFKD----GCKRYPK : 490
AtBGLU16   : --LND-DLRIDYYAHHLK-MVSDAIS-IGVNVKGYFAWSLMDNFEWSEGY-TVRFGLVFVDFED----GRKRYLK : 438
AtBGLU18   : FGTQD-HNRKYYIQRHLL-SMHDAICKDKVNVTGYFVWSLMDNFEWQDGY-KARFGLYYIDFQN----NLTRHQK : 505
AtBGLU19   : VALND-HNRKYYLQRHLL-ALNEAICEDKVNVTSYFLWSLMDNFEWQDGY-TARFGVYYIDFKN----NLTRMEK : 501
AtBGLU20   : VALSD-QHRTYYIQKHLL-SLHEAICDDKVNVTGYFHWSLMDNFEWQDGY-KARFGLYYVDYKN----NLTRHEK : 507
AtBGLU21   : VGTAD-HNRKYYLQRHLL-AMNEAICIDKVRVTGYFVWSLLDNFEWQDGY-KNRFGLYYVDFKN----NLTRYEK : 498
AtBGLU22   : VGTAD-YNRKYYLQRHLL-AMNEAICIDKVRVTGYFVWSLLDNFEWQDGY-NNRFGLYYVDFKN----NLTRYEK : 500
AtBGLU23   : VGTAD-HNRKYYLQRHLL-SMQEAVCIDKVNVTGYFVWSLLDNFEWQDGY-KNRFGLYYVDFKN----NLTRYEK : 500
AtBGLU24   : NGTAD-YNRESYLKKHLW-SMHKAICEDKVNVTGYFVWSLMDNFEWQDGF-KNRFGLYYIDYKN----NLTRHEK : 509
AtBGLU25   : DALSD-SNRKYYHMRHLM-ALHGAVCEDKVNVKGYFVSSLMDGLEWEDGY-KTRSGLYYVDYGH----NMGRHEK : 502
AtBGLU26   : NPSID-TERLEYIEGHIH-AIHQAIHEDGVRVEGYYVWSLLDNFEWNSGY-GVRYGLYYIDYKD----GLRRYPK : 482
AtBGLU27   : SNLMD-LQRTEYHKKHLQ-SIQQAIQEDGVVVEGYFAWSLLDNCEWNAGY-GVRYGLFYVDYNN----GLKRFPK : 473
AtBGLU28   : EILND-TFRISYHEDHLQ-QLQKAIIEDGCDVRGYYVWSLLDNFEWEHGY-STRFGVYYVDYDN----DLTRIPK : 494
AtBGLU29   : TILKD-TFRISYHQDHLK-QVHKAIM------------------------------------------------- : 451
AtBGLU30   : EIVKD-TFRIEYHKTHFE-ELHKAIVEDGCDVRGYYAWSLMDNFEWEHGY-TARFGLYYVDFVN----GLKRYPK : 492
AtBGLU31   : EILED-TKRIEYHQKHLQ-ELQKAITEDGCDVKGYFTWSLLDNFEWEHGY-AVRFGLYYVDYKN----GLQRHAK : 499
AtBGLU32   : EIIED-TKRIEYHQNHLQ-QLQKAITEDGCNVKGYFTWSLLDNFEWEHGY-AVRFGLYYVDYKN----GLSRHAK : 499
AtBGLU33   : EATND-YGRKEFIKSHIL-IMGKSIRMDKVRLKGYYIWSLMDNFEWDKGY-KVRFGLYYVDYND----NMKRYIR : 561
AtBGLU34   : TALAD-NGRIQNHCSHLS--CLKCAMKDGCNVAGYFAWSLMDNYEFGNGY-TLRFGMNWVNFTN----PADRKEK : 499
AtBGLU35   : TALAD-NGRIQNHCSHLS--CLKCAMKDGCNVAGYFAWSLMDNYEFGNGY-TLRFGMNWVNFTN----PADRKEK : 499
AtBGLU36   : NALAD-NGRIQFQCSHLS--CLKCAIEDGCNVAGYFAWSLMDNYEFGNGY-TLRFDMNWVNFTN----PADRREK : 472
AtBGLU37   : EAFHD-YNRIDYLCSHLC-FLRKAIK------------------------------------------------- : 467
AtBGLU38   : KATAD-YKRIDYLCSHLC-FLSKVIKEKNVNVKGYFAWSLGDNYEFCNGF-TVRFGLSYVDFANI---TGDRDLK : 501
AtBGLU39   : WCLGDNYELWPSRSFHVSPFYLLHRKDKGAFPS-FEA-------------------------------------- : 439
Os4bglu18  : DWIDD-EDRIEYLEGYLT-KLAKVIR-DGADVRGYFAWSVVDNFEWLFGY-TLRFGLYYIDYRT-----QERSPK : 488
Os4bglu14  : DFTND-TGRITYIQGYLI-SLASAIR-KGADVRGYFVWSLLDDFEWNFGY-TLRFGLYHVHYKT-----LKRTPK : 485
Os4bglu16  : DLIND-VERVNYMHDYLK-YLSSAIR-KGANVGGYFAWSIVDNFEWVYGY-TVKFGLYQVDFDT-----QERIPR : 488
AtBGLU45   : ELLND-TKRIQYMSGYLE-ALQAAMR-DGANVKGYFVWSLLDNFEWLFGY-KVRFGLFHVDLTT-----LKRSPK : 497
AtBGLU46   : ELLHD-TKRIQYLSGYLD-ALKAAMR-DGANVKGYFAWSLLDNFEWLYGY-KVRFGLFHVDFTT-----LKRTPK : 494
AtBGLU47   : VLLND-YQRVKFMSNYLD-ALKRAMR-KGADVRGYFAWSLLDNFEWISGY-TIRFGMYHVDFST-----QERTPR : 501
Os9bglu33  : KINYNDDFRSAFLQGYLE-ALYLSVR-NGSNTRGYFVWSMFDMFEFLYGY-RLRFGLCGVDFTAA---ARTRYLK : 479
Os9bglu32  : KIDYDDDFRSDFLQSYLE-VLHLSIR-NGSNTRGYFVWSLLDGFEFLSGY-GNRFGLCGVDFTAP---ARTRYVR : 484
Os9bglu31  : GNTYDDDFRSQYLQDYIE-ATLQSIR-NGSNVQGYFVWSFLDVFEYLFGY-RLRFGLYGVDFASP---ERTRYQR : 493
Os1bglu2   : NDSLDDPDRVDYIKGYIG-GVLDAIR-NGVDVRGYFVWSFVDVFELLEGY-QSRFGLYRVDFDDG---AR-RRAA : 518
Os1bglu3   : NDSLDDTDRVDYIKGYIE-GVLNATR-NGVNARGYFAWFFVDMFELLSGY-QTRYGLYRVDFDDA---ALPRRAK : 492
Os1bglu5   : NDTVHDNDRVDYLKSYIG-SILTALR-NGANVKGYFVWSFVDVFEYLTGY-GQSYGLYRVDFADE---SRPRQAR : 480
Os5bglu22  : DDSLNDTDRVDYLSSYMG-STLAALR-NGANVKGYFVWSFLDVFELLAGY-HSPFGLHYVDFEDP---NLPRQPK : 499
Os5bglu23  : D-SLNDTERVEYLSSYMG-GTLAALR-NGANVKGYFVWSFLDVFELFAGY-HSPFGLHHVDFEDP---SLPRQPK : 490
Os5bglu20  : DGTLDDVERINYLQKYIA-ATLKAIR-NGANVKGYSMWSFIDIYEIFGGYNSWHYGLVAVDFGST---ERRRQPR : 487
Os5bglu21  : SETLDDVERINYLAKYIA-ATLKAIS--GANVKGYSMWSFVDLYELFGGYSTWHFGLVAVDFDSE---KRRRQPR : 494
Os5bglu19  : NATLDDVGRIDCLTQYIA-ATLRSIR-NGANVKGYCVWSFMDQYEMFGDY-KAHFGIVAVDFGSE---ELTRQPR : 499
AtBGLU1    : ELQQKDTPRIEYLDAYIG-AVLKAVR-NGSDTRGYFVWSFMDLYELLNGY-KSSFGLYSVNFSDP---HRKRSPK : 454
AtBGLU3    : QLQQKDTPRIEYLHAYIA-AVLKSIR-NGSDTRGYFIWSFMDLYELVKGY-EFSFGLYSVNFSDP---HRTRSPK : 472
AtBGLU4    : QLKQKDIPRVEYLHAYIG-GVLKSIR-NGSDTRGYFVWSFMDLYELLGGY-EVGFGLYTVNFSDP---HRKRSPK : 479
AtBGLU5    : --------------------------------------------------------------------------- :   -
AtBGLU6    : --------------------------------------------------------------------------- :   -
AtBGLU7    : TLQ--DTPRAEFIQAYIG-AVHNAIT-NGSDTRGYFVWSMIDLYELIGRY-MTSYGMYYVNFSDP---GRKRSPK : 468
AtBGLU8    : MLQ--DTPRVEYIQAYIG-AVLNAIK-SGSDTRGYFVWSLIDLFEVQVGY-KSSFGMYYVNFSDP---GRKRSPK : 463
AtBGLU9    : TLQ--DTQRIEYIQAYID-AVLNAMK-NGSDTRGYFVWSMVDVYEILSGY-TTSFGMYHVNFSDP---GRKRTPK : 472
AtBGLU10   : TLQ--DTQRIEFIQAYIG-AMLNAIK-NGSDTRGYFVWSMIDLYELLSGY-TTSFGMYYVNFSDP---GRKRTPK : 474
AtBGLU11   : SLV--DTTRVKYLSSYIK-AVLHSLSRKGSDVKGYFQWSLMDVFELFGGY-ERSFGLLYVDFKDP---SLKRSPK : 476
Os11bglu36 : T----DLIRKPYILEHLLATYAAII-MGVRVLGYLFWTTSDN-WEWADGY-GPKFGLVAVDRAN---NLARK-PR : 536
                                         g    g   w      e   g      g

                    760         *       780         *       800         *       820
Os10bglu34 : NSVQWFKNLLASSS------------------------------------------------------------- : 510
Os3bglu6   : NSVQWFKALLKT--------------------------------------------------------------- : 521
AtBGLU40   : DSVHWFTSFLNSTS------------------------------------------------------------- : 510
Os6bglu25  : ASVQWFSQVLAQKTAII---------------------------------------------------------- : 501
AtBGLU41   : ASARWFQTILSGSSSTSDSSKLILLEEATEQQQEYKFQEK----------------------------------- : 535
Os1bglu4   : ASARWFSRFLKGDDAENKADMN----------------------------------------------------- : 483
AtBGLU42   : SSAYWFMKFLKGDE-ENKGKKE----------------------------------------------------- : 490
Os1bglu1   : MSAYWFRDLVSSKN------------------------------------------------------------- : 516
Os7bglu26  : DSAFWFKNMLSSKKRN----------------------------------------------------------- : 510
AtBGLU43   : MSALWFKQLLKRDQK------------------------------------------------------------ : 501
AtBGLU44   : MSAQWFKQLLKRNNK------------------------------------------------------------ : 512
Os3bglu8   : DSANWFKNMLQASGPGSKSGSGTSDSQVGSATSASHPVGSAISSSHRLLLPLLLSLHFLFPSFFMFLSL------ : 568
Os3bglu7   : ASAYWFRDMLKH--------------------------------------------------------------- : 504
Os12bglu38 : DSTRWFRKMIKSEV------------------------------------------------------------- : 492
Os11bglu37 : DSSYWIEDFLRRQH------------------------------------------------------------- : 497
Os11bglu35 : DSSYWIEDFLK---------------------------------------------------------------- : 498
Os9bglu30  : ESSYWIEDFLKRH-------------------------------------------------------------- : 500
Os9bglu29  : QSSYWIANFLKRKKY------------------------------------------------------------ : 508
Os8bglu28  : KSSYWFADFLRR--------------------------------------------------------------- : 500
Os8bglu27  : KSSYWFADFLKR--------------------------------------------------------------- : 499
Os4bglu10  : NSARWFKKFLRK--------------------------------------------------------------- : 533
Os4bglu11  : NSARWFKKFLQKSNRDGNKRLKRVAYNAFSN-------------------------------------------- : 529
Os4bglu9   : NSAHWFKKFLREMKQG----------------------------------------------------------- : 514
Os4bglu13  : MSAHWFKEFLQK--------------------------------------------------------------- : 506
Os4bglu12  : NSAHWFKKFLLK--------------------------------------------------------------- : 510
Os6bglu24  : RSSQWLQNFLHN--------------------------------------------------------------- : 504
AtBGLU17   : SSALWYHHFLSNSSSYQMD-------------------------------------------------------- : 517
AtBGLU12   : KSAKWFKKLLNEKK--KN--------------------------------------------------------- : 507
AtBGLU13   : KSAKWFRKLLSEKK--RN--------------------------------------------------------- : 507
AtBGLU14   : KSAHWFRHLLNGKK--EN--------------------------------------------------------- : 489
AtBGLU15   : KSAEWFRKLLNEKK--ND--------------------------------------------------------- : 506
AtBGLU16   : KSAKWFRRLLKGAHGGTNEQVAVI--------------------------------------------------- : 462
AtBGLU18   : VSGKWYSEFLKPQFP--------TSKLREEL-------------------------------------------- : 528
AtBGLU19   : ESAKWLSEFLKPGLKP-----SKSSKLHEEL-------------------------------------------- : 527
AtBGLU20   : LSAQWYSSFLHDGSKEFE---IEHEFEHDEL-------------------------------------------- : 535
AtBGLU21   : ESAKYYKDFLAQGVRP-------SALKRDEL-------------------------------------------- : 522
AtBGLU22   : ESAKYYKDFLGQGVRP-------SALKKDEL-------------------------------------------- : 524
AtBGLU23   : ESGKYYKDFLSQGVRP-------SALKKDEL-------------------------------------------- : 524
AtBGLU24   : VSGKYYREFLSEGVRP-------SAIKKDEL-------------------------------------------- : 533
AtBGLU25   : QSAKWLSKLLEKVPDTIQS--KVDSDSRKEL-------------------------------------------- : 531
AtBGLU26   : MSALWLKEFLRFDQED------DSSTSKKEEKKESYGKQLLHSVQDSQFVHSIKDSGALPAVLGSLFVVSATVGT : 551
AtBGLU27   : MSAMWFKEFLKREEEI------EDSEEEEYVLKSTMNKKRFLLATGSSASCFIPKMSESSKALELLF-------- : 534
AtBGLU28   : DSVNWFKQFLDV-----KNKEIWDVSHKERYNKTFDDVESFEASVGSILYLMTNNISRREEEERDQCAFGNLNDQ : 564
AtBGLU29   : --------------------------------------------------------------------------- :   -
AtBGLU30   : DSVKWFKRFLKKSVVGESNKEEVEEMSRAEGNKTFKG---FEESAGFFASFMAMNQSRRDEEN-NRCSFDFPHTH : 563
AtBGLU31   : HSAMWFKHFLER-------------SGKPMPMDLFKSVKRWWSTLQMI--------------------------- : 534
AtBGLU32   : NSAKWFKHFLQR-------------SGKPMPLDLFKSVKNWWSAIPMI--------------------------- : 534
AtBGLU33   : SSGKWLSEFLDSKETLHK---CYFEGHREKGYAPKLFDVEYLEPENSQLSYRSDFM------------------- : 614
AtBGLU34   : ASGKWFSKFLAK--------------------------------------------------------------- : 511
AtBGLU35   : ASGKWFSKFLAK--------------------------------------------------------------- : 511
AtBGLU36   : ASGKWFSRFIAK--------------------------------------------------------------- : 484
AtBGLU37   : --------------------------------------------------------------------------- :   -
AtBGLU38   : ASGKWFQKFINVTDEDSTNQDLLRSSVSSKNRDRKSLADA----------------------------------- : 541
AtBGLU39   : --------------------------------------------------------------------------- :   -
Os4bglu18  : LSALWYKEFLQN--------LHENQ-------------------------------------------------- : 505
Os4bglu14  : LSVDWYRKFLTGS-LLRRKFRDESQLHKFNSY------------------------------------------- : 516
Os4bglu16  : MSAKWYRDFLTSS-SLTDGLQVRSRRADS---------------------------------------------- : 516
AtBGLU45   : QSASWYKNYIEEH--VNRRDIVDNY-------------------------------------------------- : 520
AtBGLU46   : QSATWYKNFIEQN--VNIEDQIDK--------------------------------------------------- : 516
AtBGLU47   : LSASWYKNFIFQHRALSKDDWCLKQKEDTNFFLI----------------------------------------- : 535
Os9bglu33  : NSARWYSGFLRGGELRPEKSYATL--------------------------------------------------- : 503
Os9bglu32  : SSARWYSDFLNGGELRPVKPFESGRY------------------------------------------------- : 510
Os9bglu31  : HSARWYAGFLRGGELRPAAAALAGGGAYSQ--------------------------------------------- : 523
Os1bglu2   : AASEALRSLVLGLSQGKEGSRADCSAVNRSLALANQIAQEISR-------------------------------- : 561
Os1bglu3   : RSARWYRDFLKSKRQ------------------PLQIAQQ----------------------------------- : 514
Os1bglu5   : LSARWYSGFLKNREMDVD-------QSELAMAAAESRAQQ----------------------------------- : 513
Os5bglu22  : LSAHWYSKFLRGEIGIN---------IESTISPDEHEHEHADQ-------------------------------- : 533
Os5bglu23  : LSAQWYSKFLRSEIGIN---------IEKMISPDEHEHAYYQ--------------------------------- : 523
Os5bglu20  : RSASWYSDFLKNNAPIRVEMAP------LSLQPLMLNSE------------------------------------ : 520
Os5bglu21  : RSASWYSEFLKNNSVIRVEED--------GFVSAASHAQL----------------------------------- : 526
Os5bglu19  : RSARWYSDFLKNNAVIKVDDG---------SVSTAFHAQL----------------------------------- : 530
AtBGLU1    : LSAHWYSGFLKGKPTFLGSQGITQLHSNFSSSR------------------------------------------ : 487
AtBGLU3    : LSAHWYSAFLKGNTTFLGSQGIMQMQSNFSSSASS---------------------------------------- : 507
AtBGLU4    : LSAYWYSDFLKGESAFLDSQGIKELQSKYSSSF------------------------------------------ : 512
AtBGLU5    : --------------------------------------------------------------------------- :   -
AtBGLU6    : --------------------------------------------------------------------------- :   -
AtBGLU7    : LSASWYTGFLNG-TIDVASQDTIQLQRKCSGSSSL---------------------------------------- : 502
AtBGLU8    : LSASWYTGFLNG-TIDVASQDMTQLQRNFSGSSSL---------------------------------------- : 497
AtBGLU9    : LSASWYTGFLNG-TIDVASQDTIQLWSNFSVSSSL---------------------------------------- : 506
AtBGLU10   : LSASWYTGFLNG-TIDVATQDTIQLQSNISGSSSL---------------------------------------- : 508
AtBGLU11   : LSAHWYSSFLKG-TLHHPSYASS---------------------------------------------------- : 498
Os11bglu36 : PSYFLFSRVVTTGKITRQDRMSAWRELQQAAVQKKTRPFFRAVDKHGRMYAGGLDRPIQRPFILRDWRFGHYKME : 611
              s

                 *       840         *       860
Os10bglu34 : ------------------------------------ :   -
Os3bglu6   : ------------------------------------ :   -
AtBGLU40   : ------------------------------------ :   -
Os6bglu25  : ------------------------------------ :   -
AtBGLU41   : ------------------------------------ :   -
Os1bglu4   : ------------------------------------ :   -
AtBGLU42   : ------------------------------------ :   -
Os1bglu1   : ------------------------------------ :   -
Os7bglu26  : ------------------------------------ :   -
AtBGLU43   : ------------------------------------ :   -
AtBGLU44   : ------------------------------------ :   -
Os3bglu8   : ------------------------------------ :   -
Os3bglu7   : ------------------------------------ :   -
Os12bglu38 : ------------------------------------ :   -
Os11bglu37 : ------------------------------------ :   -
Os11bglu35 : ------------------------------------ :   -
Os9bglu30  : ------------------------------------ :   -
Os9bglu29  : ------------------------------------ :   -
Os8bglu28  : ------------------------------------ :   -
Os8bglu27  : ------------------------------------ :   -
Os4bglu10  : ------------------------------------ :   -
Os4bglu11  : ------------------------------------ :   -
Os4bglu9   : ------------------------------------ :   -
Os4bglu13  : ------------------------------------ :   -
Os4bglu12  : ------------------------------------ :   -
Os6bglu24  : ------------------------------------ :   -
AtBGLU17   : ------------------------------------ :   -
AtBGLU12   : ------------------------------------ :   -
AtBGLU13   : ------------------------------------ :   -
AtBGLU14   : ------------------------------------ :   -
AtBGLU15   : ------------------------------------ :   -
AtBGLU16   : ------------------------------------ :   -
AtBGLU18   : ------------------------------------ :   -
AtBGLU19   : ------------------------------------ :   -
AtBGLU20   : ------------------------------------ :   -
AtBGLU21   : ------------------------------------ :   -
AtBGLU22   : ------------------------------------ :   -
AtBGLU23   : ------------------------------------ :   -
AtBGLU24   : ------------------------------------ :   -
AtBGLU25   : ------------------------------------ :   -
AtBGLU26   : SLFFKGANN--------------------------- : 560
AtBGLU27   : ------------------------------------ :   -
AtBGLU28   : SGLLLESYNSFGFLENVW------------------ : 582
AtBGLU29   : ------------------------------------ :   -
AtBGLU30   : FGVLQGIENPSSFY---------------------- : 577
AtBGLU31   : ------------------------------------ :   -
AtBGLU32   : ------------------------------------ :   -
AtBGLU33   : ------------------------------------ :   -
AtBGLU34   : ------------------------------------ :   -
AtBGLU35   : ------------------------------------ :   -
AtBGLU36   : ------------------------------------ :   -
AtBGLU37   : ------------------------------------ :   -
AtBGLU38   : ------------------------------------ :   -
AtBGLU39   : ------------------------------------ :   -
Os4bglu18  : ------------------------------------ :   -
Os4bglu14  : ------------------------------------ :   -
Os4bglu16  : ------------------------------------ :   -
AtBGLU45   : ------------------------------------ :   -
AtBGLU46   : ------------------------------------ :   -
AtBGLU47   : ------------------------------------ :   -
Os9bglu33  : ------------------------------------ :   -
Os9bglu32  : ------------------------------------ :   -
Os9bglu31  : ------------------------------------ :   -
Os1bglu2   : ------------------------------------ :   -
Os1bglu3   : ------------------------------------ :   -
Os1bglu5   : ------------------------------------ :   -
Os5bglu22  : ------------------------------------ :   -
Os5bglu23  : ------------------------------------ :   -
Os5bglu20  : ------------------------------------ :   -
Os5bglu21  : ------------------------------------ :   -
Os5bglu19  : ------------------------------------ :   -
AtBGLU1    : ------------------------------------ :   -
AtBGLU3    : ------------------------------------ :   -
AtBGLU4    : ------------------------------------ :   -
AtBGLU5    : ------------------------------------ :   -
AtBGLU6    : ------------------------------------ :   -
AtBGLU7    : ------------------------------------ :   -
AtBGLU8    : ------------------------------------ :   -
AtBGLU9    : ------------------------------------ :   -
AtBGLU10   : ------------------------------------ :   -
AtBGLU11   : ------------------------------------ :   -
Os11bglu36 : GLQDPLSCFIRCIFAPFSRQKKIHYIEDDVISYSIN : 647


```
