# Supplementary material for: Analysis of rice glycosyl hydrolase family 1 and expression of Os4bglu12 β-glucosidase
Source: BMC Plant Biol. 2006 Dec 29;6:33. doi: 10.1186/1471-2229-6-33 (PMC1781453; doi:10.1186/1471-2229-6-33)
Supplement: Additional File 2 — Alignment of derived sequences of rice and Arabidopsis after removal of end regions and large gaps for use in phylogenetic tree generation. The alignment in Additional file 1 was edited in Genedoc to remove the nonconserved N-terminal and C-terminal sequences and most of the large gap regions. This adjusted alignment was used for generation of the phylogenetic trees shown in Figure 2. Darkest shading represents highest conservation, and the consensus for highly conserved regions is shown below the alignment. The file was exported as a rich text (.rtf) document for this picture. [file 1471-2229-6-33-S2.doc]

```
Supplementary Data Figure 1B: Alignment of rice and Arabidopsis GH1 proteins with large gap regions removed for phylogenetic analysis.
                      *        20         *        40         *        60         *
Os10bglu34 : LTRKSFPNGFVFGTASSAYQYEGAVKEDGRGPTIWDKFAH-TFGK-IIDFSNADVAVDQYHRFEEDIQLMADMGM :  73
Os3bglu6   : LTRGSFPEGFVFGTASAAYQYEGAVKEDGRGQTIWDTFAH-TFGK-ITDFSNADVAVDQYHRFEEDIQLMADMGM :  73
AtBGLU40   : ISRGSFPKGFVFGTASSAFQHEGAVKAEGRGPTIWDTFSH-TFGK-ITDFSNADVAVDQYHRYEEDVQLMKNMGM :  73
Os6bglu25  : ISRADFPPGFIFGTASSAYQYEGAVNEGQRGPTIWDTLTK-RPGR-VIDFSNADVAVDHYHRYKEDVELMNDIGM :  73
AtBGLU41   : ISRANFPDGFVFGTASSAYQFEGAVKEGNKGESIWDTFTKEKPGK-ILDFSNADTTVDQYHRFHNDIDLMKDLRM :  74
Os1bglu4   : T-RGDFPDGFVFGVATSAYQIEGARREGGKGDNIWDVFTE-NKER-ILDGSSGEVAVDHYHRYKEDIELMASLGF :  72
AtBGLU42   : THRSNFPSTFTFGVATSAYQIEGGWNEGKKGPSIWDKFTH-IEGK-ILDGSNGDVAVDHYHRYKEDVDLIGQLGF :  73
Os1bglu1   : LSRRSFPAGFVFGTAASAYQVEGMALKDGRGPSIWDAFVK-TPGE-IANNATADVTVDEYHRYKEDVNIMKSMGF :  73
Os7bglu26  : LSRRAFPEGFVFGTAASAYQVEGMAKQGGRGPSIWDAFIE-KPGT-IPNNATADVTVDEYHRYKEDVNIMKNMGF :  73
AtBGLU43   : LNRKSFPEGFLFGTATSAYQVEGETHQDGRGPSIWDAFVK-IPGK-IANNATAEITVDQYHRYKEDVDLMQNLNI :  73
AtBGLU44   : LSRQSFPKGFVFGTATSAYQVEGETHQDGRGPSIWDAFVK-IPGK-IAKNATAEITVDQYHRYKEDVDLMKKLNF :  73
Os3bglu8   : LSRAAFPKGFVFGTATSAFQVEGMAASGGRGPSIWDPFVH-TPGN-IAGNGNADVTTDEYHRYKEDVDLLKSLNF :  73
Os3bglu7   : LSRAAFPKRFVFGTATSAYQVEGMAASGGRGPSIWDAFAH-TPGN-VAGNQNGDVATDQYHRYKEDVNLMKSLNF :  73
Os12bglu38 : LTRETFPAGFVFGTASSAYQVEGNALQYGRGPCIWDTFLM-QPGV-TPDNSTANVTVDEYHRYMDDVDNMVRVGF :  73
Os11bglu37 : FSRYSFPKDFIFGTGSAAYQYEGAYKEGGKGPSIWDTFTH-VPGK-ILNNDTGDVASDFYHRYKEDVNLLKDMNM :  73
Os11bglu35 : FSRYSFPKDFIFGTGSAAYQYEGAYKEGGKGPSIWDTFTH-IPGK-ILNNDTGDVANDFHPDTREDVNLLKDMNM :  73
Os9bglu30  : FTRYSFPKDFIFGTGSAAYQYEGAYKEGGKGPSVWDNFTH-IPGK-ILNNDNGDVANDFYHRYKEDVSLLKDMNM :  73
Os9bglu29  : FNRYSFPKDFIFGTGSAAYQYEGAAKEGGRGPSVWDTFSH-IPGK-ILNGDTGDVADDFYHRYKEDVNLLKDMNM :  73
Os8bglu28  : LNRHSFPEGFLFGTGTSAYQYEGAVDK--RGQNIWDTFSR-IPGK-IADGSNADIANDFYHRYKEDLNLITAMNM :  71
Os8bglu27  : FNRFSFPEDFIFGTGSAAYQYEGAVNEGGRGPSIWDTYAH-IPGK-VEDGSNGDVAVDFYHRYKEDLNFVTDMNM :  73
Os4bglu10  : ISRRSFPKGFIFGTSSSSYQFEGAAAKGGRGPSIWDTFTHQYPDK-ITDKSNGDGACNSYHLYKEDVRIMKEMGM :  74
Os4bglu11  : ISRRSFPKGFIFGTSSSSYQFEGGAVLGGRGPSIWDTFTHQSPDK-ITDRSNGDVACDSYHLYKEDVRSMKEMGM :  74
Os4bglu9   : ISRRSFPKGFIFGTSSASYQCEGGAAEGGRGSSIWDTFTYQHPDK-IADKSNGDVADNTYHLYKEDVHMMKEMGM :  74
Os4bglu13  : ISRRSFPEGFIFGTASSSYQYEGGAREGGRGPSIWDTFTHQHPDK-IADKSNGDVAADSYHLYKEDVRIMKDMGV :  74
Os4bglu12  : VSRRSFPKGFIFGTASSSYQYEGGAAEGGRGPSIWDTFTHQHPEK-IADRSNGDVASDSYHLYKEDVRLMKDMGM :  74
Os6bglu24  : -RRSQFPEDFFFGTASSAYQYEGAVREGGRGPSIWDTFTHNHPEK-IANGSNGDIAIDSYHRYKEDVGIMKGLGL :  73
AtBGLU17   : LQRSSFPQDFRFGAASSAYQSEGAANVDGREPSIWDTFTKQYPEK-ISDGSNGDVADEFYYRFKEDVAHMKEIGL :  74
AtBGLU12   : LRRSDFPEDFIFGAATSAYQVEGAAHEDGRGPSIWDTFSEKYPEK-IKDGSNGSIASDSYHLYKEDVGLLHQIGF :  74
AtBGLU13   : LRRSDFPKDFIFGAATSAYQVEGAAHEDGRGPSIWDTFSEKYPEK-IKDGTNGSIASDSYHLYKEDVGLLHQIGF :  74
AtBGLU14   : LRKTDFPEDFIFGAATSAYQVEGAAQEDGRGPSIWDTFSEKYPEK-IKDGSNGSIADDSYHLYKEDVGLLHQIGF :  74
AtBGLU15   : LRRSDFPEDFIFGSATSAYQVEGGAHEDGRGPSIWDTFSEKYPEK-IKDGSNGSVADNSYHLYKEDVALLHQIGF :  74
AtBGLU16   : V------------------QVSG-------TPS-------------LK---NS----------QEDVNLLHQIGF :  24
AtBGLU18   : FSRLNFPEGFIWGTATAAFQVEGAVNEGCRGPSMWDTFTKKFP-H-RCENHNADVAVDFYHRYKEDIQLMKDLNT :  73
AtBGLU19   : LSRASFPEGFMFGTATAAFQVEGAVNEGCRGPSLWDIYTKKFP-H-RVKNHNADEAVDFYHRYKEDIQLMKKLNT :  73
AtBGLU20   : FTRANFPKGFIFGTATAAFQVEGAVNEGCRGPSMWDVYTKKFP-H-KCNYHNADVAVDFYHRYKEDIKLMKNLNT :  73
AtBGLU21   : LSRASFPNGFLFGTATAAFQVEGAINETCRGPALWDIYCRRNP-E-RCSGDHADVAVDFFHRYKEDIQLMKNLNT :  73
AtBGLU22   : LSRASFPNGFVFGTATAAFQVEGAINETCRGPALWDIFCKRNP-E-RCSGHNADVAVDFFHRYKEDIQLMKNLNT :  73
AtBGLU23   : LSRASFPEGFLFGTATAAYQVEGAINETCRGPALWDIYCRRYP-E-RCNNDNGDVAVDFFHRYKEDIQLMKNLNT :  73
AtBGLU24   : LSRAHFPKGFLFGTATAAYQVEGAVNETCRGPSVWDIYCKKYP-E-KCNGDNGTQAVDFFYRYKEDIQLMKNLNT :  73
AtBGLU25   : FGRGSFPDGFLFGATTSAFQHEGAAEEGGRGSSIWDSFTLKQHSE-SNNNLDGRLGVDFYHHYKEDVQLLKKLNM :  74
AtBGLU26   : KGRASFPKGFLFGTASSSYQYEGAVNEGARGQSVWDHFSNRFPHR-ISDSSDGNVAVDFYHRYKEDIKRMKDINM :  74
AtBGLU27   : FGRSDFPEGFLFGTASSAYQYEGARNEAPRGESVWDTFVRKYPER-NCYSN-ADQAIEFYNHYKDDIQRMKDINM :  73
AtBGLU28   : FDRHGFPDNFVFGTAASAFQYEGATSEGGKSPSIWDYFSHTFP-E-RTRMQNADVAVDFYHRYKDDIKLMKELNM :  73
AtBGLU29   : LDRSSFPDDFVFGTAISAFQSEGATSEGGKSPTIWDYFSHTFP-E-RTNMQNADVAVDFYHRYKDDIKLIEELNV :  73
AtBGLU30   : LDRHSFPDDFIFGTAASAFQYEGATSEGGKSPTIWDHFSLTYP-E-RTKMHNADVAIDFYHRYKDDIKLMKELNM :  73
AtBGLU31   : LNRYSFPPHFDFGVASSAYQYEGAVEEGGRSLSIWDNFTHAFP-E-RTNMDNGDVAVDFYHRYKEDIKLIKEMNM :  73
AtBGLU32   : LNRYSFPPHFDFGVASSAYQYEGAVEEGGRSPSIWDNFTHAFP-E-RTNMDNGDVAVDFYHRYKDDIKLIKEMNM :  73
AtBGLU33   : IHKQDFPADFIFGTSVSAYQVEGAKKGSGRGLTSWDEFTHMFPEK-VQQNGDGDEGVDFYTRYKDDIKLMKELNT :  74
AtBGLU34   : FNRTGFPRNFTFGAATSAYQIEGAAH---RALNGWDYFTHRYPEK-VPDRSSGDLACDSYDLYKDDVKLLKRMNV :  71
AtBGLU35   : FNRSGFPKNFTFGAATSAYQIEGAAH---RALNGWDYFTHRYPEK-VPDRSSADLACDSYDLYKDDVKLLKRMNV :  71
AtBGLU36   : ISQ----KNFTFGAATSAYQVEGAAH---RALNGWDYFTHRYPER-VSDRSIGDLACNSYDLYKDDVKLLKRMNV :  67
AtBGLU37   : FNKQDFESDFIFGVASSAYQIEGGRG---RGLNVWDGFTHRYPEKGGADLGNGDTTCDSYRTWQKDLDVMEELGV :  72
AtBGLU38   : FNSGNFEKGFIFGVASSAYQVEGGRG---RGLNVWDSFTHRFPEKGGADLGNGDTTCDSYTLWQKDIDVMDELNS :  72
AtBGLU39   : FNRKHFDDDFIF---------EGGKG---RGLNVWDGFTHRYPEKGGPDLGNGDSTCGSYEHWQKDIDVMTELGV :  63
Os4bglu18  : IHRSDFPASFLFGTATSSYQIEGAYLEGNKSLSNWDVFTHLPG--NIKDGSNGDIADDHYHRYEEDVELMNSLGV :  73
Os4bglu14  : VDRSQFPPDFLFGTSSSAYQVEGGYLEGNKGLSNWDVFTHKQG--TIEDGSNGDTANDHYHRYMEDIELMHSLGV :  73
Os4bglu16  : LRRDDFPPGFLFGAATSAYQIEGAYLDDNKGLNNWDVFTHTQAG-RISDGRNGDVADDHYHRYTEDVDILHNLGV :  74
AtBGLU45   : VDSSPFPSDFLFGTASSAYQYEGAFLTDGKSLNNWDVFTHKNPG-KILDKNNADRAVDQYNRFLEDIQLMSFLGV :  74
AtBGLU46   : -DSSPFPSDFLFGTASSAFQYEGAFLTDGKGLNNWDVFAHENPG-KIVDGSNGDIATDQYHRYMEDIQSMNFLGV :  73
AtBGLU47   : EETFHFPKNFLFGTASSAYQYEGAYLTDGKTLSNWDVFTN-ISG-KIADGSHGKVAVDHYHRYPGDLDLMEDLGV :  73
Os9bglu33  : LTRHDFPEGFVFGAGSSAFQVEGAAAEDGRKPSIWDTFINQG---YMPDGSNADVSADQYHHYKEDVKLMYDMGL :  72
Os9bglu32  : LTRHDFPEGFVFGAGTSAFQVEGAAAEDGRKPSIWDTFTHQG---YSPGGAIADVSADQYHLYKEDVKLMYDMGL :  72
Os9bglu31  : ITRADFPPEFIFGAGSSAYQVEGAFAEDGRKPSIWDTFSHSG---YSVDGATGDVTADQYHKYKEDVKLLQDMGV :  72
Os1bglu2   : YTRSDFPRDFVFGAATSAYQYDGAAAEDGRSPTIWDTFAHEGQ--KTKDKGTGDVAADGYHKYKGDVKLMAETGL :  73
Os1bglu3   : YTRNDFPADFVFGAATSAYQYEGAAAEDGRGASIWDTFTHAG---KMKDKSTGDVASDGYHKYKGDVKLMTETGL :  72
Os1bglu5   : YTRGDFPEDFVFGSATSSYQYEGGFDEDGRSPSNWDIFTHQG---KMPGRSTADVAADGYHKYKDDLKLMVDTNL :  72
Os5bglu22  : FTRQDFPGEFVFGAGTSAYQYEGATDEDGRSPSIWDTFTHAG---KMPDKSTGDMGAGGYHKYKEDVKLMSDTSL :  72
Os5bglu23  : FTRQDFPGDFVFGAGTSAYQYEGATGEDGRTPSIWDTFTHSG---RMADNSTGDRAAAGYHKYKEDVKLMSDTGL :  72
Os5bglu20  : FRRDDFPDGFAFGAGTAAYQYEGAAAEDGRTPSIWDTYTHSG---RHPEDGTGDVASDGYHKYKEDVKLMTEIGL :  72
Os5bglu21  : FTRDDFPHDFAFGAGTSAYQYEGGAAEDGRTPSIWDTYTHSG---RHPEDETGDVASDGYHKYKEDVKLMSEIGL :  72
Os5bglu19  : FTRDDFPDGFTFGAGTAAFQYEGAAAEDGRTPSIWDTYAHSW---RNPGGETGDVACDGYHKYKEDVMLMNETGL :  72
AtBGLU1    : YSRSDFPEGFVFGAGISAYQWEGAVDEDGRKPSVWDTFLHCR---K-MDN--GDIACDGYHKYKEDVQLMAETGL :  69
AtBGLU3    : -DKNDFPEGFIFGSATSAYQWEGAFDEDGRKPSVWDTFLHTR---N-LSN--GDITSDGYHKYKEDVKLMVETGL :  68
AtBGLU4    : FSRSDYPEGFVFGAGTSAYQWEGAAAEDGRKPSLWDTLCHSR---D-QGN--GDIACDGYHKYKDDVKLMVDTNL :  69
AtBGLU5    : FSRSDFPEGFLFGAGTSAYQWEGAAAEDGRKPSVWDTLCYSR---N-IGN--GDVTCDGYHKYKEDVKLMVDTNL :  69
AtBGLU6    : FSRCDFPEGFVFGSSTSAYQWEGAVAEDGRKPSVWDRFCHSH---NNQGN--GDITCDGYHKYKEDVKLMVDTNL :  70
AtBGLU7    : FTRNDFPNDFLFGAATSAYQWEGAFDEDGKSPSVWDTTSHCD---SGSNN--GDIACDGYHKYKEDVMLMAEMGL :  70
AtBGLU8    : FTRNDFPEDFLFGAGTSAYQWEGAANEDGRTPSVWDTTSHCY---NGSN---GDIACDGYHKYKEDVKLMAEMGL :  69
AtBGLU9    : FTRNSFPKDFLFGAATSAYQWEGAVAEDGRTPSVWDTFSNSY---DTG---NGDVTSDGYHKYKEDVKLMATMGL :  69
AtBGLU10   : FTRNNFPKDFLFGAATSAYQWEGAVAEDGRTPSVWDTFSHTY---NRGNLGNGDITSDGYHKYKEDVKLMAEMGL :  72
AtBGLU11   : YSRNDFPPGFVFGSGTSAYQVEGAADEDGRTPSIWDVFAHAG---H-SGVAAGNVACDQYHKYKEDVKLMADMGL :  71
Osbglu39   : -MNTSLPANFLWGNSVSSMQTEGAWNEGGKGMSVYDIREAG------ENISDWKVATDSYHRYREDFDLMQDLGM :  68
Os11bglu36 : ADFRAFPDNFFFGLATARDQRDGGSQQSWRGENIGDGFEILAESGESAGGDNCSHNVAAWHRFDAELKLAKETGI :  75
                   p  f  g    a q eG            d                        h    d

                80         *       100         *       120         *       140         *
Os10bglu34 : DAYRFSIAWSRIFPNGTGEVNQAGIDHYNKLINALLAKGIEPYVTLYHWDLPQALEDKYTGWLDRQIINDYAVYA : 148
Os3bglu6   : DAYRFSIAWSRIYPNGVGQVNQAGIDHYNKLIDALLAKGIQPYVTLYHWDLPQALEDKYKGWLDRQIVDDFAAYA : 148
AtBGLU40   : DAYRFSISWTRIFPNGVGHINEAGIDHYNKLINALLAKGIEPYVTLYHWDLPQALHDRYLGWLNPQIINDFAAYA : 148
Os6bglu25  : DAYRFSISWSRIFPNGTGEPNEEGLSYYNSLIDALLDKGIEPYVTLFHWDLPQALEDRYGGWLNSEIIEDFVQYA : 148
AtBGLU41   : DAYRFSISWSRIFPNGTGEVNPDGVKYYNSLIDALLAKGIKPYVTLYHWDLPQALEDRYEGWLSREVVDDFEHYA : 149
Os1bglu4   : RAYRFSISWPRIFPDGLGNVNEQGVAFYNDLINFMIEKGIEPYATLYHWDLPHNLQQTVGGWLSDKIVEYFALYA : 147
AtBGLU42   : GAYRFSISWSRIFPDGLGEVNEEGIAFYNDLINTLLEKGIQPYVTLYHWDLPSHLQEAIGGWTNRKIVDYFGLYA : 148
Os1bglu1   : DAYRFSISWSRIFPTGTGKVNWKGVAYYNRLINYMLKIGITPYANLYHYDLPEALEVQYGGLLNRKIVEAFADYA : 148
Os7bglu26  : DAYRFSISWSRIFPNGTGMVNQEGVDYYNRLIDYMVKKGIKPYANLYHYDLPLALHEQYLGWLSPNIVEAFADYA : 148
AtBGLU43   : DAYRFSISWSRIFPEGSGKINSNGVAYYNRLIDYLIEKGITPYANLYHYDLPLALEQKYQGLLSKQ--GRFCGLR : 146
AtBGLU44   : DAYRFSISWSRIFPEGSGKVNWKGVAYYNRLIDYMVQKGITPYANLYHYDLPLALENKYKGLLGRQVVKDFADYA : 148
Os3bglu8   : DAYRFSISWSRIFPDGEGKVNTEGVAYYNNLIDYVIKQGLIPYVNLNHYDLPLALQKKYEGWLSPKIVGVFSDYA : 148
Os3bglu7   : DAYRFSISWSRIFPDGEGRVNQEGVDYYNNLINYLLQKGITPYVNLYHYDLPLALEKKYGGWLNAKMADLFTEYA : 148
Os12bglu38 : DAYRFSISWSRIFPSGLGKINKDGVDYYHRLIDYMLANNIIPYVVLYHYDLPQVLHDQYKGWLHPRIVRDFVRFA : 148
Os11bglu37 : DAFRFSIAWTRILPNGSLGINREGVAFYNSLINDVIAKGMIPFVTIFHWDTPQALESKYGGFLSENIVKDYVDFA : 148
Os11bglu35 : DAFRFSIAWTRILPNGSLGINREGVAFYNSLINDVIAKGMIPFVTIFHWDTPQALESKYGGFLSENIVKDYADFA : 148
Os9bglu30  : DAFRFSIAWTRILPNGSLGINKEGVAFYNSLINDVIAKGMIPFVTIFHWDTPLALESKYGGFLSEDIVKEYVDFA : 148
Os9bglu29  : DAFRFSISWSRILPNGTLGVNKEGVAFYNNLINEIIAKGMKPFVTIFHWDTPQALESKYGGFLSENIIKDYVDFA : 148
Os8bglu28  : DSFRFSIAWSRILPNGTIGINKEGVEFYNSLINEVIAKGLKPFVTIFHFDTPQALEDKYGGFLSENIVKDYVDYA : 146
Os8bglu27  : DAFRFSIAWSRILPNGTIGINKEGIAFYNSLINEVISRGLKPFVTIFHFDTPQALEDKYRSFLSENIVKDFVDYA : 148
Os4bglu10  : DAYRFSISWSRILPNGSLGVNREGINYYNNLINELLSKEVQPFATLFHFDTPQALEDKYKGFLSPNIINDYKDYA : 149
Os4bglu11  : DAYRFSISWSRILPS-ALGVNREGISYYNNLINELLSKGVQPFVTLFHWDSPQALEDKYKGFLSPNIINDYKEYA : 148
Os4bglu9   : DAYRFSISWSRILPNGSLGVNIEGINYYNNLINELLLKGVQSFVTLFHYDTPQALEDKYNGFLSPNIINDYKDYA : 149
Os4bglu13  : DAYRFSISWTRILPNGSLGINREGISYYNNLINELLLKGVQPFVTLFHWDSPQALEDKYNGFLSPNIINDYKEYA : 149
Os4bglu12  : DAYRFSISWTRILPNGSLGVNKEGIKYYNNLINELLSKGVQPFITLFHWDSPQALEDKYNGFLSPNIINDFKDYA : 149
Os6bglu24  : NAYRFSVSWPRILPNGKLGVNLEGIKYYNNLIDELISKGVEPFVTLFHWDSPQALEQQYGGFLSNLIVEDFRDYA : 148
AtBGLU17   : DSFRFSISWSRILPRGTVGVNQAGINFYNHLINELISNGIRPLVTLFHWDTPQALEDEYGGFLNPQIVKDFVEYV : 149
AtBGLU12   : DAYRFSISWSRILPRENLGINQAGIDYYNNLINELLSKGIKPFATIFHWDTPQSLEDAYGGFLGAEIVNDFRDYA : 149
AtBGLU13   : GAYRFSISWSRILPRGNLGINQAGIDYYNNLINELLSKGIKPFATIFHWDTPQSLEDAYGGFFGAEIVNDFRDYA : 149
AtBGLU14   : NAYRFSISWSRILPRGNLGINQAGIDYYNNLINELLSKGIKPFATIFHWDTPQDLEDAYGGFRGAEIVNDFRDYA : 149
AtBGLU15   : NAYRFSISWSRILPRGNLGINQAGIDYYNNLINELLSKGIKPFATMFHWDTPQALEDAYGGFRGAEIVNDFRDYA : 149
AtBGLU16   : DAYRFSISWSRILPRGTLGINQAGIEYYNNLINQLISKGVKPFVTLFHWDLPDALENAYGGLLGDEFVNDFRDYA :  99
AtBGLU18   : DAFRLSIAWPRIFPHGRMGINKVGVQFYHDLIDELLKNNIIPLVTVFHWDTPQDLEDEYGGFLSGRIVQDFTEYA : 148
AtBGLU19   : DGFRLSISWPRIFPHGRMGISKEGVQFYHDLIDELLKNDITPLVTVFHWDTPADLEDEYGGFLSERIVPDFVEYA : 148
AtBGLU20   : DGFRFSIAWPRIFPHGRMGISKAGVQYYHDLIDELLANGITPLVTVFHWDTPQDLEDEYGGFLSDRIIKDFTEYA : 148
AtBGLU21   : DAFRLSIAWSRIFPHGRKGVSQAGVQFYHELIDELLKN--VPFVTVFHWDTPQDLEDEYGGFLSQNIVKDFREYA : 146
AtBGLU22   : DAFRLSIAWSRIFPHGRKGVSQAGVKFYHDLIDELLKNGIIPFVTVFHWDTPQDLEDEYGGFLSENIVKDFREYA : 148
AtBGLU23   : DAFRMSIAWPRIFPHGRKGVSQAGVQFYHDLIDELIKNGITPFVTVFHWDTPQDLEDEYGGFLSERIVKDFREYA : 148
AtBGLU24   : DSFRLSISWTRIFPHGREGVSKSGVQFYHDLIDELKRNGIIPFVTVFHWDTPQTLENEYGGFLSAHIVKDFREYA : 148
AtBGLU25   : DAFRFSISWSRIFPHGKKGVSETGVKFYNDLINELIANGVTPLVTLFQWDVPQALEDEYGGFLSDRILEDFRDFA : 149
AtBGLU26   : DSFRLSIAWPRVLPYGKRGVSEEGIKFYNDVIDELLANEITPLVTIFHWDIPQDLEDEYGGFLSEQIIDDFRDYA : 149
AtBGLU27   : DAFRFSISWPRIFPLGKKGVNKEGIQFYNDLIDELLANGITPLATLFHWDTPQALEDEYSGFLSEEAVDDFKDFA : 148
AtBGLU28   : DAFRFSISWARLIPSGKVGVNKEGVEFYKALIDELVANGIEPSMTLYHWDHPQSLEDEYGGFLSPQIVEDFRDFS : 148
AtBGLU29   : DAFRFSISWARLIPSGKVGVNKEGVQFYKALIDELIANGIQPSVTLYHWDHPQALEDEYGGFLNPQIIEDFRNFA : 148
AtBGLU30   : DAFRFSISWSRLIPSGKLGVNKEGVQFYKDLIDELLANDIQPSMTLYHWDHPQSLEDEYGGFLSPKIVEDFRDFA : 148
AtBGLU31   : DSFRFSLSWSRILPSGKLGVNKEGVQFYKNLIDELIENGIKPFVTIYHWDIPQALDDEYGSFLSPRIIDDFRNYA : 148
AtBGLU32   : DSFRFSLSWSRILPSGKLGVNKEGVQFYKNLIDELIKNGIKPFVTIYHWDIPQALDDEYGSFLSPRIIDDFRNFA : 148
AtBGLU33   : NGFRFSISWTRILPYGTIGVNEEGVKFYNDLINELLANGIQPSVTLFHWESPLALEMEYGGFLNERIVEDFREFA : 149
AtBGLU34   : QAYRLSIAWSRVLPKGRLGVDENGITYYNNLINELKANGIEPYVTIFHWDVPQTLEDEYGGFLSTRIVEDYTNYA : 146
AtBGLU35   : QAYRLSIAWSRVLPKGRLGVDENGITYYNNLINELKANGIEPYVTIFHWDVPQTLEDEYGGFLSTRIVEDYTNYA : 146
AtBGLU36   : QAYRFSIAWSRVLPKGRLGVDENGITYYNNLINELKANGIEPFVTIFHWDVPQDFRRRIWRLLKP-TYSDFKNYA : 141
AtBGLU37   : KGYRFSFAWSRILPKGKRGINEDGINYYSGLIDGLIARNITPFVTLFHWDLPQSLQDEYEGFLDRTIIDDFKDYA : 147
AtBGLU38   : TGYRFSIAWSRLLPKGKRGVNPGAIKYYNGLIDGLVAKNMTPFVTLFHWDLPQTLQDEYNGFLNKTIVDDFKDYA : 147
AtBGLU39   : DGYRFSLAWSRIAPR--E--NQAGVKYYNDLIDGLLAKNITPFVTLFHWDLPQVLQDEYEGFLNHEIIDDFKDYA : 134
Os4bglu18  : NAYRFSISWSRILPKGRFGVNPAGIDFYNKLIDSILLKGIQPFVTLTHYDIPQELEDRYGAWLNAEIQSDFGHFA : 148
Os4bglu14  : NSYRFSISWARILPKGRFDVNPDGVAFYNALIDGLVQKGIQPFVTICHYDIPHELDERYGGWLSPEIQKDFSYFA : 148
Os4bglu16  : NSYRFSISWARILPRGRLGVNSAGIAFYNRLINALLQKGIQPFVTLNHFDIPHELETRYGGWLGAAIREEFEYYS : 149
AtBGLU45   : NSYRFSISWCRILPRGRFEINYLGIKYYNIFIDALISRGIKPFVTLNHVDYPQELEDRFQSWLNPEMQKEFGYLA : 149
AtBGLU46   : NSYRLSISWSRVLPNGRFVINYKGIKYYNNLIDALIKKGITPFVTLNHFDYPQELENRFKSWLSSEMQKDFGYLA : 148
AtBGLU47   : NSYRLSLSWARILPKGRFDVNMGGIDHYNRMINDILKTGIEPFVTLTHYDIPQELEYRYGSWLNPQIREDFEHYA : 148
Os9bglu33  : DAYRFSIAWPRLIPDGRGEINPKGLEYYNNLIDELIMHGIQPHVTIYHFDLPQALQDEYGGILSPRFIEDYSAYA : 147
Os9bglu32  : DAYRFSIAWPRLIPDGRGEINPKGLEYYNNLIDELIMHGIQPHVTIYHFDLPQALQDEYGGILSPRFIEDYTAYA : 147
Os9bglu31  : DAYRMSISWSRLIPDGRGAVNPKGLEYYNNLIDELLSHGIQPHVTIYHFDFPQALQDEYNGILSPRFVEDFTAYA : 147
Os1bglu2   : EAYKFSISWSRLIPNGRGAVNQEGLKYYNNVIDELAKRGIQPHIMLCHLDLPQALEDEYDGWLSPRIVDDFTAYA : 148
Os1bglu3   : EAYRFSISWSRLIPSGRGAVNQQGLKYYNNIIDELTKRGIQVHVMLYHLDLPQALEDEYAGWLSPRIVEDFTAYA : 147
Os1bglu5   : EAYRLSISWSRIIPNGRGDVNPKGLQYYNDIIDGLVKNGIQVHIMLYQLDLPQVLEDEYDGWLSPRILEDFKAYA : 147
Os5bglu22  : EAYRFSISWSRLIPRGRGPVNPKGLEYYNSLIDELVERGIEIHVTLYHLDFPQILEDEYHGWLSPRVIDDFTAYA : 147
Os5bglu23  : EAYRFSISWSRLIPRGRGPINPKGLEYYNDLIDKLVKRGIEIHVTLYHLDFPQALQDEYNGWLSPRIIEDFTAYA : 147
Os5bglu20  : EAYRFTISWSRLIPSGRGAVNPKGLQFYNNMINELVKAGIQIQVVLYHSDLPQSLQDEYGGWINPKIVDDFTAYA : 147
Os5bglu21  : EAYRFTISWSRLIPSGRGAVNLKALQFYNSMINELVKAGIQIHVVMYHMDLPQSLQDEYGGWISPKIVDDFTAYA : 147
Os5bglu19  : EAYRFTISWSRLIPSGRGAVNPKGLQFYNSMINELVKAGIQIHAVLYHIDLPQSLQDEYGGWVSPKVVDDFAAYA : 147
AtBGLU1    : HTFRFSISWSRLISNGRGSINPKGLQFYKNFIQELVKHGIEPHVTLHHYDFPQYLEDDYGGWTNRKIIKDFTAYA : 144
AtBGLU3    : DAFRFSISWSRLIPNGRGPVNPKGLQFYKNFIQELVSHGIEPHVTLFHYDHPQYLEDEYGGWINRRIIQDFTAYA : 143
AtBGLU4    : DAFRFSISWSRLIPNGRGPVNQKGLQFYKNLIQELVSHGIEPHVTLYHYDHPQSLEDEYGGWLNHRMIKDFTTYA : 144
AtBGLU5    : DAFRFSISWSRLIPNGRGSVNQKGLQFYKNLISELITHGIEPHVTLYHYDHPQYLEDEYGGWVNNMMIKDFTAYV : 144
AtBGLU6    : DAFRFSISWSRLIPNRRGPVNQKGLQFYKNLIQELVNHGIEPYVTLHHFDHPQYLEDEYEGWLNHMIVEDFTAYA : 145
AtBGLU7    : ESFRFSISWSRLIPNGRGRINPKGLLFYKNLIKELRSHGIEPQVTLYHYDLPQSLEDEYGGWINRKIIEDFTAFA : 145
AtBGLU8    : ESFRFSISWSRLIPNGRGRINPKGLLFYKNLIKELRSHGIEPHVTLYHYDLPQSLEDEYGGWINHKIIEDFTAFA : 144
AtBGLU9    : ESFRFSISWSRLIPNGRGLINPKGLLFYNNLIKDLKSHGIEPHVTLYHYDLPQSLEDEYGGWINRKIIEDFTAYA : 144
AtBGLU10   : ESFRFSISWSRLIPNGRGLINPKGLLFYKNLIKELISHGIEPHVTLYHYDLPQSLEDEYGGWINRKIIEDFTAYA : 147
AtBGLU11   : EAYRFSISWSRLLPSGRGPINPKGLQYYNNLIDELITHGIQPHVTLHHFDLPQALEDEYGGWLSQEIVRDFTAYA : 146
Osbglu39   : NCYRFQISWSRVCPQGDGAFNDEGIAFYDRFIDDLIARGIEPMICLYHFDMPLALAQEYNGFNDRRVMEAFIRYG : 143
Os11bglu36 : SVFRMGVDWARLMPEEELSVNFAALERYRWIIQRVREYGMKVMLTLFHHSLP-PWAGKYGGWKMEKTVTYFMDFV : 149
               54f   W R6 p g       g6  Y   I  6   g  p  t6 h d P  l     g        d5   a

                    160         *       180         *       200         *       220
Os10bglu34 : ETCFQAFGDRVKHWITFNEPHTVAVQAYDSG-MHAPGRCSVLLH------LYCKK--GNSGTEPYIVAHNMILSH : 214
Os3bglu6   : ETCFREFGDRVKHWITLNEPHTVAIQGYDAG-LQAPGRCSVLLH------LYCKA--GNSGTEPYVVAHHFILAH : 214
AtBGLU40   : EVCFQRFGDRVKHWITFNEPHTFAIQGYDVG-LQAPGRCTILFK------LTCRE--GNSSTEPYIVGHNVILTH : 214
Os6bglu25  : FTCFKEFGDRVKHWITFNEPYNFAIDGYDLG-IQAPGRCSILSH------VFCRE--GKSSTEPYIVAHNILLAH : 214
AtBGLU41   : FTCFKAFGDRVKYWITFNEPHGVSIQGYDTG-IQAPGRCSLLGH------WFCKK--GKSSVEPYIVAHNILLSH : 215
Os1bglu4   : EACFANFGDRVKHWITINEPLQTAVNGYGIG-HFAPGGC------------------EGETARCYLAAHYQILAH : 203
AtBGLU42   : DACFANFGDRVKHWITLNEPLQTSVNGHCIG-IFAPGRN------------------EKPLIEPYLVSHHQVLAH : 204
Os1bglu1   : EFCFKTFGDRVKNWMTFNEPRVVAALGYDDG-NFAPGRCT----------KCTAG---NSATEPYIVAHHLILSH : 209
Os7bglu26  : DFCFQTFGDRVKDWFTFNEPRCVAALGYDNG-FHAPGRCS----------GCDAG--GNSTTEPYLAAHHLILSH : 210
AtBGLU43   : RVLFQTFGDRVKNWMTFNEPRVVAALGYDNG-IFAPGRCSEAFG------NCTDG---NSATEPYIVAHHLILAH : 211
AtBGLU44   : EFCYKTFGDRVKNWMTFNEPRVVAALGYDNG-IFAPGRCSKAFG------NCTEG---NSATEPYIVTHHLILAH : 213
Os3bglu8   : EFCFKTYGDRVKNWFTFNEPRIVAALGHDTG-TDPPNRCT----------KCAAG--GNSATEPYIVAHNIILSH : 210
Os3bglu7   : DFCFKTFGNRVKHWFTFNEPRIVALLGYDQG-TNPPKRCT----------KCAAG--GNSATEPYIVAHNFLLSH : 210
Os12bglu38 : DFCFKTYGHKVKNWFTINEPRMMANHGYGDG-FFPPGRCT----------GCQPG--GNSATEPYIAAHNLLLSH : 210
Os11bglu37 : EVCFREFGDRVKYWTTFNEPFTYNAYGYGKG-VFAPGRCSPYVS-----KSCGAG---DSSREPYLVTHHIHLSH : 214
Os11bglu35 : EVCFHEFGDRVKYWTTFNEPFTYSAYGYGGG-VFASGRCAPYVS-----KSCGAG---DSSREPYLVTHHIHLSH : 214
Os9bglu30  : EVCFREFGDRVKYWFTFNEPFTYSAYGYGKG-VFAPGRCSSYVS-----KSCGVG---DSSREPYLVAHHIHLSH : 214
Os9bglu29  : EVCFREFGDRVKFWATFNEPWTYCSQGYGTG-IHALGRCSPYVS-----TSCAGG---DSSREPYLAAHHVILAH : 214
Os8bglu28  : DLCFSLFGDRVKLWNTFNEPTIFCMNGYATG-IMAPGRCSPYAS-----ASCAAGG--DSGREPYVAGHHLLVAH : 213
Os8bglu27  : DVCFREFGDRVKSWNTFNEPMIFCAGGYGSG-TKAPGRCSPYVS-----KKCAPG---DSGNEPYVAGHNLLLAH : 214
Os4bglu10  : EICFKEFGDRVKHWITFNEPWNFCSMGYASG-TMAPGRCSSWEK-----GKCRVG---DSGREPYTACHHQLLAH : 215
Os4bglu11  : ETCFKEFGDRVKHWITFNEPWTFCSMGYASG-IMAPGRCSSWEV-----GKCRVG---DSGREPYTACHHQLLAH : 214
Os4bglu9   : EICFKEFGDRVKHWITFNEPWIFCSKAYASG-TYAPGRCSPWEM-----GKCSVG---DSGREPYTACHHQLLAH : 215
Os4bglu13  : ETCFKEFGDRVKHWITFNEPLSFCVAGYASGGMFAPGRCSPWE------GNCSAG---DSGREPYTACHHQLLAH : 215
Os4bglu12  : EICFKEFGDRVKNWITFNEPWTFCSNGYATG-LFAPGRCSPWEK-----GNCSVG---DSGREPYTACHHQLLAH : 215
Os6bglu24  : DICFREFGDRVKYWITFNEPWSFSIGGYSNG-ILAPGRCSSQGK-----SGCSKG---DSGREPYIVAHNQLLAH : 214
AtBGLU17   : DICFKEFGDRVKEWITINEPNMFAVLGYNVG-NIAPGRCSSYV------QNCTVG---NSATEPYLVAHYLILSH : 214
AtBGLU12   : DICFKNFGDRVKHWMTLNEPLTVVQQGYVAG-VMAPGRCSKFTN-----PNCTAG---NGATEPYIVGHNLILAH : 215
AtBGLU13   : DICFKNFGDRVKHWMTLNEPLTVVQQGYVAG-VMAPGRCSKFTN-----PNCTAG---NGATEPYIVGHNLILAH : 215
AtBGLU14   : DICFKSFGDRVKHWITLNEPLTVVQQGYVAG-VMAPGRCSKFTN-----PNCTAG---NGATEPYIVGHNLILAH : 215
AtBGLU15   : DICFKNFGDRVKHWMTLNEPLTVVQQGYVAG-VMAPGRCSKFTN-----PNCTDG---NGATEPYIVGHNLILSH : 215
AtBGLU16   : ELCFQKFGDRVKQWTTLNEPYTMVHEGYITG-QKAPGRCSNFYK-----PDCLGG---DAATEPYIVGHNLLLAH : 165
AtBGLU18   : NFTFHEYGHKVKHWITFNEPWVFSRAGYDNG-KKAPGRCSPYIPGYG--QHCQD---GRSGYEAYQVSHNLLLSH : 217
AtBGLU19   : NFTFHEYGDKVKNWITFNEPWVFSRSGYDVG-KKAPGRCSPYVKEFG--KLCQD---GRSGFEPYVVSHNLLVGH : 217
AtBGLU20   : NFTFQEYGDKVKHWITFNEPWVFSRAGYDIG-NKAPGRCSKYIKEHG--EMCHD---GRSGHEAYIVSHNMLLAH : 217
AtBGLU21   : DYVFTEYGGKVKNWITFNEPWVFAHAGYDLG-KKAPGRCSRYVPG------CEDR-EGQSGKEAYLVSHNLLNAH : 213
AtBGLU22   : DYVFTEYGGKVKNWITFNEPWVFAHAGYDVG-KKAPGRCSRYLKG------CEDR-DGRSGYEAYLVSHNLLNAH : 215
AtBGLU23   : DFVFQEYGGKVKHWITFNEPWVFSHAGYDVG-KKAPGRCSSYVNAK-----CQD---GRSGYEAYLVTHNLLISH : 214
AtBGLU24   : EFVFKEYGGKVKHWITFNEPWVFAHAGYDVG-KKAPGRCSPYAKDETVKGDCLG---GRSGYEAYLVSHNLLNAH : 219
AtBGLU25   : QFAFNKYGDRVKHWVTINEPYEFSRGGYETG-EKAPGRCSKYVNEK-----CVA---GKSGHEVYTVSHNLLLAH : 215
AtBGLU26   : SLCFERFGDRVSLWCTMNEPWVYSVAGYDTG-RKAPGRCSKYVNG-----ASVA---GMSGYEAYIVSHNMLLAH : 215
AtBGLU27   : ALCFEEFGDRVKLWVTLNEPWVYSIGGYDTG-RKAPGRASKYMNE-----AAVA---GESGLEVYTVSHNLLLAH : 214
AtBGLU28   : RVCFEEFGDKVKMWTTINEPYVITVAGYDTG-NKAVGRCSKWVNS-----KCQG---GDSGTEPYIASHHLLLAH : 214
AtBGLU29   : RVCFENFGDKVKMWTTINEPYVISVAGYDTG-IKAVGRCSKWVNS-----RCQA---GDSAIEPYIVSHHLLLSH : 214
AtBGLU30   : RICFEEFGDKVKMWTTINEPYIMTVAGYDQG-NKAAGRCSKWVNE-----KCQA---GDSSTEPYIVSHHTLLAH : 214
AtBGLU31   : RFCFQEFGDKVSMWTTFNEPYVYSVSGYDAG-NKAMGRCSKWVNS-----LCIA---GDSGTEPYLVSHHLLLAH : 214
AtBGLU32   : RFCFQEFGDKVSMWTTFNEPYVYSVSGYDAG-NKAIGRCSKWVNS-----LCIA---GDSGTEPYLVSHNLLLAH : 214
AtBGLU33   : NFCFKEFGDRVKNWATFNEPSVYSVAGYSKG-KKAPGRCSKWQAP-----KCPT---GDSSEEPYIVAHNQILAH : 215
AtBGLU34   : ELLFQRFGDRVKFWITLNQPFSLATKGYGDG-SYPPGRCTG---------CELG---GDSGVEPYTVAHNQLLAH : 208
AtBGLU35   : ELLFQRFGDRVKFWITLNQPLSLALKGYGNG-SYPPGRCTG---------CELG---GDSGVEPYTVAHNQLLAH : 208
AtBGLU36   : ELLFQRFGDRVKFWITLNQPYSLAVKGYGDG-QYPPGRCTD---------CEFG---GDSGTEPYIVGHHELLAH : 203
AtBGLU37   : DLCFERFGDRVKHWITINQLFTVPTRGYALG-TDAPGRCSQWVDK-----RCYG---GDSSTEPYIVAHNQLLAH : 213
AtBGLU38   : DLCFELFGDRVKNWITINQLYTVPTRGYALG-TDAPGRCSPKIDV-----RCPG---GNSSTEPYIVAHNQLLAH : 213
AtBGLU39   : NLCFKIFGDRVKKWITINQLYTVPTRGYAMG-TDAP--------------------------EPYIVAHNQLLAH : 182
Os4bglu18  : DVCFGAFGDRVKYWTTFNEPNVAVRHGYMLG-TYPPSRCSPPFG------HCARGG--DSHAEPYVAAHNVILSH : 214
Os4bglu14  : EVCFKLFGDRIKFWTTFNQPNLSIKFSYMDG-FYSPGRCSEPFG------KCALG---NSSIEPYVAGHNIILSH : 213
Os4bglu16  : DVCFNAFGDRVRFWTTFNEPNLSTRHQYILG-EFPPNHCSPPFG------NCSSG---DSRREPYAAAHNILLSH : 214
AtBGLU45   : DICFKHFGNRVKYWTTLNEPNQQLILGYLTG-KFPPSRCSSPYG------NCSQG---NSETEPFIAAHNMILAH : 214
AtBGLU46   : DICFKHFGDRVKHWITINEPNQHISLAYRSG-LFPPARCSMPYG------NCTHG---NSETEPFIAAHNMILAH : 213
AtBGLU47   : NICFRHFGDRVKFWSTFNEPNVQVILGYRTG-TYPPSRCSKPFG------NCSCG---DSYIEPLVAAHNIILSH : 213
Os9bglu33  : EVCFKNFGDRVKHWATFNQPNIEPIGGFDAG-DRPPRRCSYPFG-----TNC---TGGDSSTEPYIVAHHLLLAH : 213
Os9bglu32  : EVCFKNFGDRVKHWVTVNEPNIEPIGGYDAG-VQPPRRCSYPFG-----TNC---TGGDSSTEPYIVAHHLLLAH : 213
Os9bglu31  : DVCFKNFGDRVKHWSTVNEPNIEPIGGYDQG-ILPPRRCSFPFGV----LSC---DNGNSTTEPYIVAHHLLLAH : 214
Os1bglu2   : DVCFREFGDRVLHWTTLAEPNIAALGGYDTG-VLSPGHCSDPFGL----TEC---TVGNSTVEPYIAAHNMILTH : 215
Os1bglu3   : DVCFREFGDRVSHWTILAEPNVAALGGYDTG-EFAPGRCSDPFGV----TKC---TVGNSSVEPYVAAHNMILTH : 214
Os1bglu5   : DVCFKEFGDRVAHWITIDEPNVASIGSYDSG-QLAPGRCSDPFGI----RKC---TVGNSSVEPYIAVHNMLLAH : 214
Os5bglu22  : DVCFREFGDRVRHWTTMDEPNVLSIAAYDSG-AFPPCRCSPPFGA----N-C---TAGNSTVEPYVVAHNSILAH : 213
Os5bglu23  : DVCFREFGDLVRHWTTVGEPNVLSIAGYDSG-VIPPCRCSPPFGT----S-C---AAGDSTVEPYVAAHNSILAH : 213
Os5bglu20  : DVCFREFGDRVAHWTTVLEPNVMAQGCYDTG-TLPPNHCSYPFG-----SNC---TGGNSTVEPYLFIHHNLLAH : 213
Os5bglu21  : DVCFREFGDRVVHWTTVLEPNAMAQAGYDMG-ILPPNRCSYPFG-----SNC---TAGNSSVEPYLFIHHSLLAH : 213
Os5bglu19  : DVCFREFGDRVAHWTTSIEPNVMAQSGYDDG-YLPPNRCSYPFGR----SNC---TLGNSTVEPYLFIHHTLLAH : 214
AtBGLU1    : DVCFREFGNHVKFWTTINEANIFTIGGYNDG-NSPPGRCSFPGR------NC---TLGNSSTETYIVGHNLLLAH : 209
AtBGLU3    : NVCFREFGHHVKFWTTINEANIFTIGGYNDG-ITPPGRCSSPGR------NC---SSGNSSTEPYIVGHNLLLAH : 208
AtBGLU4    : DVCFREFGNHVKLWTTINEANIFSIGGYNDG-DTPPGRCSKPSK------NC---SSGNSSIEPYIVGHNLLLAH : 209
AtBGLU5    : DVCFREFGNYVKFWTTVNEANVFTIGGYNDG-DTPPGRCSLPGK------NC---LLGNSSTETYIVGHNLLLAH : 209
AtBGLU6    : DVCFREFGNHVKFWTTINEGNIFSIGGYNDG-DSPPGRCSIPGQ------NC---LLGNSSTEPYIVGHNLLLAH : 210
AtBGLU7    : DVCFREFGEDVKLWTKINEATLFAIGSYGD--GMRYGHCP-P-------MNY---STANVCTETYIAGHNMLLAH : 207
AtBGLU8    : DVCFREFGEDVKLWTTINEATIFAFAFYGK--DVRYGNC----------------TTGNYCMETYIAGHNMLLAH : 201
AtBGLU9    : DVCFREFGEDVKLWTTINEATIFAIGSYDQG-TAPPGHCS-PNKF----VNC---STGNSSTEPYIAGHNILLAH : 210
AtBGLU10   : DVCFREFGEDVKLWTTINEATIFAIGSYDQG-ISPPGHCS-PNKF----INC---TSGNSSTEPYLAGHNILLAH : 213
AtBGLU11   : DTCFKEFGDRVSHWTTINEVNVFALGGYDQG-ITPPARCSPPFG-----LNC---TKGNSSIEPYIAVHNMLLAH : 212
Osbglu39   : KKMIDCFGDRVKYWLTFNEQNIFHMPEAFR----ISGYLKGE----------------QTLRELYELQHHAMVAH : 198
Os11bglu36 : RLVVDCVSNLVDYWVIFNEPHVFVMLTYCAG--AWPGGDP---N------AIEVATSTLPTGVYNQALHWMAIAH : 213
               c    g  6  W t  2p      g   g    pgrc            c          ep    H     H

                 *       240         *       260         *       280         *       300
Os10bglu34 : ATVSDIYRKKYKASQNGELGISFDVIWYEPMSNSTADIEAAKRAQEFQLGWFADPFFFGDY-PATMRSRVGS-RL : 287
Os3bglu6   : AAAASIYRTKYKATQNGQLGIAFDVMWFEPMSNTTIDIEAAKRAQEFQLGWFADPFFFGDY-PATMRARVGE-RL : 287
AtBGLU40   : ATVSDIYRKKYKAKQGGSLGIAFDVMWFEPESNKTEDIEAAQRAQDFQLGWFLDPLMFGDY-PSSMRSRVGS-RL : 287
Os6bglu25  : AGAFRAYEQHFKNEQGGLIGIALNSRWYEPFSNADEDTEAAARAMDFELGWFLDPLMFGHY-PPSMQKLAGD-RL : 287
AtBGLU41   : AAAYHTYQRNFKEKQRGQIGISLDAKWYEPMSDCDEDKDAARRAMDFGLGWFMDPLINGDY-PASMKSLVEE-RL : 288
Os1bglu4   : AAAVDVYRRKFKAVQGGEVGLVVDCEWAEPFSEKTEDQVAAERRLDFQLGWYLDPIYFGDY-PESMRQRLGD-DL : 276
AtBGLU42   : ATAVSIYRSKYKESQGGQIGLSVDCEWAEPNSEKPEDKVAADRRIDFQLGWFLDPLFFGDY-PASMRQKLGD-NL : 277
Os1bglu1   : ASAVQRYRHKYQHIQKGKIGILLDFVWYEGLTNSTADQAAAQRSRDFHVGWFLHPIIYGEY-PKSLQVIVKE-RL : 282
Os7bglu26  : AAAVKRYREKYQLYQKGRIGILLDFVWYEPFSDSNADRAAAQRARDFHLGWFLDPIIHGRY-PYSMLEIVKD-RM : 283
AtBGLU43   : AAAVQRYRQNYQEKQKGRVGILLDFVWFEPLTSSQADNDAAQRARDFHVGWFIHPIVYGEY-PNTLQNIVKE-RL : 284
AtBGLU44   : AAAVQRYRKYYQAKQKGRVGILLDFVWYEPLTRSKADNLAAQRARDFHIGWFIHPLVYGEY-PKTMQNIVKE-RL : 286
Os3bglu8   : ATAVDRYRNKFQASQKGKIGIVLDFNWYEPLTNSTEDQAAAQRARDFHVGWFLDPLINGQY-PKNMRDIVKE-RL : 283
Os3bglu7   : AAAVARYRTKYQAAQQGKVGIVLDFNWYEALSNSTEDQAAAQRARDFHIGWYLDPLINGHY-SQIMQDLVKD-RL : 283
Os12bglu38 : AAAVRTYRDKYQAIQKGKIGILLDFVWYEPLTDKEEDHAAAHRAREFTLGWYLHPITYGHY-PETMQNAVKE-RL : 283
Os11bglu37 : AAADLPY----QPTQKGQIGMVVMTHWFVPYDDTVAHRGAVQRSLDFMFGWFMDPLVHGDY-PGTMRGWLGD-RL : 283
Os11bglu35 : AAVVHLYRTRYQPTQKGQIGMVVVTHWFVPYDDTAADRGAVQRSLDFMFGWFMDPLVHGDY-PGTMRGWLGD-RL : 287
Os9bglu30  : AAAVQLYRTKYQPTQKGQIGMVVVTHWFVPYDNSDADRGAVQRSLDFIYGWFMDPIVHGDY-PGTMRGWLGN-RL : 287
Os9bglu29  : ATAVHLYRTKYQPTQHGQIGITAVSHWFVPYNDTAADRRVVQRSLDFMYGWFLDPIVHGDY-PGTMRGWLGA-RL : 287
Os8bglu28  : AEAVRLYRARYRAAHGGEVGITQVSHWFEPYDASAADRRARRRALDFMLGWFMHPVAHGEY-PPAMRRLVGG-RL : 286
Os8bglu27  : AEAVRLYRQKYQATQKGQIGITQVSHWFVPYSDAAADKHAVRRSLDFMYGWFMDPIVFGDY-PGTMRKLVGD-RL : 287
Os4bglu10  : AETVRLYKEKYQFLQKGRIGIILNSEWFVPFSQSKSSNDAARRVLDFMLGWFMDPLIRGDY-PLSMRELVGN-RL : 288
Os4bglu11  : AETVRLYKEKYQ-LQKGKIGIILNADWFVPLSQSKSSSDAARRALDFMLGWFMDPLIRGDY-PLSMRELVGN-RL : 286
Os4bglu9   : AETVRLYREKYQ-LQKGKIGIIVNSQWYVPFSQSKTNKDAARRVLDFVLGWLMDPLIRGDY-PLNMRELVGN-RL : 287
Os4bglu13  : AETVRLYKEKYQ-LQKGKIGITLVSNWFVPFSRSKSNIDAARRALDFMLGWFMDPLIRGEY-PLSMRELVRN-RL : 287
Os4bglu12  : AETVRLYKAKYQ-LQKGKIGITLVSHWFVPFSRSKSNNDAAKRAIDFMFGWFMDPLIRGDY-PLSMRGLVGN-RL : 287
Os6bglu24  : AAVVQIYREKYQ-GQKGKIGIAIVSNWMIPYEDSKEDKHATKRALDFMYGWFMDPLTKGDY-PVSMRTLVGN-RL : 286
AtBGLU17   : AATVQLYREKYQSFHGGTIGMTIQTYWMIPKYNTPACREAAKRALDFFFGWFADPITYGDY-PKTMRELVGN-RL : 287
AtBGLU12   : GEAVKVYREKYKASQKGQVGIALNAGWNLPYSESAEDRLAAARAMAFTFDYFMEPLVTGKY-PIDMVNYVKGGRL : 289
AtBGLU13   : GEAVKVYREKYKASQKGQVGIALNAGWNLPYTESAEDRLAAARAMAFTFDYFMEPLVTGKY-PVDMVNNVKDGRL : 289
AtBGLU14   : GEAIKVYRKKYKASQKGQVGIALNAGWNLPYTESAEDRLAAARAMAFTFDYFMEPLVTGKY-PVDMVNNVKGGRL : 289
AtBGLU15   : GAAVQVYREKYKASQQGQVGIALNAGWNLPYTESPKDRLAAARAMAFTFDYFMEPLVTGKY-PVDMVNNVKG-RL : 288
AtBGLU16   : GVAVKVYREKYQATQKGEIGIALNTAWHYPYSDSYADRLAATRATAFTFDYFMEPIVYGRY-PIEMVSHVKDGRL : 239
AtBGLU18   : AYAVDAFRNCKQCA-GGKIGIAHSPAWFEPQDL-EHVGGSIERVLDFILGWHLAPTTYGDY-PQSMKDRVGH-RL : 288
AtBGLU19   : AEAVDAFRKCEKCK-GGKIGIAHSPAWFEPEDV-EGGQATVNRVLDFVIGWHLDPTTFGDY-PQSMKDAVGS-RL : 288
AtBGLU20   : ADAVDAFRKCDKCK-GGKIGIAHSPAWFEAHELDEEHETPVTGLIDFILGWHLHPTTYGDY-PQSMKDHIGH-RL : 289
AtBGLU21   : AEAVEVFR--QKVK-GGKIGIAHSPAWFEPHDLDSNDAPTVSRVLDFMLGWHLEPTTSGDY-PQIMKDLLGY-RL : 283
AtBGLU22   : AEAVEVFR--QKVK-GGKIGIAHSPAWFEPHDLDSNDVPTVSRVLDFMLGWHLDPTTFGDY-PQIMKDLLGH-RL : 285
AtBGLU23   : AEAVEAYRKCEKCK-GGKIGIAHSPAWFEAHDLDSQDGASIDRALDFILGWHLDTTTFGDY-PQIMKDIVGH-RL : 286
AtBGLU24   : AEAVEAFRQCEKCK-GGKIGIAHSPAWFEPHDFDEQSGATIDRALDFIMGWHLDTTMFGDY-PQTMKDIVGH-RL : 291
AtBGLU25   : AEAVEEFRKCGKCT-GGKIGIVQSPMWFEPYDKSSPSEEIVKRAMDFTLGWHMEPITHGDY-PQAMKDVVGS-RL : 287
AtBGLU26   : AEAVEVFRKCDHIK-NGQIGIAHNPLWYEPYDPDPDDVEGCNRAMDFMLGWHQHPTACGDY-PETMKKSVGD-RL : 287
AtBGLU27   : AEAVEVFRNNPKCK-DGKIGIAHCPVWFEPYDSCPKDIEACERAMEFMFGWHMDPTVYGDY-PAVMKKSIGK-RL : 286
AtBGLU28   : AAAVQEFRKCNKTQ-DGQIGIVLSPLWFEPYDSSPADNEAVKRALATELDWHLDPVIHGDY-PEMMKKLAGN-RL : 286
AtBGLU29   : AAAVQEFRNCNKTLQDGKIGIVISPWWLEPYDSSSADKEAVERGLPLELEWHLNPVIYGDY-PETMKKHVGN-RL : 287
AtBGLU30   : AAAVEEFRKCEKTSHDGQIGIVLSPRWFEPYHSSTDDKEAAERALAFEIGWHLDPVIHGDY-PEIVKKYAGN-KL : 287
AtBGLU31   : AAAVEEFRKCDKISQDSKIGIVLSPYWFEPYDSSNADKEAVERALAFNIGWHLSPLVFGDY-PETIKISAGN-RL : 287
AtBGLU32   : AAAVEEFRKCDKISQDAKIGIVLSPYWFEPYDISESDKEAVERALVFNIGWHLSPLVFGDY-PETIKTTAGN-RL : 287
AtBGLU33   : LAAVDEFRNCKKVEGGGKIGIVLVSHWFEPKDPSSEDVKAARRSLEYQLGWFLRPLTYGQY-PAEMLEDVNI-RL : 288
AtBGLU34   : AKTVSLYRKRYQKFQGGKIGTTLIGRWFAPLNESELDKAAAKRAFDFFVGWFLDPLVYGKY-PTIMREMVGD-RL : 281
AtBGLU35   : AKTVSLYRKRYQKFQGGKIGTTLIGRWFVPLNESELDKAAAKRAFDFFVGWFLDPLVYGKY-PTIMREMVGD-RL : 281
AtBGLU36   : MEAVSLYRKRYQKFQGGKIGTTLIGRWFIPLNENDLDKAAAKREFDFSVLGSTGVRTISK---D--NERLGD-RL : 272
AtBGLU37   : ATVVDLYRTRYK-YQGGKIGPVMITRWFLPYDDTLESKQATWRAKEFFLGWFMEPLTKGKY-PYIMRKLVGN-RL : 285
AtBGLU38   : AAAVDVYRTKYKDDQKGMIGPVMITRWFLPFDHSQESKDATERAKIFFHGWFMGPLTEGKY-PDIMREYVGD-RL : 286
AtBGLU39   : AKVVHLYRKKYKPKQRGQIGVVMITRWFVPYDSTQANIDATERNKEFFLGWFMEPLTKGKY-PDIMRKLVGR-RL : 255
Os4bglu18  : ATAIEIYKRKYQSKQRGMIGMVLYSTWYEPLRDVPEDRLATERALAFETPWFLDPLVYGDY-PPEMRQILGG-RL : 287
Os4bglu14  : ANAVSVYRNKYQGKQGGQIGIALSITWYEPFRNTTIDLLAVKRALSFGASWFLDPILLGDY-PTEMREVLGQ-SL : 286
Os4bglu16  : AAAVHNYKTNYQAKQGGSIGIVIAVKWYEPLTNSTEDVRAARRALAFEVDWFLDPIFFGDY-PREMREILSS-NL : 287
AtBGLU45   : AKAVNIYKTKYQKEQKGSIGIVVQTSWFEPISDSNADKEAAERAQSFYSNWILDPVIYGKY-PKEMVDILGP-AL : 287
AtBGLU46   : AKAIQIYRTKYQREQKGIIGIVVQTSWFEPISDSIADKNAAERAQSFYSNWILDPVVYGKY-PEEMVNLLGS-AL : 286
AtBGLU47   : LAAVNLYRTKFQEQQRGQIGIVMNTIWFEPISDSLADRLAADRAQAFYLTWFLDPVVFGRY-PREMREILGD-DL : 286
Os9bglu33  : ASAVSIYRQKYQAIQGGQIGITLMVRWHEPYTDKTADAAAAIRMNEFHIGWFLHPLVHGDY-PPVMRSRVGV-RL : 286
Os9bglu32  : ASAVSIYRRKYQAIQGGQIGITLLGWWYEPYTDAVADAAAAIRMNEFHIGWFMNPLVHGDY-PPVMRSRVGA-RL : 286
Os9bglu31  : SSAVSLYREKYQATQGGQIGLTLLGWWYEPGTQDPEDVAAAARMNDFHIGWYMHPLVYGDY-PPVMRKNVGS-RL : 287
Os1bglu2   : AAVVRLYREKYQALQKGIVGINMFSLWSYPLTNSIADLQAAQRYKDFSYGWILHPLVFGDY-PQVMKKTIGS-RL : 288
Os1bglu3   : AAVVRLYREKYQTLQKGIVGINVLSLWSYPLTDSTADLQAAQRYKDFTYGWILHPLVFGDY-PQVMKKAIGS-RL : 287
Os1bglu5   : ASVTKLYREKYQVAGKGIIGISVYTFWAYPLTNSTVDLEATKRCQDFIVHWVLRPLVFGDY-PQVMKNIVGS-RL : 287
Os5bglu22  : ASVTRLYRDKYQATQEGFVGMNIYSFWNYPFSSSSADIAATQRALDFMVGWILDPLVYGDY-PEIMKKKAGS-RI : 286
Os5bglu23  : ASAVRLYWDKYQAKQKGVVGTNIYSFWPYPLSRSCADIDAVQRVLDFTIGWILDPLVYGDY-PEIMKKQAGS-RI : 286
Os5bglu20  : ASAVRLYREKYQVAQKGIVGINIYSLWFYPLTDSAEDIGATERAKQFMYGWILHPLVFGDY-PETIKKVVGS-RL : 286
Os5bglu21  : ASAVRLYREKYKVAQKGIIGINIYSMWFYPFTDSAEDIGATERAKKFIYGWILHPLVFGDY-PDTMKKAAGS-RL : 286
Os5bglu19  : ASAVRLYREKHQAAQKGVVGMNIYSMWFYPLTESTEDIAATERVKDFMYGWILHPLVFGDY-PETMKKAAGS-RL : 287
AtBGLU1    : ASVSRLYKQKYKDIQGGSVGFSLFAMNFTPSTNSKDDEIATKRANDFYLGWMLEPLIYGDY-PDVMKRTIGS-RL : 282
AtBGLU3    : ASASRLYKQKYKDMQGGSVGFSLFSLGFTPSTSSKDDDIAVQRAKDFYFGWMLEPFIFGDY-PDEMKRTVGS-RL : 281
AtBGLU4    : ASVSRRYKQKYKDKQGGSIGFSLFILGLIPTTSSKDDATATQRAQDFYVGWFLRPLLFGDY-PDTMKRTIGS-RL : 282
AtBGLU5    : ASASRLYKQKYKDKQGGSIGFGLYLMGLTPSTSSKDDAIATQRAKDFYFGW------------------------ : 260
AtBGLU6    : ASVSRLYKQNYKDKQGGSIGFSILTIGFSPSTSSKDDAIATQRANDFFNGWMLGPLIYGDY-PDTMKRIVGS-RM : 283
AtBGLU7    : SSASNLYKLKYKTKQRGSVGLSIYAYGLSPYTDSKDDETATERAEAFLFGWMLKPLVVGDY-PDIMKRTLGS-RL : 280
AtBGLU8    : ASASNLYKLKYKSKQRGSIGLSIFALGLTPYTNSKDDEIATQRAKAFLYGWMLKPLVFGDY-PDEMKRTLGS-RL : 274
AtBGLU9    : ASASKLYKLKYKSKQKGSIGLSIFAFGLSPYTNSKDDEIATQRAKTFLYGWMLKPLVFGDY-PDEMKKTVGS-RL : 283
AtBGLU10   : ASASKLYKLKYKSTQKGSIGLSIFAFGLSPYTNSKDDEIATQRAKAFFYGWMLKPLVFGDY-PDEMKRTVGS-RL : 286
AtBGLU11   : ASATILYKQQYKYKQHGSVGISVYTYGAVPLTNSVKDKQATARVNDFYIGWILHPLVFGDY-PETMKTNVGS-RL : 285
Osbglu39   : MSLTEYLHQTKPG---QLMGGMLAHQLIYPATCKPRDIFCAQQYDEFLNQNLLGVFAGQGYSPAVMAVVEQEGFG : 270
Os11bglu36 : SEAYDYIHSKSKNERKPIVGVAHHVSFTRP------------------YGLFDVAAVALAN-------SLT--LF : 261
             a               g 6G         p         a  r      g    p   g y p

                      *       320         *       340         *       360         *
Os10bglu34 : PKFTEKEAAL-VNGSLDFMGINHYTTFYTKDDQSTVIE---KLLNNTLADTATISVPFRNGQ-PIGDRANSIWLY : 357
Os3bglu6   : PRFTADEAAV-VKGALDFVGINHYTTYYTRHNNTNIIG---TLLNNTLADTGTVSLPFKNGK-PIGDRANSIWLY : 357
AtBGLU40   : PVFTGSQSSL-VKGSLDFVGINHYTTYYARNNATNLIG---TLLHDAVSDSGTVTLPFK-GLSTIGDRASSIWLY : 357
Os6bglu25  : PQFSTHASKL-VSGSLDFVGINHYTTLYARNDRLRIRK---LVMDDASTDSAVIPTAYRHGK-KIGETAASSWLH : 357
AtBGLU41   : PKITPEMYKT-IKGAFDYVGINHYTTLYARNDRTRIRK---LILQDASSDSAVITSSFRGGV-AIGERAGSSWLH : 358
Os1bglu4   : PTFSEKDKEF-IRNKIDFVGINHYTSRFIAHHQDPE-D---IYFYRVQQVERIEKWNT--GE-KIGERAASEWLF : 343
AtBGLU42   : PRFTPEEKEFMLQNSWDFLGLNHYTSRLISHVSNKEAE---SNFYQAQELERIVELEN--GD-LIGERAASDWLY : 346
Os1bglu1   : PKFTADEVHM-VKGSIDYVGINQYTAYYVRDQQPNATT---LPS-YSSDWHAAPIYER-DGV-PIGPRANSDWLY : 350
Os7bglu26  : PTFSDEESRM-VKDSIDYVGINHYTSFYMKDPGPWNLT---PTS-YQDDWHVGFAYER-NGV-PIGAQANSYWLY : 351
AtBGLU43   : PKFTEEEVKM-VKGSIDFVGINQYTTYFMSDPKISTTP---KDLGYQQDWNVTFNFAK-NGT-PIGPRAHSEWLY : 353
AtBGLU44   : PKFTEKEVKM-VKGSIDFVGINQYTTYYMSEPHPTTKP---KDLGYQQDWNVEFGFAK-LGK-PIGPRAYSSWLY : 355
Os3bglu8   : PTFTPEQAKL-VKGSADYFGINQYTANYMADQPAPQQA---ATS-YSSDWHVSFIFQR-NGV-PIGQQANSNWLY : 351
Os3bglu7   : PKFTPEQARL-VKGSADYIGINQYTASYMKGQQLMQQT---PTS-YSADWQVTYVFAK-NGK-PIGPQANSNWLY : 351
Os12bglu38 : PNFTREQSEM-IKGSADYIAINHYTTYYVS--HHVNKT---SIS-YLNDWDVKISYER-NGV-PIGKQAYSNWLY : 349
Os11bglu37 : PKLTLAQSAM-VKGSYDFIGINYYTTYYAKSMPP-PNS---NELSYDVDSRANTTGFR-NGK-PISPQEFTPIFF : 351
Os11bglu35 : PKFTPAQSAM-VKGSYDFIGINYYTTYYAKSVPP-PNS---NELSYDVDSRANTTGFR-NGK-PIGPQ-FTPIFF : 354
Os9bglu30  : PEFTPEQSAM-VKGSYDFIGVNYYTTYYAKSIPP-PNS---NELSYDLDNRANTTGFR-NGK-PIGPQEFTPIFF : 355
Os9bglu29  : PAFTAEQAAA-VRGSYDFIGVNYYTTYYAKSVPL-PSS---NRLSYDTDIRANTTGFR-NGK-PIGPQEFTPIFF : 355
Os8bglu28  : PAFTAEQSEM-LRGSFDFIGLNYYTSNYAVAAPP-PNK---LHPSYLTDNWVNATGYR-NSI-PIGPPAYTPIFF : 354
Os8bglu27  : PKFTAEQSEL-VKGSYDFIGLNYYTTNYAKSVLRRPSK---LKPAYATDNWVNQTAYR-NGV-PIGPPAFTKIFF : 356
Os4bglu10  : PEFSKEQSEM-VKGAFDFIGLNYYASSYADNDPP-SYG---HNNSYNTDSHAKITGSR-NGI-PIGPQAASFWFY : 356
Os4bglu11  : PEFSKEQSGM-VKGAFDFIGLNYYTSSYADNDPP-SHG---HNNSYNTDAHAKITGSR-NGI-PIGPQAASFWFH : 354
Os4bglu9   : PKFTKEQSEM-VKGAFDFIGLNYYSSSYAENVLP-SYG---LKNSYNTDFHARITGSR-NGT-LIGPQAASSWLH : 355
Os4bglu13  : PQFTKEQSEL-IKGSFDFIGLNYYTSNYAGSLPP-SNG---LNNSYSTDARANLTAVR-NGI-PIGPQAASPWLY : 355
Os4bglu12  : PQFTKEQSKL-VKGAFDFIGLNYYTANYADNLPP-SNG---LNNSYTTDSRANLTGVR-NGI-PIGPQAASPWLY : 355
Os6bglu24  : PRFTKEQSKA-INGSFDFIGLNYYTARYIQGTKQ-DSN---SHKSYSTDSLTNERVER-NGT-DIGPKAGSSWLY : 354
AtBGLU17   : PKFTKKQSKM-VRGSFDFFGLNYYTSRYVEDVMF-YAN---TNLSYTTDSRVNQTTEK-NGV-PVGEPTSADWLF : 355
AtBGLU12   : PTFTAKQSKM-LKGSYDFIGRNYYSSSYAKDVPC--SS---ENVTLFSDPCASVTGER-EGV-PIGPKAASDWLL : 356
AtBGLU13   : PTFTAKQSKM-LKGSYDFIGINYYSSSYAKDVPC--SS---ENVTLFSDPCASVTGER-EGV-PIGPKAASDWLL : 356
AtBGLU14   : PTFTSKQSNM-LKGSYDFIGINYYSSSYAKDVPC--SS---ENVTMFSDPCASVTGER-DG-------------- : 343
AtBGLU15   : PIFTAQQSKM-LKGSYDFIGINYYSSTYAKDVPC--ST---KDVTMFSDPCASVTGER-DGV-PIGPKAASDWLL : 355
AtBGLU16   : PTFTPEESEM-LKGSYDFIGVNYYSSLYAKDVPC--AT---ENITMTTDSCVSLVGER-NGV-PIGPAAGSDWLL : 306
AtBGLU18   : PKFTEAEKKL-LKGSTDYVGMNYYTSVFAKEIS--PDP---KSPSWTTDSLVDWDSKSVDGY-KIGSKPFNGKLD : 356
AtBGLU19   : PRFTKAQKAK-LKDSTDFVGINYYTSFFAKADQK-VDS---RNPTWATDALVEFEPKTVDGSIKIGSQPNTAKMA : 358
AtBGLU20   : PKFTEAQKEK-LKNSADFVGINYYTSVFALHDEE-PDP---SQPSWQSDSLVDWEPRYVDKFNAFANKPDVAKVE : 359
AtBGLU21   : PQFTAAQKAK-LKDSTDFVGLNYYTSTFSNYNEK-PDP---SKPSWKQDSLVSWEPKNVDHS-AIGSMPLTAALP : 352
AtBGLU22   : PKFTSSQKAK-LKDSTDFVGLNYYTSTFSNHNEK-PDP---STPSWKQDSLVAWEPKNVDHS-AIGSQPLTAALP : 354
AtBGLU23   : PKFTTEQKAK-LKASTDFVGLNYYTSVFSNHLEK-PDP---SKPRWMQDSLITWESKNAQNY-AIGSKPLTAALN : 355
AtBGLU24   : PKFTTEQIAK-LKNSADFVGINYYTSTFSKHLEK-PNH---AEPKFKQDSLVEWKNKNVNNI-TIGSKPETGPLP : 360
AtBGLU25   : PSFTPEQKEK-LKGSYDFVGINYFTSTFVAHTDN-VNP---EKPSWEADSRLQLHSNNVDGF-KIGSQPATAKYP : 356
AtBGLU26   : PSFTPEQSKK-LIGSCDYVGINYYSSLFVKSIKH-VDP---TQPTWRTDQGVDWMKTNIDGK-QIAKQGGSEWSF : 356
AtBGLU27   : PSFTAAQSKK-LRGSFDFVGVNYYSAFYVKNIDE-VNH---DKPNWRSDARIEWRKENNAGQ-TLGVRGGSEWDF : 355
AtBGLU28   : PSFTPEQSKM-LKNSSDFIGINYYTARYVAHIPQ-ADP---ARPRFVTDHQLQWRVTNHSNH-QFGPGEDRGILQ : 355
AtBGLU29   : PAFTPEQSKM-LINSSDFIGVNYYSIHFTAHLPH-IDH---TRPRFRTDHHFEKKLINRSNH-ETGPGDDRGKIH : 356
AtBGLU30   : PSFTVEQSKM-LQNSSDFVGINYYTARFAAHLPH-IDP---EKPRFKTDHHVEWKLTNHSGH-IIGPGEERGFLF : 356
AtBGLU31   : PSFTKEQSMM-VKNSFDFIGVNYYTARFVAHDLN-VDI---SRPRFMTDQHLQYKLTNRTGDTISLESDGTKILW : 357
AtBGLU32   : PSFTKEQSMM-LQNSFDFIGINYYTARFVAHDLH-VDL---SRPRFTTDQHLQYKLTNRSGDHISSESDGTKILW : 357
AtBGLU33   : REFTPEESEK-LRKSLDFVGLNYYGAFFSTPLAK-VNS---SQLNYETDLRVNWTVITNNLS-LPDLQTTSMGIV : 357
AtBGLU34   : PEFTPEQSAL-VKGSLDFLGLNYYVTQYATDAPP--P----TQLNAITDARVTLGFY-RNGV-PIGVVAP--SFV : 345
AtBGLU35   : PEFTPEESAL-VKGSLDFLGLNYYVSQYATDAPP--P----TQPNAITDARVTLGFY-RNGS-PIGVVAS--SFV : 345
AtBGLU36   : PKFTPKQSAL-LKGSLDFLGLNYYVTRYATYRPPPMP----TQHSVLTDSGVTIGFE-RNGV-SIGVKA---SIN : 337
AtBGLU37   : PKFNSTEARL-LKGSYDFLGLNYYVTQYAHALDPSPP----EKLTAMTDSLANLTSLDANGQ-PPGPPFSK-GSY : 353
AtBGLU38   : PEFSETEAAL-VKGSYDFLGLNYYVTQYAQNNQTIVPS---DVHTALMDSRTTLTSKNATGH-APGPPFNA-ASY : 355
AtBGLU39   : PKFNKKEAKL-VKGSYDFLGINYYQTQYVYAIPANPP----NRLTVLNDSLSAFSYENKDG--PIGPWFNA-DSY : 322
Os4bglu18  : PSFSPEDRRK-LRYKLDFIGVNHYTTLYARDCMFSDCP---QGQ--ETQHALAAVTGESNGL-PIGTPTAMPTFY : 355
Os4bglu14  : PKFTSKQKNRLQSTKLDFIGLNHYTTCYVKDCIFSPCE---IDP--VNADARVFSLYERDGV-PIGKATGAPFFH : 355
Os4bglu16  : PKFTPEEKKLLQNNKVDFIGINHYTAIYAKDCIYSPCT---LDT--YEGNALVYAIGRRNGK-IIGKPTALHGYF : 356
AtBGLU45   : PQFSSNEVKNLEKSRADFVGINHYTSYFIQDCLTSACN---TGHGAFKAEGYALKLDRKGNV-TIGELTDVNWQH : 358
AtBGLU46   : PKFSSNEMNSLMSYKSDFLGINHYTSYFIQDCLITACN---SGDGASKSEGLALKLDRKGNV-SIGELTDVNWQH : 357
AtBGLU47   : PEFTKDDLKS-SKNALDFIGINQYTSRYAKDCLHSVCE---PGKGGSRAEGFVYANALKDGL-RLG--------- : 347
Os9bglu33  : PSITASDSEK-IRGSFDFIGINHYYVIFVQSIDAN----EQKLRDYYIDAGVQ-------------GEDDTENIQ : 343
Os9bglu32  : PSITASDSEK-IRGSFDFIGINHYFVIFVQSSDANH---DQKLRDYYVDAGVQENG---------GGGFDKEHYQ : 348
Os9bglu31  : PSFTAEESKR-VLESYDFVGFNHYVAIFVRADLSKL---DQSLRDYMGDAAVKYDLPFLKSNN--PLGLTSDFMT : 356
Os1bglu2   : PSFSQVQTEL-IKGAIDFIGINHYYSAYVNYRPLVE-----GVRDYVADRSVSAR-----VRFN----YEPTEYP : 348
Os1bglu3   : PSFSKVQTEL-VKGTLDFIGVNHYFSLYVSDLPLAK-----GVRDFIADRSVSCRGLLQGVRF-------PTRSM : 349
Os1bglu5   : PSFTKAQSED-VKGSLDFIGMNHYYSLYVNDRPLGK-----GTRDFVADISIYYRG----SKT-------PTSIG : 345
Os5bglu22  : PSFTEEQSEL-IRGSADFIGINHYTSVYISDASNGETV---GPRDYSADMAATFRISRN----Q----FVPTRLP : 349
Os5bglu23  : PSFTKEQSEL-IRGSADFIGINHYKSLYVSDGSNREKA---GLRDYNADMAAHFRGTT----------YAPSKTL : 347
Os5bglu20  : PFFSNHESEL-VTNAFDFVGLNHYSSVYTSNNNNVVKA---PLQDLTADIATLFRATKNDTPT----PEFLPGNT : 353
Os5bglu21  : PIFSNHESEM-VTNSFDFIGLNHYSSVYTSNNNNVVKA---PLQDLTADVATLFRVTKNDTPT----PVFVPGTI : 353
Os5bglu19  : PLFSDYESEL-VTNAFDFIGLNHYTSNYVSDNSNAVKA---PLQDVTDDISSLFWASKNSTPTLLLLRQFLPGTS : 358
AtBGLU1    : PVFSKEESEQ-VKGSSDFIGVIHYLTALVTNIDINPSL--SGIPDFNSDMG------------------------ : 330
AtBGLU3    : PVFSKEESEQ-VKGSSDFIGIIHYLAASVTSIKIKPSI--SGNPDFYSDMGVSMTWTVLGNFS-------AFEYA : 346
AtBGLU4    : PVFSEKESEQ-VKGSCDFVGVIHYHAASVTNIKSKPSL--SGNPDFYSYMET--DFGKSLDF----------QYA : 342
AtBGLU5    : --------------------------------------------------------------------------- :   -
AtBGLU6    : PVFSEEESEQ-VKGSSDYIGINHYLAASITNSKLKPSI--SGNPDFYSDMNV--ILSFFANFS-------SSEYD : 346
AtBGLU7    : PVFSEEESKQ-VKGSSDFVGVVHYNTFYVTNRPAPSLV-TSINKLFFADIGAYLIAAGNASL---------FEFD : 344
AtBGLU8    : PVFSEEESEQ-VKGSSDFVGIIHYTTVYVTNQPAPYIFPSSTNKDFFTDMGAYIISTGNSSS---------FVFD : 339
AtBGLU9    : PVFSEEESEQ-VKGSSDFIGIIHYTTFYVTNHQPSASLFPSMGEGFFKDMGVYIIPTGNSSF---------LVWE : 348
AtBGLU10   : PVFSEEESEQ-LKGSSDFIGIIHYTTFYVTN-KPSPSIFPSMNEGFFKDMGVYMISAANSSF---------LLWE : 350
AtBGLU11   : PAFTEEESEQ-VKGAFDFVGVINYMALYVKDNSSSLKP---NLQDFNTDIAVEMTLVGNTSIE-------N-EYA : 348
Osbglu39   : DIYRDEDLALLARTKNDFMAFSYYASKTLDSDVIPEGT---PVNYYLLHGEKNNPYLKATEWN----------WQ : 332
Os11bglu36 : PYVDSI-CD---KL--DFIGINYYGQEVISGPGLKLVDN----DEYSESGRG----------------------- : 303
             p               d  g n                          d

               380         *       400         *       420         *       440         *
Os10bglu34 : IVPRSM-RILMNYVKDRYNKP-TVYITENGMDDGNSPF--ISLKNALKD-DKRTKYHNDYLT-NLADSIREDGCD : 426
Os3bglu6   : IVPRGM-RSLMNYVKERYNSP-PVYITENGMDDSNNPF--ISIKDALKD-SKRIKYHNDYLT-NLAASIKEDGCD : 426
AtBGLU40   : IVPRGM-RSLMNYIKHRYGNP-PVFITENGMDDPNSIL--ISRKDALKD-AKRIKYHHDYLS-SLQASIKEDGCN : 426
Os6bglu25  : IVPWGM-FKLMKHVKEKYGNP-PVVITENGMDDANHPF--SRLEDVLQD-DKRIQYHNDYMS-NLLDAIRKEGCN : 426
AtBGLU41   : IVPWGI-RKLAVYVKDIYGNP-PVFITENGMDEKNSPF--IDMEKALKD-DKRIGFHRDYLS-NLSAAIRNDECD : 427
Os1bglu4   : IVPWGL-RKLLNYAAKRYGNP-VIYVTENGMDEEDDQS--ATLDQVLND-TTRVGYFKGYLA-SVAQAIK-DGAD : 411
AtBGLU42   : AVPWGI-RKTLNYMSKKYNHP-PIFITENGMDDEDDGS--ASIHDMLDD-KRRVDYFKSYLA-NVSQAIE-DGVD : 414
Os1bglu1   : IVPWGL-YKAVTYVKEKYGNP-TMFLSENGMDDPGN----VTIAQGVHD-TTRVAYYRSYIT-KLKEAID-DGAN : 416
Os7bglu26  : IVPWGI-NKAVTYVKETYGNP-TMILSENGMDQPGN----VSITQGVHD-TVRIRYYRNYIT-ELKKAID-DGAK : 417
AtBGLU43   : NVPWGM-YKALMYIEERYGNP-TMILSENGMDDPGN----ITLTQGLND-TTRVKYYRDYLV-QLKKAVD-DGAN : 419
AtBGLU44   : NVPWGM-YKALMYMKERYGNP-TMILSENGMDDPGN----VTLAQGLHD-TTRIKYYKDYLT-NLKKARD-DGAN : 421
Os3bglu8   : IVPTGM-YGAVNYIKEKYNNP-TIIISENGMDQSGN----LTREEFLHD-TERIEFYKNYLT-ELKKAID-DGAN : 417
Os3bglu7   : IVPWGM-YGCVNYIKQKYGNP-TVVITENGMDQPAN----LSRDQYLRD-TTRVHFYRSYLT-QLKKAID-EGAN : 417
Os12bglu38 : VVPWGI-YKAVMHVKEKYKDP-IIIIGENGIDQPGNETLP----GALYD-FFRIQYFDQYLH-ELKRAIK-DGAR : 415
Os11bglu37 : NYPPGI-REVLLYTKRRYNNP-AIF-TENGIDEGNNSTVP----EALRD-GHRIEFHSKHL--FVNHAIR-NGVN : 415
Os11bglu35 : NYPPGI-REVLLYTKRRYNNP-AIYITENGIDEGNNSTVP----EALRD-GHRIEFHSKHLQ-FVNHAIR-NGVN : 420
Os9bglu30  : NYPPGL-RELLLYTKRRYNNP-TIYVTENGIDEGNNSTLP----EALKD-GHRIEFHSKHLQ-FVNHAIK-NGVN : 421
Os9bglu29  : NYPPGL-RELLLYTKRRYNNP-IIYVTENGIAEGNNKSLPI--TEALKD-GHRIEFHSKHLQ-FVNHAIK-NGVN : 423
Os8bglu28  : NYPPGL-RELLLYVKRRYNNP-TIYITENGTDEANNSTIPI--SEALKD-ETRIGFHYKHLQ-FVHKAIQ-EGVK : 422
Os8bglu27  : TYAPGL-RELLLYTKRKYNDP-DIYIAENGTDEANNSTIPI--AEALKD-DNRISFHYQHLR-FTQLAIK-EGVK : 424
Os4bglu10  : IYPEGL-RELLLHIKENYGNP-TIYITENGVDEINNKTMRL--KEALKD-DIRIEYYHKHLL-ALLSAMR-DGAN : 424
Os4bglu11  : IYPEGI-CEMLLYVKENYGNP-TIYITENGVDEVNNKTMPL--EEALKD-DTRIEYYHKHLL-ALLSAMR-DGAN : 422
Os4bglu9   : IYPQGL-RELLLYIKENYGNP-TIFITENGVDEVNNKTMPL--KEALND-NTRIEYYHKHLL-ALRNAMR-DGAN : 423
Os4bglu13  : IYPQGF-RELVLYVKENYGNP-TIYITENGVDEFNNKTLPL--QEALKD-DTRIDYYHKHLL-SLLSAIR-DGAN : 423
Os4bglu12  : VYPQGF-RDLLLYVKENYGNP-TVYITENGVDEFNNKTLPL--QEALKD-DARIEYYHKHLL-SLLSAIR-DGAN : 423
Os6bglu24  : IYPKGI-EELLLYTKRTYNNP-TIYITENGVDEVNNENLSL--KEALID-TTRIEFYRQHLF-HVQRALR-QGVD : 422
AtBGLU17   : ICPEGF-QDVLLYIKSKFQNP-VILVTENGMPSENDKSLSV--NIALND-EAKIKYHQLHLT-ALLEAVS-QGAD : 423
AtBGLU12   : IYPKGI-RDLLLYAKYKFKDP-VMYITENGRDEASTGKID------LKD-SERIDYYAQHLK-MVQDAIS-IGAN : 420
AtBGLU13   : IYPKGI-RDLLLYAKYKFKDP-VMYITENGRDEASTGKID------LKD-SERIDYYAQHLK-MVQDAIS-IGAN : 420
AtBGLU14   : ----GI-RDLILYAKYKFKDP-VMYITENGRDEASTGKIL------LKD-GDRIDYYARHLK-MVQDAIL-IGAN : 403
AtBGLU15   : IYPKGI-RDLVLYAKYKFKDP-VMYITENGRDEFSTNKIF------LKD-GDRIDYYARHLE-MVQDAIS-VGAN : 419
AtBGLU16   : IYPKGI-RDLLLHAKFRYNDP-VLYITENGVDEANIGKIF------LND-DLRIDYYAHHLK-MVSDAIS-IGVN : 370
AtBGLU18   : VYSKGL-RYLLKYIKDNYGDP-EVIIAENGYGEDLGEKHN-DVNFGTQD-HNRKYYIQRHLL-SMHDAICKDKVN : 426
AtBGLU19   : VYAKGL-RKLMKYIKDRYNSP-EIIITENGYGEDLGDKDT-DLSVALND-HNRKYYLQRHLL-ALNEAICEDKVN : 428
AtBGLU20   : VYAKGL-RSLLKYIKDKYGNP-EIMITENGYGEDLGEQDT-SLVVALSD-QHRTYYIQKHLL-SLHEAICDDKVN : 429
AtBGLU21   : VYAKGF-RKLLKYIKDKYANP-EIMIMENGYGDKLGTTD--SVDVGTAD-HNRKYYLQRHLL-AMNEAICIDKVR : 421
AtBGLU22   : VYAKGF-RSLLKYIKDKYANP-EIMIMENGYGDKLKDKD--SVEVGTAD-YNRKYYLQRHLL-AMNEAICIDKVR : 423
AtBGLU23   : VYSRGF-RSLLKYIKDKYANP-EIMIMENGYGEELGASD--SVAVGTAD-HNRKYYLQRHLL-SMQEAVCIDKVN : 424
AtBGLU24   : VYSTGF-RKVLKYVKDKYANP-EIIIMENGYGENLKEND--SVENGTAD-YNRESYLKKHLW-SMHKAICEDKVN : 429
AtBGLU25   : VCADGL-RKVLKYIKENYNDP-EIIVTGNGYKETLEEKD--VLPDALSD-SNRKYYHMRHLM-ALHGAVCEDKVN : 425
AtBGLU26   : TYPTGL-RNILKYVKKTYGNP-PILITENGYGEVAEQSQSLYMYNPSID-TERLEYIEGHIH-AIHQAIHEDGVR : 427
AtBGLU27   : LYPQGL-RKFLNYAKNKYESP-KFMITENGHCDIDYEKK--PKLSNLMD-LQRTEYHKKHLQ-SIQQAIQEDGVV : 424
AtBGLU28   : SHPEGL-RKVLNYIKDKYNNP-IVYIKENGINDYDDGTK--SREEILND-TFRISYHEDHLQ-QLQKAIIEDGCD : 424
AtBGLU29   : SHPEGL-RRVLNYIKDKYNNP-IVYVKENGIDHYDDGTK--SRETILKD-TFRISYHQDHLK-QVHKAIM----- : 420
AtBGLU30   : SHPEGL-RKVLNYIKERYNNM-PVYIKENGINDNDDGTK--PREEIVKD-TFRIEYHKTHFE-ELHKAIVEDGCD : 425
AtBGLU31   : SYPEGL-RKILNYIKNKYNNP-TIYITENGFDDYENGTV--TREEILED-TKRIEYHQKHLQ-ELQKAITEDGCD : 426
AtBGLU32   : SYPEGL-RKLLNYIKNKYNNP-TIYITENGFDDYENGSV--TREEIIED-TKRIEYHQNHLQ-QLQKAITEDGCN : 426
AtBGLU33   : IYPAGL-KNILKHIKDEYMDP-EIYIMENGMDEIDYGTK--NITEATND-YGRKEFIKSHIL-IMGKSIRMDKVR : 426
AtBGLU34   : YYPPGF-RQILNYIKDNYKNP-LTYITENGVADLDLG-N-VTLATALAD-NGRIQNHCSHLS--CLKCAMKDGCN : 413
AtBGLU35   : YYPPGF-RQILNYIKDNYKNP-LTYITENGVADLDLG-N-VTLATALAD-NGRIQNHCSHLS--CLKCAMKDGCN : 413
AtBGLU36   : FDVKDL-RHLVDFFLFVELLLLSTRIPSDSKSHQKQELL-MLIANALAD-NGRIQFQCSHLS--CLKCAIEDGCN : 407
AtBGLU37   : YHPRGM-LNVMEHFKTKYGDP-LIYVTENGFSTSGGP---IPFTEAFHD-YNRIDYLCSHLC-FLRKAIK----- : 416
AtBGLU38   : YYPKGI-YYVMDYFKTTYGDP-LIYVTENGFSTPGD----EDFEKATAD-YKRIDYLCSHLC-FLSKVIKEKNVN : 422
AtBGLU39   : YHPRGI-LNVLEHFKTKYGNP-LVYITENGESGCNVK---GYFAWCLGDNYELWPSRSFHVSPFYLLHRKDKGAF : 392
Os4bglu18  : VVPDGI-EKMVKYFMRRYNNL-PMFITENGYAQGGDSYT--DAEDWIDD-EDRIEYLEGYLT-KLAKVIR-DGAD : 423
Os4bglu14  : DVPRGM-EEAVTYYKQRYNNT-PTYITENGYSQASNSNM--TAKDFTND-TGRITYIQGYLI-SLASAIR-KGAD : 423
Os4bglu16  : VVPEAM-EKVVMYVNDRYRNT-TIYITENGYSQHSDT----SMEDLIND-VERVNYMHDYLK-YLSSAIR-KGAN : 422
AtBGLU45   : IDPTGF-HKMLNYLKDRYPNM-PMFITENGFGDLQKPET--TDKELLND-TKRIQYMSGYLE-ALQAAMR-DGAN : 426
AtBGLU46   : IDPNGF-RKMLNYLKNRYHNI-PMYITENGFGQLQKPET--TVEELLHD-TKRIQYLSGYLD-ALKAAMR-DGAN : 425
AtBGLU47   : -EPVGM-EEMLMYATERYKNI-TLYVTENGFG-----EN--NTGVLLND-YQRVKFMSNYLD-ALKRAMR-KGAD : 409
Os9bglu33  : CHSWSL-GKVLNHLKLEYGNP-PVMIHENGY-----SDSPDIFGKINYNDDFRSAFLQGYLE-ALYLSVR-NGSN : 409
Os9bglu32  : LHPWAL-GKMLHHLKLKYGNP-PVMIHENGD-----ADSPETPGKIDYDDDFRSDFLQSYLE-VLHLSIR-NGSN : 414
Os9bglu31  : STPWAL-KKMLNHLQEKYKNP-IVMIHENGA-----AGQPDPSGGNTYDDDFRSQYLQDYIE-ATLQSIR-NGSN : 422
Os1bglu2   : NDPKGL-QLALEYLRESYGDF-PFYIEENGK----------GSTNDSLDDPDRVDYIKGYIG-GVLDAIR-NGVD : 409
Os1bglu3   : GDPHGL-QLMLQHLKESYGDL-PIYVQENGKLDLLPVFSGKASSNDSLDDTDRVDYIKGYIE-GVLNATR-NGVN : 420
Os1bglu5   : PDPQGL-RLMVQYLQETYGNL-PIYILENGY----------GSSNDTVHDNDRVDYLKSYIG-SILTALR-NGAN : 406
Os5bglu22  : RDPKGL-QCMLEYLRDTYQGI-PVYIQENGF-------GHFGKDDDSLNDTDRVDYLSSYMG-STLAALR-NGAN : 413
Os5bglu23  : SDPKGL-QCMLEYLKDTYEGI-PVYVQENGF-------GQFDKED-SLNDTERVEYLSSYMG-GTLAALR-NGAN : 410
Os5bglu20  : VDPQGL-ENALEYIRENYGNL-TIYIQENGS----------GAPDGTLDDVERINYLQKYIA-ATLKAIR-NGAN : 414
Os5bglu21  : VDPRGL-EHALKYIREKYGNL-PIYIQENGI------ASGSGSSSETLDDVERINYLAKYIA-ATLKAIS--GAN : 417
Os5bglu19  : LDPRGL-ELALEYLQEKYGNL-LFYIQENGS----------GS-NATLDDVGRIDCLTQYIA-ATLRSIR-NGAN : 418
AtBGLU1    : ---------ILEYIKQSYGNP-PVYILENGK-------TMNQDLELQQKDTPRIEYLDAYIG-AVLKAVR-NGSD : 386
AtBGLU3    : VAPWAM-ESVLEYIKQSYGNP-PIYILENGT-------PMKQDLQLQQKDTPRIEYLHAYIA-AVLKSIR-NGSD : 410
AtBGLU4    : NTPWAM-EVVLEYIKQSYGNP-PVYILESAKQWQQIGTPMKQDSQLKQKDIPRVEYLHAYIG-GVLKSIR-NGSD : 413
AtBGLU5    : --------------------------------------------------------------------------- :   -
AtBGLU6    : VAPWAI-EAVL---------------------------------------------------------------- : 356
AtBGLU7    : AVPWGL-EGILQHIKQSYNNP-PIYILENGK-------PMKHGSTLQ--DTPRAEFIQAYIG-AVHNAIT-NGSD : 406
AtBGLU8    : AVPWGL-EGVLQHIKHRYNNP-PIYILENGS-------PMKHDSMLQ--DTPRVEYIQAYIG-AVLNAIK-SGSD : 401
AtBGLU9    : ATPWGL-EGILEYIKQSYNNP-PVYILENGM-------PMVRDSTLQ--DTQRIEYIQAYID-AVLNAMK-NGSD : 410
AtBGLU10   : ATPWGL-EGILEYIKQSYNNP-PIYILENGM-------PMGRDSTLQ--DTQRIEFIQAYIG-AMLNAIK-NGSD : 412
AtBGLU11   : NTPWSL-QQILLYVKETYGNP-PVYILENGQ-------MTPHSSSLV--DTTRVKYLSSYIK-AVLHSLSRKGSD : 411
Osbglu39   : IDPMGF-RTIITRYANDWRM--PVFPIENGI----GVIESWDGVNPIE-DTYRIDYHRAHID-AMKAAMFEDGAE : 398
Os11bglu36 : VYPDGLFRILIQFNERYKRLNIPFVITENGV----------SDET----DLIRKPYILEHLLATYAAII-MGVRV : 363
               p g                      e g                                          g

                    460         *       480         *       500
Os10bglu34 : VRGYFAWSLLDNWEWAAGY-TSRFGLYYVDYK--NRKRYPKNSVQWFKNLLAS : 476
Os3bglu6   : VRGYFAWSLLDNWEWAAGY-SSRFGLYFVDYKD-NLKRYPKNSVQWFKALLKT : 477
AtBGLU40   : VKGYFVWSLLDNWEWAAGY-SSRFGLYFVDYRD-NLKRYPKDSVHWFTSFLNS : 477
Os6bglu25  : VHGYFVWSLLDNWEWNSGY-TVRFGLYYIDYKN-NLTRIPKASVQWFSQVLAQ : 477
AtBGLU41   : VRGYFVWSLLDNWEWNSGY-TVRFGIYYVDYKN-NLTRIPKASARWFQTILSG : 478
Os1bglu4   : VRGYFAWSFLDNFEWAMGY-TKRFGIVYVDYKN-GLSRHPKASARWFSRFLKG : 462
AtBGLU42   : IKGYFAWSLLDNFEWAQGY-TKRFGLVYVDYKN-GLTRHPKSSAYWFMKFLKG : 465
Os1bglu1   : CIGYFAWSLLDNFEWKLGY-TSRFGLVYVDFR--TLRRYPKMSAYWFRDLVSS : 466
Os7bglu26  : VIGYFAWSLLDNFEWRLGY-TSRFGIVYVDYK--TLKRYPKDSAFWFKNMLSS : 467
AtBGLU43   : LTGYFAWSLLDNFEWLSGY-TSRFGIVYVDYK--DLKRYPKMSALWFKQLLKR : 469
AtBGLU44   : VVGYFAWSLLDNFEWLSGY-TSRFGIVYVDYK--TLKRYPKMSAQWFKQLLKR : 471
Os3bglu8   : VVAYFAWSLLDNFEWLSGY-TSKFGIVYVDFT--TLKRYPKDSANWFKNMLQA : 467
Os3bglu7   : VAGYFAWSLLDNFEWLSGY-TSKFGIVYVDFN--TLERHPKASAYWFRDMLKH : 467
Os12bglu38 : VTGYFAWSLLDNFEWRLGF-TSKFGIVYVDRS--TFTRYPKDSTRWFRKMIKS : 465
Os11bglu37 : VKGYFTWTFMDCFEWGDGY-LDLFGLIYVDRKT--LTRYRKDSSYWIEDFLRR : 465
Os11bglu35 : VKGYFT-TFMDCFEWGDGY-LDRFGLIYVDRKT--LTRYRKDSSYWIEDFLK- : 468
Os9bglu30  : VKGYFTWTFMDCFEWGDGY-LDRFGLIYVDRKT--LKRYRKESSYWIEDFLKR : 471
Os9bglu29  : VKGYFTWTFMDCFEWGDGY-LDRFGLIYIDRLN-NLKRYHKQSSYWIANFLKR : 474
Os8bglu28  : VKGYFTWTFMDCFEFGDGF-KDRFGLIYVDRAT--LARFRKKSSYWFADFLRR : 472
Os8bglu27  : VKGYFTWTFMDDFEWGDGY-TGRFGLIYVDRET--LKRYRKKSSYWFADFLKR : 474
Os4bglu10  : VKGYFAWSLLDNFEWSEGY-TVRFGINFVDYDN-GMKRYPKNSARWFKKFLRK : 475
Os4bglu11  : VKGYFAWSLLDNFEWAEGY-TVRFGINFVDYDD-GMKRYPKNSARWFKKFLQK : 473
Os4bglu9   : VKGYFAWSLLDNFEWADGY-TLRFGLNFVDYDD-GMKRHPKNSAHWFKKFLRE : 474
Os4bglu13  : VKGYFAWSLLDNFEWSNGY-TVRFGINFVDYND-GAKRYPKMSAHWFKEFLQK : 474
Os4bglu12  : VKGYFAWSLLDNFEWSNGY-TVRFGINFVDYND-GRKRYPKNSAHWFKKFLLK : 474
Os6bglu24  : VRGYFAWSLFDNFEWMDGY-SVRFGINYIDYKD-GLKRYPKRSSQWLQNFLHN : 473
AtBGLU17   : VRGYYIWSLMDDFEWEFGY-KYRYGLVYVDFQD-GLKRHLKSSALWYHHFLSN : 474
AtBGLU12   : VKGFFAWSLLDNFEWATGY-AVRFGLVYVDFNG-GRKRYPKKSAKWFKKLLNE : 471
AtBGLU13   : VKGFFAWSLLDNFEWATGY-SVRFGLVYVDFND-GRKRYPKKSAKWFRKLLSE : 471
AtBGLU14   : VKGFFAWSLLDNFEWASGY-TVRFGLVYVDFND-RRKRYLKKSAHWFRHLLNG : 454
AtBGLU15   : VKGFFAWSLLDNFEWAMGY-TVRFGLVYVDFKD-GCKRYPKKSAEWFRKLLNE : 470
AtBGLU16   : VKGYFAWSLMDNFEWSEGY-TVRFGLVFVDFED-GRKRYLKKSAKWFRRLLKG : 421
AtBGLU18   : VTGYFVWSLMDNFEWQDGY-KARFGLYYIDFQN-NLTRHQKVSGKWYSEFLKP : 477
AtBGLU19   : VTSYFLWSLMDNFEWQDGY-TARFGVYYIDFKN-NLTRMEKESAKWLSEFLKP : 479
AtBGLU20   : VTGYFHWSLMDNFEWQDGY-KARFGLYYVDYKN-NLTRHEKLSAQWYSSFLHD : 480
AtBGLU21   : VTGYFVWSLLDNFEWQDGY-KNRFGLYYVDFKN-NLTRYEKESAKYYKDFLAQ : 472
AtBGLU22   : VTGYFVWSLLDNFEWQDGY-NNRFGLYYVDFKN-NLTRYEKESAKYYKDFLGQ : 474
AtBGLU23   : VTGYFVWSLLDNFEWQDGY-KNRFGLYYVDFKN-NLTRYEKESGKYYKDFLSQ : 475
AtBGLU24   : VTGYFVWSLMDNFEWQDGF-KNRFGLYYIDYKN-NLTRHEKVSGKYYREFLSE : 480
AtBGLU25   : VKGYFVSSLMDGLEWEDGY-KTRSGLYYVDYGH-NMGRHEKQSAKWLSKLLEK : 476
AtBGLU26   : VEGYYVWSLLDNFEWNSGY-GVRYGLYYIDYKD-GLRRYPKMSALWLKEFLRF : 478
AtBGLU27   : VEGYFAWSLLDNCEWNAGY-GVRYGLFYVDYNN-GLKRFPKMSAMWFKEFLKR : 475
AtBGLU28   : VRGYYVWSLLDNFEWEHGY-STRFGVYYVDYDN-DLTRIPKDSVNWFKQFLDV : 475
AtBGLU29   : ----------------------------------------------------- :   -
AtBGLU30   : VRGYYAWSLMDNFEWEHGY-TARFGLYYVDFVN-GLKRYPKDSVKWFKRFLKK : 476
AtBGLU31   : VKGYFTWSLLDNFEWEHGY-AVRFGLYYVDYKN-GLQRHAKHSAMWFKHFLER : 477
AtBGLU32   : VKGYFTWSLLDNFEWEHGY-AVRFGLYYVDYKN-GLSRHAKNSAKWFKHFLQR : 477
AtBGLU33   : LKGYYIWSLMDNFEWDKGY-KVRFGLYYVDYND-NMKRYIRSSGKWLSEFLDS : 477
AtBGLU34   : VAGYFAWSLMDNYEFGNGY-TLRFGMNWVNFTN-PADRKEKASGKWFSKFLAK : 464
AtBGLU35   : VAGYFAWSLMDNYEFGNGY-TLRFGMNWVNFTN-PADRKEKASGKWFSKFLAK : 464
AtBGLU36   : VAGYFAWSLMDNYEFGNGY-TLRFDMNWVNFTN-PADRREKASGKWFSRFIAK : 458
AtBGLU37   : ----------------------------------------------------- :   -
AtBGLU38   : VKGYFAWSLGDNYEFCNGF-TVRFGLSYVDFANITGDRDLKASGKWFQKFINV : 474
AtBGLU39   : PS-FEA----------------------------------------------- : 397
Os4bglu18  : VRGYFAWSVVDNFEWLFGY-TLRFGLYYIDYRT--QERSPKLSALWYKEFLQN : 473
Os4bglu14  : VRGYFVWSLLDDFEWNFGY-TLRFGLYHVHYKT--LKRTPKLSVDWYRKFLTG : 473
Os4bglu16  : VGGYFAWSIVDNFEWVYGY-TVKFGLYQVDFDT--QERIPRMSAKWYRDFLTS : 472
AtBGLU45   : VKGYFVWSLLDNFEWLFGY-KVRFGLFHVDLTT--LKRSPKQSASWYKNYIEE : 476
AtBGLU46   : VKGYFAWSLLDNFEWLYGY-KVRFGLFHVDFTT--LKRTPKQSATWYKNFIEQ : 475
AtBGLU47   : VRGYFAWSLLDNFEWISGY-TIRFGMYHVDFST--QERTPRLSASWYKNFIFQ : 459
Os9bglu33  : TRGYFVWSMFDMFEFLYGY-RLRFGLCGVDFTAAARTRYLKNSARWYSGFLRG : 461
Os9bglu32  : TRGYFVWSLLDGFEFLSGY-GNRFGLCGVDFTAPARTRYVRSSARWYSDFLNG : 466
Os9bglu31  : VQGYFVWSFLDVFEYLFGY-RLRFGLYGVDFASPERTRYQRHSARWYAGFLRG : 474
Os1bglu2   : VRGYFVWSFVDVFELLEGY-QSRFGLYRVDFDDGAR-RRAAAASEALRSLVLG : 460
Os1bglu3   : ARGYFAWFFVDMFELLSGY-QTRYGLYRVDFDDAALPRRAKRSARWYRDFLKS : 472
Os1bglu5   : VKGYFVWSFVDVFEYLTGY-GQSYGLYRVDFADESRPRQARLSARWYSGFLKN : 458
Os5bglu22  : VKGYFVWSFLDVFELLAGY-HSPFGLHYVDFEDPNLPRQPKLSAHWYSKFLRG : 465
Os5bglu23  : VKGYFVWSFLDVFELFAGY-HSPFGLHHVDFEDPSLPRQPKLSAQWYSKFLRS : 462
Os5bglu20  : VKGYSMWSFIDIYEIFGGYNSWHYGLVAVDFGSTERRRQPRRSASWYSDFLKN : 467
Os5bglu21  : VKGYSMWSFVDLYELFGGYSTWHFGLVAVDFDSEKRRRQPRRSASWYSEFLKN : 470
Os5bglu19  : VKGYCVWSFMDQYEMFGDY-KAHFGIVAVDFGSEELTRQPRRSARWYSDFLKN : 470
AtBGLU1    : TRGYFVWSFMDLYELLNGY-KSSFGLYSVNFSDPHRKRSPKLSAHWYSGFLKG : 438
AtBGLU3    : TRGYFIWSFMDLYELVKGY-EFSFGLYSVNFSDPHRTRSPKLSAHWYSAFLKG : 462
AtBGLU4    : TRGYFVWSFMDLYELLGGY-EVGFGLYTVNFSDPHRKRSPKLSAYWYSDFLKG : 465
AtBGLU5    : ----------------------------------------------------- :   -
AtBGLU6    : ----------------------------------------------------- :   -
AtBGLU7    : TRGYFVWSMIDLYELIGRY-MTSYGMYYVNFSDPGRKRSPKLSASWYTGFLNG : 458
AtBGLU8    : TRGYFVWSLIDLFEVQVGY-KSSFGMYYVNFSDPGRKRSPKLSASWYTGFLNG : 453
AtBGLU9    : TRGYFVWSMVDVYEILSGY-TTSFGMYHVNFSDPGRKRTPKLSASWYTGFLNG : 462
AtBGLU10   : TRGYFVWSMIDLYELLSGY-TTSFGMYYVNFSDPGRKRTPKLSASWYTGFLNG : 464
AtBGLU11   : VKGYFQWSLMDVFELFGGY-ERSFGLLYVDFKDPSLKRSPKLSAHWYSSFLKG : 463
Osbglu39   : VIGYLGWGLIDILSSQGDM-RKRYGVVYVNRENHDLKRVPKKSYAWLKQVIHT : 450
Os11bglu36 : LGYLF-WTTSDNWEWADGY-GPKFGLVAVDRANN-LARKPRPSYFLFSRVVTT : 413
               g   w   d  e   g      g            r    s

```
