# Supplementary material for: Analysis of rice glycosyl hydrolase family 1 and expression of Os4bglu12 β-glucosidase
Source: BMC Plant Biol. 2006 Dec 29;6:33. doi: 10.1186/1471-2229-6-33 (PMC1781453; doi:10.1186/1471-2229-6-33)
Supplement: Additional File 3 — Alignment of full-length derived sequences of rice and other plant GH1 enzymes. All the full-length predicted proteins from rice GH1 genes, including Os11bglu36, which is from a distinct GH1 lineage, but not the possible endophyte genes Osbglu39 and Osbglu40, were aligned the related sequences defined in Figure 3 using ClustalX. The alignment was edited and shaded with Genedoc, as described in the methods. Darkest shading represents highest conservation, and the consensus for highly conserved regions is shown below the alignment. The file was exported as a rich text (.rtf) document for this picture. [file 1471-2229-6-33-S3.doc]

Supplementary Figure 2: Alignment of Predicted Rice GH1 Proteins with other plant enzyme sequences.

```

                      *        20         *        40         *        60         *
Os9bglu33  : --------------------------------------------------MATGELALVSSLFIVVVFLLLGAV- :  24
Os9bglu32  : --------------------------------------------------MAAGARALVPSPFIVVVFLLLAAA- :  24
Os9bglu31  : ---------------------------------------------------MTPARVVFICCVVLLAAAAAAAS- :  23
Os1bglu2   : ---------------MDVLILSSRAGSQTSLISLQKHNRRSNLLLILFFLLIIMGAAAAAGFFFVLLFLSVQGG- :  59
Os1bglu3   : -------------------------------------------------------MAAAAAFFCALLFISVQHG- :  19
Os1bglu5   : ------------------------------------------------------MAAAIAVVYLSLLLLLLHGAA :  21
Os5bglu22  : -------------------------------------------------MAVSSSTSTCSSFSLLLLLLLLAAAP :  26
Os5bglu23  : ---------------------------------------------------MAAYTSSLVSLLLLLLLLLLVAG- :  23
Os5bglu20  : --------------------------------------------ME-RRPLHLHLLLLFFSAWLMLLPLL----- :  25
Os5bglu21  : --------------------------------------------METRRPLHL-LLVFLSSPWLLLLLLLLLVV- :  29
Os5bglu19  : --------------------------------------------METRRPLHP--LLLFFSPWLLLLLLL--VV- :  26
GM_AAL9211 : ---------------------------------------------MMEPPQTRLMINVFIVSFLALLVNLVVG-- :  28
Os10bglu34 : -------------------------------------MGNGGRC--------MVEVVILLVLMAMSQGCDAQNTT :  30
Os3bglu6   : ---------------------------------MGRIKSSSGRCSTARLEAVAVLVVVFGVASSSLRGCIAQQSG :  42
Os6bglu25  : -----------------------------------------------------MSLLTLVHILVSFSACVEAIS- :  21
Os4bglu18  : --------------------------------------------------MAGGSKTRIHASLVSTLLLLLPLAS :  25
Os4bglu14  : -----------------------------------------------------MAAAWLVVLLTVHRLLHLSGVS :  22
Os4bglu16  : --------------------------------------------------MAVAAATRIAVVVVVLALAVLAPAA :  25
PC_AAC6961 : -----------------------------------------------------MEVSVLMWVLLFYSLLGFQVTT :  22
Os1bglu4   : --------------------------------------------------------------------MGSTGRD :   7
HB_AAP5105 : ------------------------------------------------------------MMKEKFLKEHPYLLE :  15
Os7bglu26  : -------------------------------------MR-KFIAALR------LALAAAAHLLLTLPPAQCYWLN :  31
HV_AAA8733 : -------------------------------------MRSSPVLLLV------IALVAAAH----LAPLECDGPN :  28
Os1bglu1   : -------------------------------------MGHRLVVVLL------LALLVAGAARAAEQAAGEDGIR :  32
LE_AAL3771 : -------------------------------------MKANPPCLIL------LLLISATVLT-VHSDDDDDDVS :  31
Os3bglu8   : -------------------------------------MGCAPAAHYLPGGGRRRLLVVVVALV----VLDRAGAR :  34
Os3bglu7   : -------------------------------------MAARRANCAL-------VLVLALALL----AARDAGAA :  27
Os12bglu38 : ---------------------------------------MNMPLLLL------IAIVVVSLS------------H :  18
DL_CAB3885 : ------------------------------------------------------------MTNESNHTDHN---- :  11
OE_AAL9361 : ---------------------------------------------------MDIQSNVLTITSGSTPTDTSSNGQ :  24
CR_AAF2880 : --------------------------------MGSKDDQSLVVAISPAAEPNGNHSVPIPFAYPSIPIQPRK--- :  40
RS_AAF0367 : ------------------------------------------------------------MATQSSAVIDS---- :  11
Os11bglu37 : -------------------------------------------------MGIRMGRRLLLITLLLGALLCNN--- :  23
Os11bglu35 : -------------------------------------------------MGIRMGRRLLLITLLLGALLCNN--- :  23
Os9bglu30  : -------------------------------------------------MGIRMGRR-LLFTLFLGALFCN---- :  21
Os9bglu29  : ----------------------------------------------MAWLGIGMGRQ-IVPVLVFVAVLCS---- :  24
Os8bglu28  : -----------------------------------------------------MDRRLLLSALLFIALACSSN-- :  20
Os8bglu27  : -------------------------------------------------------MRWLLLALFLVALVSNGA-- :  18
CS_BAC7865 : ------------------------------------------------MMAAKGSVVVGVLAIVAYALVVSEVA- :  26
Os4bglu10  : ---------------------------------------------MAVAGAMVMSGGVLLL---LLAFTCAAYN- :  26
Os4bglu11  : ---------------------------------------------MAVAGAMVMSGALLLLH--LLAFTCVACN- :  27
Os4bglu9   : ---------------------------------------------MAVAGAVAMSGGILLL---LLLLLAAACV- :  26
Os4bglu13  : ---------------------------------------------MAAAGEVVMLGGILLPL--LLVVAVS---- :  24
Os4bglu12  : ---------------------------------------------MAAAG--AMPGGLLLTF--LLLAVVASGAY :  26
Os6bglu24  : ---------------------------------------------MELLW-------LLLLL--LMASSTSSRSE :  21
PS_AAL3907 : ---------------------------------------------MALQFRSLLLCVVLLLL--GFALATTNAAG :  28
PA_AAA9116 : ----------------------------------------------------------LLLL--GFALANTNAAR :  15
CA_CAC0820 : --------------------------------------------------------------------------- :   -
DC_AAF0400 : -------------------------------------------------MLAMTSKAILLLG--LLALVSTSASI :  24
CS_BAA1183 : MAAQLGLPLVSCHRGASQAASSSAHLVPGASAIMQAGNRRQKMRAPALRDRVVFARVVPVDGSVGFAGSSTEQET :  75
TR_CAA4005 : ------------------------------------------------------------------LLSITTTHI :   9
PT_BAA7870 : ---------------------------------------------------------MAITSIAHLRVVNANMSI :  18
CP_AAG2589 : ----------------------------------------------------------------MDDVVNNNS-- :   9
ME_AAB7138 : --------------------------------------------------------MMIFLTILAVN-------- :  11
HB_AAO4926 : -------------------------------------------------------MLVLLISFLALTKPAMADYD :  20
BN_CAA5791 : ---------------------------------------------------MKFPLLGLLLLVTLVGSPTRAEEG :  24
RSMyr_BAB1 : ---------------------------------------------------MKL-LHGLALVFLLAAASCKAYEE :  23
BJMyr_AAG5 : ---------------------------------------------------MKL-LYGLALVFLLAAASCKADEE :  23
ZM_AAB0326 : ----------------MAPLLAAAMNHAAAHPGLRSHLVGPNNESFSRHHLPSSSPQSSKRR-CNLSFTTRSARV :  58
ZM_AAD0985 : ----------------MAPLLAAAMNH-AAHPVLRSHLG-PNNESFSRHHL-SSSPQSSKRR-FNLSFTPRSARV :  55
SB_AAC4917 : ----------------MALLLASAINHTAHPAGLRSHPN---NESFSRHHLCSSPQNISKRR-SNLSFRPRAQTI :  55
AS_CAA5519 : ----------------MALLCSALSNS--THPSFRSHIG---ANSENLWHLSADPAQKSKRR-CNLTLSSRAARI :  53
SC_AAG0061 : ----------------MALLVGGTLNP-TTHLSLRSRAG---RNSENVWLRSAASSQTSKGRFCNLTVRAGTPSK :  55
Osbglu39   : --------------------------------------------------------------------------- :   -
Os11bglu36 : ----------------------------------------MPLPAFVAAAARLAVLVAAAATAANAASYARYRRR :  35


                80         *       100         *       120         *       140         *
Os9bglu33  : -----------AREASALTRHDFP---------EGFVFGAGSSAFQVEGAAAE---------------------- :  57
Os9bglu32  : -----------ARDASALTRHDFP---------EGFVFGAGTSAFQVEGAAAE---------------------- :  57
Os9bglu31  : -----------SSTAAGITRADFP---------PEFIFGAGSSAYQVEGAFAE---------------------- :  56
Os1bglu2   : -------------AVG-YTRSDFP---------RDFVFGAATSAYQYDGAAAE---------------------- :  89
Os1bglu3   : -------------VLGGYTRNDFP---------ADFVFGAATSAYQYEGAAAE---------------------- :  50
Os1bglu5   : -----------PAVLG-YTRGDFP---------EDFVFGSATSSYQYEGGFDE---------------------- :  53
Os5bglu22  : WRSG-EAAAAAARALN-FTRQDFP---------GEFVFGAGTSAYQYEGATDE---------------------- :  68
Os5bglu23  : -------EATAEAALN-FTRQDFP---------GDFVFGAGTSAYQYEGATGE---------------------- :  59
Os5bglu20  : ----------QGVSSLQFRRDDFP---------DGFAFGAGTAAYQYEGAAAE---------------------- :  59
Os5bglu21  : ----------QGMSSLQFTRDDFP---------HDFAFGAGTSAYQYEGGAAE---------------------- :  63
Os5bglu19  : ----------QGVRSLQFTRDDFP---------DGFTFGAGTAAFQYEGAAAE---------------------- :  60
GM_AAL9211 : -----------VLGADNYSRDDFP---------LDFVFGSGTSAYQVEGAANK---------------------- :  61
Os10bglu34 : G---------------GLTRKSFP---------NGFVFGTASSAYQYEGAVKE---------------------- :  59
Os3bglu6   : G---------------GLTRGSFP---------EGFVFGTASAAYQYEGAVKE---------------------- :  71
Os6bglu25  : -------------------RADFP---------PGFIFGTASSAYQYEGAVNE---------------------- :  46
Os4bglu18  : ----------------AIHRSDFP---------ASFLFGTATSSYQIEGAYLE---------------------- :  53
Os4bglu14  : ----------------AVDRSQFP---------PDFLFGTSSSAYQVEGGYLE---------------------- :  50
Os4bglu16  : R---------------GLRRDDFP---------PGFLFGAATSAYQIEGAYLD---------------------- :  54
PC_AAC6961 : A---------------RLDRNNFP---------SDFMFGTASSAYQYEGAVRE---------------------- :  51
Os1bglu4   : A---------------EVTRGDFP---------DGFVFGVATSAYQIEGARRE---------------------- :  36
HB_AAP5105 : K---------------EVSRSDFP---------PNFLFGVATSAYQIEGGCRE---------------------- :  44
Os7bglu26  : ---------PEIYDAGGLSRRAFP---------EGFVFGTAASAYQVEGMAKQ---------------------- :  66
HV_AAA8733 : P-------NPEIGNTGGLSRQGFP---------AGFVFGTAASAYQVEGMARQ---------------------- :  65
Os1bglu1   : GGAG-ADHQEAAGITGGLSRRSFP---------AGFVFGTAASAYQVEGMALK---------------------- :  75
LE_AAL3771 : P-------ENTILDTGGLSRESFP---------KGFTFGTATSAYQVEGSAST---------------------- :  68
Os3bglu8   : V-------RAADDDTGGLSRAAFP---------KGFVFGTATSAFQVEGMAAS---------------------- :  71
Os3bglu7   : A-------VPKPNWLGGLSRAAFP---------KRFVFGTATSAYQVEGMAAS---------------------- :  64
Os12bglu38 : G--------N--GEQTDLTRETFP---------AGFVFGTASSAYQVEGNALQ---------------------- :  52
DL_CAB3885 : ------------VTRASFNFSNG----------EKFVFGSATSAYQIEGCAME---------------------- :  42
OE_AAL9361 : AAK---------STKERIKRSDFP---------SDFVFGAATASYQVEGAWNE---------------------- :  59
CR_AAF2880 : ------------HNKPIVHRRDFP---------SDFILGAGGSAYQCEGAYNE---------------------- :  72
RS_AAF0367 : ------------NDATRISRSDFP---------ADFIMGTGSSAYQIEGGARD---------------------- :  43
Os11bglu37 : ------------VAYAKFSRYSFP---------KDFIFGTGSAAYQYEGAYKE---------------------- :  55
Os11bglu35 : ------------VAYAKFSRYSFP---------KDFIFGTGSAAYQYEGAYKE---------------------- :  55
Os9bglu30  : ------------GVYAKFTRYSFP---------KDFIFGTGSAAYQYEGAYKE---------------------- :  53
Os9bglu29  : ------------GVDASFNRYSFP---------KDFIFGTGSAAYQYEGAAKE---------------------- :  56
Os8bglu28  : ------------RVHGALNRHSFP---------EGFLFGTGTSAYQYEGAVDK---------------------- :  52
Os8bglu27  : ------------AVHGAFNRFSFP---------EDFIFGTGSAAYQYEGAVNE---------------------- :  50
CS_BAC7865 : -I---A------AQISSFNRTSFP---------DGFVFGAASSAYQFEGAAKE---------------------- :  60
Os4bglu10  : -DA---------GELPPISRRSFP---------KGFIFGTSSSSYQFEGAAAK---------------------- :  60
Os4bglu11  : -GG---------SELPPISRRSFP---------KGFIFGTSSSSYQFEGGAVL---------------------- :  61
Os4bglu9   : -EA---------GELPPISRRSFP---------KGFIFGTSSASYQCEGGAAE---------------------- :  60
Os4bglu13  : ------------GEPPPISRRSFP---------EGFIFGTASSSYQYEGGARE---------------------- :  56
Os4bglu12  : NSA---------GEPP-VSRRSFP---------KGFIFGTASSSYQYEGGAAE---------------------- :  60
Os6bglu24  : MKA---------GEVI--RRSQFP---------EDFFFGTASSAYQYEGAVRE---------------------- :  54
PS_AAL3907 : TDPP-GVCTTL-NRTN--FDTLFP---------G-FTFGAATAAYQLEGAANI---------------------- :  67
PA_AAA9116 : TDPP-VVCATL-NRTN--FDTLFP---------G-FTFGTATASYQLEGAANI---------------------- :  54
CA_CAC0820 : --------------------------------------------------------------------------- :   -
DC_AAF0400 : DFAK-EVRETI-TEVPPFNRSCFP---------SDFIFGTASSSYQYEGEG------------------------ :  64
CS_BAA1183 : AVES-ATPTAV-PSKVVLGRSSFP---------RGFIFGAASAAYQVEGAWNE---------------------- : 117
TR_CAA4005 : HAFK-PLPISF-DDFSDLNRSCFA---------PGFVFGTASSAFQYEGAAFE---------------------- :  51
PT_BAA7870 : PLAR-LRVVNA-NISIPLKRTSFP---------KKFLFGAGSASYQYEGAAHI---------------------- :  60
CP_AAG2589 : --QR-MDMI---------RRSSFP---------EDFVFGTASSAYQYEGDVFK---------------------- :  41
ME_AAB7138 : ----------------IFRMTSFL---------ERLLLLTRSKVKQLQRVDHL---------------------- :  39
HB_AAO4926 : GIP----A--------DFNRSYFP---------DDFIFGTATSAYQIEGAANI---------------------- :  52
BN_CAA5791 : PVCP-KTE--------TLSRASFP---------EGFMFGTATASYQVEGAVNE---------------------- :  59
RSMyr_BAB1 : ITCE-ENEPFTCNNTARLSSKNFP---------KDFIFGVASSAYQIEGGRGR---------------------- :  66
BJMyr_AAG5 : ITCE-QNEPFTCGNTDILSSKNFG---------KDFIFGVASSAYQIEGGRGR---------------------- :  66
ZM_AAB0326 : GSQ---NGVQMLSPSEIPQRDWFP---------SDFTFGAATSAYQIEGAWNE---------------------- :  99
ZM_AAD0985 : GNQ---NGVQLLSPSEIPRRDWFP---------SDFIFGAATSAYQIEGAWNE---------------------- :  96
SB_AAC4917 : SSES--AGIHRLSPWEIPRRDWFP---------PSFLFGAATSAYQIEGAWNE---------------------- :  97
AS_CAA5519 : SSAL--ESAKQVKPWQVPKRDWFP---------PEFMFGAASAAYQIEGAWNE---------------------- :  95
SC_AAG0061 : PSEPIGPVFTKLKPWQIPKRDWFS---------KDFLFGASTSAYQIEGAWNE---------------------- :  99
Osbglu39   : ------------------MNTSLP---------ANFLWGNSVSSMQTEGAWNE---------------------- :  26
Os11bglu36 : HLRRIPSPIDESADPLADFR-AFPSSDADDSEEDNFFFGLATAPAHVEDRLEDAWLQFATETSCDDNGNVRDQRP : 109
                                r  fp           f  g      q  g

                    160         *       180         *       200         *       220
Os9bglu33  : ----------DGRKPSIW---------D----------------TFINQG--YMPDG-SNADVSADQYHHYKED- :  93
Os9bglu32  : ----------DGRKPSIW---------D----------------TFTHQG--YSPGG-AIADVSADQYHLYKED- :  93
Os9bglu31  : ----------DGRKPSIW---------D----------------TFSHSG--YSVDG-ATGDVTADQYHKYKED- :  92
Os1bglu2   : ----------DGRSPTIW---------D----------------TFAHEGQ-KTKDK-GTGDVAADGYHKYKGD- : 126
Os1bglu3   : ----------DGRGASIW---------D----------------TFTHAG--KMKDK-STGDVASDGYHKYKGD- :  86
Os1bglu5   : ----------DGRSPSNW---------D----------------IFTHQG--KMPGR-STADVAADGYHKYKDD- :  89
Os5bglu22  : ----------DGRSPSIW---------D----------------TFTHAG--KMPDK-STGDMGAGGYHKYKED- : 104
Os5bglu23  : ----------DGRTPSIW---------D----------------TFTHSG--RMADN-STGDRAAAGYHKYKED- :  95
Os5bglu20  : ----------DGRTPSIW---------D----------------TYTHSG--RHPED-GTGDVASDGYHKYKED- :  95
Os5bglu21  : ----------DGRTPSIW---------D----------------TYTHSG--RHPED-ETGDVASDGYHKYKED- :  99
Os5bglu19  : ----------DGRTPSIW---------D----------------TYAHSW--RNPGG-ETGDVACDGYHKYKED- :  96
GM_AAL9211 : ----------DGRTPSIW---------D----------------TFAYAG---YAHG-ENGDVACDGYHKYKED- :  96
Os10bglu34 : ----------DGRGPTIW---------D----------------KFAHTFG-KIIDF-SNADVAVDQYHRFEED- :  96
Os3bglu6   : ----------DGRGQTIW---------D----------------TFAHTFG-KITDF-SNADVAVDQYHRFEED- : 108
Os6bglu25  : ----------GQRGPTIW---------D----------------TLTKRPG-RVIDF-SNADVAVDHYHRYKED- :  83
Os4bglu18  : ----------GNKSLSNW---------D----------------VFTHLPG-NIKDG-SNGDIADDHYHRYEED- :  90
Os4bglu14  : ----------GNKGLSNW---------D----------------VFTHKQG-TIEDG-SNGDTANDHYHRYMED- :  87
Os4bglu16  : ----------DNKGLNNW---------D----------------VFTHTQAGRISDG-RNGDVADDHYHRYTED- :  92
PC_AAC6961 : ----------DGKGPSTW---------D----------------ALTHMPG-RIKDS-SNGDVAVDQYHRYMED- :  88
Os1bglu4   : ----------GGKGDNIW---------D----------------VFTENKE-RILDG-SSGEVAVDHYHRYKED- :  73
HB_AAP5105 : ----------GGRGPSIW---------D----------------AFSHTKG-TILDG-SNGDVAVDHYHRYKED- :  81
Os7bglu26  : ----------GGRGPSIW---------D----------------AFIEKPG-TIPNN-ATADVTVDEYHRYKED- : 103
HV_AAA8733 : ----------GGRGPCIW---------D----------------AFVAIQG-MIAGN-GTADVTVDEYHRYKED- : 102
Os1bglu1   : ----------DGRGPSIW---------D----------------AFVKTPG-EIANN-ATADVTVDEYHRYKED- : 112
LE_AAL3771 : ----------EGRGPSIW---------D----------------TFLKIPG-LEPNN-ANGEIAVDQYHRYKED- : 105
Os3bglu8   : ----------GGRGPSIW---------D----------------PFVHTPG-NIAGN-GNADVTTDEYHRYKED- : 108
Os3bglu7   : ----------GGRGPSIW---------D----------------AFAHTPG-NVAGN-QNGDVATDQYHRYKED- : 101
Os12bglu38 : ----------YGRGPCIW---------D----------------TFLMQPG-VTPDN-STANVTVDEYHRYMDD- :  89
DL_CAB3885 : ----------FGKGLSVW---------D----------------TWTLDKPGHIIDG-TNGNVAANQYHLFKED- :  80
OE_AAL9361 : ----------GGKGMSNW---------D----------------YFTQSQPGGISDF-SNGTIAIDHYNMFKDD- :  97
CR_AAF2880 : ----------GNRGPSIW---------D----------------TFTNRYPAKIADG-SNGNQAINSYNLYKED- : 110
RS_AAF0367 : ----------GGRGPSIW---------D----------------TFTHRRPDMIRGG-TNGDVAVDSYHLYKED- :  81
Os11bglu37 : ----------GGKGPSIW---------D----------------TFTH-VPGKILNN-DTGDVASDFYHRYKED- :  92
Os11bglu35 : ----------GGKGPSIW---------D----------------TFTH-IPGKILNN-DTGDVANDFHPDTRED- :  92
Os9bglu30  : ----------GGKGPSVW---------D----------------NFTH-IPGKILNN-DNGDVANDFYHRYKED- :  90
Os9bglu29  : ----------GGRGPSVW---------D----------------TFSH-IPGKILNG-DTGDVADDFYHRYKED- :  93
Os8bglu28  : ------------RGQNIW---------D----------------TFSR-IPGKIADG-SNADIANDFYHRYKED- :  87
Os8bglu27  : ----------GGRGPSIW---------D----------------TYAH-IPGKVEDG-SNGDVAVDFYHRYKED- :  87
CS_BAC7865 : ----------GGKGPNIW---------D----------------TFTHEFPGKISNG-STGDVADDFYHRYKED- :  98
Os4bglu10  : ----------GGRGPSIW---------D----------------TFTHQYPDKITDK-SNGDGACNSYHLYKED- :  98
Os4bglu11  : ----------GGRGPSIW---------D----------------TFTHQSPDKITDR-SNGDVACDSYHLYKED- :  99
Os4bglu9   : ----------GGRGSSIW---------D----------------TFTYQHPDKIADK-SNGDVADNTYHLYKED- :  98
Os4bglu13  : ----------GGRGPSIW---------D----------------TFTHQHPDKIADK-SNGDVAADSYHLYKED- :  94
Os4bglu12  : ----------GGRGPSIW---------D----------------TFTHQHPEKIADR-SNGDVASDSYHLYKED- :  98
Os6bglu24  : ----------GGRGPSIW---------D----------------TFTHNHPEKIANG-SNGDIAIDSYHRYKED- :  92
PS_AAL3907 : ----------DGRGPSVW---------D----------------NFTHEHPEKITDG-SNGDVAIDQYHRYKED- : 105
PA_AAA9116 : ----------DGRGPSIW---------D----------------AFTHNHPEKITDG-SNGDVAIDQYHRYKED- :  92
CA_CAC0820 : --------------------------------------------TYTHNHPERIADG-SNGDVAIDQYHRYKED- :  29
DC_AAF0400 : ------------RVPSIW---------D----------------NFTHQYPEKIADR-SNGDVAVDQFHRYKKD- : 100
CS_BAA1183 : ----------GGRGPSIW---------D----------------TFTHDHPEKIADH-SNGDKATDSYKKYKED- : 155
TR_CAA4005 : ----------DGKGPSIW---------D----------------TFTHKYPEKIKDR-TNGDVAIDEYHRYKED- :  89
PT_BAA7870 : ----------DGRGLSVW---------D----------------VFTKEHPEKIADQ-SNGDVAQDFYHRYKED- :  98
CP_AAG2589 : ----------DGKGPSTW---------D----------------AYTHQHPERIADH-SNGDIAVDEYHRYKED- :  79
ME_AAB7138 : ----------VFG---------------------------------TYFPRNRIIDG-RNGDVAVDFYNRYIED- :  69
HB_AAO4926 : ----------LGRGPSVW---------D----------------TFTHESPKRIKDQ-SNGDVAVDFYNRFEED- :  90
BN_CAA5791 : ----------GCRGPSLW---------D----------------IYTKKFPHRVKN--HNADVAVDFYHRFRED- :  96
RSMyr_BAB1 : ----------GVN---IW---------D----------------GFSHRYPEKAGSDLKNGDTSCESYTRWQKD- : 102
BJMyr_AAG5 : ----------GVN---VW---------D----------------GFSHRYPEKSGSDLKNGDTSCESYTRWKKD- : 102
ZM_AAB0326 : ----------DGKGESNW---------D----------------HFCHNHPERILDG-SNSDIGANSYHMYKTD- : 137
ZM_AAD0985 : ----------DGKGESNW---------D----------------HFCHNFPERIMDG-SNADIGANSYHMYKTD- : 134
SB_AAC4917 : ----------DGKGPSTW---------D----------------HFCHNFPEWIVDR-SNGDVAADSYHMYAED- : 135
AS_CAA5519 : ----------GGKGPSSW---------D----------------NFCHSHPDRIMDK-SNADVAANSYYMYKED- : 133
SC_AAG0061 : ----------DGKGPSTW---------D----------------HFCHTYPERISDG-TNGDVAANSYHMYEED- : 137
Osbglu39   : ----------GGKGMSVY---------D----------------IREAGEN--ISDW----KVATDSYHRYRED- :  59
Os11bglu36 : VDALMASAAGDGGSQQSWRSTGGENIGDREQRKPLRVAMEAMLRGFEILAESGESAGGDNCSHNVAAWHNVPCPQ : 184
                        g               d                                        h   ed

                 *       240         *       260         *       280         *       300
Os9bglu33  : ------------VKLMYDMGLDAYRFSIAWPRLIPDG---R--GEINPKGLEYYNNLIDELIMHG--------IQ : 143
Os9bglu32  : ------------VKLMYDMGLDAYRFSIAWPRLIPDG---R--GEINPKGLEYYNNLIDELIMHG--------IQ : 143
Os9bglu31  : ------------VKLLQDMGVDAYRMSISWSRLIPDG---R--GAVNPKGLEYYNNLIDELLSHG--------IQ : 142
Os1bglu2   : ------------VKLMAETGLEAYKFSISWSRLIPNG---R--GAVNQEGLKYYNNVIDELAKRG--------IQ : 176
Os1bglu3   : ------------VKLMTETGLEAYRFSISWSRLIPSG---R--GAVNQQGLKYYNNIIDELTKRG--------IQ : 136
Os1bglu5   : ------------LKLMVDTNLEAYRLSISWSRIIPNG---R--GDVNPKGLQYYNDIIDGLVKNG--------IQ : 139
Os5bglu22  : ------------VKLMSDTSLEAYRFSISWSRLIPRG---R--GPVNPKGLEYYNSLIDELVERG--------IE : 154
Os5bglu23  : ------------VKLMSDTGLEAYRFSISWSRLIPRG---R--GPINPKGLEYYNDLIDKLVKRGEICDCSMGIE : 153
Os5bglu20  : ------------VKLMTEIGLEAYRFTISWSRLIPSG---R--GAVNPKGLQFYNNMINELVKAG--------IQ : 145
Os5bglu21  : ------------VKLMSEIGLEAYRFTISWSRLIPSG---R--GAVNLKALQFYNSMINELVKAG--------IQ : 149
Os5bglu19  : ------------VMLMNETGLEAYRFTISWSRLIPSG---R--GAVNPKGLQFYNSMINELVKAG--------IQ : 146
GM_AAL9211 : ------------VQLMLETGLDAYRFSISWSRLLPNG---R--GPVNPKGLQYSNNLINELISNG--------IQ : 146
Os10bglu34 : ------------IQLMADMGMDAYRFSIAWSRIFPNG---T--GEVNQAGIDHYNKLINALLAKG--------IE : 146
Os3bglu6   : ------------IQLMADMGMDAYRFSIAWSRIYPNG---V--GQVNQAGIDHYNKLIDALLAKG--------IQ : 158
Os6bglu25  : ------------VELMNDIGMDAYRFSISWSRIFPNG---T--GEPNEEGLSYYNSLIDALLDKG--------IE : 133
Os4bglu18  : ------------VELMNSLGVNAYRFSISWSRILPKG---RF-GGVNPAGIDFYNKLIDSILLKG--------IQ : 141
Os4bglu14  : ------------IELMHSLGVNSYRFSISWARILPKG---RF-GDVNPDGVAFYNALIDGLVQKG--------IQ : 138
Os4bglu16  : ------------VDILHNLGVNSYRFSISWARILPRG---RL-GGVNSAGIAFYNRLINALLQKG--------IQ : 143
PC_AAC6961 : ------------IELMASLGLDAYRFSISWSRILPEG---R--GEINMAGIEYYNNLIDALLQNG--------IQ : 138
Os1bglu4   : ------------IELMASLGFRAYRFSISWPRIFPDG---LG-KNVNEQGVAFYNDLINFMIEKG--------IE : 124
HB_AAP5105 : ------------IELIAKLGFDAYRFSLSWSRIFPDG---LG-TKVNEEGIAFYNNIINALLEKG--------IE : 132
Os7bglu26  : ------------VNIMKNMGFDAYRFSISWSRIFPNG---T--GMVNQEGVDYYNRLIDYMVKKG--------IK : 153
HV_AAA8733 : ------------VGIMKNMGFDAYRFSISWSRIFPDG---T--GKVNQEGVDYYNRLIDYMLQQG--------IT : 152
Os1bglu1   : ------------VNIMKSMGFDAYRFSISWSRIFPTG---T--GKVNWKGVAYYNRLINYMLKIG--------IT : 162
LE_AAL3771 : ------------IDLMAKLNFEAYRFSISWSRIFPNG---T--GKVNWKGVAYYNRLIDYMLKRG--------IT : 155
Os3bglu8   : ------------VDLLKSLNFDAYRFSISWSRIFPDG---E--GKVNTEGVAYYNNLIDYVIKQG--------LI : 158
Os3bglu7   : ------------VNLMKSLNFDAYRFSISWSRIFPDG---E--GRVNQEGVDYYNNLINYLLQKG--------IT : 151
Os12bglu38 : ------------VDNMVRVGFDAYRFSISWSRIFPSG---L--GKINKDGVDYYHRLIDYMLANN--------II : 139
DL_CAB3885 : ------------MKIMKRAGLEAYRFSISWPRILPGG---KLSTGVNKEGIKYYNDLIDAIIAEG--------MQ : 132
OE_AAL9361 : ------------VVVMKKLGLKAYRFSLSWPRILPGG---RLCHGVSKEGVQFYNDLIDALLAAD--------IE : 149
CR_AAF2880 : ------------IKIMKQTGLESYRFSISWSRVLPGG---NLSGGVNKDGVKFYHDFIDELLANG--------IK : 162
RS_AAF0367 : ------------VNILKNLGLDAYRFSISWSRVLPGG---RLSGGVNKEGINYYNNLIDGLLANG--------IK : 133
Os11bglu37 : ------------VNLLKDMNMDAFRFSIAWTRILPNG---SLSGGINREGVAFYNSLINDVIAKG--------MI : 144
Os11bglu35 : ------------VNLLKDMNMDAFRFSIAWTRILPNG---SLSGGINREGVAFYNSLINDVIAKG--------MI : 144
Os9bglu30  : ------------VSLLKDMNMDAFRFSIAWTRILPNG---SLSGGINKEGVAFYNSLINDVIAKG--------MI : 142
Os9bglu29  : ------------VNLLKDMNMDAFRFSISWSRILPNG---TLSGGVNKEGVAFYNNLINEIIAKG--------MK : 145
Os8bglu28  : ------------LNLITAMNMDSFRFSIAWSRILPNG---TISGGINKEGVEFYNSLINEVIAKG--------LK : 139
Os8bglu27  : ------------LNFVTDMNMDAFRFSIAWSRILPNG---TISGGINKEGIAFYNSLINEVISRG--------LK : 139
CS_BAC7865 : ------------VKVLKFIGLDGFRMSISWARVLPRG---KLSGGVNKEGIAFYNNVINDLLSKG--------IQ : 150
Os4bglu10  : ------------VRIMKEMGMDAYRFSISWSRILPNG---SLSGGVNREGINYYNNLINELLSKE--------VQ : 150
Os4bglu11  : ------------VRSMKEMGMDAYRFSISWSRILPS----ALSGGVNREGISYYNNLINELLSKG--------VQ : 150
Os4bglu9   : ------------VHMMKEMGMDAYRFSISWSRILPNG---SLNGGVNIEGINYYNNLINELLLKG--------VQ : 150
Os4bglu13  : ------------VRIMKDMGVDAYRFSISWTRILPNG---SLSGGINREGISYYNNLINELLLKG--------VQ : 146
Os4bglu12  : ------------VRLMKDMGMDAYRFSISWTRILPNG---SLRGGVNKEGIKYYNNLINELLSKG--------VQ : 150
Os6bglu24  : ------------VGIMKGLGLNAYRFSVSWPRILPNG---KLSGGVNLEGIKYYNNLIDELISKG--------VE : 144
PS_AAL3907 : ------------VAIMKDMGFDAYRFSISWSRILPNG---TLSGGINKKGIEYYNNLTNELLSNG--------IE : 157
PA_AAA9116 : ------------VAIMKDMGLDAYRFSISWSRLLPNG---TLSGGINKKGIEYYNNLTNELIRNG--------IE : 144
CA_CAC0820 : ------------VQIIKDLNMDSYRFSISWSRILPKG---KLSGGKNPEGIQYYNNLINESLAHG--------IK :  81
DC_AAF0400 : ------------IAIMKDMNLDAYRMSISWPRILPTG---RVSGGINQTGVDYYNRLINESLANG--------IT : 152
CS_BAA1183 : ------------VKLLKDLGLDSYRFSISWSRILPKG---TLQGGINQEGIQYYNDLINELLKNG--------IR : 207
TR_CAA4005 : ------------IGIMKDMNLDAYRFSISWPRVLPKG---KLSGGVNREGINYYNNLINEVLANG--------MQ : 141
PT_BAA7870 : ------------IKSMKEMGLESFRFSISWSRILPNG---KISGGINKLGIKFYNNLIDELLANG--------IK : 150
CP_AAG2589 : ------------VALMKSIGFGVYRFSIARTRILPLG---KLSGGVNKDGIEYYHNLIDELLANG--------IK : 131
ME_AAB7138 : ------------IKNVKKMGFNAFRMSISWSRVIPSG---RRHEGVNEEGIQFYDDVINEIISNG--------LE : 121
HB_AAO4926 : ------------IKNVKDMGFDAFRFSISWSRVIPSG---RRHEGVNEGGIEFYNTVINETIKQG--------LR : 142
BN_CAA5791 : ------------IKLMKKLNTDALRLSIAWPRIFPHG---RMEKGNSKEGVQFYHDLIDELLKND--------LT : 148
RSMyr_BAB1 : ------------VDVMGEINATGYRFSFAWSRIIPKG---KVSRGVNQGGLDYYHNLIDALLEKN--------IT : 154
BJMyr_AAG5 : ------------VEIMGELNATGYRFSLAWSRIIPKG---KVSRGVNQGGLDYYHSLIDALLEKN--------IT : 154
ZM_AAB0326 : ------------VRLLKEMGMDAYRFSISWPRILPKG---TKEGGINPDGIKYYRNLINLLLENG--------IE : 189
ZM_AAD0985 : ------------VRLLKEMGMDAYRFSISWPRILPKG---TVEGGINQDGIDYYKRLINLLLENG--------IE : 186
SB_AAC4917 : ------------VRLLKEMGMDAYRFSISWPRILPKG---TLAGGINEKGVEYYNKLIDLLLENG--------IE : 187
AS_CAA5519 : ------------VRMLKEIGMDSYRFSISWPRILPKG---TLDGGINHEGIQYYNDLLDCLIENG--------IK : 185
SC_AAG0061 : ------------VKALKDMGMKVYRFSISWSRILPNG---T--GKPNQKGIDYYNNLINSLIRHG--------IV : 187
Osbglu39   : ------------FDLMQDLGMNCYRFQISWSRVCPQG---D--GAFNDEGIAFYDRFIDDLIARG--------IE : 109
Os11bglu36 : ERLRFWSDPDAELKLAKETGISVFRMGVDWARLMPEEPTEELKSSVNFAALERYRWIIQRVREYG--------MK : 251
                                     4f   w R6 P g      g  n  g6  y          g        6

                      *       320         *       340         *       360         *
Os9bglu33  : PHVTIYHFDLPQALQDEYGGILS---PRFIEDYSAYAEVCFKNFGDRVKHWATFNQPNIEPIGGFDAG-DRPPR- : 213
Os9bglu32  : PHVTIYHFDLPQALQDEYGGILS---PRFIEDYTAYAEVCFKNFGDRVKHWVTVNEPNIEPIGGYDAG-VQPPR- : 213
Os9bglu31  : PHVTIYHFDFPQALQDEYNGILS---PRFVEDFTAYADVCFKNFGDRVKHWSTVNEPNIEPIGGYDQG-ILPPR- : 212
Os1bglu2   : PHIMLCHLDLPQALEDEYDGWLS---PRIVDDFTAYADVCFREFGDRVLHWTTLAEPNIAALGGYDTG-VLSPG- : 246
Os1bglu3   : VHVMLYHLDLPQALEDEYAGWLS---PRIVEDFTAYADVCFREFGDRVSHWTILAEPNVAALGGYDTG-EFAPG- : 206
Os1bglu5   : VHIMLYQLDLPQVLEDEYDGWLS---PRILEDFKAYADVCFKEFGDRVAHWITIDEPNVASIGSYDSG-QLAPG- : 209
Os5bglu22  : IHVTLYHLDFPQILEDEYHGWLS---PRVIDDFTAYADVCFREFGDRVRHWTTMDEPNVLSIAAYDSG-AFPPC- : 224
Os5bglu23  : IHVTLYHLDFPQALQDEYNGWLS---PRIIEDFTAYADVCFREFGDLVRHWTTVGEPNVLSIAGYDSG-VIPPC- : 223
Os5bglu20  : IQVVLYHSDLPQSLQDEYGGWIN---PKIVDDFTAYADVCFREFGDRVAHWTTVLEPNVMAQGCYDTG-TLPPN- : 215
Os5bglu21  : IHVVMYHMDLPQSLQDEYGGWIS---PKIVDDFTAYADVCFREFGDRVVHWTTVLEPNAMAQAGYDMG-ILPPN- : 219
Os5bglu19  : IHAVLYHIDLPQSLQDEYGGWVS---PKVVDDFAAYADVCFREFGDRVAHWTTSIEPNVMAQSGYDDG-YLPPN- : 216
GM_AAL9211 : PHATLYNFDLPQVLEDEYGGWIS---RDIIRDFTYYAEVEFREFGDRVLYWTTVNEPNVFALGGYDQG-NSPPR- : 216
Os10bglu34 : PYVTLYHWDLPQALEDKYTGWLD---RQIINDYAVYAETCFQAFGDRVKHWITFNEPHTVAVQAYDSG-MHAPG- : 216
Os3bglu6   : PYVTLYHWDLPQALEDKYKGWLD---RQIVDDFAAYAETCFREFGDRVKHWITLNEPHTVAIQGYDAG-LQAPG- : 228
Os6bglu25  : PYVTLFHWDLPQALEDRYGGWLN---SEIIEDFVQYAFTCFKEFGDRVKHWITFNEPYNFAIDGYDLG-IQAPG- : 203
Os4bglu18  : PFVTLTHYDIPQELEDRYGAWLN---AEIQSDFGHFADVCFGAFGDRVKYWTTFNEPNVAVRHGYMLG-TYPPS- : 211
Os4bglu14  : PFVTICHYDIPHELDERYGGWLS---PEIQKDFSYFAEVCFKLFGDRIKFWTTFNQPNLSIKFSYMDG-FYSPG- : 208
Os4bglu16  : PFVTLNHFDIPHELETRYGGWLG---AAIREEFEYYSDVCFNAFGDRVRFWTTFNEPNLSTRHQYILG-EFPPN- : 213
PC_AAC6961 : PFVTLFHFDLPKALEDSYGGWLS---PQIINDFEAYAEICFRAFGDRVKYWATVNEPNLFVPLGYTVG-IFPPT- : 208
Os1bglu4   : PYATLYHWDLPHNLQQTVGGWLS---DKIVEYFALYAEACFANFGDRVKHWITINEPLQTAVNGYGIG-HFAPG- : 194
HB_AAP5105 : PYITLYHWDLPLHLQESMGGWLN---KEIVKYFAIYADTCFASFGDRVKKWITLNEPLQTAVNGFDTG-ILAPG- : 202
Os7bglu26  : PYANLYHYDLPLALHEQYLGWLS---PNIVEAFADYADFCFQTFGDRVKDWFTFNEPRCVAALGYDNG-FHAPG- : 223
HV_AAA8733 : PYANLYHYDLPLALHQQYLGWLS---PKIVGAFADYAEFCFKVFGDRVKNWFTFNEPRVVAALGYDNG-FHAPG- : 222
Os1bglu1   : PYANLYHYDLPEALEVQYGGLLN---RKIVEAFADYAEFCFKTFGDRVKNWMTFNEPRVVAALGYDDG-NFAPG- : 232
LE_AAL3771 : PYANLNHYDLPQALQDRYNGWLG---REVVKDFADYAEFCFKTFGDRVKNWFSFNEPRVVAALGYDNG-FFAPG- : 225
Os3bglu8   : PYVNLNHYDLPLALQKKYEGWLS---PKIVGVFSDYAEFCFKTYGDRVKNWFTFNEPRIVAALGHDTG-TDPPN- : 228
Os3bglu7   : PYVNLYHYDLPLALEKKYGGWLN---AKMADLFTEYADFCFKTFGNRVKHWFTFNEPRIVALLGYDQG-TNPPK- : 221
Os12bglu38 : PYVVLYHYDLPQVLHDQYKGWLH---PRIVRDFVRFADFCFKTYGHKVKNWFTINEPRMMANHGYGDG-FFPPG- : 209
DL_CAB3885 : PYVTLFHWDLPLALELEYGGFLDKD-KRIVEHFRDYAELCFWEFGDRVKHWITINEAWSYTVEGYVNG-TCPPG- : 204
OE_AAL9361 : PYITIFHWDIPQCLQLEYGGFLH---ERVVKDFIEYSEICFWEFGDRVKYWITLNEPWSFTVQGYVAG-AFPPN- : 219
CR_AAF2880 : PFATLFHWDLPQALEDEYGGFLS---DRIVEDFTEYAEFCFWEFGDKVKFWTTFNEPHTYVASGYATG-EFAPG- : 232
RS_AAF0367 : PFVTLFHWDVPQALEDEYGGFLS---PRIVDDFCEYAELCFWEFGDRVKHWMTLNEPWTFSVHGYATG-LYAPG- : 203
Os11bglu37 : PFVTIFHWDTPQALESKYGGFLS---ENIVKDYVDFAEVCFREFGDRVKYWTTFNEPFTYNAYGYGKG-VFAPG- : 214
Os11bglu35 : PFVTIFHWDTPQALESKYGGFLS---ENIVKDYADFAEVCFHEFGDRVKYWTTFNEPFTYSAYGYGGG-VFASG- : 214
Os9bglu30  : PFVTIFHWDTPLALESKYGGFLS---EDIVKEYVDFAEVCFREFGDRVKYWFTFNEPFTYSAYGYGKG-VFAPG- : 212
Os9bglu29  : PFVTIFHWDTPQALESKYGGFLS---ENIIKDYVDFAEVCFREFGDRVKFWATFNEPWTYCSQGYGTG-IHALG- : 215
Os8bglu28  : PFVTIFHFDTPQALEDKYGGFLS---ENIVKDYVDYADLCFSLFGDRVKLWNTFNEPTIFCMNGYATG-IMAPG- : 209
Os8bglu27  : PFVTIFHFDTPQALEDKYRSFLS---ENIVKDFVDYADVCFREFGDRVKSWNTFNEPMIFCAGGYGSG-TKAPG- : 209
CS_BAC7865 : PFITIFHWDLPQALEDEYGGFLS---PHIVNDFRDFAELCFKEFGDRVKHWITMNEPWSYSYGGYDAG-LLAPG- : 220
Os4bglu10  : PFATLFHFDTPQALEDKYKGFLS---PNIINDYKDYAEICFKEFGDRVKHWITFNEPWNFCSMGYASG-TMAPG- : 220
Os4bglu11  : PFVTLFHWDSPQALEDKYKGFLS---PNIINDYKEYAETCFKEFGDRVKHWITFNEPWTFCSMGYASG-IMAPG- : 220
Os4bglu9   : SFVTLFHYDTPQALEDKYNGFLS---PNIINDYKDYAEICFKEFGDRVKHWITFNEPWIFCSKAYASG-TYAPG- : 220
Os4bglu13  : PFVTLFHWDSPQALEDKYNGFLS---PNIINDYKEYAETCFKEFGDRVKHWITFNEPLSFCVAGYASGGMFAPG- : 217
Os4bglu12  : PFITLFHWDSPQALEDKYNGFLS---PNIINDFKDYAEICFKEFGDRVKNWITFNEPWTFCSNGYATG-LFAPG- : 220
Os6bglu24  : PFVTLFHWDSPQALEQQYGGFLS---NLIVEDFRDYADICFREFGDRVKYWITFNEPWSFSIGGYSNG-ILAPG- : 214
PS_AAL3907 : PLVTLFHWDVPQALVDEYGGLLS---PRIVDDFEAYANVCYNEFGDRVKRWTTLNEPYTVSHHGYTIG-IHAPG- : 227
PA_AAA9116 : PLVTLFHWDVPQALEEEYGGVLS---PRIVYDFKAYAELCYKEFGDRVKHWTTLNEPYTISNHGYTIG-IHAPG- : 214
CA_CAC0820 : PYITLFHWDLPQALEDEYGGFLN---SSIINDFRDYADLCFHEFGDRVKDWVTFNEPWMFSNGGYAVG-SLAPG- : 151
DC_AAF0400 : PFVTIFHWDLPQALEDEYGGFLN---HSVVNDFQDYADLCFQLFGDRVKHWITLNEPSIFTANGYAYG-MFAPG- : 222
CS_BAA1183 : PMVTLFHWDVPQALEDSYKGFRS---SEIVNDFKDYADICFKEFGDRVKHWITLNEPWSLSTMGYAFG-RHAPG- : 277
TR_CAA4005 : PYVTLFHWDVPQALEDEYRGFLG---RNIVDDFRDYAELCFKEFGDRVKHWITLNEPWGVSMNAYAYG-TFAPG- : 211
PT_BAA7870 : PLVTIYHWDLPQALQDEYGGFLS---PKIVDDFLEYANLVFKEFGDRVKHWATLNEPNIMTQQGYVFG-AHAPG- : 220
CP_AAG2589 : PYVTLFHWDVPEALEIEYGGFLN---RQIVEHFQEFAELCFKEFGKKVKHWITLNEQFIFTFKSYVIG-EYAVG- : 201
ME_AAB7138 : PFVTIFHWDTPQALQDKYEGFLS---RDIVYDYDQYADLLFERFGDRVKRWMTFNEPSAYVGFAHDDG-VFAPR- : 191
HB_AAO4926 : PFVTIFHWDTPQALEDKYGGFLS---RDIVKDFREYADLLFERFGDRVKHWMTFNEPWALSGFAYDYG-VFAPG- : 212
BN_CAA5791 : PLVTIFHWDMPADLEDEYGGFLS---ERVVPDFVEYANFTFHEYGDKVKNWITFNEPWVFSRSAYDVG-KKAPG- : 218
RSMyr_BAB1 : PFVTLFHWDLPQTLQDEYEGFLD---RQIIQDFKDYADLCFREFGGKVKHWITINQLYTVPTRGYAIG-TDAPG- : 224
BJMyr_AAG5 : PFVTLYHWDLPQTLQDEYEGFLD---RQIIQDFKDYADLCFKEFGGKVKHWITINQLFTVPTRGYALG-TDAPG- : 224
ZM_AAB0326 : PYVTIFHWDVPQALEEKYGGFLDKSHKSIVEDYTYFAKVCFDNFGDKVKNWLTFNEPQTFTSFSYGTG-VFAPG- : 262
ZM_AAD0985 : PYVTIFHWDVPQALEEKYGGFLDKTQKRIVNDYKNFAKVCFDNFGDKVKNWLTFNEPQTFTSFSYGTG-VFAPG- : 259
SB_AAC4917 : PYITIFHWDTPQALVDAYGGFLD---EEDYKDYTDFAKVCFEKFGKTVKNWLTFNEPETFCSVSYGTG-VLAPG- : 257
AS_CAA5519 : PYITLFHWDTPQALADEYKDFLD---RRIVKDYTDYATVCFEHFGDKVKNWFTFNEPHSFCGLGYGTG-LHAPGA : 256
SC_AAG0061 : PYVTIWHWDTPQALEDKYGGFLD---KQIVNDYKYFAELCFQSFGDRVKNWFTFNEPHTYCCFSYGEG-IHAPG- : 257
Osbglu39   : PMICLYHFDMPLALAQEYNGFND---RRVMEAFIRYGKKMIDCFGDRVKYWLTFNEQNIFHMPEAFRISGYLKGE : 181
Os11bglu36 : VMLTLFHHSLP-PWAGKYGGWKM---EKTVTYFMDFVRLVVDCVSNLVDYWVIFNEPHVFVMLTYCAG-AWPGGD : 321
             p   6 h d P  l   y g           d5  5a  c    g  6  W    2p          g    p

               380         *       400         *       420         *       440         *
Os9bglu33  : R-------------------------------------------------------------------------- : 214
Os9bglu32  : R-------------------------------------------------------------------------- : 214
Os9bglu31  : R-------------------------------------------------------------------------- : 213
Os1bglu2   : H-------------------------------------------------------------------------- : 247
Os1bglu3   : R-------------------------------------------------------------------------- : 207
Os1bglu5   : R-------------------------------------------------------------------------- : 210
Os5bglu22  : R-------------------------------------------------------------------------- : 225
Os5bglu23  : R-------------------------------------------------------------------------- : 224
Os5bglu20  : H-------------------------------------------------------------------------- : 216
Os5bglu21  : R-------------------------------------------------------------------------- : 220
Os5bglu19  : R-------------------------------------------------------------------------- : 217
GM_AAL9211 : R-------------------------------------------------------------------------- : 217
Os10bglu34 : R-------------------------------------------------------------------------- : 217
Os3bglu6   : R-------------------------------------------------------------------------- : 229
Os6bglu25  : R-------------------------------------------------------------------------- : 204
Os4bglu18  : R-------------------------------------------------------------------------- : 212
Os4bglu14  : R-------------------------------------------------------------------------- : 209
Os4bglu16  : H-------------------------------------------------------------------------- : 214
PC_AAC6961 : R-------------------------------------------------------------------------- : 209
Os1bglu4   : G-------------------------------------------------------------------------- : 195
HB_AAP5105 : KH--------EHSYT------------------------------------------------------------ : 209
Os7bglu26  : R-------------------------------------------------------------------------- : 224
HV_AAA8733 : R-------------------------------------------------------------------------- : 223
Os1bglu1   : R-------------------------------------------------------------------------- : 233
LE_AAL3771 : R-------------------------------------------------------------------------- : 226
Os3bglu8   : R-------------------------------------------------------------------------- : 229
Os3bglu7   : R-------------------------------------------------------------------------- : 222
Os12bglu38 : R-------------------------------------------------------------------------- : 210
DL_CAB3885 : RG--------ASAPSDDMALQTAEILRNRTLSTILDAIGSQAIAELKDIEKLIYTLIPNNIKDLISKFHIEEFVK : 271
OE_AAL9361 : RG--------VTPKDTEETQKHARLHRG----------GGKLLAAFK---------------------------- : 248
CR_AAF2880 : RG--------GAD-------------------------------------------------------------- : 237
RS_AAF0367 : RG--------RTSPEHVNHPTVQH--------------------------------------------------- : 219
Os11bglu37 : R-------------------------------------------------------------------------- : 215
Os11bglu35 : R-------------------------------------------------------------------------- : 215
Os9bglu30  : R-------------------------------------------------------------------------- : 213
Os9bglu29  : R-------------------------------------------------------------------------- : 216
Os8bglu28  : R-------------------------------------------------------------------------- : 210
Os8bglu27  : R-------------------------------------------------------------------------- : 210
CS_BAC7865 : R-------------------------------------------------------------------------- : 221
Os4bglu10  : R-------------------------------------------------------------------------- : 221
Os4bglu11  : R-------------------------------------------------------------------------- : 221
Os4bglu9   : R-------------------------------------------------------------------------- : 221
Os4bglu13  : R-------------------------------------------------------------------------- : 218
Os4bglu12  : R-------------------------------------------------------------------------- : 221
Os6bglu24  : R-------------------------------------------------------------------------- : 215
PS_AAL3907 : R-------------------------------------------------------------------------- : 228
PA_AAA9116 : R-------------------------------------------------------------------------- : 215
CA_CAC0820 : R-------------------------------------------------------------------------- : 152
DC_AAF0400 : R-------------------------------------------------------------------------- : 223
CS_BAA1183 : R-------------------------------------------------------------------------- : 278
TR_CAA4005 : R-------------------------------------------------------------------------- : 212
PT_BAA7870 : R-------------------------------------------------------------------------- : 221
CP_AAG2589 : RGAEWDKSH------FLGGN------------------------------------------------------- : 215
ME_AAB7138 : R-------------------------------------------------------------------------- : 192
HB_AAO4926 : R-------------------------------------------------------------------------- : 213
BN_CAA5791 : R-------------------------------------------------------------------------- : 219
RSMyr_BAB1 : R-------------------------------------------------------------------------- : 225
BJMyr_AAG5 : R-------------------------------------------------------------------------- : 225
ZM_AAB0326 : R-------------------------------------------------------------------------- : 263
ZM_AAD0985 : R-------------------------------------------------------------------------- : 260
SB_AAC4917 : R-------------------------------------------------------------------------- : 258
AS_CAA5519 : R-------------------------------------------------------------------------- : 257
SC_AAG0061 : R-------------------------------------------------------------------------- : 258
Osbglu39   : Q-------------------------------------------------------------------------- : 182
Os11bglu36 : PN--------AIEVAT------STLPTG----------------------------------------------- : 335


                    460         *       480         *       500         *       520
Os9bglu33  : ----------CSYPFG-------------------TNC--TGGD-SSTEPYIVAHHLLLAHASAVSIYRQKYQ-- : 255
Os9bglu32  : ----------CSYPFG-------------------TNC--TGGD-SSTEPYIVAHHLLLAHASAVSIYRRKYQ-- : 255
Os9bglu31  : ----------CSFPFGV------------------LSC--DNGN-STTEPYIVAHHLLLAHSSAVSLYREKYQ-- : 255
Os1bglu2   : ----------CSDPFGL------------------TEC--TVGN-STVEPYIAAHNMILTHAAVVRLYREKYQ-- : 289
Os1bglu3   : ----------CSDPFGV------------------TKC--TVGN-SSVEPYVAAHNMILTHAAVVRLYREKYQ-- : 249
Os1bglu5   : ----------CSDPFGI------------------RKC--TVGN-SSVEPYIAVHNMLLAHASVTKLYREKYQ-- : 252
Os5bglu22  : ----------CSPPFG-------------------ANC--TAGN-STVEPYVVAHNSILAHASVTRLYRDKYQ-- : 266
Os5bglu23  : ----------CSPPFG-------------------TSC--AAGD-STVEPYVAAHNSILAHASAVRLYWDKYQ-- : 265
Os5bglu20  : ----------CSYPFG-------------------SNC--TGGN-STVEPYLFIHHNLLAHASAVRLYREKYQ-- : 257
Os5bglu21  : ----------CSYPFG-------------------SNC--TAGN-SSVEPYLFIHHSLLAHASAVRLYREKYK-- : 261
Os5bglu19  : ----------CSYPFGR------------------SNC--TLGN-STVEPYLFIHHTLLAHASAVRLYREKHQ-- : 259
GM_AAL9211 : ----------CSPPFCA------------------TND--TMGN-STYEPYLAVHHILLSHSSAARLYWRKYR-- : 259
Os10bglu34 : ----------CSVLLH-------------------LYC--KKGN-SGTEPYIVAHNMILSHATVSDIYRKKYK-- : 258
Os3bglu6   : ----------CSVLLH-------------------LYC--KAGN-SGTEPYVVAHHFILAHAAAASIYRTKYK-- : 270
Os6bglu25  : ----------CSILSH-------------------VFC--REGK-SSTEPYIVAHNILLAHAGAFRAYEQHFK-- : 245
Os4bglu18  : ----------CSPPFG--------------------HC--ARGGDSHAEPYVAAHNVILSHATAIEIYKRKYQ-- : 253
Os4bglu14  : ----------CSEPFG--------------------KC--ALG-NSSIEPYVAGHNIILSHANAVSVYRNKYQ-- : 249
Os4bglu16  : ----------CSPPFG--------------------NC--SSG-DSRREPYAAAHNILLSHAAAVHNYKTNYQ-- : 254
PC_AAC6961 : ----------CAAPHANPL------------------C--MTGNCSSAEPYLAAHHVLLAHASAVEKYREKYQ-- : 252
Os1bglu4   : ----------CEGETA--------------------RC------------YLAAHYQILAHAAAVDVYRRKFK-- : 226
HB_AAP5105 : ------------------------------------------------EPFLASHHQILAHATAVSIYRSMYK-- : 234
Os7bglu26  : ----------CSG------------------------C--DAGGNSTTEPYLAAHHLILSHAAAVKRYREKYQ-- : 261
HV_AAA8733 : ----------CSK------------------------C--PAGGDSRTEPYIVTHNIILSHAAAVQRYREKYQ-- : 260
Os1bglu1   : ----------CTK------------------------C--TAG-NSATEPYIVAHHLILSHASAVQRYRHKYQ-- : 269
LE_AAL3771 : ----------CSKPFG--------------------NC--TEG-DSATEPYIVAHNLILCHASAAQRYREKYQ-- : 266
Os3bglu8   : ----------CTK------------------------C--AAGGNSATEPYIVAHNIILSHATAVDRYRNKFQ-- : 266
Os3bglu7   : ----------CTK------------------------C--AAGGNSATEPYIVAHNFLLSHAAAVARYRTKYQ-- : 259
Os12bglu38 : ----------CTG------------------------C--QPGGNSATEPYIAAHNLLLSHAAAVRTYRDKYQ-- : 247
DL_CAB3885 : NIIPEVLKQKCPQLVDFIVDILQSFHVDYKRTGMARAC--SNG-DPGTEPYIVAHNIILAHAAAVRLYKTKFQ-- : 341
OE_AAL9361 : -----------------------------------------YG-NPGTEPYKVAHNLILCHAHAVDIYRTKYQ-- : 279
CR_AAF2880 : ----------------------------------------GKG-EPGKEPYIATHNLLLSHKAAVEVYRKNFQ-- : 269
RS_AAF0367 : ---------RCSTVAPQ------------------CIC--STG-NPGTEPYWVTHHLLLAHAAAVELYKNKFQ-- : 262
Os11bglu37 : ----------CSPYVSK-------------------SC--GAG-DSSREPYLVTHHIHLSHAAADLPY----Q-- : 252
Os11bglu35 : ----------CAPYVSK-------------------SC--GAG-DSSREPYLVTHHIHLSHAAVVHLYRTRYQ-- : 256
Os9bglu30  : ----------CSSYVSK-------------------SC--GVG-DSSREPYLVAHHIHLSHAAAVQLYRTKYQ-- : 254
Os9bglu29  : ----------CSPYVST-------------------SC--AGG-DSSREPYLAAHHVILAHATAVHLYRTKYQ-- : 257
Os8bglu28  : ----------CSPYASA-------------------SC--AAGGDSGREPYVAGHHLLVAHAEAVRLYRARYR-- : 252
Os8bglu27  : ----------CSPYVSK-------------------KC--APG-DSGNEPYVAGHNLLLAHAEAVRLYRQKYQ-- : 251
CS_BAC7865 : ----------CSAFMAF--------------------C--PKG-NSGTEPYIVTHNLLLSHAAAVKLYKEKYQ-- : 261
Os4bglu10  : ----------CSSWEKG-------------------KC--RVG-DSGREPYTACHHQLLAHAETVRLYKEKYQFT : 264
Os4bglu11  : ----------CSSWEVG-------------------KC--RVG-DSGREPYTACHHQLLAHAETVRLYKEKYQ-- : 262
Os4bglu9   : ----------CSPWEMG-------------------KC--SVG-DSGREPYTACHHQLLAHAETVRLYREKYQ-- : 262
Os4bglu13  : ----------CSPWE-G-------------------NC--SAG-DSGREPYTACHHQLLAHAETVRLYKEKYQ-- : 258
Os4bglu12  : ----------CSPWEKG-------------------NC--SVG-DSGREPYTACHHQLLAHAETVRLYKAKYQ-- : 262
Os6bglu24  : ----------CSSQGKS-------------------GC--SKG-DSGREPYIVAHNQLLAHAAVVQIYREKYQ-- : 256
PS_AAL3907 : ----------CSSWYDP-------------------TC--LGG-DSSTEPYLVTHHLLLAHAAAVKLYKENYQ-- : 269
PA_AAA9116 : ----------CSSWYDP-------------------TC--LGG-DSGTEPYLVTHNLLLAHAAAVKLYREKYQ-- : 256
CA_CAC0820 : ----------CS---DP-------------------TC--LGG-NSGTEPYTVTHNQILAHAHAVRVYRTKYK-- : 190
DC_AAF0400 : ----------CSPSYNP-------------------TC--TGG-DAGTETYLVAHNLILSHAATVQVYKRKYQ-- : 264
CS_BAA1183 : ----------CSTWY---------------------GC--PAG-DSANEPYEVTHNLLLAHANAVKIYRDNYK-- : 317
TR_CAA4005 : ----------CSDWLKL-------------------NC--TGG-DSGREPYLAAHYQLLAHAAAARLYKTKYQ-- : 253
PT_BAA7870 : ----------CSHFEW--------------------NC--PAGN-SGTEPYIVGHHLLLCHAAAFQLYKQKYK-- : 261
CP_AAG2589 : ---------------------------------------------SGTEPYTVGHNLILAHAAAVNVYQTKYQ-- : 243
ME_AAB7138 : ----------CSSWVNR-------------------QC--LAGD-SATEPYIVAHNLLLSHAAAVHQYRKYYQ-- : 233
HB_AAO4926 : ----------CSSWVNR-------------------RC--RAGN-SATEPYIVAHHLLLSHAAVVQIYRENYQ-- : 254
BN_CAA5791 : ----------CSPYIKDFGH----------------LC--QDGR-SGFEAYVVSHNLLVSHAEAVDAFRKCE--- : 262
RSMyr_BAB1 : ----------CSPMVDTKH-----------------RC--YGGN-SSTEPYIVAHNQLLAHATVVDLYRTKY--- : 267
BJMyr_AAG5 : ----------CSPMVDSKH-----------------RC--YGGN-SSTEPYIVAHNELLAHAAVVDLYRKNY--- : 267
ZM_AAB0326 : ----------CSPGLD---------------------CAYPTG-NSLVEPYTAGHNILLAHAEAVDLYNKHY--- : 303
ZM_AAD0985 : ----------CSPGLD---------------------CAIPTG-NSLVEPYIAGHNILLAHAEAVDLYNKYY--- : 300
SB_AAC4917 : ----------CSPGVS---------------------CAVPTG-NSLSEPYIVAHNLLRAHAETVDIYNKYH--- : 298
AS_CAA5519 : ----------CSAGMT---------------------CVIPEE-DALRNPYIVGHNLLLAHAETVDVYNKFY--- : 297
SC_AAG0061 : ----------CSPGLD---------------------CAVPEG-DSLREPYTAGHHILLAHAEAVELFKAHYN-- : 299
Osbglu39   : ---------------------------------------------TLRELYELQHHAMVAHMSLTEYLHQTKP-- : 210
Os11bglu36 : ------------------------------------------------VYNQALHWMAIAHSEAYDYIHSKSK-- : 360
                       c                          c    g     ep    H     Ha

                 *       540         *       560         *       580         *       600
Os9bglu33  : ---------------------AIQGGQIGITLMVRWHEPYTD-KTADAAAAIRMNEFHIG----WFLHPLVHGDY : 304
Os9bglu32  : ---------------------AIQGGQIGITLLGWWYEPYTD-AVADAAAAIRMNEFHIG----WFMNPLVHGDY : 304
Os9bglu31  : ---------------------ATQGGQIGLTLLGWWYEPGTQ-DPEDVAAAARMNDFHIG----WYMHPLVYGDY : 304
Os1bglu2   : ---------------------ALQKGIVGINMFSLWSYPLTN-SIADLQAAQRYKDFSYG----WILHPLVFGDY : 338
Os1bglu3   : ---------------------TLQKGIVGINVLSLWSYPLTD-STADLQAAQRYKDFTYG----WILHPLVFGDY : 298
Os1bglu5   : ---------------------VAGKGIIGISVYTFWAYPLTN-STVDLEATKRCQDFIVH----WVLRPLVFGDY : 301
Os5bglu22  : ---------------------ATQEGFVGMNIYSFWNYPFSS-SSADIAATQRALDFMVG----WILDPLVYGDY : 315
Os5bglu23  : ---------------------AKQKGVVGTNIYSFWPYPLSR-SCADIDAVQRVLDFTIG----WILDPLVYGDY : 314
Os5bglu20  : ---------------------VAQKGIVGINIYSLWFYPLTD-SAEDIGATERAKQFMYG----WILHPLVFGDY : 306
Os5bglu21  : ---------------------VAQKGIIGINIYSMWFYPFTD-SAEDIGATERAKKFIYG----WILHPLVFGDY : 310
Os5bglu19  : ---------------------AAQKGVVGMNIYSMWFYPLTE-STEDIAATERVKDFMYG----WILHPLVFGDY : 308
GM_AAL9211 : ---------------------DKQHGFVGISIYTFGIFPQTN-TEKDRVASQRARDFFVG----WIMEPLQYGDY : 308
Os10bglu34 : ---------------------ASQNGELGISFDVIWYEPMSN-STADIEAAKRAQEFQLG----WFADPFFFGDY : 307
Os3bglu6   : ---------------------ATQNGQLGIAFDVMWFEPMSN-TTIDIEAAKRAQEFQLG----WFADPFFFGDY : 319
Os6bglu25  : ---------------------NEQGGLIGIALNSRWYEPFSN-ADEDTEAAARAMDFELG----WFLDPLMFGHY : 294
Os4bglu18  : ---------------------SKQRGMIGMVLYSTWYEPLRD-VPEDRLATERALAFETP----WFLDPLVYGDY : 302
Os4bglu14  : ---------------------GKQGGQIGIALSITWYEPFRN-TTIDLLAVKRALSFGAS----WFLDPILLGDY : 298
Os4bglu16  : ---------------------AKQGGSIGIVIAVKWYEPLTN-STEDVRAARRALAFEVD----WFLDPIFFGDY : 303
PC_AAC6961 : ---------------------KIQGGSIGLVISAPWYEPLEN-SPEERSAVDRILSFNLR----WFLDPIVFGDY : 301
Os1bglu4   : ---------------------AVQGGEVGLVVDCEWAEPFSE-KTEDQVAAERRLDFQLG----WYLDPIYFGDY : 275
HB_AAP5105 : ---------------------DNQGGEVGLVVDCEWAESNSD-KIEDKAAAAKRLEFQLG----WYLHPLYYGDY : 283
Os7bglu26  : --------------------LYQKGRIGILLDFVWYEPFSD-SNADRAAAQRARDFHLG----WFLDPIIHGRYP : 311
HV_AAA8733 : ---------------------PHQKGRIGILLDFVWYEPHSD-TDADQAAAQRARDFHIG----WFLDPITNGRY : 309
Os1bglu1   : ---------------------HIQKGKIGILLDFVWYEGLTN-STADQAAAQRSRDFHVG----WFLHPIIYGEY : 318
LE_AAL3771 : --------------------EKQKGKFGILLDFVWYEPLTK-GKADNYAAQRARDFHLG----WFLHPLVYGEYP : 316
Os3bglu8   : --------------------ASQKGKIGIVLDFNWYEPLTN-STEDQAAAQRARDFHVG----WFLDPLINGQYP : 316
Os3bglu7   : --------------------AAQQGKVGIVLDFNWYEALSN-STEDQAAAQRARDFHIG----WYLDPLINGHYS : 309
Os12bglu38 : --------------------AIQKGKIGILLDFVWYEPLTD-KEEDHAAAHRAREFTLG----WYLHPITYGHYP : 297
DL_CAB3885 : ---------------------AYQGGKIGMTNNVTYYLPYDEKYHEDVEASKRGVDFMLY----WFVEPIVTGKY : 391
OE_AAL9361 : ---------------------ESQGGKIGITNCISWNEPLTDS-QEDKDAATRGNDFMLG----WFVEPVVTGEY : 328
CR_AAF2880 : ---------------------KCQGGEIGIVLNSMWMEPLNET-KEDIDARERGLDFMLG----WFIEPLTTGEY : 318
RS_AAF0367 : ---------------------RGQEGQIGISHATQWMEPWDENSASDVEAAARALDFMLG----WFMEPITSGDY : 312
Os11bglu37 : ---------------------PTQKGQIGMVVMTHWFVPYDD-TVAHRGAVQRSLDFMFG----WFMDPLVHGDY : 301
Os11bglu35 : ---------------------PTQKGQIGMVVVTHWFVPYDD-TAADRGAVQRSLDFMFG----WFMDPLVHGDY : 305
Os9bglu30  : ---------------------PTQKGQIGMVVVTHWFVPYDN-SDADRGAVQRSLDFIYG----WFMDPIVHGDY : 303
Os9bglu29  : ---------------------PTQHGQIGITAVSHWFVPYND-TAADRRVVQRSLDFMYG----WFLDPIVHGDY : 306
Os8bglu28  : ---------------------AAHGGEVGITQVSHWFEPYDAGSAADRRARRRALDFMLG----WFMHPVAHGEY : 302
Os8bglu27  : ---------------------ATQKGQIGITQVSHWFVPYSD-AAADKHAVRRSLDFMYG----WFMDPIVFGDY : 300
CS_BAC7865 : ---------------------AYQKGQIGITLVTYWMIPYSN-SKADKDAAQRALDFMYG----WFIEPLSFGEY : 310
Os4bglu10  : EEAIRQSPFIRDNNLNRRSAKALQKGRIGIILNSEWFVPFSQ-SKSSNDAARRVLDFMLG----WFMDPLIRGDY : 334
Os4bglu11  : ---------------------ALQKGKIGIILNADWFVPLSQ-SKSSSDAARRALDFMLG----WFMDPLIRGDY : 311
Os4bglu9   : ---------------------ALQKGKIGIIVNSQWYVPFSQ-SKTNKDAARRVLDFVLG----WLMDPLIRGDY : 311
Os4bglu13  : ---------------------VLQKGKIGITLVSNWFVPFSR-SKSNIDAARRALDFMLG----WFMDPLIRGEY : 307
Os4bglu12  : ---------------------ALQKGKIGITLVSHWFVPFSR-SKSNNDAAKRAIDFMFG----WFMDPLIRGDY : 311
Os6bglu24  : ---------------------GGQKGKIGIAIVSNWMIPYED-SKEDKHATKRALDFMYG----WFMDPLTKGDY : 305
PS_AAL3907 : ---------------------ASQNGVIGITTVSHWFEPFSE-SQEDKDATSRALDFMYG----WFMDPLTRGDY : 318
PA_AAA9116 : ---------------------ASQEGVIGITVVSHWFEPASE-SQKDINASVRALDFMYG----WFMDPLTRGDY : 305
CA_CAC0820 : ---------------------AKQNGKIGITLVSNWFLPLRD-NVEDELATRRALDFQLG----WFMEPLTTGNY : 239
DC_AAF0400 : ---------------------EHQKGTIGISLHVVWVIPLSN-STSDQNATQRYLDFTCG----WFMDPLTAGRY : 313
CS_BAA1183 : ---------------------ATQNGEIGITLNSLWYEPYSK-SHEDVEAATRALDFMFG----WYMDPLVNGDY : 366
TR_CAA4005 : ---------------------ASQNGIIGITLVSHWFEPASK-EKADVDAAKRGLDFMLG----WFMHPLTKGRY : 302
PT_BAA7870 : ---------------------DDQKGIIGITTATQMAIPLND-NVANLLAASRAIDFNIG----WFLHPVVYGEY : 310
CP_AAG2589 : ------------EDQKGEIGITLESTWYVPYSD-SEADKKARDRAFDFSLG----WFLNPIVYGDYPQSMRDLVG : 301
ME_AAB7138 : ---------------------GTQKGKIGITLFTFWYEPLS-DSKVDVQAAKTALDFMFG----LWMDPMTYGRY : 282
HB_AAO4926 : ---------------------TTQNGKIGITLFTFWFEPLS-NRTIDIEASRTALDFMFG----LWMDPLTYGRY : 303
BN_CAA5791 : ---------------------KCKGDKIGIAHSPAWFEPE--DVEGGQRTVDRVLDFIMG----WHLDPTTYGDY : 310
RSMyr_BAB1 : ---------------------KFQRGKIGPVMITRWFLPFDESDPASIEAAERMNQFFHG----WYMEPLTKGRY : 317
BJMyr_AAG5 : ---------------------ADQKGKIGPVMITRWFLPYDEADPSCREAADRMNQFFHG----WYMEPLTKGKY : 317
ZM_AAB0326 : -------------------KRDDTRIGLAFDVMGRVPYGT-SFLDKQAEERSWDINLG----WFLEPVVRGDYPF : 354
ZM_AAD0985 : ---------------------KGENGRIGLAFDVMGRVPYGT-SFLDEQAKERSMDINLG----WFLEPVVRGDY : 349
SB_AAC4917 : ---------------------KGADGRIGLALNVFGRVPYTN-TFLDQQAQERSMDKCLG----WFLEPVVRGDY : 347
AS_CAA5519 : ---------------------KGDDGQIGMVLDVMAYEPYGN-NFLDQQAQERAIDFHIG----WFLEPMVRGDY : 346
SC_AAG0061 : ---------------------KHGDSKIGMAFDVMGYEPYQD-SFLDDQARERSIDYNMG----WFLEPVVRGDY : 348
Osbglu39   : ------------------------GQLMGGMLAHQLIYPATCKPRDIFCAQQYDEFLNQN----LLGVFAGQGYS : 257
Os11bglu36 : ---------------------NERKPIVGVAHHVSFTRPYGLFDVAAVALANSLTLFPYVDSICDKLDFIGINYY : 414
                                      g  g         p          a                  p   g

                      *       620         *       640         *       660         *
Os9bglu33  : PPVMRSRVGVRLPSITASDSEKIR--GSFDFIGINHYYVIFVQSIDAN--EQ--KLRDYYIDAGVQ--------- : 364
Os9bglu32  : PPVMRSRVGARLPSITASDSEKIR--GSFDFIGINHYFVIFVQSSDANH-DQ--KLRDYYVDAGVQENG------ : 368
Os9bglu31  : PPVMRKNVGSRLPSFTAEESKRVL--ESYDFVGFNHYVAIFVRADLSKL-DQ--SLRDYMGDAAVKYDLPFLKSN : 374
Os1bglu2   : PQVMKKTIGSRLPSFSQVQTELIK--GAIDFIGINHYYSAYVNYRPLVE-----GVRDYVADRSVSAR-----VR : 401
Os1bglu3   : PQVMKKAIGSRLPSFSKVQTELVK--GTLDFIGVNHYFSLYVSDLPLAK-----GVRDFIADRSVSCRGLLQGVR : 366
Os1bglu5   : PQVMKNIVGSRLPSFTKAQSEDVK--GSLDFIGMNHYYSLYVNDRPLGK-----GTRDFVADISIYYRG----SK : 365
Os5bglu22  : PEIMKKKAGSRIPSFTEEQSELIR--GSADFIGINHYTSVYISDASNGE-TV--GPRDYSADMAATFRISRN--- : 382
Os5bglu23  : PEIMKKQAGSRIPSFTKEQSELIR--GSADFIGINHYKSLYVSDGSNRE-KA--GLRDYNADMAAHFRGTT---- : 380
Os5bglu20  : PETIKKVVGSRLPFFSNHESELVT--NAFDFVGLNHYSSVYTSNNNNVV-KA--PLQDLTADIATLFRATKNDTP : 376
Os5bglu21  : PDTMKKAAGSRLPIFSNHESEMVT--NSFDFIGLNHYSSVYTSNNNNVV-KA--PLQDLTADVATLFRVTKNDTP : 380
Os5bglu19  : PETMKKAAGSRLPLFSDYESELVT--NAFDFIGLNHYTSNYVSDNSNAV-KA--PLQDVTDDISSLFWASKNSTP : 378
GM_AAL9211 : PISMKTNAGERIPAFTNHESKQVK--GSFDFIGVIHYTNLNVSDNSDAL-KN--QLRDFTADMAANIFGEDLFS- : 377
Os10bglu34 : PATMRSRVGSRLPKFTEKEAALVN--GSLDFMGINHYTTFYTKDDQS---TVIEKLLNNTLADTATISVPFRNGQ : 377
Os3bglu6   : PATMRARVGERLPRFTADEAAVVK--GALDFVGINHYTTYYTRHNNT---NIIGTLLNNTLADTGTVSLPFKNGK : 389
Os6bglu25  : PPSMQKLAGDRLPQFSTHASKLVS--GSLDFVGINHYTTLYARNDRL---RIRKLVMDDASTDSAVIPTAYRHGK : 364
Os4bglu18  : PPEMRQILGGRLPSFSPEDRRKLR--YKLDFIGVNHYTTLYARDC-----MFSDCPQGQETQHALAAVTGESNGL : 370
Os4bglu14  : PTEMREVLGQSLPKFTSKQKNRLQS-TKLDFIGLNHYTTCYVKDC-----IFSPCEIDPVNADARVFSLYERDGV : 367
Os4bglu16  : PREMREILSSNLPKFTPEEKKLLQN-NKVDFIGINHYTAIYAKDC-----IYSPCTLDTYEGNALVYAIGRRNGK : 372
PC_AAC6961 : PQEMRERLGSRLPSISSELSAKLR--GSFDYMGINHYTTLYATSTP----PLSPDHTQYLYPDSRVYLTGERHGV : 370
Os1bglu4   : PESMRQRLGDDLPTFSEKDKEFIR--NKIDFVGINHYTSRFIAHHQ----D-PEDIYFYRVQQVERIEKWN-TGE : 342
HB_AAP5105 : PEVMRKILGGGLPKFSEEDKELLR--NSLDFIGLNHYSSRFIKHVT----DSPAECYYYKAQEIERLAKWE-DGE : 351
Os7bglu26  : YSMLEIVKDRMPTFSDEESRMVK--DSIDYVGINHYTSFYMKDP-----GPWNLTPTSYQDDWHVGFAYERNGVP : 379
HV_AAA8733 : PSSMLKIVGNRLPGFSADESRMVK--GSIDYVGINQYTSYYMKDP-----GAWNQTPVSYQDDWHVGFVYERNGV : 377
Os1bglu1   : PKSLQVIVKERLPKFTADEVHMVK--GSIDYVGINQYTAYYVRDQ-----QPNATTLPSYSSDWHAAPIYERDGV : 386
LE_AAL3771 : KTMQNIVGTRLPKFSKEEVKMVK--GSFDYVGINQYTSYYMYDP-----HYTTPQPLGYQQDWNVGFAYDRKGVP : 384
Os3bglu8   : KNMRDIVKERLPTFTPEQAKLVK--GSADYFGINQYTANYMADQ-----PAPQQAATSYSSDWHVSFIFQRNGVP : 384
Os3bglu7   : QIMQDLVKDRLPKFTPEQARLVK--GSADYIGINQYTASYMKGQ-----QLMQQTPTSYSADWQVTYVFAKNGKP : 377
Os12bglu38 : ETMQNAVKERLPNFTREQSEMIK--GSADYIAINHYTTYYVS-------HHVNKTSISYLNDWDVKISYERNGVP : 363
DL_CAB3885 : PERMVKRVGKRLPCFTVEEEEMVR--GSYDFLGVNYYTTYYAINLP----IPPIAPPNYFSDMGVLSTP-TRGGV : 459
OE_AAL9361 : PESMIKYVGDRLPKFSEKEEKLVK--GSYDFLGINYYTSTYTSDDP----TKPTT-DSYFTDSHTKTSH-ERNKV : 395
CR_AAF2880 : PKSMRALVGSRLPEFSTEVSEKLT--GCYDFIGMNYYTTTYVSNADKIPDTPGYETDARINKNIFVKKV-DGKEV : 390
RS_AAF0367 : PKSMKKFVGSRLPKFSPEQSKMLK--GSYDFVGLNYYTASYVTNAS--TNSSGSNNFSYNTDIHVTYET-DRNGV : 382
Os11bglu37 : PGTMRGWLGDRLPKLTLAQSAMVK--GSYDFIGINYYTTYYAKSMPP-PNSNE---LSYDVDSRANTTG-FRNGK : 369
Os11bglu35 : PGTMRGWLGDRLPKFTPAQSAMVK--GSYDFIGINYYTTYYAKSVPP-PNSNE---LSYDVDSRANTTG-FRNGK : 373
Os9bglu30  : PGTMRGWLGNRLPEFTPEQSAMVK--GSYDFIGVNYYTTYYAKSIPP-PNSNE---LSYDLDNRANTTG-FRNGK : 371
Os9bglu29  : PGTMRGWLGARLPAFTAEQAAAVR--GSYDFIGVNYYTTYYAKSVPL-PSSNR---LSYDTDIRANTTG-FRNGK : 374
Os8bglu28  : PPAMRRLVGGRLPAFTAEQSEMLR--GSFDFIGLNYYTSNYAVAAPP-PNKLH---PSYLTDNWVNATG-YRNSI : 370
Os8bglu27  : PGTMRKLVGDRLPKFTAEQSELVK--GSYDFIGLNYYTTNYAKSVLRRPSKLK---PAYATDNWVNQTA-YRNGV : 369
CS_BAC7865 : PKSMRRLVGKRLPRFTKEQAMLVK--GSFDFLGLNYYIANYVLNVPT-SNSVN---LSYTTDSLSNQTA-FRNGV : 378
Os4bglu10  : PLSMRELVGNRLPEFSKEQSEMVK--GAFDFIGLNYYASSYADNDPPSYGHN----NSYNTDSHAKITG-SRNGI : 402
Os4bglu11  : PLSMRELVGNRLPEFSKEQSGMVK--GAFDFIGLNYYTSSYADNDPPSHGHN----NSYNTDAHAKITG-SRNGI : 379
Os4bglu9   : PLNMRELVGNRLPKFTKEQSEMVK--GAFDFIGLNYYSSSYAENVLPSYGLK----NSYNTDFHARITG-SRNGT : 379
Os4bglu13  : PLSMRELVRNRLPQFTKEQSELIK--GSFDFIGLNYYTSNYAGSLPPSNGLN----NSYSTDARANLTA-VRNGI : 375
Os4bglu12  : PLSMRGLVGNRLPQFTKEQSKLVK--GAFDFIGLNYYTANYADNLPPSNGLN----NSYTTDSRANLTG-VRNGI : 379
Os6bglu24  : PVSMRTLVGNRLPRFTKEQSKAIN--GSFDFIGLNYYTARYIQGTKQDSNSH----KSYSTDSLTNERV-ERNGT : 373
PS_AAL3907 : PQTMRSIVGSRLPNFTEEQSKSLT--GSYDYIGVNYYSARYASAYTNNYSVPT-P-PSYATDAYVNVTTTDLNGI : 389
PA_AAA9116 : PQSMRSLVKERLPNFTEEQSKSLI--GSYDYIGVNYYSARYASAYPEDYSIPT-P-PSYLTDAYVNVTT-ELNGV : 375
CA_CAC0820 : SLSMQNIVKTRLPKFTTEQSRLVN--GSFDFLGLNYYTSTYISNAPPQENVP----PSYTTDSRTNTSS-EKNGR : 307
DC_AAF0400 : PDSMQYLVGDRLPKFTTDQAKLVK--GSFDFIGLNYYTTNYATKSDASTCCP----PSYLTDPQVTLLQ-QRNGV : 381
CS_BAA1183 : PFIMRALVRDRLPFFTHAESELIK--GSYDFIGINYYTSNYAQHAPVTEDHTP---DNSYFDSYVNQSG-EKNGV : 435
TR_CAA4005 : PESMRYLVRKRLPKFSTEESKELT--GSFDFLGLNYYSSYYAAKAPRIPNARP----AIQTDSLINATF-EHNGK : 370
PT_BAA7870 : PQTMRERLGSRLPKFTEKESEMLK--QSFDFIGLNYYSTDYAAASSFSVDPVN---VSYTTDSRATLSA-IKDGV : 379
CP_AAG2589 : RRLPTFTKDETTFIM--NSFDFLGINYYTANYAKDNPSDIHPAQ----SYLNDIHATLST-DCDGIS-IGPKVSS : 368
ME_AAB7138 : PETMVDLAGDRLIGFTDEESQLLR--GSYDFVGLQYYTAYYAKPNITVDPN----FRTYKTDSGVNATPYDNNGN : 351
HB_AAO4926 : PRTVRDLIGDRLLKFTDEETQMLR--GSYDFVGIQYYTSYFAKPNAAIDPN----HRRYKTDSQIIETPYDYDGN : 372
BN_CAA5791 : PQSMKDAVGARLPKFTKAQKAKLK--GSADFVGINYYSSFYAKASEKPDYR----QPSWATDSLVEFEPKTVDGS : 379
RSMyr_BAB1 : PDIMRQIVGSRLPNFTEEEAALVA--RSYDFLGLNYYVTQYAQPKPNTYPSP---KHTAQDDAGVKLSYKNSRGE : 387
BJMyr_AAG5 : PDIMRKIVGSRLPNFTEAEAKLVA--GSYDFLGLNYYVTQYAQPKANPLLSE---KHTAMMDAGVGLTYDNSRGE : 387
ZM_AAB0326 : SMRSLARERLPFFKDEQKEKLA--GSYNMLGLNYYTSRFSKNIDISPNYSP---VLNTDDAYASQEVNGPDGKP- : 423
ZM_AAD0985 : PFSMRSLARERLPFFSDKQQEKLV--GSYNMLGINYYTSIFSKHIDISPKYSP---VLNTDDAYASQETYGPDGK : 419
SB_AAC4917 : PFSMRVSARDRVPYFKEKEQEKLV--GSYDMIGINYYTSTFSKHIDLSPNNSP---VLNTDDAYASQETKGPDGN : 417
AS_CAA5519 : PFSMRSLVGDRLPFFTKSEQEKLV--SSYDFVGINYYTSRFAKHIDISPEFIP---KINTDDVYSNPEVNDSNGI : 416
SC_AAG0061 : PFSMRSLIGDRLPMFTKEEQEKLG--SLCDIMGLNYYTSRFSKHVDISSDYTP---TLNTDDAYASSETTGSDGN : 418
Osbglu39   : PAVMAVVEQEGFGDIYRDEDLALLARTKNDFMAFSYYASKTLDSD-----------VIPEGTPVNYYLLHGEKNN : 321
Os11bglu36 : GQEVISGPGLKLVDNDEYSESGRGVYPDGLFRILIQFNERYKRLNIP---------------------------- : 461
             p           p f                 g

               680         *       700         *       720         *       740         *
Os9bglu33  : ------------GEDDTENIQCHSWSLGKVLNHLKLEYGN-----PPVMIHENGY-------SDSPDIFG----- : 410
Os9bglu32  : -----------GGGFDKEHYQLHPWALGKMLHHLKLKYGN-----PPVMIHENGD-------ADSPETPG----- : 415
Os9bglu31  : N------EF--PLGLTSDFMTSTPWALKKMLNHLQEKYKN-----PIVMIHENGA-------AGQPDPSG----- : 424
Os1bglu2   : FININMNVGN----YEPTEYPNDPKGLQLALEYLRESYGD-----FPFYIEENGK------------GST----- : 450
Os1bglu3   : FIAQTMQA--------PTRSMGDPHGLQLMLQHLKESYGD-----LPIYVQENGKLKLDLLPVFSGKASS----- : 423
Os1bglu5   : TDPPPGKAA-------PTSIGPDPQGLRLMVQYLQETYGN-----LPIYILENGY------------GSS----- : 411
Os5bglu22  : ----DTPSGQ----FVPTRLPRDPKGLQCMLEYLRDTYQG-----IPVYIQENGF---------GHFGKD----- : 430
Os5bglu23  : --------------YAPSKTLSDPKGLQCMLEYLKDTYEG-----IPVYVQENGF---------GQFDKE----- : 422
Os5bglu20  : T------------PEFLPGNTVDPQGLENALEYIRENYGN-----LTIYIQENGS------------GAP----- : 417
Os5bglu21  : T------------PVFVPGTIVDPRGLEHALKYIREKYGN-----LPIYIQENGI--------ASGSGSS----- : 425
Os5bglu19  : TRETVTWFCLLLLRQFLPGTSLDPRGLELALEYLQEKYGN-----LLFYIQENGS------------GS------ : 430
GM_AAL9211 : ----------------NEEYLITPWGLRQELNKFKLLYGN-----PPIFIHENGQ-----------RTAS----- : 415
Os10bglu34 : P-IGDRANS--------IWLYIVPRSMRILMNYVKDRYNK-----PTVYITENGMD--------DGNSPF----- : 425
Os3bglu6   : P-IGDRANS--------IWLYIVPRGMRSLMNYVKERYNS-----PPVYITENGMD--------DSNNPF----- : 437
Os6bglu25  : K-IGETAAS--------SWLHIVPWGMFKLMKHVKEKYGN-----PPVVITENGMD--------DANHPF----- : 412
Os4bglu18  : P-IGTPTAM--------PTFYVVPDGIEKMVKYFMRRYNN-----LPMFITENGYA--------QGGDSY----- : 418
Os4bglu14  : P-IGKATGA--------PFFHDVPRGMEEAVTYYKQRYNN-----TPTYITENGYS--------QASNSN----- : 415
Os4bglu16  : I-IGKPTAL--------HGYFVVPEAMEKVVMYVNDRYRN-----TTIYITENGYS--------QHSDT------ : 419
PC_AAC6961 : S-IGERTGM--------DGLFVVPHGIQKIVEYVKEFYDN-----PTIIIAENGYP--------ESEESS----- : 418
Os1bglu4   : K-IGERAAS--------EWLFIVPWGLRKLLNYAAKRYGN-----PVIYVTENGMD--------EEDDQS----- : 390
HB_AAP5105 : P-IGERAAS--------EWLYVRPWGLRKVLNYIVQRYNN-----PIIYVTENGMD--------DEDS-S----- : 398
Os7bglu26  : -IGAQANS--------YWLYIVPWGINKAVTYVKETYGN-----PTMILSENGMD--------QPGN-------- : 424
HV_AAA8733 : P-IGPRANS--------DWLYIVPWGMNKAVTYVKERYGN-----PTMILSENGMD--------QPGN------- : 423
Os1bglu1   : P-IGPRANS--------DWLYIVPWGLYKAVTYVKEKYGN-----PTMFLSENGMD--------DPGN------- : 432
LE_AAL3771 : -IGPRAHS--------YWLYIVPWGLYKAINYVKEHYGN-----PTIILAENGMD--------YAGN-------- : 429
Os3bglu8   : -IGQQANS--------NWLYIVPTGMYGAVNYIKEKYNN-----PTIIISENGMD--------QSGN-------- : 429
Os3bglu7   : -IGPQANS--------NWLYIVPWGMYGCVNYIKQKYGN-----PTVVITENGMD--------QPAN-------- : 422
Os12bglu38 : -IGKQAYS--------NWLYVVPWGIYKAVMHVKEKYKD-----PIIIIGENGID--------QPGN-------- : 408
DL_CAB3885 : P-IGIQCGQG-------GWIYIYPRGLYLILIEMTNKFKDKNDQGPLIYITENGAS--------ENANT------ : 512
OE_AAL9361 : P-IGAQAGS--------DWLYIVPWGIYRVMVDMKKRYND-----PVIYITENGVD--------EVNDK------ : 442
CR_AAF2880 : R-IGEPCYG--------GWQHVVPSGLYNLLVYTKEKYHV-----PVIYVSECGVV--------EENRTNILLTE : 443
RS_AAF0367 : P-IGPQSGS--------DWLLIYPEGIRKILVYTKKTYNV-----PLIYVTENGVD--------DVKNT------ : 429
Os11bglu37 : P-ISPQEFT--------PIFFNYPPGIREVLLYTKRRYNN-----PAIF-TENGID--------EGNNST----- : 416
Os11bglu35 : P-IGPQ-FT--------PIFFNYPPGIREVLLYTKRRYNN-----PAIYITENGID--------EGNNST----- : 420
Os9bglu30  : P-IGPQEFT--------PIFFNYPPGLRELLLYTKRRYNN-----PTIYVTENGID--------EGNNST----- : 419
Os9bglu29  : P-IGPQEFT--------PIFFNYPPGLRELLLYTKRRYNN-----PIIYVTENGIA--------EGNNKS----- : 422
Os8bglu28  : P-IGPPAYT--------PIFFNYPPGLRELLLYVKRRYNN-----PTIYITENGTD--------EANNST----- : 418
Os8bglu27  : P-IGPPAFT--------KIFFTYAPGLRELLLYTKRKYND-----PDIYIAENGTD--------EANNST----- : 417
CS_BAC7865 : A-IGRPTGV--------PAFFMYPKGLKDLLVYTKEKYND-----PVIYITENGMG--------DNNNVT----- : 426
Os4bglu10  : P-IGPQAAS--------FWFYIYPEGLRELLLHIKENYGN-----PTIYITENGVD--------EINNKT----- : 450
Os4bglu11  : P-IGPQAAS--------FWFHIYPEGICEMLLYVKENYGN-----PTIYITENGVD--------EVNNKT----- : 427
Os4bglu9   : L-IGPQAAS--------SWLHIYPQGLRELLLYIKENYGN-----PTIFITENGVD--------EVNNKT----- : 427
Os4bglu13  : P-IGPQAAS--------PWLYIYPQGFRELVLYVKENYGN-----PTIYITENGVD--------EFNNKT----- : 423
Os4bglu12  : P-IGPQAAS--------PWLYVYPQGFRDLLLYVKENYGN-----PTVYITENGVD--------EFNNKT----- : 427
Os6bglu24  : D-IGPKAGS--------SWLYIYPKGIEELLLYTKRTYNN-----PTIYITENGVD--------EVNNEN----- : 421
PS_AAL3907 : P-IGPRAAS--------DWLYVYPKGLYDLVLYTKEKYND-----PVMYITENGMD--------EFNVPK----- : 437
PA_AAA9116 : P-IGPQAAS--------DWLYVYPKGLYDLVLYTKNKYND-----PIMYITENGMD--------EFNNPK----- : 423
CA_CAC0820 : P-LGPRAAS--------SWLYVYPRGLRDLLLHIKEKYNN-----PAIYIHENGMD--------EFNDPT----- : 355
DC_AAF0400 : F-IGPVTPS--------GWMCIYPKGLRDLLLYFKEKYNN-----PLVYITENGID--------EKNDAS----- : 429
CS_BAA1183 : P-IGPLQG---------SWIYFYPRGLKELLLYVKRRYCN-----PKIYITENGTA--------EVEKEK----- : 482
TR_CAA4005 : P-LGPMAAS--------SWLCIYPQGIRKLLLYVKNHYNN-----PVIYITENGRN--------SSTIN------ : 417
PT_BAA7870 : P-IG-DPTFM-------SWLHIYPEGILTLLRYVKERYNN-----PFVMITENGMA--------DENKGSLAEDP : 432
CP_AAG2589 : S-------SWLAVYPHGLKELLIYIKEKYND-----PVIYITENGYL--------DYDSPNVDEL---------- : 413
ME_AAB7138 : L-IGPRAYSS--------WFYIFPKSIRHFLNYTKDTYND-----PVIYVTENGV---------DNYNNES---- : 399
HB_AAO4926 : L-IGPQAYSP--------WFYIFPQGIRHLLNYTKDTYNN-----PVIYITENGV---------DNLNNET---- : 420
BN_CAA5791 : VKIGSQPSTA--------KMAVYAAGLRKLVKYIKDRYGN-----PEIIITENGYG--------EDLGEKD---- : 429
RSMyr_BAB1 : F-IGPLFVED----KDNGNSYYYPKGIYYVMDYFKTKYGN-----PLIYVTENGF-----------STPDS---- : 437
BJMyr_AAG5 : F-IGPLFIED----KIAGNSYYYPKGIYYVMEYFKTQYND-----PLIYVTENGF-----------STPSS---- : 437
ZM_AAB0326 : IGPPMGN--------PWIYMYPEGLKDLLMIMKNKYGN-----PPIYITENGIG--------DVDTKETP----- : 472
ZM_AAD0985 : P-IGPPMGN--------PWIYLYPEGLKDILMIMKNKYGN-----PPIYITENGIG--------DVDTKEKP--- : 469
SB_AAC4917 : A-IGPPTGN--------AWINMYPKGLHDILMTMKNKYGN-----PPMYITENGMG--------DIDKGD----- : 465
AS_CAA5519 : P-IGPDVGM--------YFIYSYPKGLKNILLRMKEKYGN-----PPIYITENGTA--------DMDGWGN---- : 465
SC_AAG0061 : E-IGPITGT--------YWIYMYPKGLTDLLLIMKEKYGN-----PPIFITENGIA--------DVEGDP----- : 466
Osbglu39   : PYLKATEWN----------WQIDPMGFRTIITRYANDWRM------PVFPIENGIG--------VIESWDG---- : 368
Os11bglu36 : -----------------------------------------------FVITENGVS------------------- : 470
                                    p                           eng

                    760         *       780         *       800         *       820
Os9bglu33  : -------KINYNDDFRSAFLQGYLEALYLSVRNG-SNTRGYFVWSMFDMFEFLYGY-RLRFGLCGVDFTA---AA : 473
Os9bglu32  : -------KIDYDDDFRSDFLQSYLEVLHLSIRNG-SNTRGYFVWSLLDGFEFLSGY-GNRFGLCGVDFTA---PA : 478
Os9bglu31  : -------GNTYDDDFRSQYLQDYIEATLQSIRNG-SNVQGYFVWSFLDVFEYLFGY-RLRFGLYGVDFAS---PE : 487
Os1bglu2   : -------NDSLDDPDRVDYIKGYIGGVLDAIRNG-VDVRGYFVWSFVDVFELLEGY-QSRFGLYRVDFDD---GA : 513
Os1bglu3   : -------NDSLDDTDRVDYIKGYIEGVLNATRNG-VNARGYFAWFFVDMFELLSGY-QTRYGLYRVDFDD---AA : 486
Os1bglu5   : -------NDTVHDNDRVDYLKSYIGSILTALRNG-ANVKGYFVWSFVDVFEYLTGY-GQSYGLYRVDFAD---ES : 474
Os5bglu22  : -------DDSLNDTDRVDYLSSYMGSTLAALRNG-ANVKGYFVWSFLDVFELLAGY-HSPFGLHYVDFED---PN : 493
Os5bglu23  : -------D-SLNDTERVEYLSSYMGGTLAALRNG-ANVKGYFVWSFLDVFELFAGY-HSPFGLHHVDFED---PS : 484
Os5bglu20  : -------DGTLDDVERINYLQKYIAATLKAIRNG-ANVKGYSMWSFIDIYEIFGGYNSWHYGLVAVDFGS---TE : 481
Os5bglu21  : -------SETLDDVERINYLAKYIAATLKAIS-G-ANVKGYSMWSFVDLYELFGGYSTWHFGLVAVDFDS---EK : 488
Os5bglu19  : -------NATLDDVGRIDCLTQYIAATLRSIRNG-ANVKGYCVWSFMDQYEMFGDY-KAHFGIVAVDFGS---EE : 493
GM_AAL9211 : -------NSSLQDVDKGEILHGYIGSVLDALRDA-SNIKGYFRMAFPG-FVRVARWIQVSFGLYYVDRDD---PQ : 478
Os10bglu34 : ----ISLKNALKDDKRTKYHNDYLTNLADSIREDGCDVRGYFAWSLLDNWEWAAGY-TSRFGLYYVDYK----N- : 490
Os3bglu6   : ----ISIKDALKDSKRIKYHNDYLTNLAASIKEDGCDVRGYFAWSLLDNWEWAAGY-SSRFGLYFVDYKD---N- : 503
Os6bglu25  : ----SRLEDVLQDDKRIQYHNDYMSNLLDAIRKEGCNVHGYFVWSLLDNWEWNSGY-TVRFGLYYIDYKN---N- : 478
Os4bglu18  : ----TDAEDWIDDEDRIEYLEGYLTKLAKVIR-DGADVRGYFAWSVVDNFEWLFGY-TLRFGLYYIDYRT----- : 482
Os4bglu14  : ----MTAKDFTNDTGRITYIQGYLISLASAIR-KGADVRGYFVWSLLDDFEWNFGY-TLRFGLYHVHYKT----- : 479
Os4bglu16  : -----SMEDLINDVERVNYMHDYLKYLSSAIR-KGANVGGYFAWSIVDNFEWVYGY-TVKFGLYQVDFDT----- : 482
PC_AAC6961 : ----STLQENLNDVRRIRFHGDCLSYLSAAIK-NGSDVRGYFVWSLLDNFEWAFGY-TIRFGLYHVDFIS---D- : 483
Os1bglu4   : ----ATLDQVLNDTTRVGYFKGYLASVAQAIK-DGADVRGYFAWSFLDNFEWAMGY-TKRFGIVYVDYKN---G- : 455
HB_AAP5105 : ----APLHEMLDDKLRVRYFKGYLAAVAQAIK-DGADVRGYFAWSLLDNFEWAQGY-TKRFGLIYVDYKN---G- : 463
Os7bglu26  : ---VSITQGVHDTVRIRYYRNYITELKKAID-DGAKVIGYFAWSLLDNFEWRLGY-TSRFGIVYVDYKT-----L : 489
HV_AAA8733 : ----VSIADGVHDTVRIRYYRDYITELKKAID-NGARVAGYFAWSLLDNFEWRLGY-TARFGIVYVDFNT----- : 487
Os1bglu1   : ----VTIAQGVHDTTRVAYYRSYITKLKEAID-DGANCIGYFAWSLLDNFEWKLGY-TSRFGLVYVDFRT----- : 496
LE_AAL3771 : ---ITLPKALHDTKRINYYKSYLQQLKKTVD-DGANVIGYFAWSLLDNFEWRLGY-TSRFGIVYVDFNT-----L : 494
Os3bglu8   : ---LTREEFLHDTERIEFYKNYLTELKKAID-DGANVVAYFAWSLLDNFEWLSGY-TSKFGIVYVDFTT-----L : 494
Os3bglu7   : ---LSRDQYLRDTTRVHFYRSYLTQLKKAID-EGANVAGYFAWSLLDNFEWLSGY-TSKFGIVYVDFNT-----L : 487
Os12bglu38 : ---ETLPGALYDFFRIQYFDQYLHELKRAIK-DGARVTGYFAWSLLDNFEWRLGF-TSKFGIVYVDRST-----F : 473
DL_CAB3885 : ---TFTVCEARYDPIRVLYHNDHLWYLKKAMEDG-VNLKGYFIWSFADNFEWNAGY-TSRFGIFYVDFVN---GQ : 579
OE_AAL9361 : ---SKTSTEALKDDIRIHYHQEHLYYLKLAMDQG-VNVKGYFIWSLFDNFEWAAGF-SVRFGVMYVDYAN---GR : 509
CR_AAF2880 : GKTNILLTEARHDKLRVDFLQSHLASVRDAIDDG-VNVKGFFVWSFFDNFEWNLGY-ICRYGIIHVDY-----KT : 511
RS_AAF0367 : ---NLTLSEARKDSMRLKYLQDHIFNVRQAMNDG-VNVKGYFAWSLLDNFEWGEGY-GVRFGIIHIDYN----DN : 495
Os11bglu37 : ----VP--EALRDGHRIEFHSKHL-FVNHAIRNG-VNVKGYFTWTFMDCFEWGDGY-LDLFGLIYVDRK----T- : 477
Os11bglu35 : ----VP--EALRDGHRIEFHSKHLQFVNHAIRNG-VNVKGYFT-TFMDCFEWGDGY-LDRFGLIYVDRK----T- : 481
Os9bglu30  : ----LP--EALKDGHRIEFHSKHLQFVNHAIKNG-VNVKGYFTWTFMDCFEWGDGY-LDRFGLIYVDRK----T- : 481
Os9bglu29  : ----LPITEALKDGHRIEFHSKHLQFVNHAIKNG-VNVKGYFTWTFMDCFEWGDGY-LDRFGLIYIDRL----NN : 487
Os8bglu28  : ----IPISEALKDETRIGFHYKHLQFVHKAIQEG-VKVKGYFTWTFMDCFEFGDGF-KDRFGLIYVDRA----T- : 482
Os8bglu27  : ----IPIAEALKDDNRISFHYQHLRFTQLAIKEG-VKVKGYFTWTFMDDFEWGDGY-TGRFGLIYVDRE----T- : 481
CS_BAC7865 : ----TE--EGIKDPQRVYFYNQHLLSLKNAIAAG-VKVKGYFTWAFLDNFEWLSGY-TQRFGIVYVDFK----DG : 489
Os4bglu10  : ----MRLKEALKDDIRIEYYHKHLLALLSAMRDG-ANVKGYFAWSLLDNFEWSEGY-TVRFGINFVDYD----NG : 515
Os4bglu11  : ----MPLEEALKDDTRIEYYHKHLLALLSAMRDG-ANVKGYFAWSLLDNFEWAEGY-TVRFGINFVDYD----DG : 492
Os4bglu9   : ----MPLKEALNDNTRIEYYHKHLLALRNAMRDG-ANVKGYFAWSLLDNFEWADGY-TLRFGLNFVDYD----DG : 492
Os4bglu13  : ----LPLQEALKDDTRIDYYHKHLLSLLSAIRDG-ANVKGYFAWSLLDNFEWSNGY-TVRFGINFVDYN----DG : 488
Os4bglu12  : ----LPLQEALKDDARIEYYHKHLLSLLSAIRDG-ANVKGYFAWSLLDNFEWSNGY-TVRFGINFVDYN----DG : 492
Os6bglu24  : ----LSLKEALIDTTRIEFYRQHLFHVQRALRQG-VDVRGYFAWSLFDNFEWMDGY-SVRFGINYIDYK----DG : 486
PS_AAL3907 : ----LSLDEALDDANRIDYYYHHLCYLQAAIKEG-ANVQGYFAWSLLDNFEWSEGY-TVRFGINYVEYD----SG : 502
PA_AAA9116 : ----ISLEQALNDSNRIDYCYRHLCYLQEAIIEG-ANVQGYFAWSLLDNFEWSEGY-TVRFGINYVDYD----NG : 488
CA_CAC0820 : ----LPVKEALLDTFRIDYYFRHLYYIRSAIQLG-ANVKAFLAWSLFDNFEWGGGY-QHRFGLNYIDYK----DG : 420
DC_AAF0400 : ----LSLEESLIDTYRIDSYYRHLFYVRYAIRSG-ANVKGFFAWSLLDNFEWAEGY-TSRFGLYFVNYT----T- : 493
CS_BAA1183 : -------GVPLHDPERKEYLTYHLAQVLQAIREG-VRVKGHFTWALTDNFEWDKGY-TERFGLIYIDYD----KD : 544
TR_CAA4005 : -------TVTSRIPF------------------------------------------------------------ : 425
PT_BAA7870 : --------MALKDNVRIRYHREHLYYVLEAIKEG-VNVGGYYAWTWMDDFEWGSGY-TPRFGLNFVDFD----ND : 493
CP_AAG2589 : -LRDERRVKYFHDHLYYLYEAIEAG-VKVRGYFAWSLLDNFEWANGY-SMRFGLTYVDFK----NDLTRTQKDSA : 481
ME_AAB7138 : ---QPNG-EALQDDFRISYYKKHMWNALGSLKNYSVNLKGYFAWSYLDNFEWNIGY-TSRFGLYYVDYK----NN : 465
HB_AAO4926 : ---ESID-EALQDEFRVNYYRKHMWNALGSLKEYNVNIKGYFAWSYLDNFEWNIGY-TSRFGLFYVDYK----KN : 486
BN_CAA5791 : ---TDHS-VALNDHNRKYYHQRHLLSLHQAICEDKVNVTSYFVWSLMDNFEWLDGY-TARFGLYYIDFQ----NN : 495
RSMyr_BAB1 : ---ENRE-QAIADYRRIDYLCSHLCFLRKVINEKGINVRGYFAWALGDNYEFCKGF-TVRFGLSYVNWA----DL : 503
BJMyr_AAG5 : ---ENRC-EAIADYKRIDYLCSHLCFLRKVIKDRGVNVRGYFAWALGDNYEFCKGF-TVRFGLSYVNWD----DL : 503
ZM_AAB0326 : --LPMEAALNDYKRLDYIQRHIATLKESIDLG-SNVQGYFAWSLLDNFEWFAGF-TERYGIVYVDRN----NNCT : 539
ZM_AAD0985 : ----LPMEAALNDYKRLDYIQRHISTLKESIDLG-ANVHGYFAWSLLDNFEWYAGY-TERYGIVYVDRK----NN : 534
SB_AAC4917 : ----LPKPVALEDHTRLDYIQRHLSVLKQSIDLG-ADVRGYFAWSLLDNFEWSSGY-TERFGIVYVDRE----NG : 530
AS_CAA5519 : ----PPMTDPLDDPLRIEYLQQHMTAIKEAIDLGRRTLRGHFTWSLIDNFEWSLGY-LSRFGIVYIDRN----DG : 531
SC_AAG0061 : -----EMPDPLDDWKRLDYLQRHISAVKDAIDQG-ADVRGHFTWGLIDNFEWGSGY-SSRFGLVYIDKE----DG : 530
Osbglu39   : -------VNPIEDTYRIDYHRAHIDAMKAAMFEDGAEVIGYLGWGLIDILSSQGDM-RKRYGVVYVNRENHDLKD : 435
Os11bglu36 : ---------DETDLIRKPYILEHLLATYAAIIM-GVRVLGYLFWTTSDNWEWADGY-GPKFGLVAVDRANNLA-- : 532
                         d                          g   w      e   g      g

                 *       840         *       860         *       880         *       900
Os9bglu33  : RTRYLKNSARW-YSGFLRGGEL--------R-----------------PEKSYATL------------------- : 503
Os9bglu32  : RTRYVRSSARW-YSDFLNGGEL--------R-----------------PVKPFESGRY----------------- : 510
Os9bglu31  : RTRYQRHSARW-YAGFLRGGEL--------R-----------------PAAAALAGGGAYSQ------------- : 523
Os1bglu2   : R-RRAAAASEA-LRSLVLGLSQGKEGSRADCSA---------------VNRSLALANQIAQEISR---------- : 561
Os1bglu3   : LPRRAKRSARW-YRDFLKSKRQ--------------------------P-------LQIAQQ------------- : 514
Os1bglu5   : RPRQARLSARW-YSGFLKNREMD-----VD--Q---------------SELAMAAAESRAQQ------------- : 513
Os5bglu22  : LPRQPKLSAHW-YSKFLRGEIG------INIES---------------TISPDEHEHEHADQ------------- : 533
Os5bglu23  : LPRQPKLSAQW-YSKFLRSEIG------INIEK---------------MISPDEHEHAYYQ-------------- : 523
Os5bglu20  : RRRQPRRSASW-YSDFLKNNAP------IRVEM---------------APLSLQPLMLNSE-------------- : 520
Os5bglu21  : RRRQPRRSASW-YSEFLKNNSV------IRVEE---------------D--GFVSAASHAQL------------- : 526
Os5bglu19  : LTRQPRRSARW-YSDFLKNNAV------IKVDD---------------G---SVSTAFHAQL------------- : 530
GM_AAL9211 : LKKIPKLFCKNGTTGFLKGRRTS-ILDLFELEQDPITCSKSPIIFSKISKWVLASLLFLIQHKIKFMWREPLPGQ : 552
Os10bglu34 : RKRYPKNSVQW-FKNLLASSS------------------------------------------------------ : 510
Os3bglu6   : LKRYPKNSVQW-FKALLKT-------------------------------------------------------- : 521
Os6bglu25  : LTRIPKASVQW-FSQVLAQKTAI------------------------I--------------------------- : 501
Os4bglu18  : QERSPKLSALW-YKEFLQN-------------------------------LHENQ-------------------- : 505
Os4bglu14  : LKRTPKLSVDW-YRKFLTGSLLR------------------------RKFRDESQLHKFNSY------------- : 516
Os4bglu16  : QERIPRMSAKW-YRDFLTSSSLT------------------------DGLQVRSRRADS---------------- : 516
PC_AAC6961 : QKRYPKLSAQW-FRQFLQHDD-Q------------------------GSIRSSSSI------------------- : 513
Os1bglu4   : LSRHPKASARW-FSRFLKGDD----------------------------AENKADMN------------------ : 483
HB_AAP5105 : LARHPKSSAYW-FMRFLKGDE----------------------------GKNGKE-------------------- : 489
Os7bglu26  : KRYPKDSAFW-FKNMLSSKKRN----------------------------------------------------- : 510
HV_AAA8733 : LKRYPKDSALW-FKNMLSEKKRS---------------------------------------------------- : 509
Os1bglu1   : LRRYPKMSAYW-FRDLVSSKN------------------------------------------------------ : 516
LE_AAL3771 : RRYPKMSAYW-FKKLLKRQKH------------------------------------------------------ : 514
Os3bglu8   : KRYPKDSANW-FKNMLQASGPG------------------------SKSGSGTSDSQVGSATSASHPVGSAISSS : 544
Os3bglu7   : ERHPKASAYW-FRDMLKH--------------------------------------------------------- : 504
Os12bglu38 : TRYPKDSTRW-FRKMIKSEV------------------------------------------------------- : 492
DL_CAB3885 : YTRYPKSSALW-WTNFLHDVQEL------------------------KVPDTSNVTNKRSRNTEEENATAKKPKN : 629
OE_AAL9361 : YTRLPKRSAVW-WRNFLT--KPT------------------------AVPLKNEPEKSEDRRKRLRGST------ : 551
CR_AAF2880 : FQRYPKDSAIW-YKNFIS-----------------------------EGFVTNTAKKR-FREEDKLVELVKKQKY : 555
RS_AAF0367 : FARYPKDSAVW-LMNSFH-----------------------------KNISKLPAVKRSIREDDEEQVSSKRLRK : 540
Os11bglu37 : LTRYRKDSSYW-IEDFLRRQH------------------------------------------------------ : 497
Os11bglu35 : LTRYRKDSSYW-IEDFLK--------------------------------------------------------- : 498
Os9bglu30  : LKRYRKESSYW-IEDFLKRH------------------------------------------------------- : 500
Os9bglu29  : LKRYHKQSSYW-IANFLKRKKY----------------------------------------------------- : 508
Os8bglu28  : LARFRKKSSYW-FADFLRR-------------------------------------------------------- : 500
Os8bglu27  : LKRYRKKSSYW-FADFLKR-------------------------------------------------------- : 499
CS_BAC7865 : LKRYPKHSALW-FKKFLLK-------------------------------------------------------- : 507
Os4bglu10  : MKRYPKNSARW-FKKFLRK-------------------------------------------------------- : 533
Os4bglu11  : MKRYPKNSARW-FKKFLQKSNRD------------------------GNKRLKRVAYNAFSN------------- : 529
Os4bglu9   : MKRHPKNSAHW-FKKFLREMKQG---------------------------------------------------- : 514
Os4bglu13  : AKRYPKMSAHW-FKEFLQK-------------------------------------------------------- : 506
Os4bglu12  : RKRYPKNSAHW-FKKFLLK-------------------------------------------------------- : 510
Os6bglu24  : LKRYPKRSSQW-LQNFLHN-------------------------------------------------------- : 504
PS_AAL3907 : LERHSKLSKHW-FKSFLKKSSIS------------------------KKKIRRSGNTNARATKFVYQM------- : 545
PA_AAA9116 : LKRHSKLSTHW-FKNFLKRSSIS------------------------KEKIRRCGNNNARARKFVYRI------- : 531
CA_CAC0820 : LKRYPKVSAQW-YQNFLKRD------------------------------------------------------- : 439
DC_AAF0400 : LNRYPKLSATW-FKYFLARDQES------------------------AKLEILAPKARWSLSTMIKEEKTKPKRG : 543
CS_BAA1183 : FNRQPKDSTKW-FSKFLRT-------------------------------------------------------- : 562
TR_CAA4005 : --------------------------------------------------------------------------- :   -
PT_BAA7870 : LKRTPKDSYFW-FKDFLAN-------------------------------------------------------- : 511
CP_AAG2589 : KW-FLNFLTT----------------------------------------------------------------- : 490
ME_AAB7138 : LTRYPKESALW-FTKFLNISVN--------------------------ANNIFECPSKDSRKVGKFYVM------ : 507
HB_AAO4926 : LTRIPKSSAFW-FAAFLNP-ES--------------------------SKKITQTTSRNSRKVGKFYIM------ : 527
BN_CAA5791 : LTRMEKESA----TCSLNS-----------------------------SNRA----------------------- : 514
RSMyr_BAB1 : NDRNLKESGKW-YQRFISGTVKN------------------------PAKQDFLRSSLSSQSQKKR--LADA--- : 548
BJMyr_AAG5 : DDRNLKESGKW-YQRFINGTSKN------------------------PTKQDFLRSSLSFLKARRRGLLADA--- : 550
ZM_AAB0326 : RYMKESAKW-LKEFN-TAKKP-------------------------SKKILTPA--------------------- : 566
ZM_AAD0985 : YTRYMKESAKW-LKEFN-TAKKP-------------------------SKKIITPA------------------- : 563
SB_AAC4917 : CERTMKRSARW-LQEFNGAAKKV------------------------ENNKILTPAGQLN--------------- : 565
AS_CAA5519 : CKRIMKKSAKW-LKEFN-GATKK------------------------LNNKILGASSCCSGVTHGGGTA------ : 574
SC_AAG0061 : NKRKLKKSAKW-FAKFN-SVPKT------------------------LLKTTNNNATVTASVSV----------- : 568
Osbglu39   : LKRVPKKSYAW-LKQVIHTNGREM--------------------------------------------------- : 458
Os11bglu36 : --RKPRPSYFL-FSRVVTTGKITRQDRMSAWRELQQAAVQKKTRPFFRAVDKHGRMYAGGLDRPIQRPFILRDWR : 604
                       w

                      *       920         *       940
Os9bglu33  : ------------------------------------------- :   -
Os9bglu32  : ------------------------------------------- :   -
Os9bglu31  : ------------------------------------------- :   -
Os1bglu2   : ------------------------------------------- :   -
Os1bglu3   : ------------------------------------------- :   -
Os1bglu5   : ------------------------------------------- :   -
Os5bglu22  : ------------------------------------------- :   -
Os5bglu23  : ------------------------------------------- :   -
Os5bglu20  : ------------------------------------------- :   -
Os5bglu21  : ------------------------------------------- :   -
Os5bglu19  : ------------------------------------------- :   -
GM_AAL9211 : IPLKLVMF----------------------------------- : 560
Os10bglu34 : ------------------------------------------- :   -
Os3bglu6   : ------------------------------------------- :   -
Os6bglu25  : ------------------------------------------- :   -
Os4bglu18  : ------------------------------------------- :   -
Os4bglu14  : ------------------------------------------- :   -
Os4bglu16  : ------------------------------------------- :   -
PC_AAC6961 : ------------------------------------------- :   -
Os1bglu4   : ------------------------------------------- :   -
HB_AAP5105 : ------------------------------------------- :   -
Os7bglu26  : ------------------------------------------- :   -
HV_AAA8733 : ------------------------------------------- :   -
Os1bglu1   : ------------------------------------------- :   -
LE_AAL3771 : ------------------------------------------- :   -
Os3bglu8   : HRLLLPLLLSLHFLFPSFFMFLSL------------------- : 568
Os3bglu7   : ------------------------------------------- :   -
Os12bglu38 : ------------------------------------------- :   -
DL_CAB3885 : TSVNSLGFLDNLD------------------------------ : 642
OE_AAL9361 : ------------------------------------------- :   -
CR_AAF2880 : ------------------------------------------- :   -
RS_AAF0367 : ------------------------------------------- :   -
Os11bglu37 : ------------------------------------------- :   -
Os11bglu35 : ------------------------------------------- :   -
Os9bglu30  : ------------------------------------------- :   -
Os9bglu29  : ------------------------------------------- :   -
Os8bglu28  : ------------------------------------------- :   -
Os8bglu27  : ------------------------------------------- :   -
CS_BAC7865 : ------------------------------------------- :   -
Os4bglu10  : ------------------------------------------- :   -
Os4bglu11  : ------------------------------------------- :   -
Os4bglu9   : ------------------------------------------- :   -
Os4bglu13  : ------------------------------------------- :   -
Os4bglu12  : ------------------------------------------- :   -
Os6bglu24  : ------------------------------------------- :   -
PS_AAL3907 : ------------------------------------------- :   -
PA_AAA9116 : ------------------------------------------- :   -
CA_CAC0820 : ------------------------------------------- :   -
DC_AAF0400 : IEGF--------------------------------------- : 547
CS_BAA1183 : ------------------------------------------- :   -
TR_CAA4005 : ------------------------------------------- :   -
PT_BAA7870 : ------------------------------------------- :   -
CP_AAG2589 : ------------------------------------------- :   -
ME_AAB7138 : ------------------------------------------- :   -
HB_AAO4926 : ------------------------------------------- :   -
BN_CAA5791 : ------------------------------------------- :   -
RSMyr_BAB1 : ------------------------------------------- :   -
BJMyr_AAG5 : ------------------------------------------- :   -
ZM_AAB0326 : ------------------------------------------- :   -
ZM_AAD0985 : ------------------------------------------- :   -
SB_AAC4917 : ------------------------------------------- :   -
AS_CAA5519 : ------------------------------------------- :   -
SC_AAG0061 : ------------------------------------------- :   -
Osbglu39   : ------------------------------------------- :   -
Os11bglu36 : FGHYKMEGLQDPLSCFIRCIFAPFSRQKKIHYIEDDVISYSIN : 647


```
